# ASFV epitope mapping by high density peptides microarrays

**DOI:** 10.1016/j.virusres.2023.199287

**Published:** 2023-12-02

**Authors:** Cloé Desmet, Bruna Coelho-Cruz, Dora Mehn, Pascal Colpo, Ana Ruiz-Moreno

**Affiliations:** European Commission, Joint Research Centre (JRC), Ispra, Italy

**Keywords:** African swine fever, ASF, Protective antigen, Microarrays, Vaccine

## Abstract

African swine fever (ASF) is an acute, highly contagious and deadly infectious disease. It is a threat to animal health with major potential economic and societal impact. Despite decades of ASF vaccine research, still some gaps in knowledge are hindering the development of a functional vaccine. Worth mentioning are gaps in understanding the mechanism of ASF infection and immunity, as well as the fact that - in case of this disease - virus proteins, so-called protective antigens, responsible for inducing protective immune responses in pigs are not identified yet. In this paper we elaborate on a methodology to identify protective antigens based on epitope mapping by microarray technology. High density peptide microarrays, combined with fluorescence scanning, have been used to analyze the interaction of peptide sequences of African swine fever virus (ASFV) proteins with antibodies present in inactivated serum from infected and healthy animals. The study evidenced ASFV proteins already under the radar for vaccine development, such as p54, and identified specific sequences in those proteins that may become the focus for future vaccine candidates. Such methodology is amenable to automation and high-throughput and may help developing better targeting for next generation vaccines.

## Highlights

• The presence of anti-ASF antibodies in pig sera has been detected by peptide microarrays.
• Microarray-based identification of ASFV antigenic epitopes has been demonstrated.
• The methodology is a step towards the identification of ASFV protective antigens.
• Microarray technology is a promising tool to advance safe ASF vaccine development.

## Graphical abstract

**Figure:**
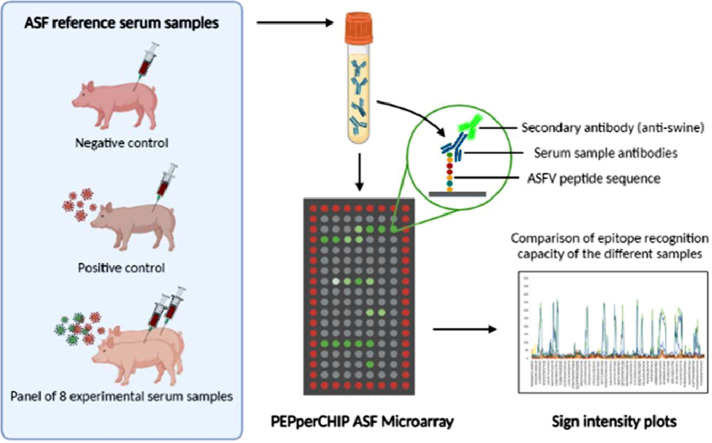

## 1 Introduction

African Swine Fever (ASF) is a devastating infectious disease that affects domestic and wild swine of all breeds and ages and that is of mandatory declaration to the World Organization for Animal Health (WOAH) (WOAH, 2023; Bosch-Camós et al., 2020; Duan et al., 2022). So far, no treatments or vaccines have been approved for ASF control, and the only available countermeasure to prevent the rapid spreading of the disease is culling of infected animals (Monoldorova et al., 2022). Being a highly contagious and lethal disease, ASF has a strong economic impact (Li et al., 2022) and represents a significant threat to the pork industry worldwide (Wang et al., 2021).

The causative agent of the disease is the African Swine Fever Virus (ASFV), a large linear double-stranded DNA arbovirus and the sole member of the *Asfarviridae* family. ASFV has a complex multiple-layer structure, consisting of a nucleoprotein core, a core shell surrounded by an internal lipid envelope, an icosahedral capsid, and an outer lipid envelope (Blome et al., 2020; Jia et al., 2017; Wang et al., 2021).

ASF was first reported in Kenya in 1921 and further spread across sub-Saharan African countries. In 1957, ASFV was detected in Portugal, and subsequently propagated to other European countries, Brazil and the Caribbean. In the 1990′s, ASFV genotype I, responsible for these first outbreaks, was eradicated in Europe (except in Sardinia) and America, after 30–40 years of efforts and containment measures. A new round of transmission started in 2007, when ASFV genotype II appeared in Georgia, then spreading to Russia, Europe and China where it killed more than 4 million pigs. ASF propagation continued, occurring in various regions of Asia, Oceania, and America. From 2020 to 2022, ASF outbreaks had been recorded in 35 different countries worldwide (Gaudreault and Richt, 2019; Monoldorova et al., 2022; Wang et al., 2022).

Currently, 24 ASFV genotypes and at least 8 serogroups have been identified (Blome et al., 2020; Gaudreault and Richt, 2019). The genotype classification is based on the sequence variation in the C-terminal of the B646L gene, encoding for p72 - a major capsid protein - (Bosch-Camós et al., 2020; Gaudreault and Richt, 2019), while serogroups are defined through hemadsorption inhibition assay and depend on hemagglutinin CD2-like protein and C-type lectin (Gaudreault and Richt, 2019; Li et al., 2022). This genetic diversity solely exists in Africa, since only strains with genotypes I and II were found outside this region (Blome et al., 2020). Interestingly, virulence and pathogenicity of a strain do not relate to its genotype (Borca et al., 2020a; Li et al., 2022).

In spite of the host's ability to develop an immune response upon infection, ASFV encodes several proteins involved in mechanisms to escape both innate and acquired immunity, which contribute to virus survival and progeny production. These include: NF-κB and IFN-I expression inhibition by the innate immune cells, impairing processes such as cytokine secretion and repression of virus growth; interference with ASFV antigen processing and presentation by dendritic cells; apoptosis and autophagy inhibition in host infected cells; and hindering of antibody based neutralization through aggregation of viral particles (Wang et al., 2021, 2022).

ASF clinical signs are highly variable and dependant on the strain's virulence, viral dose, age and immune status of the infected animals (WOAH, 2023; Li et al., 2022). Accordingly, while highly virulent strains cause acute or peracute disease with up to 100% lethality within 7–10 days, moderate virulent strains lead to an acute clinical picture with less pronounced symptoms and a mortality rate of 30–70%. As for low virulence strains, subclinical and chronic courses are induced, with unspecific symptoms and little mortality (Blome et al., 2020; Li et al., 2022).

Due to the emergence of ASFV naturally attenuated and low virulent strains that cause non-obvious symptoms, it is crucial to identify chronic and asymptomatically infected animals that might act as continuous reservoirs (WOAH, 2023; Wang et al., 2022). According to the WOAH Terrestrial Manual (“African Swine Fever (Infection With African Swine Fever Virus)” 2022), ASF detection can be achieved by direct identification of the virus, using methods such as haemadsorption test, fluorescent antibody test (FAT), conventional or real-time polymerase chain reaction, enzyme-linked immunosorbent assay (ELISA). Alternatively, serological tests for detection of specific immune response against ASFV can be used for diagnosis purposes, since anti-ASFV antibodies appear 7–10 days after infection and persist for several months or even years. These assays present several advantages: they only require serum/plasma samples, little specialised equipment and facilities. ELISA, the most widely used method for antibody detection, can be performed with validated commercial kits, which can be costly, or by using a validated procedure for assay building. The antigens used to coat ELISA's micro plates are obtained from monkey stable cells, which are infected with an adapted ASFV virus and grown in the presence of pig serum. The plate is incubated with the experimental serum sample and indirect detection is performed. Importantly, confirmatory testing of ELISA positive samples should be carried out using techniques as indirect immunoperoxidase test (IPT), indirect FAT, or immunoblotting (WOAH, 2023; Gallardo et al., 2019).

Although there is an urgent need for an ASF vaccine and despite the efforts made in the last years (Arias et al., 2017; Bosch-Camós et al., 2020; Lacasta et al., 2014; Revilla et al., 2018), some challenges are still remaining to achieve a safe, sustainable and effective vaccine against ASFV. Traditional inactivated vaccines have failed to induce protection against ASFV (Blome et al., 2014). As for live-attenuated vaccines (LAVs), some positive results have been obtained using both natural attenuated strains (Barasona et al., 2019; Gallardo et al., 2019; Leitão et al., 2001) and recombinant LAVs (Borca et al., 2021, 2020b; Chen et al., 2020; Donnell et al., 2017, 2015a, 2015b; Gladue et al., 2023; Tran et al., 2022; Zhang et al., 2021). Indeed, this type of vaccines confer a level of protection against experimental ASF infection far better than any other vaccine strategy tested so far (Bosch-Camós et al., 2020). Nevertheless, limited effect has been detected under field conditions and there are some concerns regarding their safety, due to the potential of returning to virulence and to cause chronic or persistent infections (Gaudreault and Richt, 2019; Wang et al., 2022). On the other hand, subunit vaccines arise as an ideal option for the future development of ASF vaccines, in virtue of their better safety and stability. Yet, there is still a long way to go in this field, since some gaps in the knowledge of infection and immune response mechanisms remain, and ASF protective antigens have not been identified (Bosch-Camós et al., 2020; Gaudreault and Richt, 2019).

In this sense, further characterization of humoral immune responses to viral proteins and identification of novel epitopes may be the next steps towards a better understanding of the host immune response to ASFV. This could not only contribute to the optimization of diagnostic and prognostic methods, but also for breaking ground for developing new vaccine strategies. To this end, peptide microarray-based technologies are valuable tools to study host-pathogen interactions at biomolecular level due to their potential for epitope detection at the amino acid resolution (Acharjee et al., 2023).

Microarrays are miniaturised tests consisting of arrays of biomolecules (peptides, proteins, DNA,…) immobilised on a surface at precise positions and exposed to a sample (e.g. serum, purified antibodies, …) containing complementary molecules, which may interact with the immobilised molecules. Visualization of such interactions can be achieved by fluorescence labelling and imaging. Recent arraying technologies allow positioning of thousands of different molecules on the surface of standard glass slides. Therefore, such high-density microarrays offer the advantage to simultaneously analyze multiple interactions with high throughput capability and using low sample volumes. Microarrays have been already used for investigating multiple pathogens in the last two decades (Acharjee et al., 2022; Davies et al., 2005; Natesan and Ulrich, 2010). Several fields of application include vaccine development and validation, epidemiological studies, molecular diagnostics and personalised patient response to infectious diseases.

In this study, we have applied high-density peptide microarrays to analyze the differential immune response of pigs upon infection with various ASFV strains. The presence of anti-ASFV antibodies in reference serum samples and their specific binding epitopes have been detected using a peptide microarray consisting of 15-mer sequences of ASFV proteins. By identifying the parts of antigenic ASFV proteins that are recognized by antibodies produced in infected animals, such methodology could support the development of safe and efficacious ASF vaccines.

## 2 Materials and methods

### 2.1 Materials

African swine fever reference materials (lyophilised sera) were obtained from the European Union Reference Laboratory for African Swine Fever (Madrid, Spain). The reference samples were isolated from animals infected with different strains of the virus, as listed in Table 1. One positive control (ASF-CP C + 121), one negative control (ASF-CN C-72) and a batch (ASF-Ref-1 BATCH 3) of 8 different reference sera were tested (Table 2). The inactivated and lyophilised sera were reconstituted with milliQ water and stored at -20 °C.

**Table 1:** Characteristics of the ASFV strains used to infect domestic pigs from which experimental serum samples were obtained.

Strain ID	Origin	Host	Genotype	Virulence
Est16/WB/Virus8 (Gallardo et al., 2021)	Estonia, 2016	Wild boar	II	HAD; Moderate virulence
NH/P68 (Gallardo et al., 2018; Gil et al., 2008)	Portugal, 1968		I	Non-HAD; Low virulence
Arm07 (Gallardo et al., 2018)	Armenia, 2007		II	HAD; High virulence
E75 (de Villiers et al., 2010; Portugal et al., 2015)	Spain, 1975	Domestic pig	I	HAD; Moderate virulence
Ken05/Tk1 (Bishop et al., 2015; Sánchez-Cordón et al., 2021)	Kenya, 2005	Tick from a warthog	X	Moderate virulence
L60 (Gallardo et al., 2018)	Portugal (Lisbon), 1960		I	HAD; High virulence
POL16/DP/OUT21 (Gallardo et al., 2021)	Poland, 2016	Domestic pig	II	HAD; High virulence
Es15/WB/Tartu14 (Gallardo et al., 2019)	Estonia, 2015	Wild boar	II (CVR2)	HAD; Moderate virulence

**Table 2:** Description of the serum samples provided by the European Union Reference Laboratory for African Swine Fever.

			First infection					Challenge 1			Challenge 2		
Batch ID	Sample ID	Day post infection	Genotype	ASFV isolate	Clinical form	Infection type	Virulence	ASFV isolate Challenge 1	Day post infection	Virulence	ASFV isolate Challenge 2	Day post infection	Virulence
ASF-CP	C + 121	125	II	NH/P68	C	I	A	L60	29	V	Arm07	62	V
ASF-CN	C-72	Naïve pig											
ASF-Ref-1 Batch 3	S24	Naïve pig											
	S25	26	II	Est16/WB/Viru8	SA	N	M	–	–	–	–	–	–
	S26	52	I	NH/P68	C	I	A	Arm07	43	V	–	–	–
	S27	15	I	E75	SA	I	M	–	–	–	–	–	–
	S28	70	X	Ken05/Tk1	SA	N	M	–	–	–	–	–	–
	S29	72	I	NH/P68	C	I	A	L60	29	V	Arm07	63	V
	S30	20	II	POL16/DP/OUT21	A	N	V	–	–	–	–	–	–
	S31	126	I	NH/P68	C	I	A	Arm07	30	V	–	–	–
	S32	206	II	Es15/WB/Tartu14	C	N	A	–	–	–	–	–	–

Day post infection: number of days after the first infection.

Clinical form: C: Chronic, SA: SubAcute, A: Acute.

Infection type: N: Naturally infected, I: Inoculated.

Virulence: A: Attenuated, M: Moderate, V: Virulent.

### 2.2 Methods

#### 2.2.1 Epitope mapping

Epitope mapping was performed using the PEPperCHIP African swine fever virus microarray from PEPperPRINT (Heidelberg, Germany). The microarray covers the following 12 selected antigens from African Swine Fever Virus (strain Badajoz 1971 Vero-adapted): Attachment protein p12, Cd2-like protein, B646L, E120R, B438L, Envelope protein p54, D117L, Protein E248R, BA71V-CP2475L (P220), Polyprotein pp62, Cysteine protease S273R and BA71V-K78R (P10). The antigens are translated on the microarray into 5410 linear 15-mer peptides with a 14-mer overlap (shift 1) for full epitope coverage, printed in duplicate for a total of 10,820 spots. The microarrays are framed by additional influenza virus hemagglutinin (HA) (YPYDVPDYAG) and c-Myc (EQKLISEEDL) epitope peptides as built-in positive controls (116 spots for each control).

Unless specified otherwise, each incubation step of the protocol below (adapted from Sabalza et al. 2017) was performed in a light free incubation tray at room temperature and using an orbital shaker at 140 rpm. The slides were placed into the incubation tray with the microarray surface up. 2 mL of solution was used to cover the array for each step, and the solution was removed by aspiration before the successive one. The microarrays were first equilibrated to room temperature for 30 min to avoid formation of water condensation. The following buffers were used: Phosphate-buffered saline (PBS) with 0.05% Tween20, pH 7.4 (washing buffer), Rockland Blocking Buffer MB-070 (blocking buffer), PBS with 0.05% Tween20 and 10% blocking buffer, pH 7.4 (staining buffer), 1 mM Tris buffer, pH 7 (dipping buffer).

#### 2.2.2 Pre-staining with secondary antibody

The microarrays were submitted to a pre-staining step to discriminate background interactions from sample-specific signals. They were incubated in washing buffer for 15 min, then blocked with blocking buffer for 30 min and stained with secondary anti-swine IgG Dylight680 antibodies, diluted 1:5000 in staining buffer for 45 min. Each slide was then washed 3 times for 1 min in washing buffer, removed from the incubation tray and dipped 3 times in dipping buffer. After complete drying of the surface with pressurized air stream, the microarrays were scanned to acquire fluorescence images.

#### 2.2.3 Incubation with serum sample

The arrays were re-assembled in the incubation tray and incubated in washing buffer for 15 min. The serum samples diluted 1:1000 in staining buffer were then incubated overnight at 4 °C under orbital agitation at 140 rpm. The slides were then washed 3 times for 1 min in washing buffer and stained with secondary anti-swine IgG Dylight680 antibodies, diluted 1:5000 in staining buffer for 45 min. The slides were then washed, dried and scanned as described in Section 2.2.2.

#### 2.2.4 Staining with control antibody

The arrays were re-assembled in the incubation tray and incubated in washing buffer for 15 min. A monoclonal mouse anti-HA (12CA5) DyLight800 control antibody diluted 1:2000 in staining buffer was then incubated for 45 min. The slides were then washed, dried and scanned as described in Section 2.2.2.

#### 2.2.5 Image acquisition and analysis

Fluorescence images were acquired with an InnoScan 710-IR near infrared microarray scanner, equipped with two lasers (wavelengths 670 nm and 785 nm). The peptide microarrays were scanned at both wavelengths simultaneously, with a pixel resolution of 3 µm and a scanning speed of 35 lines/*sec*. Raw fluorescence images were acquired as 16 bit .tiff files and analyzed with MAPIX software and the microarray grid file provided together with the slides. The grid is divided in five blocks of spots: four blocks for control spots that frame the array, and one central for the actual ASFV array. The whole grid was aligned on the array using the control spots and a background area was defined inside of the incubation area but outside of the spots. Spot intensities are measured in an arbitrary fluorescence intensity unit (AU). The fluorescence signal for each peptide was determined as the mean signal of measured pixels in the peptide spot, background subtracted and averaged for each two-spot duplicate.

For data visualisation, the signal intensity for each peptide was plotted linearly, for each protein of the array, as recommended by the microarray provider and presented in different application notes for epitope mapping (https://www.pepperprint.com/downloads-resources). The results obtained for the different serum samples were presented in overlaid plot for easier comparison. Data normalisation was also performed, using the maximum signal intensity obtained for each protein. The protein structural prediction obtained from Spinard et al. (2022) were modelled in 3D with the software Chimera in order to assess the responsive epitopes’ position for each of them.

## 3 Results and discussion

**Fig. 1:**
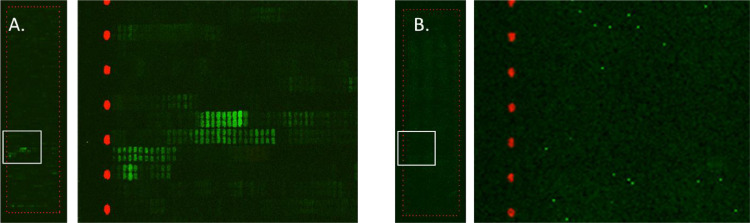
Fluorescent image of peptide array chips after reaction with A) ASF positive pig serum sample (S31) (A. right: enlarged image detail showing duplicate spots of the peptide array), and B) ASF negative pig serum sample (B. right: enlarged image detail). The spots in red are HA control spots obtained after incubation of anti-HA Dylight800 antibodies, the green ones correspond to antibodies binding to the epitope spots, subsequently incubated with an anti-pig IgG secondary antibody Dylight680 labelled.

ASF positive pig serum samples showed clear affinity responses when tested using high density peptide array chips. Fig. 1 illustrates a fluorescent image of an ASF positive sample as example of raw data obtained from the experiments, compared to a sample from a non-infected animal.

**Fig. 2:**
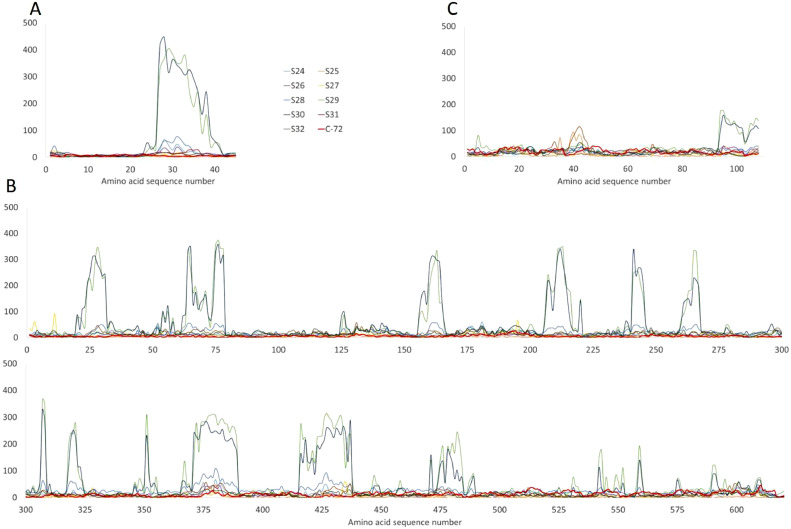
Overlaid plots of the fluorescence intensities detected for different sera at the various spots (average of replicates) with amino acid sequence numbers arranged along the x axis for (A) p12, (B) B646L (p72) and (C) E120R (p14.5). The sequence of each peptide can be found in Supplementary Table 1.

The different positive serum samples showed large variability regarding their affinity to the various proteins and their possible antibody (AB) binding epitopes. Fig. 2 shows the fluorescent intensities detected for the different serum samples at the various spots (average of replicates) as a function of the corresponding 15-mer sequences arranged along the x axis for three proteins of the chip, namely p12, p72 and p14.5. This type of representation is used for high density microarrays to present fluorescence signal as a function of the peptide sequence and thus facilitate the comparison between samples for a very high number of probes. Such overlay plots allow not only the comparison of the detected fluorescent intensities between samples regarding a single sequence, but also a better identification of possible artefacts. Wider peaks in the plot indicate higher probability of a true AB binding epitopes as the interaction between serum and peptide sequences covers a range of similar 15-mer sequences with 1 amino acid (AA) shift between them. On the other hand, sharp, smaller intensity peaks covering single sequences might be considered to be less trustable hits. Indeed, one would expect the binding affinity to decrease with the absence of an AA or short AA sequence that could be important for the interaction, but not attachments to only one 15-mer sequence.

The response of the different serum samples was analyzed after grouping to search for a correlation between type of isolate, genotype, virulence and clinical form at the time of serum sampling. The comparison between acute/sub-acute and chronic, between genotype I and genotype II, between short and long term infection (number of days post infection) samples did not show any significant trend. Virulence seems to have an impact on the global serum response on some of the arrays. For instance, the signal intensity obtained for samples S29 and S30 is 3 to 4 times higher than all of the others, while they differ in terms of genotype, clinical form and type of inoculation of ASFV, but both samples come from animals inoculated or challenged with virulent strains. However, drawing conclusions on the reason of differences between samples is challenging because of the low number of samples analyzed. In order to compare the effect of the different parameters cited previously, larger pools of serum should be analyzed. Indeed, a set of animals submitted to the same treatment would be necessary to smooth the impact of inter-individual variability.

Some observations can however be collected on the data obtained within this panel. The differences in fluorescence signal over the microarray for some of the positive samples will be described in the following paragraphs, focusing on the most significant epitopes identified. Not all of the 12 antigens present on the microarray gave a significant response to positive sera, 5 of them showed high intensity signals on different spots, corresponding to the interaction of antibodies with different 15-mer peptides. Those results will be discussed on the basis of previous findings on neutralizing antibodies and protein roles in viral infection.

Among the various ASF positive pig serum samples, incubation with S29 and S30 generated significantly higher signals on many areas of the chip compared to others (Supporting material figures). This result can be explained considering that S30 was an animal in acute phase of the disease, 20 days after natural infection, and the sample from S29 was collected 9 days after infection with a virulent strain. In these cases, the early immune reaction with high antibody levels against structural elements of the virus such as the attachment protein p12 (inner and outer envelope), the major capsid protein B646L or the N terminal domain of E120R clearly exceeds the levels found in other animals infected with a modified virus or in samples collected at later days post infection or exposure (DPI/DPE). On the other hand, the response by S26 (another sample collected at 9 DPI with virulent strain where high antibody content might be expected) is close to the one of negative control(s) for p12 and B646L. This observation and the overall high variability highlight the weight of historical immunological events probably combined with the effect of individual differences between animals and illustrates well the difficulties in selecting single proteins (or epitopes) as targets for serological assays.

Interestingly, we found both serum samples originated from animals infected with Estonian ASF isolate (S25 and S32) and the serum from S31 reacting with the EEFEPIPDYDTTST peptide sequence of E120R, an ASF capsid protein (Fig. 2C). In case of S31 and S32 the antibodies recognising the epitope are not early immune response products as the serum from these animals was collected after more than 90 days of infection/challenge. The result becomes even more thought-provoking if we consider that E120R was shown to interact with the host's immune system by blocking IFN-beta production (Liu et al., 2021) additionally to its role in the microtubule-mediated transport of virus particles from the viral factories to the plasma membrane (Andrés et al., 2001). E120R is highly conserved between ASF strains and was also suggested as target for live-attenuated vaccine development against ASF (Liu et al., 2021).

**Fig. 3:**
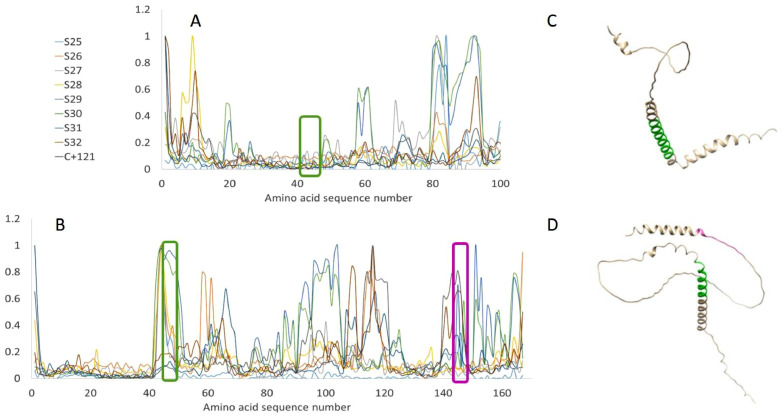
Normalized fluorescent intensities detected for different sera at the various spots (average of replicates) with corresponding amino acid sequence numbers arranged along the x axis for A) D117L (p17), B) p54. The line plots illustrate intensity values belonging to each sequence on the chip, presented in SI. C) and D) show structural models of D117L-encoded protein (p17) and p54, respectively. For p17, the transmembrane domain is highlighted in green. For p54, the AB binding epitope covering also transmembrane domain is highlighted in green while dynein binding domain is highlighted in pink.

Apart from E120R, some other proteins of the selection on the chip, which possess functions beyond their primary role as structural elements were also highlighted by our experiments. We found most of the positive serum samples reacting with certain sequences of D117L (encoding for p17) and p54, even if with high variability between samples regarding the detected maximum fluorescent intensity. In order to make the comparison easier, Fig. 3 depicts the normalised fluorescence intensities for sequences of D117L and p54.

The product of the D117L gene, p17, is another highly conserved component of the virus that is present as transmembrane protein in the inner envelop and is required for the formation of the icosahedral virus particles (Suárez et al., 2010). It is a small, but indispensable stabilising element that appears in a trimeric form between the large pseudo-hexameric blocks of the capsid built of the major capsid protein B646L (Liu et al., 2021). Moreover, p17 was shown to be involved in the immune evasion mechanisms applied by ASF (Zheng et al., 2022). As a protein involved in the regulation of the hosts immune response, it is not a surprise that D117L became also a candidate gene targeted by novel vaccines (L. Li et al., 2022). However, according to our results, the identified AB binding D117L amino acid (AA) sequences are closer to the C and N terminal of the protein and are not covering the transmembrane domain (AA 39–59, highlighted in green in Fig. 3A and C) that was found to be active in interacting with downstream mRNA expressions of certain interferons (Zheng et al., 2022). In the same study, the importance of post-translational modifications of the protein were studied, with glycosylation present in three different sites (N12, N61 and N97), but none in the transmembrane domain. In this case, the absence of signal on the transmembrane domain cannot be due to the lack of post-translational modification of the peptides on the array. Still, it is important to note a possible drawback of the use of the peptide arrays: if antibodies are produced by the infected animal against ASFV, they may not bind to the corresponding peptide if it does not present the post-translational modification such as glycosylation or phosphorylation which contribute to shaping the final structure of numerous epitopes. This comment can be enlarged to the issue of using linear peptides. Indeed, in their complete shape, viruses are composed of proteins with 3D structures that can also be arranged in multimers. These higher order structures can be essential to form epitopes, and are obviously absent when using linear peptides.

Regarding p54, our results are in line with former findings that p54 is a potent immunogen of ASF. The envelop protein p54 - similarly to B646L - is known to be a viral component that can induce neutralizing antibodies in pigs although these do not provide protection against a virulent ASFV challenge (Neilan et al., 2004). All positive serum samples in our study – including the S27 from a subacute animal - showed reaction against some sequences of p54. Among them IVLIYLFSSRKKKAA, which is highlighted in green in Fig. 3B and D, seems to be the most universally reactive peptide. This sequence partially covers the transmembrane domain of the protein (AA that is supposed to be immersed in the virion's inner envelop).

Another often selected target, the dynein binding domain of p54 (AA 149–161, highlighted in pink in Fig. 3B and D) seems to be less universal, but still reacted with many of the positive serum samples. This domain has a role in the intracellular transit of the virus through microtubule linked cargo transport, thus it is critical for the internalisation phase (Alonso et al., 2001). Apart from this, other sequences mainly on the ectodomain region of p54 were also recognised by ABs in the positive serum samples. This finding adds up to the discussion on the lack of post-translational modifications in peptide arrays. The C-terminal part of the p45 ectodomain is predicted to possess multiple glycosylation and phosphorylation sites (Sun et al., 1995). Still, the corresponding (post-translational modification free) sequence on the chip was well recognised by ABs of the ASF positive samples.

## 4 Conclusions and future perspectives

We tested the performance of a commercially available, high-density and high resolution peptide array chip in the identification of sequences of virus proteins recognised by antibodies in pig sera. The set of ASF positive samples contained heat inactivated, lyophilised serum from various serologically positive animals, including subacute, acute and chronic phase infections, collected at various time points (days after exposure or inoculation). Negative control sera from healthy animals with negative ASF antibody diagnostic conclusion were also involved in the study.

Our findings show that the peptide array chip combined with fluorescence detection is an efficient tool in identifying possible targets for ASF serological assay or even vaccine development. Despite the fact that inter-individual differences might strongly affect the recognition of individual epitopes in serological assays, the selection of a limited set of peptides that in combination might provide a reliable single response becomes extremely efficient. The use of high resolution peptide arrays can pave the road to developing cheap but efficient peptide-set based ELISA assays for ASF antibody detection in serum and in even more challenging samples, e.g. meat exudate, which could be useful for trade controls and disease surveillance programmes.

Our experiments clearly identified ASF proteins that are already selected targets in vaccine development and highlighted sequences that might become future candidates. The drawbacks of peptide arrays, i.e. not considering post-translational modifications, like glycosylation and missing the direct recognition of non-linear epitopes is balanced by the high throughput and the immense amount of information gained from the results.

## CRediT authorship contribution statement

**Cloé Desmet:** Formal analysis, Investigation, Methodology, Writing – original draft. **Bruna Coelho-Cruz:** Formal analysis, Investigation, Writing – original draft. **Dora Mehn:** Formal analysis, Investigation, Writing – original draft. **Pascal Colpo:** Writing – review & editing. **Ana Ruiz-Moreno:** Conceptualization, Methodology, Writing – review & editing, Supervision.

## Declaration of Competing Interest

The authors declare that they have no known competing financial interests or personal relationships that could have appeared to influence the work reported in this paper.

## Data Availability

Data will be made available on request.

## Appendix Supplementary materials

Image, application 1

Image, application 2
