# Supplementary material for: ASFV epitope mapping by high density peptides microarrays
Source: Virus Res. 2023 Dec 2;339:199287. doi: 10.1016/j.virusres.2023.199287 (PMC10711508; doi:10.1016/j.virusres.2023.199287)

**Supplementary information -** **Supplementary Figure 1.** Overlaid plots of the fluorescence intensities detected for different sera at the various spots (average of replicates) with amino acid sequence numbers arranged along the x axis for Cd2-like protein, E248R, pp62, p10, S273R, B438L and p220 . The sequence of each peptide can be found in Supplementary Table 1.


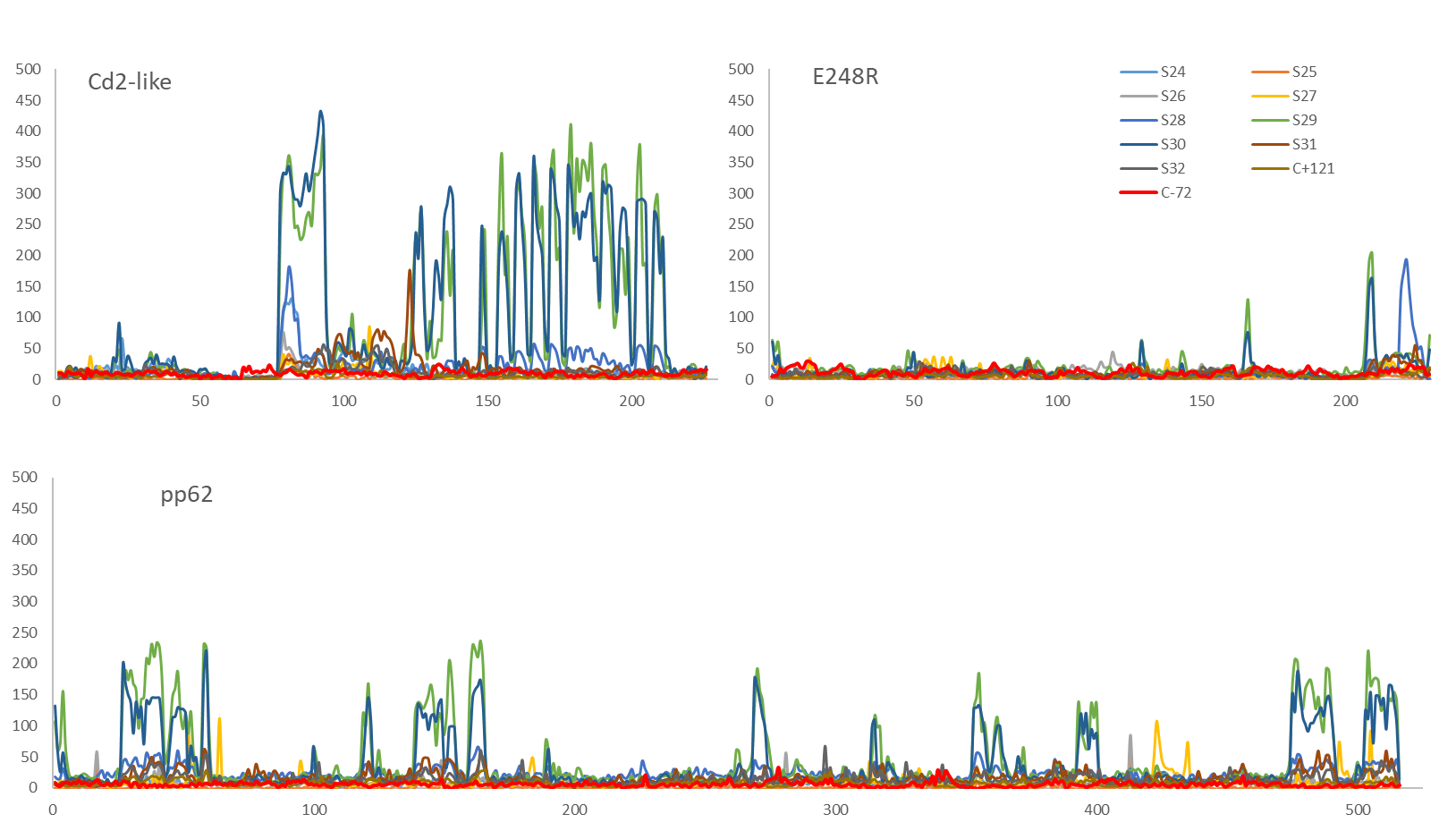


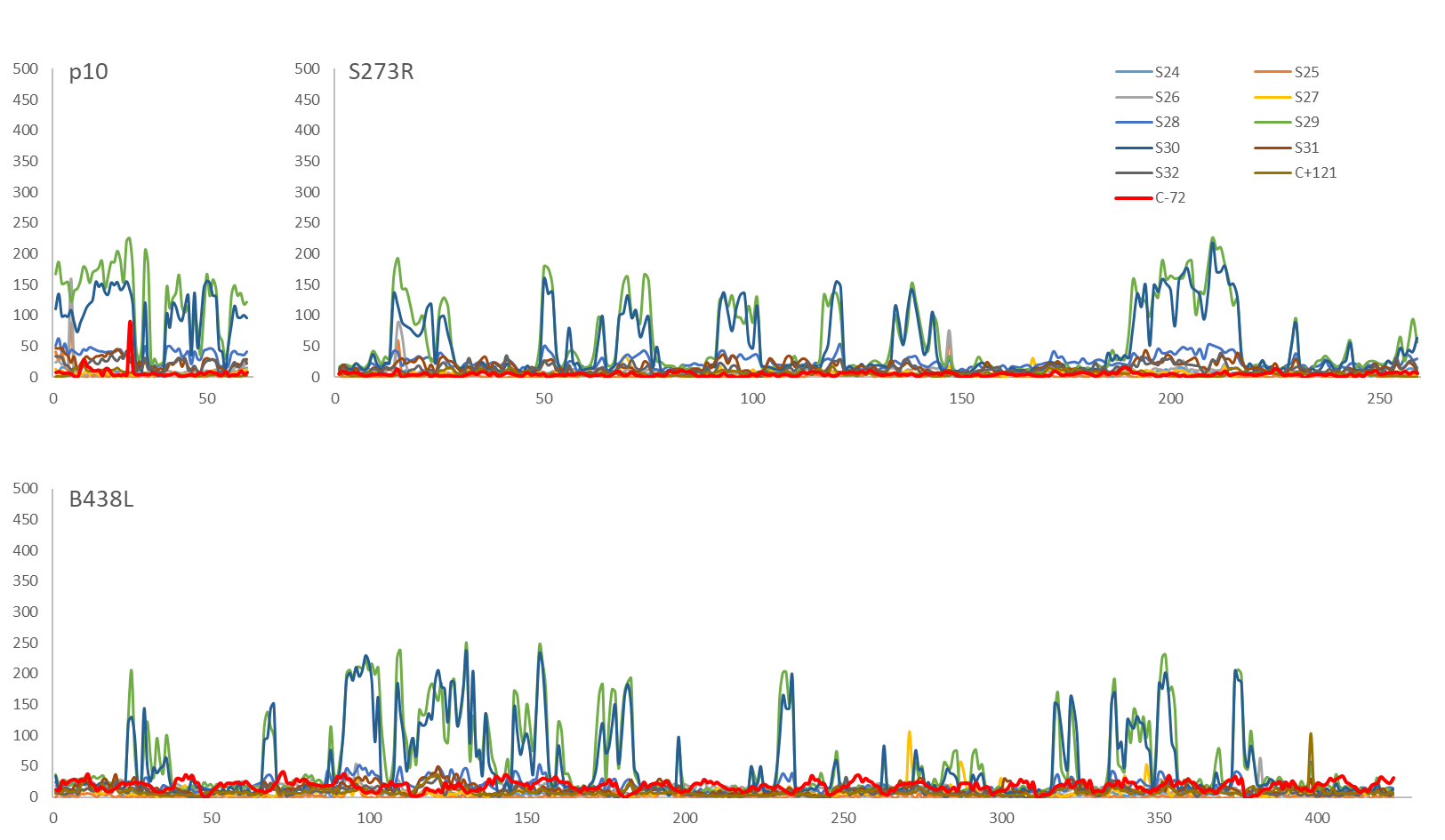


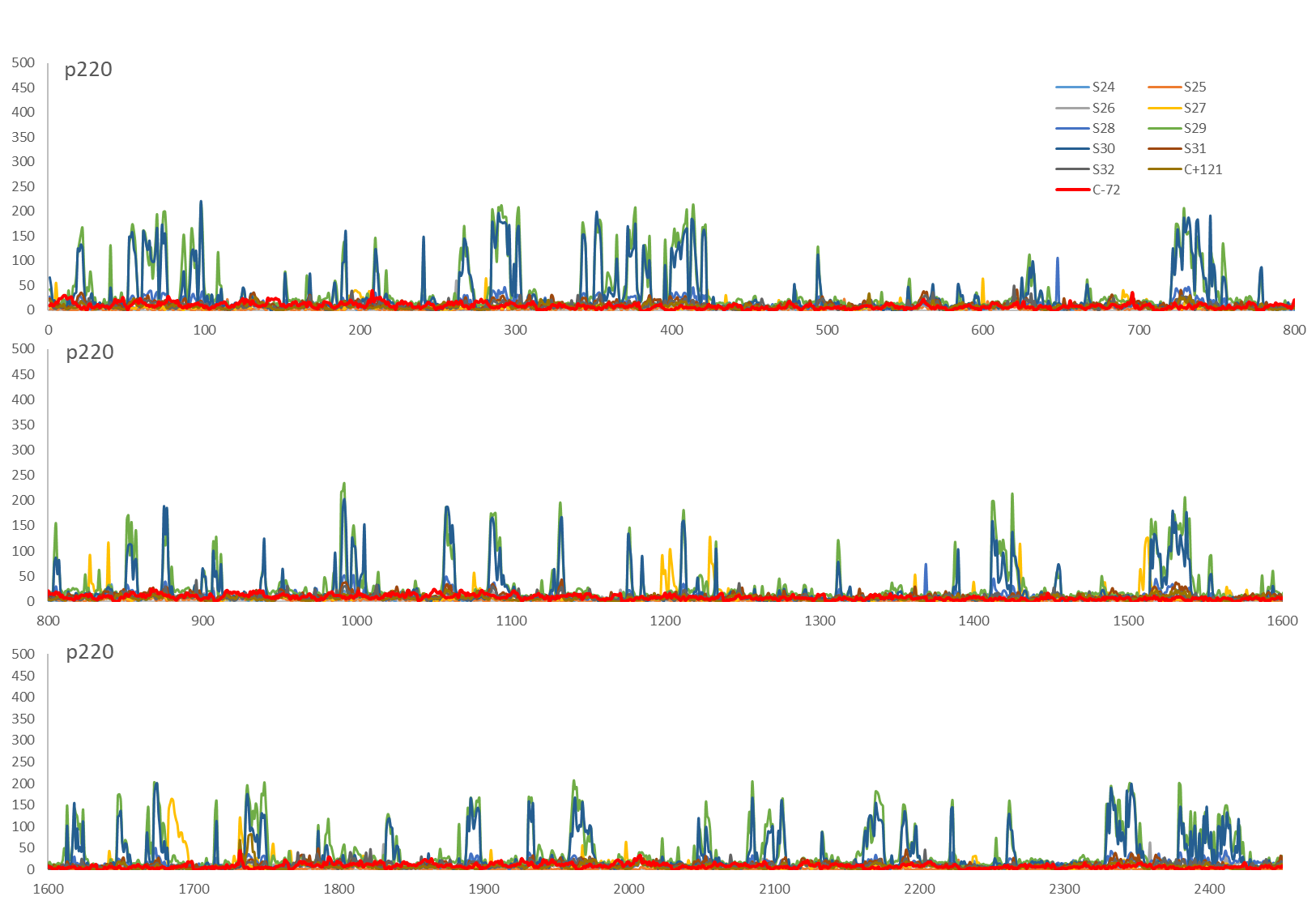

Supplement: Supplementary file 1 [file mmc1.docx]
