# Supplementary material for: ASFV epitope mapping by high density peptides microarrays
Source: Virus Res. 2023 Dec 2;339:199287. doi: 10.1016/j.virusres.2023.199287 (PMC10711508; doi:10.1016/j.virusres.2023.199287)
Supplement: Supplementary file 2 [file mmc2.pdf]

Supplementary information - Table 1. Amino acid sequences

| Protein | Gene | Localization | Sequence number | Peptide |
|---|---|---|---|---|
| Attachment protein p12 | O61R | Outer and inner envelope | 1 | GSGSGSGMALDGSSG |
| Attachment protein p12 | O61R | Outer and inner envelope | 2 | SGSGSGMALDGSSGG |
| Attachment protein p12 | O61R | Outer and inner envelope | 3 | GSGSGMALDGSSGGG |
| Attachment protein p12 | O61R | Outer and inner envelope | 4 | SGSGMALDGSSGGGS |
| Attachment protein p12 | O61R | Outer and inner envelope | 5 | GSGMALDGSSGGGSN |
| Attachment protein p12 | O61R | Outer and inner envelope | 6 | SGMALDGSSGGGSNV |
| Attachment protein p12 | O61R | Outer and inner envelope | 7 | GMALDGSSGGGSNVE |
| Attachment protein p12 | O61R | Outer and inner envelope | 8 | MALDGSSGGGSNVET |
| Attachment protein p12 | O61R | Outer and inner envelope | 9 | ALDGSSGGGSNVETL |
| Attachment protein p12 | O61R | Outer and inner envelope | 10 | LDGSSGGGSNVETLL |
| Attachment protein p12 | O61R | Outer and inner envelope | 11 | DGSSGGGSNVETLLI |
| Attachment protein p12 | O61R | Outer and inner envelope | 12 | GSSGGGSNVETLLIV |
| Attachment protein p12 | O61R | Outer and inner envelope | 13 | SSGGGSNVETLLIVA |
| Attachment protein p12 | O61R | Outer and inner envelope | 14 | SGGGSNVETLLIVAI |
| Attachment protein p12 | O61R | Outer and inner envelope | 15 | GGGSNVETLLIVAII |
| Attachment protein p12 | O61R | Outer and inner envelope | 16 | GGSNVETLLIVAIIV |
| Attachment protein p12 | O61R | Outer and inner envelope | 17 | GSNVETLLIVAIIVV |
| Attachment protein p12 | O61R | Outer and inner envelope | 18 | SNVETLLIVAIIVVI |
| Attachment protein p12 | O61R | Outer and inner envelope | 19 | NVETLLIVAIIVVIM |
| Attachment protein p12 | O61R | Outer and inner envelope | 20 | VETLLIVAIIVVIMA |
| Attachment protein p12 | O61R | Outer and inner envelope | 21 | ETLLIVAIIVVIMAI |
| Attachment protein p12 | O61R | Outer and inner envelope | 22 | TLLIVAIIVVIMAIM |
| Attachment protein p12 | O61R | Outer and inner envelope | 23 | LLIVAIIVVIMAIML |
| Attachment protein p12 | O61R | Outer and inner envelope | 24 | LIVAIIVVIMAIMLY |
| Attachment protein p12 | O61R | Outer and inner envelope | 25 | IVAIIVVIMAIMLYY |
| Attachment protein p12 | O61R | Outer and inner envelope | 26 | VAIIVVIMAIMLYYF |
| Attachment protein p12 | O61R | Outer and inner envelope | 27 | AIIVVIMAIMLYYFW |
| Attachment protein p12 | O61R | Outer and inner envelope | 28 | IIVVIMAIMLYYFWW |
| Attachment protein p12 | O61R | Outer and inner envelope | 29 | IVVIMAIMLYYFWWM |
| Attachment protein p12 | O61R | Outer and inner envelope | 30 | VVIMAIMLYYFWWMP |
| Attachment protein p12 | O61R | Outer and inner envelope | 31 | VIMAIMLYYFWWMPR |
| Attachment protein p12 | O61R | Outer and inner envelope | 32 | IMAIMLYYFWWMPRQ |
| Attachment protein p12 | O61R | Outer and inner envelope | 33 | MAIMLYYFWWMPRQQ |
| Attachment protein p12 | O61R | Outer and inner envelope | 34 | AIMLYYFWWMPRQQK |
| Attachment protein p12 | O61R | Outer and inner envelope | 35 | IMLYYFWWMPRQQKK |
| Attachment protein p12 | O61R | Outer and inner envelope | 36 | MLYYFWWMPRQQKKC |
| Attachment protein p12 | O61R | Outer and inner envelope | 37 | LYYFWWMPRQQKKCS |
| Attachment protein p12 | O61R | Outer and inner envelope | 38 | YYFWWMPRQQKKCSK |
| Attachment protein p12 | O61R | Outer and inner envelope | 39 | YFWWMPRQQKKCSKA |
| Attachment protein p12 | O61R | Outer and inner envelope | 40 | FWWMPRQQKKCSKAE |
| Attachment protein p12 | O61R | Outer and inner envelope | 41 | WWMPRQQKKCSKAEE |
| Attachment protein p12 | O61R | Outer and inner envelope | 42 | WMPRQQKKCSKAEEC |
| Attachment protein p12 | O61R | Outer and inner envelope | 43 | MPRQQKKCSKAEECT |
| Attachment protein p12 | O61R | Outer and inner envelope | 44 | PRQQKKCSKAEECTC |
| Attachment protein p12 | O61R | Outer and inner envelope | 45 | RQQKKCSKAEECTCN |
| Attachment protein p12 | O61R | Outer and inner envelope | 46 | QQKKCSKAEECTCNN |
| Attachment protein p12 | O61R | Outer and inner envelope | 47 | QKKCSKAEECTCNNG |
| Attachment protein p12 | O61R | Outer and inner envelope | 48 | KKCSKAEECTCNNGS |
| Attachment protein p12 | O61R | Outer and inner envelope | 49 | KCSKAEECTCNNGSC |
| Attachment protein p12 | O61R | Outer and inner envelope | 50 | CSKAEECTCNNGSCS |
| Attachment protein p12 | O61R | Outer and inner envelope | 51 | SKAEECTCNNGSCSL |
| Attachment protein p12 | O61R | Outer and inner envelope | 52 | KAEECTCNNGSCSLK |
| Attachment protein p12 | O61R | Outer and inner envelope | 53 | AEECTCNNGSCSLKT |
| Attachment protein p12 | O61R | Outer and inner envelope | 54 | EECTCNNGSCSLKTS |
| Attachment protein p12 | O61R | Outer and inner envelope | 55 | ECTCNNGSCSLKTSG |
| Attachment protein p12 | O61R | Outer and inner envelope | 56 | CTCNNGSCSLKTSGS |
| Attachment protein p12 | O61R | Outer and inner envelope | 57 | TCNNGSCSLKTSGSG |
| Attachment protein p12 | O61R | Outer and inner envelope | 58 | CNNGSCSLKTSGSGS |
| Attachment protein p12 | O61R | Outer and inner envelope | 59 | NNGSCSLKTSGSGSG |
| Attachment protein p12 | O61R | Outer and inner envelope | 60 | NGSCSLKTSGSGSGS |
| Attachment protein p12 | O61R | Outer and inner envelope | 61 | GSCSLKTSGSGSGSG |
| Cd2-like protein | EP402R | Outer envelope | 1 | GSGSGSGVNIYLIIN |
| Cd2-like protein | EP402R | Outer envelope | 2 | SGSGSGVNIYLIINN |
| Cd2-like protein | EP402R | Outer envelope | 3 | GSGSGVNIYLIINNT |

| | | | | |
|---|---|---|---|---|
| Cd2-like protein | EP402R | Outer envelope | 4 | SGSGVNIYLIINNTI |
| Cd2-like protein | EP402R | Outer envelope | 5 | GSGVNIYLIINNTIV |
| Cd2-like protein | EP402R | Outer envelope | 6 | SGVNIYLIINNTIVN |
| Cd2-like protein | EP402R | Outer envelope | 7 | GVNIYLIINNTIVND |
| Cd2-like protein | EP402R | Outer envelope | 8 | VNIYLIINNTIVNDT |
| Cd2-like protein | EP402R | Outer envelope | 9 | NIYLIINNTIVNDTN |
| Cd2-like protein | EP402R | Outer envelope | 10 | IYLIINNTIVNDTNG |
| Cd2-like protein | EP402R | Outer envelope | 11 | YLIINNTIVNDTNGD |
| Cd2-like protein | EP402R | Outer envelope | 12 | LIINNTIVNDTNGDI |
| Cd2-like protein | EP402R | Outer envelope | 13 | IINNTIVNDTNGDIL |
| Cd2-like protein | EP402R | Outer envelope | 14 | INNTIVNDTNGDILN |
| Cd2-like protein | EP402R | Outer envelope | 15 | NNTIVNDTNGDILNY |
| Cd2-like protein | EP402R | Outer envelope | 16 | NTIVNDTNGDILNYY |
| Cd2-like protein | EP402R | Outer envelope | 17 | TIVNDTNGDILNYYW |
| Cd2-like protein | EP402R | Outer envelope | 18 | IVNDTNGDILNYYWN |
| Cd2-like protein | EP402R | Outer envelope | 19 | VNDTNGDILNYYWNG |
| Cd2-like protein | EP402R | Outer envelope | 20 | NDTNGDILNYYWNGN |
| Cd2-like protein | EP402R | Outer envelope | 21 | DTNGDILNYYWNGNN |
| Cd2-like protein | EP402R | Outer envelope | 22 | TNGDILNYYWNGNNN |
| Cd2-like protein | EP402R | Outer envelope | 23 | NGDILNYYWNGNNNF |
| Cd2-like protein | EP402R | Outer envelope | 24 | GDILNYYWNGNNNFT |
| Cd2-like protein | EP402R | Outer envelope | 25 | DILNYYWNGNNNFTA |
| Cd2-like protein | EP402R | Outer envelope | 26 | ILNYYWNGNNNFTAT |
| Cd2-like protein | EP402R | Outer envelope | 27 | LNYYWNGNNNFTATC |
| Cd2-like protein | EP402R | Outer envelope | 28 | NYYWNGNNNFTATCM |
| Cd2-like protein | EP402R | Outer envelope | 29 | YYWNGNNNFTATCMI |
| Cd2-like protein | EP402R | Outer envelope | 30 | YWNGNNNFTATCMIN |
| Cd2-like protein | EP402R | Outer envelope | 31 | WNGNNNFTATCMINN |
| Cd2-like protein | EP402R | Outer envelope | 32 | NGNNNFTATCMINNT |
| Cd2-like protein | EP402R | Outer envelope | 33 | GNNNFTATCMINNTI |
| Cd2-like protein | EP402R | Outer envelope | 34 | NNNFTATCMINNTIS |
| Cd2-like protein | EP402R | Outer envelope | 35 | NNFTATCMINNTISS |
| Cd2-like protein | EP402R | Outer envelope | 36 | NFTATCMINNTISSL |
| Cd2-like protein | EP402R | Outer envelope | 37 | FTATCMINNTISSLN |
| Cd2-like protein | EP402R | Outer envelope | 38 | TATCMINNTISSLNE |
| Cd2-like protein | EP402R | Outer envelope | 39 | ATCMINNTISSLNET |
| Cd2-like protein | EP402R | Outer envelope | 40 | TCMINNTISSLNETE |
| Cd2-like protein | EP402R | Outer envelope | 41 | CMINNTISSLNETEN |
| Cd2-like protein | EP402R | Outer envelope | 42 | MINNTISSLNETENI |
| Cd2-like protein | EP402R | Outer envelope | 43 | INNTISSLNETENIN |
| Cd2-like protein | EP402R | Outer envelope | 44 | NNTISSLNETENINC |
| Cd2-like protein | EP402R | Outer envelope | 45 | NTISSLNETENINCT |
| Cd2-like protein | EP402R | Outer envelope | 46 | TISSLNETENINCTN |
| Cd2-like protein | EP402R | Outer envelope | 47 | ISSLNETENINCTNP |
| Cd2-like protein | EP402R | Outer envelope | 48 | SSLNETENINCTNPI |
| Cd2-like protein | EP402R | Outer envelope | 49 | SLNETENINCTNPIL |
| Cd2-like protein | EP402R | Outer envelope | 50 | LNETENINCTNPILK |
| Cd2-like protein | EP402R | Outer envelope | 51 | NETENINCTNPILKY |
| Cd2-like protein | EP402R | Outer envelope | 52 | ETENINCTNPILKYQ |
| Cd2-like protein | EP402R | Outer envelope | 53 | TENINCTNPILKYQN |
| Cd2-like protein | EP402R | Outer envelope | 54 | ENINCTNPILKYQNY |
| Cd2-like protein | EP402R | Outer envelope | 55 | NINCTNPILKYQNYL |
| Cd2-like protein | EP402R | Outer envelope | 56 | INCTNPILKYQNYLS |
| Cd2-like protein | EP402R | Outer envelope | 57 | NCTNPILKYQNYLST |
| Cd2-like protein | EP402R | Outer envelope | 58 | CTNPILKYQNYLSTL |
| Cd2-like protein | EP402R | Outer envelope | 59 | TNPILKYQNYLSTLF |
| Cd2-like protein | EP402R | Outer envelope | 60 | NPILKYQNYLSTLFY |
| Cd2-like protein | EP402R | Outer envelope | 61 | PILKYQNYLSTLFYI |
| Cd2-like protein | EP402R | Outer envelope | 62 | ILKYQNYLSTLFYII |
| Cd2-like protein | EP402R | Outer envelope | 63 | LKYQNYLSTLFYIII |
| Cd2-like protein | EP402R | Outer envelope | 64 | KYQNYLSTLFYIIIF |
| Cd2-like protein | EP402R | Outer envelope | 65 | YQNYLSTLFYIIIFI |
| Cd2-like protein | EP402R | Outer envelope | 66 | QNYLSTLFYIIIFIV |
| Cd2-like protein | EP402R | Outer envelope | 67 | NYLSTLFYIIIFIVS |
| Cd2-like protein | EP402R | Outer envelope | 68 | YLSTLFYIIIFIVSG |
| Cd2-like protein | EP402R | Outer envelope | 69 | LSTLFYIIIFIVSGL |
| Cd2-like protein | EP402R | Outer envelope | 70 | STLFYIIIFIVSGLI |

| | | | | |
|---|---|---|---|---|
| Cd2-like protein | EP402R | Outer envelope | 71 | TLFYIIIFIVSGLII |
| Cd2-like protein | EP402R | Outer envelope | 72 | LFYIIIFIVSGLIIG |
| Cd2-like protein | EP402R | Outer envelope | 73 | FYIIIFIVSGLIIGI |
| Cd2-like protein | EP402R | Outer envelope | 74 | YIIIFIVSGLIIGIF |
| Cd2-like protein | EP402R | Outer envelope | 75 | IIIFIVSGLIIGIFI |
| Cd2-like protein | EP402R | Outer envelope | 76 | IIFIVSGLIIGIFIS |
| Cd2-like protein | EP402R | Outer envelope | 77 | IFIVSGLIIGIFISI |
| Cd2-like protein | EP402R | Outer envelope | 78 | FIVSGLIIGIFISII |
| Cd2-like protein | EP402R | Outer envelope | 79 | IVSGLIIGIFISIIS |
| Cd2-like protein | EP402R | Outer envelope | 80 | VSGLIIGIFISIISV |
| Cd2-like protein | EP402R | Outer envelope | 81 | SGLIIGIFISIISVL |
| Cd2-like protein | EP402R | Outer envelope | 82 | GLIIGIFISIISVLS |
| Cd2-like protein | EP402R | Outer envelope | 83 | LIIGIFISIISVLSI |
| Cd2-like protein | EP402R | Outer envelope | 84 | IIGIFISIISVLSIR |
| Cd2-like protein | EP402R | Outer envelope | 85 | IGIFISIISVLSIRR |
| Cd2-like protein | EP402R | Outer envelope | 86 | GIFISIISVLSIRRK |
| Cd2-like protein | EP402R | Outer envelope | 87 | IFISIISVLSIRRKR |
| Cd2-like protein | EP402R | Outer envelope | 88 | FISIISVLSIRRKRK |
| Cd2-like protein | EP402R | Outer envelope | 89 | ISIISVLSIRRKRKK |
| Cd2-like protein | EP402R | Outer envelope | 90 | SIISVLSIRRKRKKH |
| Cd2-like protein | EP402R | Outer envelope | 91 | IISVLSIRRKRKKHV |
| Cd2-like protein | EP402R | Outer envelope | 92 | ISVLSIRRKRKKHVE |
| Cd2-like protein | EP402R | Outer envelope | 93 | SVLSIRRKRKKHVEE |
| Cd2-like protein | EP402R | Outer envelope | 94 | VLSIRRKRKKHVEEI |
| Cd2-like protein | EP402R | Outer envelope | 95 | LSIRRKRKKHVEEIE |
| Cd2-like protein | EP402R | Outer envelope | 96 | SIRRKRKKHVEEIES |
| Cd2-like protein | EP402R | Outer envelope | 97 | IRRKRKKHVEEIESP |
| Cd2-like protein | EP402R | Outer envelope | 98 | RRKRKKHVEEIESPP |
| Cd2-like protein | EP402R | Outer envelope | 99 | RKRKKHVEEIESPPP |
| Cd2-like protein | EP402R | Outer envelope | 100 | KRKKHVEEIESPPPS |
| Cd2-like protein | EP402R | Outer envelope | 101 | RKKHVEEIESPPPSE |
| Cd2-like protein | EP402R | Outer envelope | 102 | KKHVEEIESPPPSES |
| Cd2-like protein | EP402R | Outer envelope | 103 | KHVEEIESPPPSESN |
| Cd2-like protein | EP402R | Outer envelope | 104 | HVEEIESPPPSESNE |
| Cd2-like protein | EP402R | Outer envelope | 105 | VEEIESPPPSESNEE |
| Cd2-like protein | EP402R | Outer envelope | 106 | EEIESPPPSESNEED |
| Cd2-like protein | EP402R | Outer envelope | 107 | EIESPPPSESNEEDI |
| Cd2-like protein | EP402R | Outer envelope | 108 | IESPPPSESNEEDIS |
| Cd2-like protein | EP402R | Outer envelope | 109 | ESPPPSESNEEDISH |
| Cd2-like protein | EP402R | Outer envelope | 110 | SPPPSESNEEDISHD |
| Cd2-like protein | EP402R | Outer envelope | 111 | PPPSESNEEDISHDD |
| Cd2-like protein | EP402R | Outer envelope | 112 | PPSESNEEDISHDDT |
| Cd2-like protein | EP402R | Outer envelope | 113 | PSESNEEDISHDDTT |
| Cd2-like protein | EP402R | Outer envelope | 114 | SESNEEDISHDDTTS |
| Cd2-like protein | EP402R | Outer envelope | 115 | ESNEEDISHDDTTSI |
| Cd2-like protein | EP402R | Outer envelope | 116 | SNEEDISHDDTTSIH |
| Cd2-like protein | EP402R | Outer envelope | 117 | NEEDISHDDTTSIHE |
| Cd2-like protein | EP402R | Outer envelope | 118 | EEDISHDDTTSIHEP |
| Cd2-like protein | EP402R | Outer envelope | 119 | EDISHDDTTSIHEPS |
| Cd2-like protein | EP402R | Outer envelope | 120 | DISHDDTTSIHEPSP |
| Cd2-like protein | EP402R | Outer envelope | 121 | ISHDDTTSIHEPSPR |
| Cd2-like protein | EP402R | Outer envelope | 122 | SHDDTTSIHEPSPRE |
| Cd2-like protein | EP402R | Outer envelope | 123 | HDDTTSIHEPSPREP |
| Cd2-like protein | EP402R | Outer envelope | 124 | DDTTSIHEPSPREPL |
| Cd2-like protein | EP402R | Outer envelope | 125 | DTTSIHEPSPREPLL |
| Cd2-like protein | EP402R | Outer envelope | 126 | TTSIHEPSPREPLLP |
| Cd2-like protein | EP402R | Outer envelope | 127 | TSIHEPSPREPLLPK |
| Cd2-like protein | EP402R | Outer envelope | 128 | SIHEPSPREPLLPKP |
| Cd2-like protein | EP402R | Outer envelope | 129 | IHEPSPREPLLPKPY |
| Cd2-like protein | EP402R | Outer envelope | 130 | HEPSPREPLLPKPYS |
| Cd2-like protein | EP402R | Outer envelope | 131 | EPSPREPLLPKPYSR |
| Cd2-like protein | EP402R | Outer envelope | 132 | PSPREPLLPKPYSRY |
| Cd2-like protein | EP402R | Outer envelope | 133 | SPREPLLPKPYSRYQ |
| Cd2-like protein | EP402R | Outer envelope | 134 | PREPLLPKPYSRYQY |
| Cd2-like protein | EP402R | Outer envelope | 135 | REPLLPKPYSRYQYN |
| Cd2-like protein | EP402R | Outer envelope | 136 | EPLLPKPYSRYQYNT |
| Cd2-like protein | EP402R | Outer envelope | 137 | PLLPKPYSRYQYNTP |

| | | | |
|---|---|---|---|
| Cd2-like protein | EP402R | Outer envelope | 138 LLPKPYSRYQYNTPI |
| Cd2-like protein | EP402R | Outer envelope | 139 LPKPYSRYQYNTPIY |
| Cd2-like protein | EP402R | Outer envelope | 140 PKPYSRYQYNTPIYY |
| Cd2-like protein | EP402R | Outer envelope | 141 KPYSRYQYNTPIYYM |
| Cd2-like protein | EP402R | Outer envelope | 142 PYSRYQYNTPIYYMR |
| Cd2-like protein | EP402R | Outer envelope | 143 YSRYQYNTPIYYMRP |
| Cd2-like protein | EP402R | Outer envelope | 144 SRYQYNTPIYYMRPS |
| Cd2-like protein | EP402R | Outer envelope | 145 RYQYNTPIYYMRPST |
| Cd2-like protein | EP402R | Outer envelope | 146 YQYNTPIYYMRPSTQ |
| Cd2-like protein | EP402R | Outer envelope | 147 QYNTPIYYMRPSTQP |
| Cd2-like protein | EP402R | Outer envelope | 148 YNTPIYYMRPSTQPL |
| Cd2-like protein | EP402R | Outer envelope | 149 NTPIYYMRPSTQPLN |
| Cd2-like protein | EP402R | Outer envelope | 150 TPIYYMRPSTQPLNP |
| Cd2-like protein | EP402R | Outer envelope | 151 PIYYMRPSTQPLNPF |
| Cd2-like protein | EP402R | Outer envelope | 152 IYYMRPSTQPLNPFP |
| Cd2-like protein | EP402R | Outer envelope | 153 YYMRPSTQPLNPFPL |
| Cd2-like protein | EP402R | Outer envelope | 154 YMRPSTQPLNPFPLP |
| Cd2-like protein | EP402R | Outer envelope | 155 MRPSTQPLNPFPLPK |
| Cd2-like protein | EP402R | Outer envelope | 156 RPSTQPLNPFPLPKP |
| Cd2-like protein | EP402R | Outer envelope | 157 PSTQPLNPFPLPKPC |
| Cd2-like protein | EP402R | Outer envelope | 158 STQPLNPFPLPKPCP |
| Cd2-like protein | EP402R | Outer envelope | 159 TQPLNPFPLPKPCPP |
| Cd2-like protein | EP402R | Outer envelope | 160 QPLNPFPLPKPCPPP |
| Cd2-like protein | EP402R | Outer envelope | 161 PLNPFPLPKPCPPPK |
| Cd2-like protein | EP402R | Outer envelope | 162 LNPFPLPKPCPPPKP |
| Cd2-like protein | EP402R | Outer envelope | 163 NPFPLPKPCPPPKPC |
| Cd2-like protein | EP402R | Outer envelope | 164 PFPLPKPCPPPKPCP |
| Cd2-like protein | EP402R | Outer envelope | 165 FPLPKPCPPPKPCPP |
| Cd2-like protein | EP402R | Outer envelope | 166 PLPKPCPPPKPCPPP |
| Cd2-like protein | EP402R | Outer envelope | 167 LPKPCPPPKPCPPPK |
| Cd2-like protein | EP402R | Outer envelope | 168 PKPCPPPKPCPPPKP |
| Cd2-like protein | EP402R | Outer envelope | 169 KPCPPPKPCPPPKPC |
| Cd2-like protein | EP402R | Outer envelope | 170 PCPPPKPCPPPKPCP |
| Cd2-like protein | EP402R | Outer envelope | 171 CPPPKPCPPPKPCPP |
| Cd2-like protein | EP402R | Outer envelope | 172 PPPKPCPPPKPCPPP |
| Cd2-like protein | EP402R | Outer envelope | 173 PPKPCPPPKPCPPPK |
| Cd2-like protein | EP402R | Outer envelope | 174 PKPCPPPKPCPPPKP |
| Cd2-like protein | EP402R | Outer envelope | 175 KPCPPPKPCPPPKPC |
| Cd2-like protein | EP402R | Outer envelope | 176 PCPPPKPCPPPKPCP |
| Cd2-like protein | EP402R | Outer envelope | 177 CPPPKPCPPPKPCPP |
| Cd2-like protein | EP402R | Outer envelope | 178 PPPKPCPPPKPCPPP |
| Cd2-like protein | EP402R | Outer envelope | 179 PPKPCPPPKPCPPPK |
| Cd2-like protein | EP402R | Outer envelope | 180 PKPCPPPKPCPPPKP |
| Cd2-like protein | EP402R | Outer envelope | 181 KPCPPPKPCPPPKPC |
| Cd2-like protein | EP402R | Outer envelope | 182 PCPPPKPCPPPKPCS |
| Cd2-like protein | EP402R | Outer envelope | 183 CPPPKPCPPPKPCSP |
| Cd2-like protein | EP402R | Outer envelope | 184 PPPKPCPPPKPCSPP |
| Cd2-like protein | EP402R | Outer envelope | 185 PPKPCPPPKPCSPPK |
| Cd2-like protein | EP402R | Outer envelope | 186 PKPCPPPKPCSPPKP |
| Cd2-like protein | EP402R | Outer envelope | 187 KPCPPPKPCSPPKPC |
| Cd2-like protein | EP402R | Outer envelope | 188 PCPPPKPCSPPKPCR |
| Cd2-like protein | EP402R | Outer envelope | 189 CPPPKPCSPPKPCRP |
| Cd2-like protein | EP402R | Outer envelope | 190 PPPKPCSPPKPCRPP |
| Cd2-like protein | EP402R | Outer envelope | 191 PPKPCSPPKPCRPPK |
| Cd2-like protein | EP402R | Outer envelope | 192 PKPCSPPKPCRPPKP |
| Cd2-like protein | EP402R | Outer envelope | 193 KPCSPPKPCRPPKPC |
| Cd2-like protein | EP402R | Outer envelope | 194 PCSPPKPCRPPKPCP |
| Cd2-like protein | EP402R | Outer envelope | 195 CSPPKPCRPPKPCPP |
| Cd2-like protein | EP402R | Outer envelope | 196 SPPKPCRPPKPCPPP |
| Cd2-like protein | EP402R | Outer envelope | 197 PPKPCRPPKPCPPPK |
| Cd2-like protein | EP402R | Outer envelope | 198 PKPCRPPKPCPPPKP |
| Cd2-like protein | EP402R | Outer envelope | 199 KPCRPPKPCPPPKPC |
| Cd2-like protein | EP402R | Outer envelope | 200 PCRPPKPCPPPKPCP |
| Cd2-like protein | EP402R | Outer envelope | 201 CRPPKPCPPPKPCPP |
| Cd2-like protein | EP402R | Outer envelope | 202 RPPKPCPPPKPCPPP |
| Cd2-like protein | EP402R | Outer envelope | 203 PPKPCPPPKPCPPPK |
| Cd2-like protein | EP402R | Outer envelope | 204 PKPCPPPKPCPPPKP |

| | | | | |
|---|---|---|---|---|
| Cd2-like protein | EP402R | Outer envelope | 205 | KPCPPPKPCPPPKPC |
| Cd2-like protein | EP402R | Outer envelope | 206 | PCPPPKPCPPPKPCP |
| Cd2-like protein | EP402R | Outer envelope | 207 | CPPPKPCPPPKPCPP |
| Cd2-like protein | EP402R | Outer envelope | 208 | PPPKPCPPPKPCPPP |
| Cd2-like protein | EP402R | Outer envelope | 209 | PPKPCPPPKPCPPPK |
| Cd2-like protein | EP402R | Outer envelope | 210 | PKPCPPPKPCPPPKP |
| Cd2-like protein | EP402R | Outer envelope | 211 | KPCPPPKPCPPPKPC |
| Cd2-like protein | EP402R | Outer envelope | 212 | PCPPPKPCPPPKPCP |
| Cd2-like protein | EP402R | Outer envelope | 213 | CPPPKPCPPPKPCPP |
| Cd2-like protein | EP402R | Outer envelope | 214 | PPPKPCPPPKPCPPP |
| Cd2-like protein | EP402R | Outer envelope | 215 | PPKPCPPPKPCPPPK |
| Cd2-like protein | EP402R | Outer envelope | 216 | PKPCPPPKPCPPPKP |
| Cd2-like protein | EP402R | Outer envelope | 217 | KPCPPPKPCPPPKPC |
| Cd2-like protein | EP402R | Outer envelope | 218 | PCPPPKPCPPPKPCP |
| Cd2-like protein | EP402R | Outer envelope | 219 | CPPPKPCPPPKPCPS |
| Cd2-like protein | EP402R | Outer envelope | 220 | PPPKPCPPPKPCPSP |
| Cd2-like protein | EP402R | Outer envelope | 221 | PPKPCPPPKPCPSPE |
| Cd2-like protein | EP402R | Outer envelope | 222 | PKPCPPPKPCPSPES |
| Cd2-like protein | EP402R | Outer envelope | 223 | KPCPPPKPCPSPESY |
| Cd2-like protein | EP402R | Outer envelope | 224 | PCPPPKPCPSPESYS |
| Cd2-like protein | EP402R | Outer envelope | 225 | CPPPKPCPSPESYSP |
| Cd2-like protein | EP402R | Outer envelope | 226 | PPPKPCPSPESYSPP |
| Cd2-like protein | EP402R | Outer envelope | 227 | PPKPCPSPESYSPPK |
| Cd2-like protein | EP402R | Outer envelope | 228 | PKPCPSPESYSPPKP |
| Cd2-like protein | EP402R | Outer envelope | 229 | KPCPSPESYSPPKPL |
| Cd2-like protein | EP402R | Outer envelope | 230 | PCPSPESYSPPKPLP |
| Cd2-like protein | EP402R | Outer envelope | 231 | CPSPESYSPPKPLPS |
| Cd2-like protein | EP402R | Outer envelope | 232 | PSPESYSPPKPLPSI |
| Cd2-like protein | EP402R | Outer envelope | 233 | SPESYSPPKPLPSIP |
| Cd2-like protein | EP402R | Outer envelope | 234 | PESYSPPKPLPSIPG |
| Cd2-like protein | EP402R | Outer envelope | 235 | ESYSPPKPLPSIPGS |
| Cd2-like protein | EP402R | Outer envelope | 236 | SYSPPKPLPSIPGSG |
| Cd2-like protein | EP402R | Outer envelope | 237 | YSPPKPLPSIPGSGS |
| Cd2-like protein | EP402R | Outer envelope | 238 | SPPKPLPSIPGSGSG |
| Cd2-like protein | EP402R | Outer envelope | 239 | PPKPLPSIPGSGSGS |
| Cd2-like protein | EP402R | Outer envelope | 240 | PKPLPSIPGSGSGSG |
| B646L | p72 | Capsid | 1 | GSGSGSGMASGGAFC |
| B646L | p72 | Capsid | 2 | SGSGSGMASGGAFCL |
| B646L | p72 | Capsid | 3 | GSGSGMASGGAFCLI |
| B646L | p72 | Capsid | 4 | SGSGMASGGAFCLIA |
| B646L | p72 | Capsid | 5 | GSGMASGGAFCLIAN |
| B646L | p72 | Capsid | 6 | SGMASGGAFCLIAND |
| B646L | p72 | Capsid | 7 | GMASGGAFCLIANDG |
| B646L | p72 | Capsid | 8 | MASGGAFCLIANDGK |
| B646L | p72 | Capsid | 9 | ASGGAFCLIANDGKA |
| B646L | p72 | Capsid | 10 | SGGAFCLIANDGKAD |
| B646L | p72 | Capsid | 11 | GGAFCLIANDGKADK |
| B646L | p72 | Capsid | 12 | GAFCLIANDGKADKI |
| B646L | p72 | Capsid | 13 | AFCLIANDGKADKII |
| B646L | p72 | Capsid | 14 | FCLIANDGKADKIIL |
| B646L | p72 | Capsid | 15 | CLIANDGKADKIILA |
| B646L | p72 | Capsid | 16 | LIANDGKADKIILAQ |
| B646L | p72 | Capsid | 17 | IANDGKADKIILAQD |
| B646L | p72 | Capsid | 18 | ANDGKADKIILAQDL |
| B646L | p72 | Capsid | 19 | NDGKADKIILAQDLL |
| B646L | p72 | Capsid | 20 | DGKADKIILAQDLLN |
| B646L | p72 | Capsid | 21 | GKADKIILAQDLLNS |
| B646L | p72 | Capsid | 22 | KADKIILAQDLLNSR |
| B646L | p72 | Capsid | 23 | ADKIILAQDLLNSRI |
| B646L | p72 | Capsid | 24 | DKIILAQDLLNSRIS |
| B646L | p72 | Capsid | 25 | KIILAQDLLNSRISN |
| B646L | p72 | Capsid | 26 | IILAQDLLNSRISNI |
| B646L | p72 | Capsid | 27 | ILAQDLLNSRISNIK |
| B646L | p72 | Capsid | 28 | LAQDLLNSRISNIKN |
| B646L | p72 | Capsid | 29 | AQDLLNSRISNIKNV |
| B646L | p72 | Capsid | 30 | QDLLNSRISNIKNVN |
| B646L | p72 | Capsid | 31 | DLLNSRISNIKNVNK |

| | | | |
|---|---|---|---|
| B646L | p72 | Capsid | 32 LLNSRISNIKNVNKS |
| B646L | p72 | Capsid | 33 LNSRISNIKNVNKSY |
| B646L | p72 | Capsid | 34 NSRISNIKNVNKSYG |
| B646L | p72 | Capsid | 35 SRISNIKNVNKSYGK |
| B646L | p72 | Capsid | 36 RISNIKNVNKSYGKP |
| B646L | p72 | Capsid | 37 ISNIKNVNKSYGKPD |
| B646L | p72 | Capsid | 38 SNIKNVNKSYGKPDP |
| B646L | p72 | Capsid | 39 NIKNVNKSYGKPDPE |
| B646L | p72 | Capsid | 40 IKNVNKSYGKPDPEP |
| B646L | p72 | Capsid | 41 KNVNKSYGKPDPEPT |
| B646L | p72 | Capsid | 42 NVNKSYGKPDPEPTL |
| B646L | p72 | Capsid | 43 VNKSYGKPDPEPTLS |
| B646L | p72 | Capsid | 44 NKSYGKPDPEPTLSQ |
| B646L | p72 | Capsid | 45 KSYGKPDPEPTLSQI |
| B646L | p72 | Capsid | 46 SYGKPDPEPTLSQIE |
| B646L | p72 | Capsid | 47 YGKPDPEPTLSQIEE |
| B646L | p72 | Capsid | 48 GKPDPEPTLSQIEET |
| B646L | p72 | Capsid | 49 KPDPEPTLSQIEETH |
| B646L | p72 | Capsid | 50 PDPEPTLSQIEETHL |
| B646L | p72 | Capsid | 51 DPEPTLSQIEETHLV |
| B646L | p72 | Capsid | 52 PEPTLSQIEETHLVH |
| B646L | p72 | Capsid | 53 EPTLSQIEETHLVHF |
| B646L | p72 | Capsid | 54 PTLSQIEETHLVHFN |
| B646L | p72 | Capsid | 55 TLSQIEETHLVHFNA |
| B646L | p72 | Capsid | 56 LSQIEETHLVHFNAH |
| B646L | p72 | Capsid | 57 SQIEETHLVHFNAHF |
| B646L | p72 | Capsid | 58 QIEETHLVHFNAHFK |
| B646L | p72 | Capsid | 59 IEETHLVHFNAHFKP |
| B646L | p72 | Capsid | 60 EETHLVHFNAHFKPY |
| B646L | p72 | Capsid | 61 ETHLVHFNAHFKPYV |
| B646L | p72 | Capsid | 62 THLVHFNAHFKPYVP |
| B646L | p72 | Capsid | 63 HLVHFNAHFKPYVPV |
| B646L | p72 | Capsid | 64 LVHFNAHFKPYVPVG |
| B646L | p72 | Capsid | 65 VHFNAHFKPYVPVGF |
| B646L | p72 | Capsid | 66 HFNAHFKPYVPVGFE |
| B646L | p72 | Capsid | 67 FNAHFKPYVPVGFEY |
| B646L | p72 | Capsid | 68 NAHFKPYVPVGFEYN |
| B646L | p72 | Capsid | 69 AHFKPYVPVGFEYNK |
| B646L | p72 | Capsid | 70 HFKPYVPVGFEYNKV |
| B646L | p72 | Capsid | 71 FKPYVPVGFEYNKVR |
| B646L | p72 | Capsid | 72 KPYVPVGFEYNKVRP |
| B646L | p72 | Capsid | 73 PYVPVGFEYNKVRPH |
| B646L | p72 | Capsid | 74 YVPVGFEYNKVRPHT |
| B646L | p72 | Capsid | 75 VPVGFEYNKVRPHTG |
| B646L | p72 | Capsid | 76 PVGFEYNKVRPHTGT |
| B646L | p72 | Capsid | 77 VGFEYNKVRPHTGTP |
| B646L | p72 | Capsid | 78 GFEYNKVRPHTGTPT |
| B646L | p72 | Capsid | 79 FEYNKVRPHTGTPTL |
| B646L | p72 | Capsid | 80 EYNKVRPHTGTPTLG |
| B646L | p72 | Capsid | 81 YNKVRPHTGTPTLGN |
| B646L | p72 | Capsid | 82 NKVRPHTGTPTLGNK |
| B646L | p72 | Capsid | 83 KVRPHTGTPTLGNKL |
| B646L | p72 | Capsid | 84 VRPHTGTPTLGNKLT |
| B646L | p72 | Capsid | 85 RPHTGTPTLGNKLTF |
| B646L | p72 | Capsid | 86 PHTGTPTLGNKLTFG |
| B646L | p72 | Capsid | 87 HTGTPTLGNKLTFGI |
| B646L | p72 | Capsid | 88 TGTPTLGNKLTFGIP |
| B646L | p72 | Capsid | 89 GTPTLGNKLTFGIPQ |
| B646L | p72 | Capsid | 90 TPTLGNKLTFGIPQY |
| B646L | p72 | Capsid | 91 PTLGNKLTFGIPQYG |
| B646L | p72 | Capsid | 92 TLGNKLTFGIPQYGD |
| B646L | p72 | Capsid | 93 LGNKLTFGIPQYGDF |
| B646L | p72 | Capsid | 94 GNKLTFGIPQYGDFF |
| B646L | p72 | Capsid | 95 NKLTFGIPQYGDFFH |
| B646L | p72 | Capsid | 96 KLTFGIPQYGDFFHD |
| B646L | p72 | Capsid | 97 LTFGIPQYGDFFHDM |
| B646L | p72 | Capsid | 98 TFGIPQYGDFFHDMV |

| | | | | |
|---|---|---|---|---|
| B646L | p72 | Capsid | 99 | FGIPQYGDFFHDMVG |
| B646L | p72 | Capsid | 100 | GIPQYGDFFHDMVGH |
| B646L | p72 | Capsid | 101 | IPQYGDFFHDMVGHH |
| B646L | p72 | Capsid | 102 | PQYGDFFHDMVGHHI |
| B646L | p72 | Capsid | 103 | QYGDFFHDMVGHHIL |
| B646L | p72 | Capsid | 104 | YGDFFHDMVGHHILG |
| B646L | p72 | Capsid | 105 | GDFFHDMVGHHILGA |
| B646L | p72 | Capsid | 106 | DFFHDMVGHHILGAC |
| B646L | p72 | Capsid | 107 | FFHDMVGHHILGACH |
| B646L | p72 | Capsid | 108 | FHDMVGHHILGACHS |
| B646L | p72 | Capsid | 109 | HDMVGHHILGACHSS |
| B646L | p72 | Capsid | 110 | DMVGHHILGACHSSW |
| B646L | p72 | Capsid | 111 | MVGHHILGACHSSWQ |
| B646L | p72 | Capsid | 112 | VGHHILGACHSSWQD |
| B646L | p72 | Capsid | 113 | GHHILGACHSSWQDA |
| B646L | p72 | Capsid | 114 | HHILGACHSSWQDAP |
| B646L | p72 | Capsid | 115 | HILGACHSSWQDAPI |
| B646L | p72 | Capsid | 116 | ILGACHSSWQDAPIQ |
| B646L | p72 | Capsid | 117 | LGACHSSWQDAPIQG |
| B646L | p72 | Capsid | 118 | GACHSSWQDAPIQGT |
| B646L | p72 | Capsid | 119 | ACHSSWQDAPIQGTS |
| B646L | p72 | Capsid | 120 | CHSSWQDAPIQGTSQ |
| B646L | p72 | Capsid | 121 | HSSWQDAPIQGTSQM |
| B646L | p72 | Capsid | 122 | SSWQDAPIQGTSQMG |
| B646L | p72 | Capsid | 123 | SWQDAPIQGTSQMGA |
| B646L | p72 | Capsid | 124 | WQDAPIQGTSQMGAH |
| B646L | p72 | Capsid | 125 | QDAPIQGTSQMGAHG |
| B646L | p72 | Capsid | 126 | DAPIQGTSQMGAHGQ |
| B646L | p72 | Capsid | 127 | APIQGTSQMGAHGQL |
| B646L | p72 | Capsid | 128 | PIQGTSQMGAHGQLQ |
| B646L | p72 | Capsid | 129 | IQGTSQMGAHGQLQT |
| B646L | p72 | Capsid | 130 | QGTSQMGAHGQLQTF |
| B646L | p72 | Capsid | 131 | GTSQMGAHGQLQTFP |
| B646L | p72 | Capsid | 132 | TSQMGAHGQLQTFPR |
| B646L | p72 | Capsid | 133 | SQMGAHGQLQTFPRN |
| B646L | p72 | Capsid | 134 | QMGAHGQLQTFPRNG |
| B646L | p72 | Capsid | 135 | MGAHGQLQTFPRNGY |
| B646L | p72 | Capsid | 136 | GAHGQLQTFPRNGYD |
| B646L | p72 | Capsid | 137 | AHGQLQTFPRNGYDW |
| B646L | p72 | Capsid | 138 | HGQLQTFPRNGYDWD |
| B646L | p72 | Capsid | 139 | GQLQTFPRNGYDWDN |
| B646L | p72 | Capsid | 140 | QLQTFPRNGYDWDNQ |
| B646L | p72 | Capsid | 141 | LQTFPRNGYDWDNQT |
| B646L | p72 | Capsid | 142 | QTFPRNGYDWDNQTP |
| B646L | p72 | Capsid | 143 | TFPRNGYDWDNQTPL |
| B646L | p72 | Capsid | 144 | FPRNGYDWDNQTPLE |
| B646L | p72 | Capsid | 145 | PRNGYDWDNQTPLEG |
| B646L | p72 | Capsid | 146 | RNGYDWDNQTPLEGA |
| B646L | p72 | Capsid | 147 | NGYDWDNQTPLEGAV |
| B646L | p72 | Capsid | 148 | GYDWDNQTPLEGAVY |
| B646L | p72 | Capsid | 149 | YDWDNQTPLEGAVYT |
| B646L | p72 | Capsid | 150 | DWDNQTPLEGAVYTL |
| B646L | p72 | Capsid | 151 | WDNQTPLEGAVYTLV |
| B646L | p72 | Capsid | 152 | DNQTPLEGAVYTLVD |
| B646L | p72 | Capsid | 153 | NQTPLEGAVYTLVDP |
| B646L | p72 | Capsid | 154 | QTPLEGAVYTLVDPF |
| B646L | p72 | Capsid | 155 | TPLEGAVYTLVDPFG |
| B646L | p72 | Capsid | 156 | PLEGAVYTLVDPFGR |
| B646L | p72 | Capsid | 157 | LEGAVYTLVDPFGRP |
| B646L | p72 | Capsid | 158 | EGAVYTLVDPFGRPI |
| B646L | p72 | Capsid | 159 | GAVYTLVDPFGRPIV |
| B646L | p72 | Capsid | 160 | AVYTLVDPFGRPIVP |
| B646L | p72 | Capsid | 161 | VYTLVDPFGRPIVPG |
| B646L | p72 | Capsid | 162 | YTLVDPFGRPIVPGT |
| B646L | p72 | Capsid | 163 | TLVDPFGRPIVPGTK |
| B646L | p72 | Capsid | 164 | LVDPFGRPIVPGTKN |
| B646L | p72 | Capsid | 165 | VDPFGRPIVPGTKNA |

| | | | |
|---|---|---|---|
| B646L | p72 | Capsid | 166 DPFGRPIVPGTKNAY |
| B646L | p72 | Capsid | 167 PFGRPIVPGTKNAYR |
| B646L | p72 | Capsid | 168 FGRPIVPGTKNAYRN |
| B646L | p72 | Capsid | 169 GRPIVPGTKNAYRNL |
| B646L | p72 | Capsid | 170 RPIVPGTKNAYRNLV |
| B646L | p72 | Capsid | 171 PIVPGTKNAYRNLVY |
| B646L | p72 | Capsid | 172 IVPGTKNAYRNLVYY |
| B646L | p72 | Capsid | 173 VPGTKNAYRNLVYYC |
| B646L | p72 | Capsid | 174 PGTKNAYRNLVYYCE |
| B646L | p72 | Capsid | 175 GTKNAYRNLVYYCEY |
| B646L | p72 | Capsid | 176 TKNAYRNLVYYCEYP |
| B646L | p72 | Capsid | 177 KNAYRNLVYYCEYPG |
| B646L | p72 | Capsid | 178 NAYRNLVYYCEYPGE |
| B646L | p72 | Capsid | 179 AYRNLVYYCEYPGER |
| B646L | p72 | Capsid | 180 YRNLVYYCEYPGERL |
| B646L | p72 | Capsid | 181 RNLVYYCEYPGERLY |
| B646L | p72 | Capsid | 182 NLVYYCEYPGERLYE |
| B646L | p72 | Capsid | 183 LVYYCEYPGERLYEN |
| B646L | p72 | Capsid | 184 VYYCEYPGERLYENV |
| B646L | p72 | Capsid | 185 YYCEYPGERLYENVR |
| B646L | p72 | Capsid | 186 YCEYPGERLYENVRF |
| B646L | p72 | Capsid | 187 CEYPGERLYENVRFD |
| B646L | p72 | Capsid | 188 EYPGERLYENVRFDV |
| B646L | p72 | Capsid | 189 YPGERLYENVRFDVN |
| B646L | p72 | Capsid | 190 PGERLYENVRFDVNG |
| B646L | p72 | Capsid | 191 GERLYENVRFDVNGN |
| B646L | p72 | Capsid | 192 ERLYENVRFDVNGNS |
| B646L | p72 | Capsid | 193 RLYENVRFDVNGNSL |
| B646L | p72 | Capsid | 194 LYENVRFDVNGNSLD |
| B646L | p72 | Capsid | 195 YENVRFDVNGNSLDE |
| B646L | p72 | Capsid | 196 ENVRFDVNGNSLDEY |
| B646L | p72 | Capsid | 197 NVRFDVNGNSLDEYS |
| B646L | p72 | Capsid | 198 VRFDVNGNSLDEYSS |
| B646L | p72 | Capsid | 199 RFDVNGNSLDEYSSD |
| B646L | p72 | Capsid | 200 FDVNGNSLDEYSSDV |
| B646L | p72 | Capsid | 201 DVNGNSLDEYSSDVT |
| B646L | p72 | Capsid | 202 VNGNSLDEYSSDVTT |
| B646L | p72 | Capsid | 203 NGNSLDEYSSDVTTL |
| B646L | p72 | Capsid | 204 GNSLDEYSSDVTTLV |
| B646L | p72 | Capsid | 205 NSLDEYSSDVTTLVR |
| B646L | p72 | Capsid | 206 SLDEYSSDVTTLVRK |
| B646L | p72 | Capsid | 207 LDEYSSDVTTLVRKF |
| B646L | p72 | Capsid | 208 DEYSSDVTTLVRKFC |
| B646L | p72 | Capsid | 209 EYSSDVTTLVRKFCI |
| B646L | p72 | Capsid | 210 YSSDVTTLVRKFCIP |
| B646L | p72 | Capsid | 211 SSDVTTLVRKFCIPG |
| B646L | p72 | Capsid | 212 SDVTTLVRKFCIPGD |
| B646L | p72 | Capsid | 213 DVTTLVRKFCIPGDK |
| B646L | p72 | Capsid | 214 VTTLVRKFCIPGDKM |
| B646L | p72 | Capsid | 215 TTLVRKFCIPGDKMT |
| B646L | p72 | Capsid | 216 TLVRKFCIPGDKMTG |
| B646L | p72 | Capsid | 217 LVRKFCIPGDKMTGY |
| B646L | p72 | Capsid | 218 VRKFCIPGDKMTGYK |
| B646L | p72 | Capsid | 219 RKFCIPGDKMTGYKH |
| B646L | p72 | Capsid | 220 KFCIPGDKMTGYKHL |
| B646L | p72 | Capsid | 221 FCIPGDKMTGYKHLV |
| B646L | p72 | Capsid | 222 CIPGDKMTGYKHLVG |
| B646L | p72 | Capsid | 223 IPGDKMTGYKHLVGQ |
| B646L | p72 | Capsid | 224 PGDKMTGYKHLVGQE |
| B646L | p72 | Capsid | 225 GDKMTGYKHLVGQEV |
| B646L | p72 | Capsid | 226 DKMTGYKHLVGQEVS |
| B646L | p72 | Capsid | 227 KMTGYKHLVGQEVSV |
| B646L | p72 | Capsid | 228 MTGYKHLVGQEVSVE |
| B646L | p72 | Capsid | 229 TGYKHLVGQEVSVEG |
| B646L | p72 | Capsid | 230 GYKHLVGQEVSVEGT |
| B646L | p72 | Capsid | 231 YKHLVGQEVSVEGTS |
| B646L | p72 | Capsid | 232 KHLVGQEVSVEGTSG |

| | | | |
|---|---|---|---|
| B646L | p72 | Capsid | 233 HLVGQEVSVEGTSGP |
| B646L | p72 | Capsid | 234 LVGQEVSVEGTSGPL |
| B646L | p72 | Capsid | 235 VGQEVSVEGTSGPLL |
| B646L | p72 | Capsid | 236 GQEVSVEGTSGPLLC |
| B646L | p72 | Capsid | 237 QEVSVEGTSGPLLCN |
| B646L | p72 | Capsid | 238 EVSVEGTSGPLLCNI |
| B646L | p72 | Capsid | 239 VSVEGTSGPLLCNIH |
| B646L | p72 | Capsid | 240 SVEGTSGPLLCNIHD |
| B646L | p72 | Capsid | 241 VEGTSGPLLCNIHDL |
| B646L | p72 | Capsid | 242 EGTSGPLLCNIHDLH |
| B646L | p72 | Capsid | 243 GTSGPLLCNIHDLHK |
| B646L | p72 | Capsid | 244 TSGPLLCNIHDLHKP |
| B646L | p72 | Capsid | 245 SGPLLCNIHDLHKPH |
| B646L | p72 | Capsid | 246 GPLLCNIHDLHKPHQ |
| B646L | p72 | Capsid | 247 PLLCNIHDLHKPHQS |
| B646L | p72 | Capsid | 248 LLCNIHDLHKPHQSK |
| B646L | p72 | Capsid | 249 LCNIHDLHKPHQSKP |
| B646L | p72 | Capsid | 250 CNIHDLHKPHQSKPI |
| B646L | p72 | Capsid | 251 NIHDLHKPHQSKPIL |
| B646L | p72 | Capsid | 252 IHDLHKPHQSKPILT |
| B646L | p72 | Capsid | 253 HDLHKPHQSKPILTD |
| B646L | p72 | Capsid | 254 DLHKPHQSKPILTDE |
| B646L | p72 | Capsid | 255 LHKPHQSKPILTDEN |
| B646L | p72 | Capsid | 256 HKPHQSKPILTDEND |
| B646L | p72 | Capsid | 257 KPHQSKPILTDENDT |
| B646L | p72 | Capsid | 258 PHQSKPILTDENDTQ |
| B646L | p72 | Capsid | 259 HQSKPILTDENDTQR |
| B646L | p72 | Capsid | 260 QSKPILTDENDTQRT |
| B646L | p72 | Capsid | 261 SKPILTDENDTQRTC |
| B646L | p72 | Capsid | 262 KPILTDENDTQRTCS |
| B646L | p72 | Capsid | 263 PILTDENDTQRTCSH |
| B646L | p72 | Capsid | 264 ILTDENDTQRTCSHT |
| B646L | p72 | Capsid | 265 LTDENDTQRTCSHTN |
| B646L | p72 | Capsid | 266 TDENDTQRTCSHTNP |
| B646L | p72 | Capsid | 267 DENDTQRTCSHTNPK |
| B646L | p72 | Capsid | 268 ENDTQRTCSHTNPKF |
| B646L | p72 | Capsid | 269 NDTQRTCSHTNPKFL |
| B646L | p72 | Capsid | 270 DTQRTCSHTNPKFLS |
| B646L | p72 | Capsid | 271 TQRTCSHTNPKFLSQ |
| B646L | p72 | Capsid | 272 QRTCSHTNPKFLSQH |
| B646L | p72 | Capsid | 273 RTCSHTNPKFLSQHF |
| B646L | p72 | Capsid | 274 TCSHTNPKFLSQHFP |
| B646L | p72 | Capsid | 275 CSHTNPKFLSQHFPE |
| B646L | p72 | Capsid | 276 SHTNPKFLSQHFPEN |
| B646L | p72 | Capsid | 277 HTNPKFLSQHFPENS |
| B646L | p72 | Capsid | 278 TNPKFLSQHFPENSH |
| B646L | p72 | Capsid | 279 NPKFLSQHFPENSHN |
| B646L | p72 | Capsid | 280 PKFLSQHFPENSHNI |
| B646L | p72 | Capsid | 281 KFLSQHFPENSHNIQ |
| B646L | p72 | Capsid | 282 FLSQHFPENSHNIQT |
| B646L | p72 | Capsid | 283 LSQHFPENSHNIQTA |
| B646L | p72 | Capsid | 284 SQHFPENSHNIQTAG |
| B646L | p72 | Capsid | 285 QHFPENSHNIQTAGK |
| B646L | p72 | Capsid | 286 HFPENSHNIQTAGKQ |
| B646L | p72 | Capsid | 287 FPENSHNIQTAGKQD |
| B646L | p72 | Capsid | 288 PENSHNIQTAGKQDI |
| B646L | p72 | Capsid | 289 ENSHNIQTAGKQDIT |
| B646L | p72 | Capsid | 290 NSHNIQTAGKQDITP |
| B646L | p72 | Capsid | 291 SHNIQTAGKQDITPI |
| B646L | p72 | Capsid | 292 HNIQTAGKQDITPIT |
| B646L | p72 | Capsid | 293 NIQTAGKQDITPITD |
| B646L | p72 | Capsid | 294 IQTAGKQDITPITDA |
| B646L | p72 | Capsid | 295 QTAGKQDITPITDAT |
| B646L | p72 | Capsid | 296 TAGKQDITPITDATY |
| B646L | p72 | Capsid | 297 AGKQDITPITDATYL |
| B646L | p72 | Capsid | 298 GKQDITPITDATYLD |
| B646L | p72 | Capsid | 299 KQDITPITDATYLDI |

| | | | | |
|---|---|---|---|---|
| B646L | p72 | Capsid | 300 | QDITPITDATYLDIR |
| B646L | p72 | Capsid | 301 | DITPITDATYLDIRR |
| B646L | p72 | Capsid | 302 | ITPITDATYLDIRRN |
| B646L | p72 | Capsid | 303 | TPITDATYLDIRRNV |
| B646L | p72 | Capsid | 304 | PITDATYLDIRRNVH |
| B646L | p72 | Capsid | 305 | ITDATYLDIRRNVHY |
| B646L | p72 | Capsid | 306 | TDATYLDIRRNVHYS |
| B646L | p72 | Capsid | 307 | DATYLDIRRNVHYSC |
| B646L | p72 | Capsid | 308 | ATYLDIRRNVHYSCN |
| B646L | p72 | Capsid | 309 | TYLDIRRNVHYSCNG |
| B646L | p72 | Capsid | 310 | YLDIRRNVHYSCNGP |
| B646L | p72 | Capsid | 311 | LDIRRNVHYSCNGPQ |
| B646L | p72 | Capsid | 312 | DIRRNVHYSCNGPQT |
| B646L | p72 | Capsid | 313 | IRRNVHYSCNGPQTP |
| B646L | p72 | Capsid | 314 | RRNVHYSCNGPQTPK |
| B646L | p72 | Capsid | 315 | RNVHYSCNGPQTPKY |
| B646L | p72 | Capsid | 316 | NVHYSCNGPQTPKYY |
| B646L | p72 | Capsid | 317 | VHYSCNGPQTPKYYQ |
| B646L | p72 | Capsid | 318 | HYSCNGPQTPKYYQP |
| B646L | p72 | Capsid | 319 | YSCNGPQTPKYYQPP |
| B646L | p72 | Capsid | 320 | SCNGPQTPKYYQPPL |
| B646L | p72 | Capsid | 321 | CNGPQTPKYYQPPLA |
| B646L | p72 | Capsid | 322 | NGPQTPKYYQPPLAL |
| B646L | p72 | Capsid | 323 | GPQTPKYYQPPLALW |
| B646L | p72 | Capsid | 324 | PQTPKYYQPPLALWI |
| B646L | p72 | Capsid | 325 | QTPKYYQPPLALWIK |
| B646L | p72 | Capsid | 326 | TPKYYQPPLALWIKL |
| B646L | p72 | Capsid | 327 | PKYYQPPLALWIKLR |
| B646L | p72 | Capsid | 328 | KYYQPPLALWIKLRF |
| B646L | p72 | Capsid | 329 | YYQPPLALWIKLRFW |
| B646L | p72 | Capsid | 330 | YQPPLALWIKLRFWF |
| B646L | p72 | Capsid | 331 | QPPLALWIKLRFWFN |
| B646L | p72 | Capsid | 332 | PPLALWIKLRFWFNE |
| B646L | p72 | Capsid | 333 | PLALWIKLRFWFNEN |
| B646L | p72 | Capsid | 334 | LALWIKLRFWFNENV |
| B646L | p72 | Capsid | 335 | ALWIKLRFWFNENVN |
| B646L | p72 | Capsid | 336 | LWIKLRFWFNENVNL |
| B646L | p72 | Capsid | 337 | WIKLRFWFNENVNLA |
| B646L | p72 | Capsid | 338 | IKLRFWFNENVNLAI |
| B646L | p72 | Capsid | 339 | KLRFWFNENVNLAIP |
| B646L | p72 | Capsid | 340 | LRFWFNENVNLAIPS |
| B646L | p72 | Capsid | 341 | RFWFNENVNLAIPSV |
| B646L | p72 | Capsid | 342 | FWFNENVNLAIPSVS |
| B646L | p72 | Capsid | 343 | WFNENVNLAIPSVSI |
| B646L | p72 | Capsid | 344 | FNENVNLAIPSVSIP |
| B646L | p72 | Capsid | 345 | NENVNLAIPSVSIPF |
| B646L | p72 | Capsid | 346 | ENVNLAIPSVSIPFG |
| B646L | p72 | Capsid | 347 | NVNLAIPSVSIPFGE |
| B646L | p72 | Capsid | 348 | VNLAIPSVSIPFGER |
| B646L | p72 | Capsid | 349 | NLAIPSVSIPFGERF |
| B646L | p72 | Capsid | 350 | LAIPSVSIPFGERFI |
| B646L | p72 | Capsid | 351 | AIPSVSIPFGERFIT |
| B646L | p72 | Capsid | 352 | IPSVSIPFGERFITI |
| B646L | p72 | Capsid | 353 | PSVSIPFGERFITIK |
| B646L | p72 | Capsid | 354 | SVSIPFGERFITIKL |
| B646L | p72 | Capsid | 355 | VSIPFGERFITIKLA |
| B646L | p72 | Capsid | 356 | SIPFGERFITIKLAS |
| B646L | p72 | Capsid | 357 | IPFGERFITIKLASQ |
| B646L | p72 | Capsid | 358 | PFGERFITIKLASQK |
| B646L | p72 | Capsid | 359 | FGERFITIKLASQKD |
| B646L | p72 | Capsid | 360 | GERFITIKLASQKDL |
| B646L | p72 | Capsid | 361 | ERFITIKLASQKDLV |
| B646L | p72 | Capsid | 362 | RFITIKLASQKDLVN |
| B646L | p72 | Capsid | 363 | FITIKLASQKDLVNE |
| B646L | p72 | Capsid | 364 | ITIKLASQKDLVNEF |
| B646L | p72 | Capsid | 365 | TIKLASQKDLVNEFP |
| B646L | p72 | Capsid | 366 | IKLASQKDLVNEFPG |

| | | | | |
|---|---|---|---|---|
| B646L | p72 | Capsid | 367 | KLASQKDLVNEFPGL |
| B646L | p72 | Capsid | 368 | LASQKDLVNEFPGLF |
| B646L | p72 | Capsid | 369 | ASQKDLVNEFPGLFV |
| B646L | p72 | Capsid | 370 | SQKDLVNEFPGLFVR |
| B646L | p72 | Capsid | 371 | QKDLVNEFPGLFVRQ |
| B646L | p72 | Capsid | 372 | KDLVNEFPGLFVRQS |
| B646L | p72 | Capsid | 373 | DLVNEFPGLFVRQSR |
| B646L | p72 | Capsid | 374 | LVNEFPGLFVRQSRF |
| B646L | p72 | Capsid | 375 | VNEFPGLFVRQSRFI |
| B646L | p72 | Capsid | 376 | NEFPGLFVRQSRFIA |
| B646L | p72 | Capsid | 377 | EFPGLFVRQSRFIAG |
| B646L | p72 | Capsid | 378 | FPGLFVRQSRFIAGR |
| B646L | p72 | Capsid | 379 | PGLFVRQSRFIAGRP |
| B646L | p72 | Capsid | 380 | GLFVRQSRFIAGRPS |
| B646L | p72 | Capsid | 381 | LFVRQSRFIAGRPSR |
| B646L | p72 | Capsid | 382 | FVRQSRFIAGRPSRR |
| B646L | p72 | Capsid | 383 | VRQSRFIAGRPSRRN |
| B646L | p72 | Capsid | 384 | RQSRFIAGRPSRRNI |
| B646L | p72 | Capsid | 385 | QSRFIAGRPSRRNIR |
| B646L | p72 | Capsid | 386 | SRFIAGRPSRRNIRF |
| B646L | p72 | Capsid | 387 | RFIAGRPSRRNIRFK |
| B646L | p72 | Capsid | 388 | FIAGRPSRRNIRFKP |
| B646L | p72 | Capsid | 389 | IAGRPSRRNIRFKPW |
| B646L | p72 | Capsid | 390 | AGRPSRRNIRFKPWF |
| B646L | p72 | Capsid | 391 | GRPSRRNIRFKPWFI |
| B646L | p72 | Capsid | 392 | RPSRRNIRFKPWFIP |
| B646L | p72 | Capsid | 393 | PSRRNIRFKPWFIPG |
| B646L | p72 | Capsid | 394 | SRRNIRFKPWFIPGV |
| B646L | p72 | Capsid | 395 | RRNIRFKPWFIPGVI |
| B646L | p72 | Capsid | 396 | RNIRFKPWFIPGVIN |
| B646L | p72 | Capsid | 397 | NIRFKPWFIPGVINE |
| B646L | p72 | Capsid | 398 | IRFKPWFIPGVINEI |
| B646L | p72 | Capsid | 399 | RFKPWFIPGVINEIS |
| B646L | p72 | Capsid | 400 | FKPWFIPGVINEISL |
| B646L | p72 | Capsid | 401 | KPWFIPGVINEISLT |
| B646L | p72 | Capsid | 402 | PWFIPGVINEISLTN |
| B646L | p72 | Capsid | 403 | WFIPGVINEISLTNN |
| B646L | p72 | Capsid | 404 | FIPGVINEISLTNNE |
| B646L | p72 | Capsid | 405 | IPGVINEISLTNNEL |
| B646L | p72 | Capsid | 406 | PGVINEISLTNNELY |
| B646L | p72 | Capsid | 407 | GVINEISLTNNELYI |
| B646L | p72 | Capsid | 408 | VINEISLTNNELYIN |
| B646L | p72 | Capsid | 409 | INEISLTNNELYINN |
| B646L | p72 | Capsid | 410 | NEISLTNNELYINNL |
| B646L | p72 | Capsid | 411 | EISLTNNELYINNLF |
| B646L | p72 | Capsid | 412 | ISLTNNELYINNLFV |
| B646L | p72 | Capsid | 413 | SLTNNELYINNLFVT |
| B646L | p72 | Capsid | 414 | LTNNELYINNLFVTP |
| B646L | p72 | Capsid | 415 | TNNELYINNLFVTPE |
| B646L | p72 | Capsid | 416 | NNELYINNLFVTPEI |
| B646L | p72 | Capsid | 417 | NELYINNLFVTPEIH |
| B646L | p72 | Capsid | 418 | ELYINNLFVTPEIHN |
| B646L | p72 | Capsid | 419 | LYINNLFVTPEIHNL |
| B646L | p72 | Capsid | 420 | YINNLFVTPEIHNLF |
| B646L | p72 | Capsid | 421 | INNLFVTPEIHNLFV |
| B646L | p72 | Capsid | 422 | NNLFVTPEIHNLFVK |
| B646L | p72 | Capsid | 423 | NLFVTPEIHNLFVKR |
| B646L | p72 | Capsid | 424 | LFVTPEIHNLFVKRV |
| B646L | p72 | Capsid | 425 | FVTPEIHNLFVKRVR |
| B646L | p72 | Capsid | 426 | VTPEIHNLFVKRVRF |
| B646L | p72 | Capsid | 427 | TPEIHNLFVKRVRFS |
| B646L | p72 | Capsid | 428 | PEIHNLFVKRVRFSL |
| B646L | p72 | Capsid | 429 | EIHNLFVKRVRFSLI |
| B646L | p72 | Capsid | 430 | IHNLFVKRVRFSLIR |
| B646L | p72 | Capsid | 431 | HNLFVKRVRFSLIRV |
| B646L | p72 | Capsid | 432 | NLFVKRVRFSLIRVH |
| B646L | p72 | Capsid | 433 | LFVKRVRFSLIRVHK |

| | | | | |
|---|---|---|---|---|
| B646L | p72 | Capsid | 434 | FVKRVRFSLIRVHKT |
| B646L | p72 | Capsid | 435 | VKRVRFSLIRVHKTQ |
| B646L | p72 | Capsid | 436 | KRVRFSLIRVHKTQV |
| B646L | p72 | Capsid | 437 | RVRFSLIRVHKTQVT |
| B646L | p72 | Capsid | 438 | VRFSLIRVHKTQVTH |
| B646L | p72 | Capsid | 439 | RFSLIRVHKTQVTHT |
| B646L | p72 | Capsid | 440 | FSLIRVHKTQVTHTN |
| B646L | p72 | Capsid | 441 | SLIRVHKTQVTHTNN |
| B646L | p72 | Capsid | 442 | LIRVHKTQVTHTNNN |
| B646L | p72 | Capsid | 443 | IRVHKTQVTHTNNNH |
| B646L | p72 | Capsid | 444 | RVHKTQVTHTNNNHH |
| B646L | p72 | Capsid | 445 | VHKTQVTHTNNNHHD |
| B646L | p72 | Capsid | 446 | HKTQVTHTNNNHHDE |
| B646L | p72 | Capsid | 447 | KTQVTHTNNNHHDEK |
| B646L | p72 | Capsid | 448 | TQVTHTNNNHHDEKL |
| B646L | p72 | Capsid | 449 | QVTHTNNNHHDEKLM |
| B646L | p72 | Capsid | 450 | VTHTNNNHHDEKLMS |
| B646L | p72 | Capsid | 451 | THTNNNHHDEKLMSA |
| B646L | p72 | Capsid | 452 | HTNNNHHDEKLMSAL |
| B646L | p72 | Capsid | 453 | TNNNHHDEKLMSALK |
| B646L | p72 | Capsid | 454 | NNNHHDEKLMSALKW |
| B646L | p72 | Capsid | 455 | NNHHDEKLMSALKWP |
| B646L | p72 | Capsid | 456 | NHHDEKLMSALKWPI |
| B646L | p72 | Capsid | 457 | HHDEKLMSALKWPIE |
| B646L | p72 | Capsid | 458 | HDEKLMSALKWPIEY |
| B646L | p72 | Capsid | 459 | DEKLMSALKWPIEYM |
| B646L | p72 | Capsid | 460 | EKLMSALKWPIEYMF |
| B646L | p72 | Capsid | 461 | KLMSALKWPIEYMFI |
| B646L | p72 | Capsid | 462 | LMSALKWPIEYMFIG |
| B646L | p72 | Capsid | 463 | MSALKWPIEYMFIGL |
| B646L | p72 | Capsid | 464 | SALKWPIEYMFIGLK |
| B646L | p72 | Capsid | 465 | ALKWPIEYMFIGLKP |
| B646L | p72 | Capsid | 466 | LKWPIEYMFIGLKPT |
| B646L | p72 | Capsid | 467 | KWPIEYMFIGLKPTW |
| B646L | p72 | Capsid | 468 | WPIEYMFIGLKPTWN |
| B646L | p72 | Capsid | 469 | PIEYMFIGLKPTWNI |
| B646L | p72 | Capsid | 470 | IEYMFIGLKPTWNIS |
| B646L | p72 | Capsid | 471 | EYMFIGLKPTWNISD |
| B646L | p72 | Capsid | 472 | YMFIGLKPTWNISDQ |
| B646L | p72 | Capsid | 473 | MFIGLKPTWNISDQN |
| B646L | p72 | Capsid | 474 | FIGLKPTWNISDQNP |
| B646L | p72 | Capsid | 475 | IGLKPTWNISDQNPH |
| B646L | p72 | Capsid | 476 | GLKPTWNISDQNPHQ |
| B646L | p72 | Capsid | 477 | LKPTWNISDQNPHQH |
| B646L | p72 | Capsid | 478 | KPTWNISDQNPHQHR |
| B646L | p72 | Capsid | 479 | PTWNISDQNPHQHRD |
| B646L | p72 | Capsid | 480 | TWNISDQNPHQHRDW |
| B646L | p72 | Capsid | 481 | WNISDQNPHQHRDWH |
| B646L | p72 | Capsid | 482 | NISDQNPHQHRDWHK |
| B646L | p72 | Capsid | 483 | ISDQNPHQHRDWHKF |
| B646L | p72 | Capsid | 484 | SDQNPHQHRDWHKFG |
| B646L | p72 | Capsid | 485 | DQNPHQHRDWHKFGH |
| B646L | p72 | Capsid | 486 | QNPHQHRDWHKFGHV |
| B646L | p72 | Capsid | 487 | NPHQHRDWHKFGHVV |
| B646L | p72 | Capsid | 488 | PHQHRDWHKFGHVVN |
| B646L | p72 | Capsid | 489 | HQHRDWHKFGHVVNA |
| B646L | p72 | Capsid | 490 | QHRDWHKFGHVVNAI |
| B646L | p72 | Capsid | 491 | HRDWHKFGHVVNAIM |
| B646L | p72 | Capsid | 492 | RDWHKFGHVVNAIMQ |
| B646L | p72 | Capsid | 493 | DWHKFGHVVNAIMQP |
| B646L | p72 | Capsid | 494 | WHKFGHVVNAIMQPT |
| B646L | p72 | Capsid | 495 | HKFGHVVNAIMQPTH |
| B646L | p72 | Capsid | 496 | KFGHVVNAIMQPTHH |
| B646L | p72 | Capsid | 497 | FGHVVNAIMQPTHHA |
| B646L | p72 | Capsid | 498 | GHVVNAIMQPTHHAE |
| B646L | p72 | Capsid | 499 | HVVNAIMQPTHHAEI |
| B646L | p72 | Capsid | 500 | VVNAIMQPTHHAEIS |

| | | | |
|---|---|---|---|
| B646L | p72 | Capsid | 501 VNAIMQPTHHAEISF |
| B646L | p72 | Capsid | 502 NAIMQPTHHAEISFQ |
| B646L | p72 | Capsid | 503 AIMQPTHHAEISFQD |
| B646L | p72 | Capsid | 504 IMQPTHHAEISFQDR |
| B646L | p72 | Capsid | 505 MQPTHHAEISFQDRD |
| B646L | p72 | Capsid | 506 QPTHHAEISFQDRDT |
| B646L | p72 | Capsid | 507 PTHHAEISFQDRDTA |
| B646L | p72 | Capsid | 508 THHAEISFQDRDTAL |
| B646L | p72 | Capsid | 509 HHAEISFQDRDTALP |
| B646L | p72 | Capsid | 510 HAEISFQDRDTALPD |
| B646L | p72 | Capsid | 511 AEISFQDRDTALPDA |
| B646L | p72 | Capsid | 512 EISFQDRDTALPDAC |
| B646L | p72 | Capsid | 513 ISFQDRDTALPDACS |
| B646L | p72 | Capsid | 514 SFQDRDTALPDACSS |
| B646L | p72 | Capsid | 515 FQDRDTALPDACSSI |
| B646L | p72 | Capsid | 516 QDRDTALPDACSSIS |
| B646L | p72 | Capsid | 517 DRDTALPDACSSISD |
| B646L | p72 | Capsid | 518 RDTALPDACSSISDI |
| B646L | p72 | Capsid | 519 DTALPDACSSISDIS |
| B646L | p72 | Capsid | 520 TALPDACSSISDISP |
| B646L | p72 | Capsid | 521 ALPDACSSISDISPV |
| B646L | p72 | Capsid | 522 LPDACSSISDISPVT |
| B646L | p72 | Capsid | 523 PDACSSISDISPVTY |
| B646L | p72 | Capsid | 524 DACSSISDISPVTYP |
| B646L | p72 | Capsid | 525 ACSSISDISPVTYPI |
| B646L | p72 | Capsid | 526 CSSISDISPVTYPIT |
| B646L | p72 | Capsid | 527 SSISDISPVTYPITL |
| B646L | p72 | Capsid | 528 SISDISPVTYPITLP |
| B646L | p72 | Capsid | 529 ISDISPVTYPITLPI |
| B646L | p72 | Capsid | 530 SDISPVTYPITLPII |
| B646L | p72 | Capsid | 531 DISPVTYPITLPIIK |
| B646L | p72 | Capsid | 532 ISPVTYPITLPIIKN |
| B646L | p72 | Capsid | 533 SPVTYPITLPIIKNI |
| B646L | p72 | Capsid | 534 PVTYPITLPIIKNIS |
| B646L | p72 | Capsid | 535 VTYPITLPIIKNISV |
| B646L | p72 | Capsid | 536 TYPITLPIIKNISVT |
| B646L | p72 | Capsid | 537 YPITLPIIKNISVTA |
| B646L | p72 | Capsid | 538 PITLPIIKNISVTAH |
| B646L | p72 | Capsid | 539 ITLPIIKNISVTAHG |
| B646L | p72 | Capsid | 540 TLPIIKNISVTAHGI |
| B646L | p72 | Capsid | 541 LPIIKNISVTAHGIN |
| B646L | p72 | Capsid | 542 PIIKNISVTAHGINL |
| B646L | p72 | Capsid | 543 IIKNISVTAHGINLI |
| B646L | p72 | Capsid | 544 IKNISVTAHGINLID |
| B646L | p72 | Capsid | 545 KNISVTAHGINLIDK |
| B646L | p72 | Capsid | 546 NISVTAHGINLIDKF |
| B646L | p72 | Capsid | 547 ISVTAHGINLIDKFP |
| B646L | p72 | Capsid | 548 SVTAHGINLIDKFPS |
| B646L | p72 | Capsid | 549 VTAHGINLIDKFPSK |
| B646L | p72 | Capsid | 550 TAHGINLIDKFPSKF |
| B646L | p72 | Capsid | 551 AHGINLIDKFPSKFC |
| B646L | p72 | Capsid | 552 HGINLIDKFPSKFCS |
| B646L | p72 | Capsid | 553 GINLIDKFPSKFCSS |
| B646L | p72 | Capsid | 554 INLIDKFPSKFCSSY |
| B646L | p72 | Capsid | 555 NLIDKFPSKFCSSYI |
| B646L | p72 | Capsid | 556 LIDKFPSKFCSSYIP |
| B646L | p72 | Capsid | 557 IDKFPSKFCSSYIPF |
| B646L | p72 | Capsid | 558 DKFPSKFCSSYIPFH |
| B646L | p72 | Capsid | 559 KFPSKFCSSYIPFHY |
| B646L | p72 | Capsid | 560 FPSKFCSSYIPFHYG |
| B646L | p72 | Capsid | 561 PSKFCSSYIPFHYGG |
| B646L | p72 | Capsid | 562 SKFCSSYIPFHYGGN |
| B646L | p72 | Capsid | 563 KFCSSYIPFHYGGNA |
| B646L | p72 | Capsid | 564 FCSSYIPFHYGGNAI |
| B646L | p72 | Capsid | 565 CSSYIPFHYGGNAIK |
| B646L | p72 | Capsid | 566 SSYIPFHYGGNAIKT |
| B646L | p72 | Capsid | 567 SYIPFHYGGNAIKTP |

| | | | |
|---|---|---|---|
| B646L | p72 | Capsid | 568 YIPFHYGGNAIKTPD |
| B646L | p72 | Capsid | 569 IPFHYGGNAIKTPDD |
| B646L | p72 | Capsid | 570 PFHYGGNAIKTPDDP |
| B646L | p72 | Capsid | 571 FHYGGNAIKTPDDPG |
| B646L | p72 | Capsid | 572 HYGGNAIKTPDDPGA |
| B646L | p72 | Capsid | 573 YGGNAIKTPDDPGAM |
| B646L | p72 | Capsid | 574 GGNAIKTPDDPGAMM |
| B646L | p72 | Capsid | 575 GNAIKTPDDPGAMMI |
| B646L | p72 | Capsid | 576 NAIKTPDDPGAMMIT |
| B646L | p72 | Capsid | 577 AIKTPDDPGAMMITF |
| B646L | p72 | Capsid | 578 IKTPDDPGAMMITFA |
| B646L | p72 | Capsid | 579 KTPDDPGAMMITFAL |
| B646L | p72 | Capsid | 580 TPDDPGAMMITFALK |
| B646L | p72 | Capsid | 581 PDDPGAMMITFALKP |
| B646L | p72 | Capsid | 582 DDPGAMMITFALKPR |
| B646L | p72 | Capsid | 583 DPGAMMITFALKPRE |
| B646L | p72 | Capsid | 584 PGAMMITFALKPREE |
| B646L | p72 | Capsid | 585 GAMMITFALKPREEY |
| B646L | p72 | Capsid | 586 AMMITFALKPREEYQ |
| B646L | p72 | Capsid | 587 MMITFALKPREEYQP |
| B646L | p72 | Capsid | 588 MITFALKPREEYQPS |
| B646L | p72 | Capsid | 589 ITFALKPREEYQPSG |
| B646L | p72 | Capsid | 590 TFALKPREEYQPSGH |
| B646L | p72 | Capsid | 591 FALKPREEYQPSGHI |
| B646L | p72 | Capsid | 592 ALKPREEYQPSGHIN |
| B646L | p72 | Capsid | 593 LKPREEYQPSGHINV |
| B646L | p72 | Capsid | 594 KPREEYQPSGHINVS |
| B646L | p72 | Capsid | 595 PREEYQPSGHINVSR |
| B646L | p72 | Capsid | 596 REEYQPSGHINVSRA |
| B646L | p72 | Capsid | 597 EEYQPSGHINVSRAR |
| B646L | p72 | Capsid | 598 EYQPSGHINVSRARE |
| B646L | p72 | Capsid | 599 YQPSGHINVSRAREF |
| B646L | p72 | Capsid | 600 QPSGHINVSRAREFY |
| B646L | p72 | Capsid | 601 PSGHINVSRAREFYI |
| B646L | p72 | Capsid | 602 SGHINVSRAREFYIS |
| B646L | p72 | Capsid | 603 GHINVSRAREFYISW |
| B646L | p72 | Capsid | 604 HINVSRAREFYISWD |
| B646L | p72 | Capsid | 605 INVSRAREFYISWDT |
| B646L | p72 | Capsid | 606 NVSRAREFYISWDTD |
| B646L | p72 | Capsid | 607 VSRAREFYISWDTDY |
| B646L | p72 | Capsid | 608 SRAREFYISWDTDYV |
| B646L | p72 | Capsid | 609 RAREFYISWDTDYVG |
| B646L | p72 | Capsid | 610 AREFYISWDTDYVGS |
| B646L | p72 | Capsid | 611 REFYISWDTDYVGSI |
| B646L | p72 | Capsid | 612 EFYISWDTDYVGSIT |
| B646L | p72 | Capsid | 613 FYISWDTDYVGSITT |
| B646L | p72 | Capsid | 614 YISWDTDYVGSITTA |
| B646L | p72 | Capsid | 615 ISWDTDYVGSITTAD |
| B646L | p72 | Capsid | 616 SWDTDYVGSITTADL |
| B646L | p72 | Capsid | 617 WDTDYVGSITTADLV |
| B646L | p72 | Capsid | 618 DTDYVGSITTADLVV |
| B646L | p72 | Capsid | 619 TDYVGSITTADLVVS |
| B646L | p72 | Capsid | 620 DYVGSITTADLVVSA |
| B646L | p72 | Capsid | 621 YVGSITTADLVVSAS |
| B646L | p72 | Capsid | 622 VGSITTADLVVSASA |
| B646L | p72 | Capsid | 623 GSITTADLVVSASAI |
| B646L | p72 | Capsid | 624 SITTADLVVSASAIN |
| B646L | p72 | Capsid | 625 ITTADLVVSASAINF |
| B646L | p72 | Capsid | 626 TTADLVVSASAINFL |
| B646L | p72 | Capsid | 627 TADLVVSASAINFLL |
| B646L | p72 | Capsid | 628 ADLVVSASAINFLLL |
| B646L | p72 | Capsid | 629 DLVVSASAINFLLLQ |
| B646L | p72 | Capsid | 630 LVVSASAINFLLLQN |
| B646L | p72 | Capsid | 631 VVSASAINFLLLQNG |
| B646L | p72 | Capsid | 632 VSASAINFLLLQNGS |
| B646L | p72 | Capsid | 633 SASAINFLLLQNGSA |
| B646L | p72 | Capsid | 634 ASAINFLLLQNGSAV |

| | | | | |
|---|---|---|---|---|
| B646L | p72 | Capsid | 635 | SAINFLLLQNGSAVL |
| B646L | p72 | Capsid | 636 | AINFLLLQNGSAVLR |
| B646L | p72 | Capsid | 637 | INFLLLQNGSAVLRY |
| B646L | p72 | Capsid | 638 | NFLLLQNGSAVLRYS |
| B646L | p72 | Capsid | 639 | FLLLQNGSAVLRYST |
| B646L | p72 | Capsid | 640 | LLLQNGSAVLRYSTG |
| B646L | p72 | Capsid | 641 | LLQNGSAVLRYSTGS |
| B646L | p72 | Capsid | 642 | LQNGSAVLRYSTGSG |
| B646L | p72 | Capsid | 643 | QNGSAVLRYSTGSGS |
| B646L | p72 | Capsid | 644 | NGSAVLRYSTGSGSG |
| B646L | p72 | Capsid | 645 | GSAVLRYSTGSGSGS |
| B646L | p72 | Capsid | 646 | SAVLRYSTGSGSGSG |
| E120R | E120R CDS | Capsid | 1 | GSGSGSGMADFNSPI |
| E120R | E120R CDS | Capsid | 2 | SGSGSGMADFNSPIQ |
| E120R | E120R CDS | Capsid | 3 | GSGSGMADFNSPIQY |
| E120R | E120R CDS | Capsid | 4 | SGSGMADFNSPIQYL |
| E120R | E120R CDS | Capsid | 5 | GSGMADFNSPIQYLK |
| E120R | E120R CDS | Capsid | 6 | SGMADFNSPIQYLKE |
| E120R | E120R CDS | Capsid | 7 | GMADFNSPIQYLKED |
| E120R | E120R CDS | Capsid | 8 | MADFNSPIQYLKEDS |
| E120R | E120R CDS | Capsid | 9 | ADFNSPIQYLKEDSR |
| E120R | E120R CDS | Capsid | 10 | DFNSPIQYLKEDSRD |
| E120R | E120R CDS | Capsid | 11 | FNSPIQYLKEDSRDR |
| E120R | E120R CDS | Capsid | 12 | NSPIQYLKEDSRDRT |
| E120R | E120R CDS | Capsid | 13 | SPIQYLKEDSRDRTS |
| E120R | E120R CDS | Capsid | 14 | PIQYLKEDSRDRTSI |
| E120R | E120R CDS | Capsid | 15 | IQYLKEDSRDRTSIG |
| E120R | E120R CDS | Capsid | 16 | QYLKEDSRDRTSIGS |
| E120R | E120R CDS | Capsid | 17 | YLKEDSRDRTSIGSL |
| E120R | E120R CDS | Capsid | 18 | LKEDSRDRTSIGSLE |
| E120R | E120R CDS | Capsid | 19 | KEDSRDRTSIGSLEY |
| E120R | E120R CDS | Capsid | 20 | EDSRDRTSIGSLEYD |
| E120R | E120R CDS | Capsid | 21 | DSRDRTSIGSLEYDE |
| E120R | E120R CDS | Capsid | 22 | SRDRTSIGSLEYDEN |
| E120R | E120R CDS | Capsid | 23 | RDRTSIGSLEYDENA |
| E120R | E120R CDS | Capsid | 24 | DRTSIGSLEYDENAD |
| E120R | E120R CDS | Capsid | 25 | RTSIGSLEYDENADT |
| E120R | E120R CDS | Capsid | 26 | TSIGSLEYDENADTM |
| E120R | E120R CDS | Capsid | 27 | SIGSLEYDENADTMI |
| E120R | E120R CDS | Capsid | 28 | IGSLEYDENADTMIP |
| E120R | E120R CDS | Capsid | 29 | GSLEYDENADTMIPS |
| E120R | E120R CDS | Capsid | 30 | SLEYDENADTMIPSF |
| E120R | E120R CDS | Capsid | 31 | LEYDENADTMIPSFA |
| E120R | E120R CDS | Capsid | 32 | EYDENADTMIPSFAA |
| E120R | E120R CDS | Capsid | 33 | YDENADTMIPSFAAG |
| E120R | E120R CDS | Capsid | 34 | DENADTMIPSFAAGL |
| E120R | E120R CDS | Capsid | 35 | ENADTMIPSFAAGLE |
| E120R | E120R CDS | Capsid | 36 | NADTMIPSFAAGLEE |
| E120R | E120R CDS | Capsid | 37 | ADTMIPSFAAGLEEF |
| E120R | E120R CDS | Capsid | 38 | DTMIPSFAAGLEEFE |
| E120R | E120R CDS | Capsid | 39 | TMIPSFAAGLEEFEP |
| E120R | E120R CDS | Capsid | 40 | MIPSFAAGLEEFEPI |
| E120R | E120R CDS | Capsid | 41 | IPSFAAGLEEFEPIP |
| E120R | E120R CDS | Capsid | 42 | PSFAAGLEEFEPIPD |
| E120R | E120R CDS | Capsid | 43 | SFAAGLEEFEPIPDY |
| E120R | E120R CDS | Capsid | 44 | FAAGLEEFEPIPDYD |
| E120R | E120R CDS | Capsid | 45 | AAGLEEFEPIPDYDP |
| E120R | E120R CDS | Capsid | 46 | AGLEEFEPIPDYDPT |
| E120R | E120R CDS | Capsid | 47 | GLEEFEPIPDYDPTT |
| E120R | E120R CDS | Capsid | 48 | LEEFEPIPDYDPTTS |
| E120R | E120R CDS | Capsid | 49 | EEFEPIPDYDPTTST |
| E120R | E120R CDS | Capsid | 50 | EFEPIPDYDPTTSTS |
| E120R | E120R CDS | Capsid | 51 | FEPIPDYDPTTSTSL |
| E120R | E120R CDS | Capsid | 52 | EPIPDYDPTTSTSLY |
| E120R | E120R CDS | Capsid | 53 | PIPDYDPTTSTSLYS |
| E120R | E120R CDS | Capsid | 54 | IPDYDPTTSTSLYSQ |
| E120R | E120R CDS | Capsid | 55 | PDYDPTTSTSLYSQL |

| | | | | |
|---|---|---|---|---|
| E120R | E120R CDS | Capsid | 56 | DYDPTTSTSLYSQLT |
| E120R | E120R CDS | Capsid | 57 | YDPTTSTSLYSQLTH |
| E120R | E120R CDS | Capsid | 58 | DPTTSTSLYSQLTHN |
| E120R | E120R CDS | Capsid | 59 | PTTSTSLYSQLTHNM |
| E120R | E120R CDS | Capsid | 60 | TTSTSLYSQLTHNME |
| E120R | E120R CDS | Capsid | 61 | TSTSLYSQLTHNMEK |
| E120R | E120R CDS | Capsid | 62 | STSLYSQLTHNMEKI |
| E120R | E120R CDS | Capsid | 63 | TSLYSQLTHNMEKIA |
| E120R | E120R CDS | Capsid | 64 | SLYSQLTHNMEKIAE |
| E120R | E120R CDS | Capsid | 65 | LYSQLTHNMEKIAEE |
| E120R | E120R CDS | Capsid | 66 | YSQLTHNMEKIAEEE |
| E120R | E120R CDS | Capsid | 67 | SQLTHNMEKIAEEED |
| E120R | E120R CDS | Capsid | 68 | QLTHNMEKIAEEEDS |
| E120R | E120R CDS | Capsid | 69 | LTHNMEKIAEEEDSN |
| E120R | E120R CDS | Capsid | 70 | THNMEKIAEEEDSNF |
| E120R | E120R CDS | Capsid | 71 | HNMEKIAEEEDSNFL |
| E120R | E120R CDS | Capsid | 72 | NMEKIAEEEDSNFLH |
| E120R | E120R CDS | Capsid | 73 | MEKIAEEEDSNFLHD |
| E120R | E120R CDS | Capsid | 74 | EKIAEEEDSNFLHDT |
| E120R | E120R CDS | Capsid | 75 | KIAEEEDSNFLHDTR |
| E120R | E120R CDS | Capsid | 76 | IAEEEDSNFLHDTRE |
| E120R | E120R CDS | Capsid | 77 | AEEEDSNFLHDTREF |
| E120R | E120R CDS | Capsid | 78 | EEEDSNFLHDTREFT |
| E120R | E120R CDS | Capsid | 79 | EEDSNFLHDTREFTS |
| E120R | E120R CDS | Capsid | 80 | EDSNFLHDTREFTSL |
| E120R | E120R CDS | Capsid | 81 | DSNFLHDTREFTSLV |
| E120R | E120R CDS | Capsid | 82 | SNFLHDTREFTSLVP |
| E120R | E120R CDS | Capsid | 83 | NFLHDTREFTSLVPD |
| E120R | E120R CDS | Capsid | 84 | FLHDTREFTSLVPDE |
| E120R | E120R CDS | Capsid | 85 | LHDTREFTSLVPDEA |
| E120R | E120R CDS | Capsid | 86 | HDTREFTSLVPDEAD |
| E120R | E120R CDS | Capsid | 87 | DTREFTSLVPDEADN |
| E120R | E120R CDS | Capsid | 88 | TREFTSLVPDEADNK |
| E120R | E120R CDS | Capsid | 89 | REFTSLVPDEADNKP |
| E120R | E120R CDS | Capsid | 90 | EFTSLVPDEADNKPE |
| E120R | E120R CDS | Capsid | 91 | FTSLVPDEADNKPED |
| E120R | E120R CDS | Capsid | 92 | TSLVPDEADNKPEDD |
| E120R | E120R CDS | Capsid | 93 | SLVPDEADNKPEDDE |
| E120R | E120R CDS | Capsid | 94 | LVPDEADNKPEDDEE |
| E120R | E120R CDS | Capsid | 95 | VPDEADNKPEDDEES |
| E120R | E120R CDS | Capsid | 96 | PDEADNKPEDDEESG |
| E120R | E120R CDS | Capsid | 97 | DEADNKPEDDEESGA |
| E120R | E120R CDS | Capsid | 98 | EADNKPEDDEESGAK |
| E120R | E120R CDS | Capsid | 99 | ADNKPEDDEESGAKP |
| E120R | E120R CDS | Capsid | 100 | DNKPEDDEESGAKPK |
| E120R | E120R CDS | Capsid | 101 | NKPEDDEESGAKPKK |
| E120R | E120R CDS | Capsid | 102 | KPEDDEESGAKPKKK |
| E120R | E120R CDS | Capsid | 103 | PEDDEESGAKPKKKK |
| E120R | E120R CDS | Capsid | 104 | EDDEESGAKPKKKKH |
| E120R | E120R CDS | Capsid | 105 | DDEESGAKPKKKKHL |
| E120R | E120R CDS | Capsid | 106 | DEESGAKPKKKKHLF |
| E120R | E120R CDS | Capsid | 107 | EESGAKPKKKKHLFP |
| E120R | E120R CDS | Capsid | 108 | ESGAKPKKKKHLFPK |
| E120R | E120R CDS | Capsid | 109 | SGAKPKKKKHLFPKL |
| E120R | E120R CDS | Capsid | 110 | GAKPKKKKHLFPKLS |
| E120R | E120R CDS | Capsid | 111 | AKPKKKKHLFPKLSS |
| E120R | E120R CDS | Capsid | 112 | KPKKKKHLFPKLSSH |
| E120R | E120R CDS | Capsid | 113 | PKKKKHLFPKLSSHK |
| E120R | E120R CDS | Capsid | 114 | KKKKHLFPKLSSHKS |
| E120R | E120R CDS | Capsid | 115 | KKKHLFPKLSSHKSK |
| E120R | E120R CDS | Capsid | 116 | KKHLFPKLSSHKSKG |
| E120R | E120R CDS | Capsid | 117 | KHLFPKLSSHKSKGS |
| E120R | E120R CDS | Capsid | 118 | HLFPKLSSHKSKGSG |
| E120R | E120R CDS | Capsid | 119 | LFPKLSSHKSKGSGS |
| E120R | E120R CDS | Capsid | 120 | FPKLSSHKSKGSGSG |
| E120R | E120R CDS | Capsid | 121 | PKLSSHKSKGSGSGS |
| E120R | E120R CDS | Capsid | 122 | KLSSHKSKGSGSGSG |

| | | | | |
|---|---|---|---|---|
| B438L | B438L CDS | Capsid | 123 | GSGSGSGMYHDYASK |
| B438L | B438L CDS | Capsid | 124 | SGSGSGMYHDYASKL |
| B438L | B438L CDS | Capsid | 125 | GSGSGMYHDYASKLL |
| B438L | B438L CDS | Capsid | 126 | SGSGMYHDYASKLLA |
| B438L | B438L CDS | Capsid | 127 | GSGMYHDYASKLLAD |
| B438L | B438L CDS | Capsid | 128 | SGMYHDYASKLLADY |
| B438L | B438L CDS | Capsid | 129 | GMYHDYASKLLADYR |
| B438L | B438L CDS | Capsid | 130 | MYHDYASKLLADYRS |
| B438L | B438L CDS | Capsid | 131 | YHDYASKLLADYRSD |
| B438L | B438L CDS | Capsid | 132 | HDYASKLLADYRSDP |
| B438L | B438L CDS | Capsid | 133 | DYASKLLADYRSDPP |
| B438L | B438L CDS | Capsid | 134 | YASKLLADYRSDPPL |
| B438L | B438L CDS | Capsid | 135 | ASKLLADYRSDPPLW |
| B438L | B438L CDS | Capsid | 136 | SKLLADYRSDPPLWE |
| B438L | B438L CDS | Capsid | 137 | KLLADYRSDPPLWES |
| B438L | B438L CDS | Capsid | 138 | LLADYRSDPPLWESD |
| B438L | B438L CDS | Capsid | 139 | LADYRSDPPLWESDL |
| B438L | B438L CDS | Capsid | 140 | ADYRSDPPLWESDLP |
| B438L | B438L CDS | Capsid | 141 | DYRSDPPLWESDLPR |
| B438L | B438L CDS | Capsid | 142 | YRSDPPLWESDLPRH |
| B438L | B438L CDS | Capsid | 143 | RSDPPLWESDLPRHN |
| B438L | B438L CDS | Capsid | 144 | SDPPLWESDLPRHNR |
| B438L | B438L CDS | Capsid | 145 | DPPLWESDLPRHNRY |
| B438L | B438L CDS | Capsid | 146 | PPLWESDLPRHNRYS |
| B438L | B438L CDS | Capsid | 147 | PLWESDLPRHNRYSD |
| B438L | B438L CDS | Capsid | 148 | LWESDLPRHNRYSDN |
| B438L | B438L CDS | Capsid | 149 | WESDLPRHNRYSDNI |
| B438L | B438L CDS | Capsid | 150 | ESDLPRHNRYSDNIL |
| B438L | B438L CDS | Capsid | 151 | SDLPRHNRYSDNILN |
| B438L | B438L CDS | Capsid | 152 | DLPRHNRYSDNILNS |
| B438L | B438L CDS | Capsid | 153 | LPRHNRYSDNILNSR |
| B438L | B438L CDS | Capsid | 154 | PRHNRYSDNILNSRY |
| B438L | B438L CDS | Capsid | 155 | RHNRYSDNILNSRYC |
| B438L | B438L CDS | Capsid | 156 | HNRYSDNILNSRYCG |
| B438L | B438L CDS | Capsid | 157 | NRYSDNILNSRYCGN |
| B438L | B438L CDS | Capsid | 158 | RYSDNILNSRYCGNK |
| B438L | B438L CDS | Capsid | 159 | YSDNILNSRYCGNKN |
| B438L | B438L CDS | Capsid | 160 | SDNILNSRYCGNKNG |
| B438L | B438L CDS | Capsid | 161 | DNILNSRYCGNKNGA |
| B438L | B438L CDS | Capsid | 162 | NILNSRYCGNKNGAA |
| B438L | B438L CDS | Capsid | 163 | ILNSRYCGNKNGAAP |
| B438L | B438L CDS | Capsid | 164 | LNSRYCGNKNGAAPV |
| B438L | B438L CDS | Capsid | 165 | NSRYCGNKNGAAPVY |
| B438L | B438L CDS | Capsid | 166 | SRYCGNKNGAAPVYN |
| B438L | B438L CDS | Capsid | 167 | RYCGNKNGAAPVYNE |
| B438L | B438L CDS | Capsid | 168 | YCGNKNGAAPVYNEY |
| B438L | B438L CDS | Capsid | 169 | CGNKNGAAPVYNEYT |
| B438L | B438L CDS | Capsid | 170 | GNKNGAAPVYNEYTN |
| B438L | B438L CDS | Capsid | 171 | NKNGAAPVYNEYTNS |
| B438L | B438L CDS | Capsid | 172 | KNGAAPVYNEYTNSP |
| B438L | B438L CDS | Capsid | 173 | NGAAPVYNEYTNSPE |
| B438L | B438L CDS | Capsid | 174 | GAAPVYNEYTNSPEK |
| B438L | B438L CDS | Capsid | 175 | AAPVYNEYTNSPEKA |
| B438L | B438L CDS | Capsid | 176 | APVYNEYTNSPEKAE |
| B438L | B438L CDS | Capsid | 177 | PVYNEYTNSPEKAEK |
| B438L | B438L CDS | Capsid | 178 | VYNEYTNSPEKAEKG |
| B438L | B438L CDS | Capsid | 179 | YNEYTNSPEKAEKGL |
| B438L | B438L CDS | Capsid | 180 | NEYTNSPEKAEKGLQ |
| B438L | B438L CDS | Capsid | 181 | EYTNSPEKAEKGLQL |
| B438L | B438L CDS | Capsid | 182 | YTNSPEKAEKGLQLS |
| B438L | B438L CDS | Capsid | 183 | TNSPEKAEKGLQLSD |
| B438L | B438L CDS | Capsid | 184 | NSPEKAEKGLQLSDL |
| B438L | B438L CDS | Capsid | 185 | SPEKAEKGLQLSDLR |
| B438L | B438L CDS | Capsid | 186 | PEKAEKGLQLSDLRN |
| B438L | B438L CDS | Capsid | 187 | EKAEKGLQLSDLRNF |
| B438L | B438L CDS | Capsid | 188 | KAEKGLQLSDLRNFS |
| B438L | B438L CDS | Capsid | 189 | AEKGLQLSDLRNFSF |

| | | | | |
|---|---|---|---|---|
| B438L | B438L CDS | Capsid | 190 | EKGLQLSDLRNFSFM |
| B438L | B438L CDS | Capsid | 191 | KGLQLSDLRNFSFML |
| B438L | B438L CDS | Capsid | 192 | GLQLSDLRNFSFMLN |
| B438L | B438L CDS | Capsid | 193 | LQLSDLRNFSFMLNP |
| B438L | B438L CDS | Capsid | 194 | QLSDLRNFSFMLNPQ |
| B438L | B438L CDS | Capsid | 195 | LSDLRNFSFMLNPQH |
| B438L | B438L CDS | Capsid | 196 | SDLRNFSFMLNPQHK |
| B438L | B438L CDS | Capsid | 197 | DLRNFSFMLNPQHKN |
| B438L | B438L CDS | Capsid | 198 | LRNFSFMLNPQHKNI |
| B438L | B438L CDS | Capsid | 199 | RNFSFMLNPQHKNIG |
| B438L | B438L CDS | Capsid | 200 | NFSFMLNPQHKNIGY |
| B438L | B438L CDS | Capsid | 201 | FSFMLNPQHKNIGYG |
| B438L | B438L CDS | Capsid | 202 | SFMLNPQHKNIGYGD |
| B438L | B438L CDS | Capsid | 203 | FMLNPQHKNIGYGDA |
| B438L | B438L CDS | Capsid | 204 | MLNPQHKNIGYGDAQ |
| B438L | B438L CDS | Capsid | 205 | LNPQHKNIGYGDAQD |
| B438L | B438L CDS | Capsid | 206 | NPQHKNIGYGDAQDL |
| B438L | B438L CDS | Capsid | 207 | PQHKNIGYGDAQDLE |
| B438L | B438L CDS | Capsid | 208 | QHKNIGYGDAQDLEP |
| B438L | B438L CDS | Capsid | 209 | HKNIGYGDAQDLEPY |
| B438L | B438L CDS | Capsid | 210 | KNIGYGDAQDLEPYS |
| B438L | B438L CDS | Capsid | 211 | NIGYGDAQDLEPYSS |
| B438L | B438L CDS | Capsid | 212 | IGYGDAQDLEPYSSI |
| B438L | B438L CDS | Capsid | 213 | GYGDAQDLEPYSSIP |
| B438L | B438L CDS | Capsid | 214 | YGDAQDLEPYSSIPK |
| B438L | B438L CDS | Capsid | 215 | GDAQDLEPYSSIPKN |
| B438L | B438L CDS | Capsid | 216 | DAQDLEPYSSIPKNK |
| B438L | B438L CDS | Capsid | 217 | AQDLEPYSSIPKNKL |
| B438L | B438L CDS | Capsid | 218 | QDLEPYSSIPKNKLF |
| B438L | B438L CDS | Capsid | 219 | DLEPYSSIPKNKLFN |
| B438L | B438L CDS | Capsid | 220 | LEPYSSIPKNKLFNH |
| B438L | B438L CDS | Capsid | 221 | EPYSSIPKNKLFNHF |
| B438L | B438L CDS | Capsid | 222 | PYSSIPKNKLFNHFK |
| B438L | B438L CDS | Capsid | 223 | YSSIPKNKLFNHFKN |
| B438L | B438L CDS | Capsid | 224 | SSIPKNKLFNHFKNH |
| B438L | B438L CDS | Capsid | 225 | SIPKNKLFNHFKNHR |
| B438L | B438L CDS | Capsid | 226 | IPKNKLFNHFKNHRP |
| B438L | B438L CDS | Capsid | 227 | PKNKLFNHFKNHRPA |
| B438L | B438L CDS | Capsid | 228 | KNKLFNHFKNHRPAF |
| B438L | B438L CDS | Capsid | 229 | NKLFNHFKNHRPAFS |
| B438L | B438L CDS | Capsid | 230 | KLFNHFKNHRPAFST |
| B438L | B438L CDS | Capsid | 231 | LFNHFKNHRPAFSTH |
| B438L | B438L CDS | Capsid | 232 | FNHFKNHRPAFSTHT |
| B438L | B438L CDS | Capsid | 233 | NHFKNHRPAFSTHTE |
| B438L | B438L CDS | Capsid | 234 | HFKNHRPAFSTHTEN |
| B438L | B438L CDS | Capsid | 235 | FKNHRPAFSTHTENL |
| B438L | B438L CDS | Capsid | 236 | KNHRPAFSTHTENLI |
| B438L | B438L CDS | Capsid | 237 | NHRPAFSTHTENLIR |
| B438L | B438L CDS | Capsid | 238 | HRPAFSTHTENLIRR |
| B438L | B438L CDS | Capsid | 239 | RPAFSTHTENLIRRN |
| B438L | B438L CDS | Capsid | 240 | PAFSTHTENLIRRNV |
| B438L | B438L CDS | Capsid | 241 | AFSTHTENLIRRNVV |
| B438L | B438L CDS | Capsid | 242 | FSTHTENLIRRNVVR |
| B438L | B438L CDS | Capsid | 243 | STHTENLIRRNVVRT |
| B438L | B438L CDS | Capsid | 244 | THTENLIRRNVVRTE |
| B438L | B438L CDS | Capsid | 245 | HTENLIRRNVVRTEK |
| B438L | B438L CDS | Capsid | 246 | TENLIRRNVVRTEKK |
| B438L | B438L CDS | Capsid | 247 | ENLIRRNVVRTEKKT |
| B438L | B438L CDS | Capsid | 248 | NLIRRNVVRTEKKTF |
| B438L | B438L CDS | Capsid | 249 | LIRRNVVRTEKKTFP |
| B438L | B438L CDS | Capsid | 250 | IRRNVVRTEKKTFPQ |
| B438L | B438L CDS | Capsid | 251 | RRNVVRTEKKTFPQV |
| B438L | B438L CDS | Capsid | 252 | RNVVRTEKKTFPQVA |
| B438L | B438L CDS | Capsid | 253 | NVVRTEKKTFPQVAS |
| B438L | B438L CDS | Capsid | 254 | VVRTEKKTFPQVASL |
| B438L | B438L CDS | Capsid | 255 | VRTEKKTFPQVASLK |
| B438L | B438L CDS | Capsid | 256 | RTEKKTFPQVASLKG |

| | | | |
|---|---|---|---|
| B438L | B438L CDS | Capsid | 257 TEKKTFPQVASLKGT |
| B438L | B438L CDS | Capsid | 258 EKKTFPQVASLKGTQ |
| B438L | B438L CDS | Capsid | 259 KKTFPQVASLKGTQK |
| B438L | B438L CDS | Capsid | 260 KTFPQVASLKGTQKN |
| B438L | B438L CDS | Capsid | 261 TFPQVASLKGTQKNC |
| B438L | B438L CDS | Capsid | 262 FPQVASLKGTQKNCL |
| B438L | B438L CDS | Capsid | 263 PQVASLKGTQKNCLT |
| B438L | B438L CDS | Capsid | 264 QVASLKGTQKNCLTQ |
| B438L | B438L CDS | Capsid | 265 VASLKGTQKNCLTQP |
| B438L | B438L CDS | Capsid | 266 ASLKGTQKNCLTQPS |
| B438L | B438L CDS | Capsid | 267 SLKGTQKNCLTQPSS |
| B438L | B438L CDS | Capsid | 268 LKGTQKNCLTQPSSL |
| B438L | B438L CDS | Capsid | 269 KGTQKNCLTQPSSLP |
| B438L | B438L CDS | Capsid | 270 GTQKNCLTQPSSLPS |
| B438L | B438L CDS | Capsid | 271 TQKNCLTQPSSLPSL |
| B438L | B438L CDS | Capsid | 272 QKNCLTQPSSLPSLK |
| B438L | B438L CDS | Capsid | 273 KNCLTQPSSLPSLKN |
| B438L | B438L CDS | Capsid | 274 NCLTQPSSLPSLKNP |
| B438L | B438L CDS | Capsid | 275 CLTQPSSLPSLKNPK |
| B438L | B438L CDS | Capsid | 276 LTQPSSLPSLKNPKN |
| B438L | B438L CDS | Capsid | 277 TQPSSLPSLKNPKNS |
| B438L | B438L CDS | Capsid | 278 QPSSLPSLKNPKNSS |
| B438L | B438L CDS | Capsid | 279 PSSLPSLKNPKNSSV |
| B438L | B438L CDS | Capsid | 280 SSLPSLKNPKNSSVP |
| B438L | B438L CDS | Capsid | 281 SLPSLKNPKNSSVPS |
| B438L | B438L CDS | Capsid | 282 LPSLKNPKNSSVPST |
| B438L | B438L CDS | Capsid | 283 PSLKNPKNSSVPSTR |
| B438L | B438L CDS | Capsid | 284 SLKNPKNSSVPSTRF |
| B438L | B438L CDS | Capsid | 285 LKNPKNSSVPSTRFS |
| B438L | B438L CDS | Capsid | 286 KNPKNSSVPSTRFSE |
| B438L | B438L CDS | Capsid | 287 NPKNSSVPSTRFSEH |
| B438L | B438L CDS | Capsid | 288 PKNSSVPSTRFSEHT |
| B438L | B438L CDS | Capsid | 289 KNSSVPSTRFSEHTK |
| B438L | B438L CDS | Capsid | 290 NSSVPSTRFSEHTKF |
| B438L | B438L CDS | Capsid | 291 SSVPSTRFSEHTKFF |
| B438L | B438L CDS | Capsid | 292 SVPSTRFSEHTKFFS |
| B438L | B438L CDS | Capsid | 293 VPSTRFSEHTKFFSY |
| B438L | B438L CDS | Capsid | 294 PSTRFSEHTKFFSYE |
| B438L | B438L CDS | Capsid | 295 STRFSEHTKFFSYED |
| B438L | B438L CDS | Capsid | 296 TRFSEHTKFFSYEDL |
| B438L | B438L CDS | Capsid | 297 RFSEHTKFFSYEDLP |
| B438L | B438L CDS | Capsid | 298 FSEHTKFFSYEDLPK |
| B438L | B438L CDS | Capsid | 299 SEHTKFFSYEDLPKL |
| B438L | B438L CDS | Capsid | 300 EHTKFFSYEDLPKLR |
| B438L | B438L CDS | Capsid | 301 HTKFFSYEDLPKLRT |
| B438L | B438L CDS | Capsid | 302 TKFFSYEDLPKLRTK |
| B438L | B438L CDS | Capsid | 303 KFFSYEDLPKLRTKG |
| B438L | B438L CDS | Capsid | 304 FFSYEDLPKLRTKGT |
| B438L | B438L CDS | Capsid | 305 FSYEDLPKLRTKGTI |
| B438L | B438L CDS | Capsid | 306 SYEDLPKLRTKGTIK |
| B438L | B438L CDS | Capsid | 307 YEDLPKLRTKGTIKH |
| B438L | B438L CDS | Capsid | 308 EDLPKLRTKGTIKHE |
| B438L | B438L CDS | Capsid | 309 DLPKLRTKGTIKHEQ |
| B438L | B438L CDS | Capsid | 310 LPKLRTKGTIKHEQH |
| B438L | B438L CDS | Capsid | 311 PKLRTKGTIKHEQHL |
| B438L | B438L CDS | Capsid | 312 KLRTKGTIKHEQHLG |
| B438L | B438L CDS | Capsid | 313 LRTKGTIKHEQHLGD |
| B438L | B438L CDS | Capsid | 314 RTKGTIKHEQHLGDQ |
| B438L | B438L CDS | Capsid | 315 TKGTIKHEQHLGDQM |
| B438L | B438L CDS | Capsid | 316 KGTIKHEQHLGDQMP |
| B438L | B438L CDS | Capsid | 317 GTIKHEQHLGDQMPG |
| B438L | B438L CDS | Capsid | 318 TIKHEQHLGDQMPGQ |
| B438L | B438L CDS | Capsid | 319 IKHEQHLGDQMPGQH |
| B438L | B438L CDS | Capsid | 320 KHEQHLGDQMPGQHY |
| B438L | B438L CDS | Capsid | 321 HEQHLGDQMPGQHYN |
| B438L | B438L CDS | Capsid | 322 EQHLGDQMPGQHYNG |
| B438L | B438L CDS | Capsid | 323 QHLGDQMPGQHYNGY |

| | | | | |
|---|---|---|---|---|
| B438L | B438L CDS | Capsid | 324 | HLGDQMPGQHYNGYI |
| B438L | B438L CDS | Capsid | 325 | LGDQMPGQHYNGYIP |
| B438L | B438L CDS | Capsid | 326 | GDQMPGQHYNGYIPH |
| B438L | B438L CDS | Capsid | 327 | DQMPGQHYNGYIPHK |
| B438L | B438L CDS | Capsid | 328 | QMPGQHYNGYIPHKD |
| B438L | B438L CDS | Capsid | 329 | MPGQHYNGYIPHKDV |
| B438L | B438L CDS | Capsid | 330 | PGQHYNGYIPHKDVY |
| B438L | B438L CDS | Capsid | 331 | GQHYNGYIPHKDVYN |
| B438L | B438L CDS | Capsid | 332 | QHYNGYIPHKDVYNI |
| B438L | B438L CDS | Capsid | 333 | HYNGYIPHKDVYNIL |
| B438L | B438L CDS | Capsid | 334 | YNGYIPHKDVYNILC |
| B438L | B438L CDS | Capsid | 335 | NGYIPHKDVYNILCL |
| B438L | B438L CDS | Capsid | 336 | GYIPHKDVYNILCLA |
| B438L | B438L CDS | Capsid | 337 | YIPHKDVYNILCLAH |
| B438L | B438L CDS | Capsid | 338 | IPHKDVYNILCLAHN |
| B438L | B438L CDS | Capsid | 339 | PHKDVYNILCLAHNL |
| B438L | B438L CDS | Capsid | 340 | HKDVYNILCLAHNLP |
| B438L | B438L CDS | Capsid | 341 | KDVYNILCLAHNLPA |
| B438L | B438L CDS | Capsid | 342 | DVYNILCLAHNLPAS |
| B438L | B438L CDS | Capsid | 343 | VYNILCLAHNLPASV |
| B438L | B438L CDS | Capsid | 344 | YNILCLAHNLPASVE |
| B438L | B438L CDS | Capsid | 345 | NILCLAHNLPASVEK |
| B438L | B438L CDS | Capsid | 346 | ILCLAHNLPASVEKG |
| B438L | B438L CDS | Capsid | 347 | LCLAHNLPASVEKGI |
| B438L | B438L CDS | Capsid | 348 | CLAHNLPASVEKGIA |
| B438L | B438L CDS | Capsid | 349 | LAHNLPASVEKGIAG |
| B438L | B438L CDS | Capsid | 350 | AHNLPASVEKGIAGR |
| B438L | B438L CDS | Capsid | 351 | HNLPASVEKGIAGRG |
| B438L | B438L CDS | Capsid | 352 | NLPASVEKGIAGRGI |
| B438L | B438L CDS | Capsid | 353 | LPASVEKGIAGRGIP |
| B438L | B438L CDS | Capsid | 354 | PASVEKGIAGRGIPL |
| B438L | B438L CDS | Capsid | 355 | ASVEKGIAGRGIPLG |
| B438L | B438L CDS | Capsid | 356 | SVEKGIAGRGIPLGN |
| B438L | B438L CDS | Capsid | 357 | VEKGIAGRGIPLGNP |
| B438L | B438L CDS | Capsid | 358 | EKGIAGRGIPLGNPH |
| B438L | B438L CDS | Capsid | 359 | KGIAGRGIPLGNPHV |
| B438L | B438L CDS | Capsid | 360 | GIAGRGIPLGNPHVK |
| B438L | B438L CDS | Capsid | 361 | IAGRGIPLGNPHVKP |
| B438L | B438L CDS | Capsid | 362 | AGRGIPLGNPHVKPN |
| B438L | B438L CDS | Capsid | 363 | GRGIPLGNPHVKPNI |
| B438L | B438L CDS | Capsid | 364 | RGIPLGNPHVKPNIE |
| B438L | B438L CDS | Capsid | 365 | GIPLGNPHVKPNIEQ |
| B438L | B438L CDS | Capsid | 366 | IPLGNPHVKPNIEQE |
| B438L | B438L CDS | Capsid | 367 | PLGNPHVKPNIEQEL |
| B438L | B438L CDS | Capsid | 368 | LGNPHVKPNIEQELI |
| B438L | B438L CDS | Capsid | 369 | GNPHVKPNIEQELIK |
| B438L | B438L CDS | Capsid | 370 | NPHVKPNIEQELIKS |
| B438L | B438L CDS | Capsid | 371 | PHVKPNIEQELIKST |
| B438L | B438L CDS | Capsid | 372 | HVKPNIEQELIKSTS |
| B438L | B438L CDS | Capsid | 373 | VKPNIEQELIKSTST |
| B438L | B438L CDS | Capsid | 374 | KPNIEQELIKSTSTY |
| B438L | B438L CDS | Capsid | 375 | PNIEQELIKSTSTYT |
| B438L | B438L CDS | Capsid | 376 | NIEQELIKSTSTYTD |
| B438L | B438L CDS | Capsid | 377 | IEQELIKSTSTYTDV |
| B438L | B438L CDS | Capsid | 378 | EQELIKSTSTYTDVP |
| B438L | B438L CDS | Capsid | 379 | QELIKSTSTYTDVPM |
| B438L | B438L CDS | Capsid | 380 | ELIKSTSTYTDVPML |
| B438L | B438L CDS | Capsid | 381 | LIKSTSTYTDVPMLG |
| B438L | B438L CDS | Capsid | 382 | IKSTSTYTDVPMLGP |
| B438L | B438L CDS | Capsid | 383 | KSTSTYTDVPMLGPL |
| B438L | B438L CDS | Capsid | 384 | STSTYTDVPMLGPLP |
| B438L | B438L CDS | Capsid | 385 | TSTYTDVPMLGPLPP |
| B438L | B438L CDS | Capsid | 386 | STYTDVPMLGPLPPK |
| B438L | B438L CDS | Capsid | 387 | TYTDVPMLGPLPPKD |
| B438L | B438L CDS | Capsid | 388 | YTDVPMLGPLPPKDS |
| B438L | B438L CDS | Capsid | 389 | TDVPMLGPLPPKDSQ |
| B438L | B438L CDS | Capsid | 390 | DVPMLGPLPPKDSQH |

| | | | | |
|---|---|---|---|---|
| B438L | B438L CDS | Capsid | 391 | VPMLGPLPPKDSQHG |
| B438L | B438L CDS | Capsid | 392 | PMLGPLPPKDSQHGR |
| B438L | B438L CDS | Capsid | 393 | MLGPLPPKDSQHGRE |
| B438L | B438L CDS | Capsid | 394 | LGPLPPKDSQHGREY |
| B438L | B438L CDS | Capsid | 395 | GPLPPKDSQHGREYQ |
| B438L | B438L CDS | Capsid | 396 | PLPPKDSQHGREYQE |
| B438L | B438L CDS | Capsid | 397 | LPPKDSQHGREYQEF |
| B438L | B438L CDS | Capsid | 398 | PPKDSQHGREYQEFS |
| B438L | B438L CDS | Capsid | 399 | PKDSQHGREYQEFSA |
| B438L | B438L CDS | Capsid | 400 | KDSQHGREYQEFSAN |
| B438L | B438L CDS | Capsid | 401 | DSQHGREYQEFSANR |
| B438L | B438L CDS | Capsid | 402 | SQHGREYQEFSANRH |
| B438L | B438L CDS | Capsid | 403 | QHGREYQEFSANRHM |
| B438L | B438L CDS | Capsid | 404 | HGREYQEFSANRHML |
| B438L | B438L CDS | Capsid | 405 | GREYQEFSANRHMLQ |
| B438L | B438L CDS | Capsid | 406 | REYQEFSANRHMLQV |
| B438L | B438L CDS | Capsid | 407 | EYQEFSANRHMLQVS |
| B438L | B438L CDS | Capsid | 408 | YQEFSANRHMLQVSN |
| B438L | B438L CDS | Capsid | 409 | QEFSANRHMLQVSNI |
| B438L | B438L CDS | Capsid | 410 | EFSANRHMLQVSNIL |
| B438L | B438L CDS | Capsid | 411 | FSANRHMLQVSNILH |
| B438L | B438L CDS | Capsid | 412 | SANRHMLQVSNILHS |
| B438L | B438L CDS | Capsid | 413 | ANRHMLQVSNILHSV |
| B438L | B438L CDS | Capsid | 414 | NRHMLQVSNILHSVF |
| B438L | B438L CDS | Capsid | 415 | RHMLQVSNILHSVFA |
| B438L | B438L CDS | Capsid | 416 | HMLQVSNILHSVFAN |
| B438L | B438L CDS | Capsid | 417 | MLQVSNILHSVFANH |
| B438L | B438L CDS | Capsid | 418 | LQVSNILHSVFANHS |
| B438L | B438L CDS | Capsid | 419 | QVSNILHSVFANHSI |
| B438L | B438L CDS | Capsid | 420 | VSNILHSVFANHSIK |
| B438L | B438L CDS | Capsid | 421 | SNILHSVFANHSIKP |
| B438L | B438L CDS | Capsid | 422 | NILHSVFANHSIKPQ |
| B438L | B438L CDS | Capsid | 423 | ILHSVFANHSIKPQI |
| B438L | B438L CDS | Capsid | 424 | LHSVFANHSIKPQIL |
| B438L | B438L CDS | Capsid | 425 | HSVFANHSIKPQILE |
| B438L | B438L CDS | Capsid | 426 | SVFANHSIKPQILED |
| B438L | B438L CDS | Capsid | 427 | VFANHSIKPQILEDI |
| B438L | B438L CDS | Capsid | 428 | FANHSIKPQILEDIP |
| B438L | B438L CDS | Capsid | 429 | ANHSIKPQILEDIPV |
| B438L | B438L CDS | Capsid | 430 | NHSIKPQILEDIPVL |
| B438L | B438L CDS | Capsid | 431 | HSIKPQILEDIPVLN |
| B438L | B438L CDS | Capsid | 432 | SIKPQILEDIPVLNA |
| B438L | B438L CDS | Capsid | 433 | IKPQILEDIPVLNAQ |
| B438L | B438L CDS | Capsid | 434 | KPQILEDIPVLNAQL |
| B438L | B438L CDS | Capsid | 435 | PQILEDIPVLNAQLT |
| B438L | B438L CDS | Capsid | 436 | QILEDIPVLNAQLTS |
| B438L | B438L CDS | Capsid | 437 | ILEDIPVLNAQLTSI |
| B438L | B438L CDS | Capsid | 438 | LEDIPVLNAQLTSIK |
| B438L | B438L CDS | Capsid | 439 | EDIPVLNAQLTSIKP |
| B438L | B438L CDS | Capsid | 440 | DIPVLNAQLTSIKPV |
| B438L | B438L CDS | Capsid | 441 | IPVLNAQLTSIKPVS |
| B438L | B438L CDS | Capsid | 442 | PVLNAQLTSIKPVSP |
| B438L | B438L CDS | Capsid | 443 | VLNAQLTSIKPVSPF |
| B438L | B438L CDS | Capsid | 444 | LNAQLTSIKPVSPFL |
| B438L | B438L CDS | Capsid | 445 | NAQLTSIKPVSPFLN |
| B438L | B438L CDS | Capsid | 446 | AQLTSIKPVSPFLNK |
| B438L | B438L CDS | Capsid | 447 | QLTSIKPVSPFLNKA |
| B438L | B438L CDS | Capsid | 448 | LTSIKPVSPFLNKAY |
| B438L | B438L CDS | Capsid | 449 | TSIKPVSPFLNKAYQ |
| B438L | B438L CDS | Capsid | 450 | SIKPVSPFLNKAYQT |
| B438L | B438L CDS | Capsid | 451 | IKPVSPFLNKAYQTH |
| B438L | B438L CDS | Capsid | 452 | KPVSPFLNKAYQTHY |
| B438L | B438L CDS | Capsid | 453 | PVSPFLNKAYQTHYM |
| B438L | B438L CDS | Capsid | 454 | VSPFLNKAYQTHYME |
| B438L | B438L CDS | Capsid | 455 | SPFLNKAYQTHYMEN |
| B438L | B438L CDS | Capsid | 456 | PFLNKAYQTHYMENI |
| B438L | B438L CDS | Capsid | 457 | FLNKAYQTHYMENIV |

| | | | | |
|---|---|---|---|---|
| B438L | B438L CDS | Capsid | 458 | LNKAYQTHYMENIVT |
| B438L | B438L CDS | Capsid | 459 | NKAYQTHYMENIVTL |
| B438L | B438L CDS | Capsid | 460 | KAYQTHYMENIVTLV |
| B438L | B438L CDS | Capsid | 461 | AYQTHYMENIVTLVP |
| B438L | B438L CDS | Capsid | 462 | YQTHYMENIVTLVPR |
| B438L | B438L CDS | Capsid | 463 | QTHYMENIVTLVPRF |
| B438L | B438L CDS | Capsid | 464 | THYMENIVTLVPRFK |
| B438L | B438L CDS | Capsid | 465 | HYMENIVTLVPRFKS |
| B438L | B438L CDS | Capsid | 466 | YMENIVTLVPRFKSI |
| B438L | B438L CDS | Capsid | 467 | MENIVTLVPRFKSIA |
| B438L | B438L CDS | Capsid | 468 | ENIVTLVPRFKSIAN |
| B438L | B438L CDS | Capsid | 469 | NIVTLVPRFKSIANY |
| B438L | B438L CDS | Capsid | 470 | IVTLVPRFKSIANYS |
| B438L | B438L CDS | Capsid | 471 | VTLVPRFKSIANYSS |
| B438L | B438L CDS | Capsid | 472 | TLVPRFKSIANYSSP |
| B438L | B438L CDS | Capsid | 473 | LVPRFKSIANYSSPI |
| B438L | B438L CDS | Capsid | 474 | VPRFKSIANYSSPIP |
| B438L | B438L CDS | Capsid | 475 | PRFKSIANYSSPIPN |
| B438L | B438L CDS | Capsid | 476 | RFKSIANYSSPIPNY |
| B438L | B438L CDS | Capsid | 477 | FKSIANYSSPIPNYS |
| B438L | B438L CDS | Capsid | 478 | KSIANYSSPIPNYSK |
| B438L | B438L CDS | Capsid | 479 | SIANYSSPIPNYSKR |
| B438L | B438L CDS | Capsid | 480 | IANYSSPIPNYSKRN |
| B438L | B438L CDS | Capsid | 481 | ANYSSPIPNYSKRNS |
| B438L | B438L CDS | Capsid | 482 | NYSSPIPNYSKRNSG |
| B438L | B438L CDS | Capsid | 483 | YSSPIPNYSKRNSGQ |
| B438L | B438L CDS | Capsid | 484 | SSPIPNYSKRNSGQA |
| B438L | B438L CDS | Capsid | 485 | SPIPNYSKRNSGQAE |
| B438L | B438L CDS | Capsid | 486 | PIPNYSKRNSGQAEY |
| B438L | B438L CDS | Capsid | 487 | IPNYSKRNSGQAEYF |
| B438L | B438L CDS | Capsid | 488 | PNYSKRNSGQAEYFD |
| B438L | B438L CDS | Capsid | 489 | NYSKRNSGQAEYFDT |
| B438L | B438L CDS | Capsid | 490 | YSKRNSGQAEYFDTS |
| B438L | B438L CDS | Capsid | 491 | SKRNSGQAEYFDTSK |
| B438L | B438L CDS | Capsid | 492 | KRNSGQAEYFDTSKQ |
| B438L | B438L CDS | Capsid | 493 | RNSGQAEYFDTSKQT |
| B438L | B438L CDS | Capsid | 494 | NSGQAEYFDTSKQTI |
| B438L | B438L CDS | Capsid | 495 | SGQAEYFDTSKQTIS |
| B438L | B438L CDS | Capsid | 496 | GQAEYFDTSKQTISR |
| B438L | B438L CDS | Capsid | 497 | QAEYFDTSKQTISRH |
| B438L | B438L CDS | Capsid | 498 | AEYFDTSKQTISRHN |
| B438L | B438L CDS | Capsid | 499 | EYFDTSKQTISRHNN |
| B438L | B438L CDS | Capsid | 500 | YFDTSKQTISRHNNY |
| B438L | B438L CDS | Capsid | 501 | FDTSKQTISRHNNYI |
| B438L | B438L CDS | Capsid | 502 | DTSKQTISRHNNYIP |
| B438L | B438L CDS | Capsid | 503 | TSKQTISRHNNYIPK |
| B438L | B438L CDS | Capsid | 504 | SKQTISRHNNYIPKY |
| B438L | B438L CDS | Capsid | 505 | KQTISRHNNYIPKYT |
| B438L | B438L CDS | Capsid | 506 | QTISRHNNYIPKYTG |
| B438L | B438L CDS | Capsid | 507 | TISRHNNYIPKYTGG |
| B438L | B438L CDS | Capsid | 508 | ISRHNNYIPKYTGGI |
| B438L | B438L CDS | Capsid | 509 | SRHNNYIPKYTGGIG |
| B438L | B438L CDS | Capsid | 510 | RHNNYIPKYTGGIGD |
| B438L | B438L CDS | Capsid | 511 | HNNYIPKYTGGIGDS |
| B438L | B438L CDS | Capsid | 512 | NNYIPKYTGGIGDSK |
| B438L | B438L CDS | Capsid | 513 | NYIPKYTGGIGDSKL |
| B438L | B438L CDS | Capsid | 514 | YIPKYTGGIGDSKLD |
| B438L | B438L CDS | Capsid | 515 | IPKYTGGIGDSKLDS |
| B438L | B438L CDS | Capsid | 516 | PKYTGGIGDSKLDST |
| B438L | B438L CDS | Capsid | 517 | KYTGGIGDSKLDSTF |
| B438L | B438L CDS | Capsid | 518 | YTGGIGDSKLDSTFP |
| B438L | B438L CDS | Capsid | 519 | TGGIGDSKLDSTFPK |
| B438L | B438L CDS | Capsid | 520 | GGIGDSKLDSTFPKD |
| B438L | B438L CDS | Capsid | 521 | GIGDSKLDSTFPKDF |
| B438L | B438L CDS | Capsid | 522 | IGDSKLDSTFPKDFN |
| B438L | B438L CDS | Capsid | 523 | GDSKLDSTFPKDFNA |
| B438L | B438L CDS | Capsid | 524 | DSKLDSTFPKDFNAS |

| | | | | |
|---|---|---|---|---|
| B438L | B438L CDS | Capsid | 525 | SKLDSTFPKDFNASS |
| B438L | B438L CDS | Capsid | 526 | KLDSTFPKDFNASSV |
| B438L | B438L CDS | Capsid | 527 | LDSTFPKDFNASSVP |
| B438L | B438L CDS | Capsid | 528 | DSTFPKDFNASSVPL |
| B438L | B438L CDS | Capsid | 529 | STFPKDFNASSVPLT |
| B438L | B438L CDS | Capsid | 530 | TFPKDFNASSVPLTS |
| B438L | B438L CDS | Capsid | 531 | FPKDFNASSVPLTSA |
| B438L | B438L CDS | Capsid | 532 | PKDFNASSVPLTSAE |
| B438L | B438L CDS | Capsid | 533 | KDFNASSVPLTSAEK |
| B438L | B438L CDS | Capsid | 534 | DFNASSVPLTSAEKD |
| B438L | B438L CDS | Capsid | 535 | FNASSVPLTSAEKDH |
| B438L | B438L CDS | Capsid | 536 | NASSVPLTSAEKDHS |
| B438L | B438L CDS | Capsid | 537 | ASSVPLTSAEKDHSL |
| B438L | B438L CDS | Capsid | 538 | SSVPLTSAEKDHSLR |
| B438L | B438L CDS | Capsid | 539 | SVPLTSAEKDHSLRG |
| B438L | B438L CDS | Capsid | 540 | VPLTSAEKDHSLRGD |
| B438L | B438L CDS | Capsid | 541 | PLTSAEKDHSLRGDN |
| B438L | B438L CDS | Capsid | 542 | LTSAEKDHSLRGDNS |
| B438L | B438L CDS | Capsid | 543 | TSAEKDHSLRGDNSA |
| B438L | B438L CDS | Capsid | 544 | SAEKDHSLRGDNSAC |
| B438L | B438L CDS | Capsid | 545 | AEKDHSLRGDNSACC |
| B438L | B438L CDS | Capsid | 546 | EKDHSLRGDNSACCI |
| B438L | B438L CDS | Capsid | 547 | KDHSLRGDNSACCIS |
| B438L | B438L CDS | Capsid | 548 | DHSLRGDNSACCISS |
| B438L | B438L CDS | Capsid | 549 | HSLRGDNSACCISSI |
| B438L | B438L CDS | Capsid | 550 | SLRGDNSACCISSIS |
| B438L | B438L CDS | Capsid | 551 | LRGDNSACCISSISP |
| B438L | B438L CDS | Capsid | 552 | RGDNSACCISSISPS |
| B438L | B438L CDS | Capsid | 553 | GDNSACCISSISPSL |
| B438L | B438L CDS | Capsid | 554 | DNSACCISSISPSLG |
| B438L | B438L CDS | Capsid | 555 | NSACCISSISPSLGS |
| B438L | B438L CDS | Capsid | 556 | SACCISSISPSLGSG |
| B438L | B438L CDS | Capsid | 557 | ACCISSISPSLGSGS |
| B438L | B438L CDS | Capsid | 558 | CCISSISPSLGSGSG |
| B438L | B438L CDS | Capsid | 559 | CISSISPSLGSGSGS |
| B438L | B438L CDS | Capsid | 560 | ISSISPSLGSGSGSG |
| Envelope protein p54 | p54 | Inner envelope | 1 | GSGSGSGMDSEFFQP |
| Envelope protein p54 | p54 | Inner envelope | 2 | SGSGSGMDSEFFQPV |
| Envelope protein p54 | p54 | Inner envelope | 3 | GSGSGMDSEFFQPVY |
| Envelope protein p54 | p54 | Inner envelope | 4 | SGSGMDSEFFQPVYP |
| Envelope protein p54 | p54 | Inner envelope | 5 | GSGMDSEFFQPVYPR |
| Envelope protein p54 | p54 | Inner envelope | 6 | SGMDSEFFQPVYPRH |
| Envelope protein p54 | p54 | Inner envelope | 7 | GMDSEFFQPVYPRHY |
| Envelope protein p54 | p54 | Inner envelope | 8 | MDSEFFQPVYPRHYG |
| Envelope protein p54 | p54 | Inner envelope | 9 | DSEFFQPVYPRHYGE |
| Envelope protein p54 | p54 | Inner envelope | 10 | SEFFQPVYPRHYGEC |
| Envelope protein p54 | p54 | Inner envelope | 11 | EFFQPVYPRHYGECL |
| Envelope protein p54 | p54 | Inner envelope | 12 | FFQPVYPRHYGECLS |
| Envelope protein p54 | p54 | Inner envelope | 13 | FQPVYPRHYGECLSP |
| Envelope protein p54 | p54 | Inner envelope | 14 | QPVYPRHYGECLSPV |
| Envelope protein p54 | p54 | Inner envelope | 15 | PVYPRHYGECLSPVT |
| Envelope protein p54 | p54 | Inner envelope | 16 | VYPRHYGECLSPVTT |
| Envelope protein p54 | p54 | Inner envelope | 17 | YPRHYGECLSPVTTP |
| Envelope protein p54 | p54 | Inner envelope | 18 | PRHYGECLSPVTTPS |
| Envelope protein p54 | p54 | Inner envelope | 19 | RHYGECLSPVTTPSF |
| Envelope protein p54 | p54 | Inner envelope | 20 | HYGECLSPVTTPSFF |
| Envelope protein p54 | p54 | Inner envelope | 21 | YGECLSPVTTPSFFS |
| Envelope protein p54 | p54 | Inner envelope | 22 | GECLSPVTTPSFFST |
| Envelope protein p54 | p54 | Inner envelope | 23 | ECLSPVTTPSFFSTH |
| Envelope protein p54 | p54 | Inner envelope | 24 | CLSPVTTPSFFSTHM |
| Envelope protein p54 | p54 | Inner envelope | 25 | LSPVTTPSFFSTHMY |
| Envelope protein p54 | p54 | Inner envelope | 26 | SPVTTPSFFSTHMYT |
| Envelope protein p54 | p54 | Inner envelope | 27 | PVTTPSFFSTHMYTI |
| Envelope protein p54 | p54 | Inner envelope | 28 | VTTPSFFSTHMYTIL |
| Envelope protein p54 | p54 | Inner envelope | 29 | TTPSFFSTHMYTILI |
| Envelope protein p54 | p54 | Inner envelope | 30 | TPSFFSTHMYTILIA |
| Envelope protein p54 | p54 | Inner envelope | 31 | PSFFSTHMYTILIAI |

| | | | | |
|---|---|---|---|---|
| Envelope protein p54 | p54 | Inner envelope | 32 | SFFSTHMYTILIAIV |
| Envelope protein p54 | p54 | Inner envelope | 33 | FFSTHMYTILIAIVV |
| Envelope protein p54 | p54 | Inner envelope | 34 | FSTHMYTILIAIVVL |
| Envelope protein p54 | p54 | Inner envelope | 35 | STHMYTILIAIVVLV |
| Envelope protein p54 | p54 | Inner envelope | 36 | THMYTILIAIVVLVI |
| Envelope protein p54 | p54 | Inner envelope | 37 | HMYTILIAIVVLVII |
| Envelope protein p54 | p54 | Inner envelope | 38 | MYTILIAIVVLVIII |
| Envelope protein p54 | p54 | Inner envelope | 39 | YTILIAIVVLVIIII |
| Envelope protein p54 | p54 | Inner envelope | 40 | TILIAIVVLVIIIIV |
| Envelope protein p54 | p54 | Inner envelope | 41 | ILIAIVVLVIIIIVL |
| Envelope protein p54 | p54 | Inner envelope | 42 | LIAIVVLVIIIIVLI |
| Envelope protein p54 | p54 | Inner envelope | 43 | IAIVVLVIIIIVLIY |
| Envelope protein p54 | p54 | Inner envelope | 44 | AIVVLVIIIIVLIYL |
| Envelope protein p54 | p54 | Inner envelope | 45 | IVVLVIIIIVLIYLF |
| Envelope protein p54 | p54 | Inner envelope | 46 | VVLVIIIIVLIYLFS |
| Envelope protein p54 | p54 | Inner envelope | 47 | VLVIIIIVLIYLFSS |
| Envelope protein p54 | p54 | Inner envelope | 48 | LVIIIIVLIYLFSSR |
| Envelope protein p54 | p54 | Inner envelope | 49 | VIIIIVLIYLFSSRK |
| Envelope protein p54 | p54 | Inner envelope | 50 | IIIIVLIYLFSSRKK |
| Envelope protein p54 | p54 | Inner envelope | 51 | IIIVLIYLFSSRKKK |
| Envelope protein p54 | p54 | Inner envelope | 52 | IIVLIYLFSSRKKKA |
| Envelope protein p54 | p54 | Inner envelope | 53 | IVLIYLFSSRKKKAA |
| Envelope protein p54 | p54 | Inner envelope | 54 | VLIYLFSSRKKKAAA |
| Envelope protein p54 | p54 | Inner envelope | 55 | LIYLFSSRKKKAAAI |
| Envelope protein p54 | p54 | Inner envelope | 56 | IYLFSSRKKKAAAIE |
| Envelope protein p54 | p54 | Inner envelope | 57 | YLFSSRKKKAAAIEE |
| Envelope protein p54 | p54 | Inner envelope | 58 | LFSSRKKKAAAIEEE |
| Envelope protein p54 | p54 | Inner envelope | 59 | FSSRKKKAAAIEEED |
| Envelope protein p54 | p54 | Inner envelope | 60 | SSRKKKAAAIEEEDI |
| Envelope protein p54 | p54 | Inner envelope | 61 | SRKKKAAAIEEEDIQ |
| Envelope protein p54 | p54 | Inner envelope | 62 | RKKKAAAIEEEDIQF |
| Envelope protein p54 | p54 | Inner envelope | 63 | KKKAAAIEEEDIQFI |
| Envelope protein p54 | p54 | Inner envelope | 64 | KKAAAIEEEDIQFIN |
| Envelope protein p54 | p54 | Inner envelope | 65 | KAAAIEEEDIQFINP |
| Envelope protein p54 | p54 | Inner envelope | 66 | AAAIEEEDIQFINPY |
| Envelope protein p54 | p54 | Inner envelope | 67 | AAIEEEDIQFINPYQ |
| Envelope protein p54 | p54 | Inner envelope | 68 | AIEEEDIQFINPYQD |
| Envelope protein p54 | p54 | Inner envelope | 69 | IEEEDIQFINPYQDQ |
| Envelope protein p54 | p54 | Inner envelope | 70 | EEEDIQFINPYQDQQ |
| Envelope protein p54 | p54 | Inner envelope | 71 | EEDIQFINPYQDQQW |
| Envelope protein p54 | p54 | Inner envelope | 72 | EDIQFINPYQDQQWV |
| Envelope protein p54 | p54 | Inner envelope | 73 | DIQFINPYQDQQWVE |
| Envelope protein p54 | p54 | Inner envelope | 74 | IQFINPYQDQQWVEV |
| Envelope protein p54 | p54 | Inner envelope | 75 | QFINPYQDQQWVEVT |
| Envelope protein p54 | p54 | Inner envelope | 76 | FINPYQDQQWVEVTP |
| Envelope protein p54 | p54 | Inner envelope | 77 | INPYQDQQWVEVTPQ |
| Envelope protein p54 | p54 | Inner envelope | 78 | NPYQDQQWVEVTPQP |
| Envelope protein p54 | p54 | Inner envelope | 79 | PYQDQQWVEVTPQPG |
| Envelope protein p54 | p54 | Inner envelope | 80 | YQDQQWVEVTPQPGT |
| Envelope protein p54 | p54 | Inner envelope | 81 | QDQQWVEVTPQPGTS |
| Envelope protein p54 | p54 | Inner envelope | 82 | DQQWVEVTPQPGTSK |
| Envelope protein p54 | p54 | Inner envelope | 83 | QQWVEVTPQPGTSKP |
| Envelope protein p54 | p54 | Inner envelope | 84 | QWVEVTPQPGTSKPA |
| Envelope protein p54 | p54 | Inner envelope | 85 | WVEVTPQPGTSKPAG |
| Envelope protein p54 | p54 | Inner envelope | 86 | VEVTPQPGTSKPAGA |
| Envelope protein p54 | p54 | Inner envelope | 87 | EVTPQPGTSKPAGAT |
| Envelope protein p54 | p54 | Inner envelope | 88 | VTPQPGTSKPAGATT |
| Envelope protein p54 | p54 | Inner envelope | 89 | TPQPGTSKPAGATTA |
| Envelope protein p54 | p54 | Inner envelope | 90 | PQPGTSKPAGATTAS |
| Envelope protein p54 | p54 | Inner envelope | 91 | QPGTSKPAGATTASV |
| Envelope protein p54 | p54 | Inner envelope | 92 | PGTSKPAGATTASVG |
| Envelope protein p54 | p54 | Inner envelope | 93 | GTSKPAGATTASVGK |
| Envelope protein p54 | p54 | Inner envelope | 94 | TSKPAGATTASVGKP |
| Envelope protein p54 | p54 | Inner envelope | 95 | SKPAGATTASVGKPV |
| Envelope protein p54 | p54 | Inner envelope | 96 | KPAGATTASVGKPVT |
| Envelope protein p54 | p54 | Inner envelope | 97 | PAGATTASVGKPVTG |
| Envelope protein p54 | p54 | Inner envelope | 98 | AGATTASVGKPVTGR |

| | | | | |
|---|---|---|---|---|
| Envelope protein p54 | p54 | Inner envelope | 99 | GATTASVGKPVTGRP |
| Envelope protein p54 | p54 | Inner envelope | 100 | ATTASVGKPVTGRPA |
| Envelope protein p54 | p54 | Inner envelope | 101 | TTASVGKPVTGRPAT |
| Envelope protein p54 | p54 | Inner envelope | 102 | TASVGKPVTGRPATN |
| Envelope protein p54 | p54 | Inner envelope | 103 | ASVGKPVTGRPATNR |
| Envelope protein p54 | p54 | Inner envelope | 104 | SVGKPVTGRPATNRP |
| Envelope protein p54 | p54 | Inner envelope | 105 | VGKPVTGRPATNRPA |
| Envelope protein p54 | p54 | Inner envelope | 106 | GKPVTGRPATNRPAT |
| Envelope protein p54 | p54 | Inner envelope | 107 | KPVTGRPATNRPATN |
| Envelope protein p54 | p54 | Inner envelope | 108 | PVTGRPATNRPATNK |
| Envelope protein p54 | p54 | Inner envelope | 109 | VTGRPATNRPATNKP |
| Envelope protein p54 | p54 | Inner envelope | 110 | TGRPATNRPATNKPV |
| Envelope protein p54 | p54 | Inner envelope | 111 | GRPATNRPATNKPVT |
| Envelope protein p54 | p54 | Inner envelope | 112 | RPATNRPATNKPVTD |
| Envelope protein p54 | p54 | Inner envelope | 113 | PATNRPATNKPVTDN |
| Envelope protein p54 | p54 | Inner envelope | 114 | ATNRPATNKPVTDNP |
| Envelope protein p54 | p54 | Inner envelope | 115 | TNRPATNKPVTDNPV |
| Envelope protein p54 | p54 | Inner envelope | 116 | NRPATNKPVTDNPVT |
| Envelope protein p54 | p54 | Inner envelope | 117 | RPATNKPVTDNPVTD |
| Envelope protein p54 | p54 | Inner envelope | 118 | PATNKPVTDNPVTDR |
| Envelope protein p54 | p54 | Inner envelope | 119 | ATNKPVTDNPVTDRL |
| Envelope protein p54 | p54 | Inner envelope | 120 | TNKPVTDNPVTDRLV |
| Envelope protein p54 | p54 | Inner envelope | 121 | NKPVTDNPVTDRLVM |
| Envelope protein p54 | p54 | Inner envelope | 122 | KPVTDNPVTDRLVMA |
| Envelope protein p54 | p54 | Inner envelope | 123 | PVTDNPVTDRLVMAT |
| Envelope protein p54 | p54 | Inner envelope | 124 | VTDNPVTDRLVMATG |
| Envelope protein p54 | p54 | Inner envelope | 125 | TDNPVTDRLVMATGG |
| Envelope protein p54 | p54 | Inner envelope | 126 | DNPVTDRLVMATGGP |
| Envelope protein p54 | p54 | Inner envelope | 127 | NPVTDRLVMATGGPA |
| Envelope protein p54 | p54 | Inner envelope | 128 | PVTDRLVMATGGPAA |
| Envelope protein p54 | p54 | Inner envelope | 129 | VTDRLVMATGGPAAA |
| Envelope protein p54 | p54 | Inner envelope | 130 | TDRLVMATGGPAAAP |
| Envelope protein p54 | p54 | Inner envelope | 131 | DRLVMATGGPAAAPA |
| Envelope protein p54 | p54 | Inner envelope | 132 | RLVMATGGPAAAPAA |
| Envelope protein p54 | p54 | Inner envelope | 133 | LVMATGGPAAAPAAA |
| Envelope protein p54 | p54 | Inner envelope | 134 | VMATGGPAAAPAAAS |
| Envelope protein p54 | p54 | Inner envelope | 135 | MATGGPAAAPAAASA |
| Envelope protein p54 | p54 | Inner envelope | 136 | ATGGPAAAPAAASAP |
| Envelope protein p54 | p54 | Inner envelope | 137 | TGGPAAAPAAASAPA |
| Envelope protein p54 | p54 | Inner envelope | 138 | GGPAAAPAAASAPAH |
| Envelope protein p54 | p54 | Inner envelope | 139 | GPAAAPAAASAPAHP |
| Envelope protein p54 | p54 | Inner envelope | 140 | PAAAPAAASAPAHPA |
| Envelope protein p54 | p54 | Inner envelope | 141 | AAAPAAASAPAHPAE |
| Envelope protein p54 | p54 | Inner envelope | 142 | AAPAAASAPAHPAEP |
| Envelope protein p54 | p54 | Inner envelope | 143 | APAAASAPAHPAEPY |
| Envelope protein p54 | p54 | Inner envelope | 144 | PAAASAPAHPAEPYT |
| Envelope protein p54 | p54 | Inner envelope | 145 | AAASAPAHPAEPYTT |
| Envelope protein p54 | p54 | Inner envelope | 146 | AASAPAHPAEPYTTV |
| Envelope protein p54 | p54 | Inner envelope | 147 | ASAPAHPAEPYTTVT |
| Envelope protein p54 | p54 | Inner envelope | 148 | SAPAHPAEPYTTVTT |
| Envelope protein p54 | p54 | Inner envelope | 149 | APAHPAEPYTTVTTQ |
| Envelope protein p54 | p54 | Inner envelope | 150 | PAHPAEPYTTVTTQN |
| Envelope protein p54 | p54 | Inner envelope | 151 | AHPAEPYTTVTTQNT |
| Envelope protein p54 | p54 | Inner envelope | 152 | HPAEPYTTVTTQNTA |
| Envelope protein p54 | p54 | Inner envelope | 153 | PAEPYTTVTTQNTAS |
| Envelope protein p54 | p54 | Inner envelope | 154 | AEPYTTVTTQNTASQ |
| Envelope protein p54 | p54 | Inner envelope | 155 | EPYTTVTTQNTASQT |
| Envelope protein p54 | p54 | Inner envelope | 156 | PYTTVTTQNTASQTM |
| Envelope protein p54 | p54 | Inner envelope | 157 | YTTVTTQNTASQTMS |
| Envelope protein p54 | p54 | Inner envelope | 158 | TTVTTQNTASQTMSA |
| Envelope protein p54 | p54 | Inner envelope | 159 | TVTTQNTASQTMSAI |
| Envelope protein p54 | p54 | Inner envelope | 160 | VTTQNTASQTMSAIE |
| Envelope protein p54 | p54 | Inner envelope | 161 | TTQNTASQTMSAIEN |
| Envelope protein p54 | p54 | Inner envelope | 162 | TQNTASQTMSAIENL |
| Envelope protein p54 | p54 | Inner envelope | 163 | QNTASQTMSAIENLR |
| Envelope protein p54 | p54 | Inner envelope | 164 | NTASQTMSAIENLRQ |
| Envelope protein p54 | p54 | Inner envelope | 165 | TASQTMSAIENLRQR |

| | | | | |
|---|---|---|---|---|
| Envelope protein p54 | p54 | Inner envelope | 166 | ASQTMSAIENLRQRN |
| Envelope protein p54 | p54 | Inner envelope | 167 | SQTMSAIENLRQRNT |
| Envelope protein p54 | p54 | Inner envelope | 168 | QTMSAIENLRQRNTY |
| Envelope protein p54 | p54 | Inner envelope | 169 | TMSAIENLRQRNTYT |
| Envelope protein p54 | p54 | Inner envelope | 170 | MSAIENLRQRNTYTH |
| Envelope protein p54 | p54 | Inner envelope | 171 | SAIENLRQRNTYTHK |
| Envelope protein p54 | p54 | Inner envelope | 172 | AIENLRQRNTYTHKD |
| Envelope protein p54 | p54 | Inner envelope | 173 | IENLRQRNTYTHKDL |
| Envelope protein p54 | p54 | Inner envelope | 174 | ENLRQRNTYTHKDLE |
| Envelope protein p54 | p54 | Inner envelope | 175 | NLRQRNTYTHKDLEN |
| Envelope protein p54 | p54 | Inner envelope | 176 | LRQRNTYTHKDLENS |
| Envelope protein p54 | p54 | Inner envelope | 177 | RQRNTYTHKDLENSL |
| Envelope protein p54 | p54 | Inner envelope | 178 | QRNTYTHKDLENSLG |
| Envelope protein p54 | p54 | Inner envelope | 179 | RNTYTHKDLENSLGS |
| Envelope protein p54 | p54 | Inner envelope | 180 | NTYTHKDLENSLGSG |
| Envelope protein p54 | p54 | Inner envelope | 181 | TYTHKDLENSLGSGS |
| Envelope protein p54 | p54 | Inner envelope | 182 | YTHKDLENSLGSGSG |
| Envelope protein p54 | p54 | Inner envelope | 183 | THKDLENSLGSGSGS |
| Envelope protein p54 | p54 | Inner envelope | 184 | HKDLENSLGSGSGSG |
| D117L | D117L CDS | Inner envelope | 1 | GSGSGSGMDTETSPL |
| D117L | D117L CDS | Inner envelope | 2 | SGSGSGMDTETSPLL |
| D117L | D117L CDS | Inner envelope | 3 | GSGSGMDTETSPLLS |
| D117L | D117L CDS | Inner envelope | 4 | SGSGMDTETSPLLSH |
| D117L | D117L CDS | Inner envelope | 5 | GSGMDTETSPLLSHN |
| D117L | D117L CDS | Inner envelope | 6 | SGMDTETSPLLSHNL |
| D117L | D117L CDS | Inner envelope | 7 | GMDTETSPLLSHNLS |
| D117L | D117L CDS | Inner envelope | 8 | MDTETSPLLSHNLST |
| D117L | D117L CDS | Inner envelope | 9 | DTETSPLLSHNLSTR |
| D117L | D117L CDS | Inner envelope | 10 | TETSPLLSHNLSTRE |
| D117L | D117L CDS | Inner envelope | 11 | ETSPLLSHNLSTREG |
| D117L | D117L CDS | Inner envelope | 12 | TSPLLSHNLSTREGI |
| D117L | D117L CDS | Inner envelope | 13 | SPLLSHNLSTREGIK |
| D117L | D117L CDS | Inner envelope | 14 | PLLSHNLSTREGIKQ |
| D117L | D117L CDS | Inner envelope | 15 | LLSHNLSTREGIKQS |
| D117L | D117L CDS | Inner envelope | 16 | LSHNLSTREGIKQST |
| D117L | D117L CDS | Inner envelope | 17 | SHNLSTREGIKQSTQ |
| D117L | D117L CDS | Inner envelope | 18 | HNLSTREGIKQSTQG |
| D117L | D117L CDS | Inner envelope | 19 | NLSTREGIKQSTQGL |
| D117L | D117L CDS | Inner envelope | 20 | LSTREGIKQSTQGLL |
| D117L | D117L CDS | Inner envelope | 21 | STREGIKQSTQGLLA |
| D117L | D117L CDS | Inner envelope | 22 | TREGIKQSTQGLLAH |
| D117L | D117L CDS | Inner envelope | 23 | REGIKQSTQGLLAHT |
| D117L | D117L CDS | Inner envelope | 24 | EGIKQSTQGLLAHTI |
| D117L | D117L CDS | Inner envelope | 25 | GIKQSTQGLLAHTIA |
| D117L | D117L CDS | Inner envelope | 26 | IKQSTQGLLAHTIAK |
| D117L | D117L CDS | Inner envelope | 27 | KQSTQGLLAHTIAKY |
| D117L | D117L CDS | Inner envelope | 28 | QSTQGLLAHTIAKYP |
| D117L | D117L CDS | Inner envelope | 29 | STQGLLAHTIAKYPG |
| D117L | D117L CDS | Inner envelope | 30 | TQGLLAHTIAKYPGT |
| D117L | D117L CDS | Inner envelope | 31 | QGLLAHTIAKYPGTT |
| D117L | D117L CDS | Inner envelope | 32 | GLLAHTIAKYPGTTA |
| D117L | D117L CDS | Inner envelope | 33 | LLAHTIAKYPGTTAI |
| D117L | D117L CDS | Inner envelope | 34 | LAHTIAKYPGTTAIL |
| D117L | D117L CDS | Inner envelope | 35 | AHTIAKYPGTTAILL |
| D117L | D117L CDS | Inner envelope | 36 | HTIAKYPGTTAILLG |
| D117L | D117L CDS | Inner envelope | 37 | TIAKYPGTTAILLGI |
| D117L | D117L CDS | Inner envelope | 38 | IAKYPGTTAILLGIL |
| D117L | D117L CDS | Inner envelope | 39 | AKYPGTTAILLGILI |
| D117L | D117L CDS | Inner envelope | 40 | KYPGTTAILLGILIL |
| D117L | D117L CDS | Inner envelope | 41 | YPGTTAILLGILILL |
| D117L | D117L CDS | Inner envelope | 42 | PGTTAILLGILILLI |
| D117L | D117L CDS | Inner envelope | 43 | GTTAILLGILILLII |
| D117L | D117L CDS | Inner envelope | 44 | TTAILLGILILLIII |
| D117L | D117L CDS | Inner envelope | 45 | TAILLGILILLIIIL |
| D117L | D117L CDS | Inner envelope | 46 | AILLGILILLIIILI |
| D117L | D117L CDS | Inner envelope | 47 | ILLGILILLIIILII |
| D117L | D117L CDS | Inner envelope | 48 | LLGILILLIIILIIV |

| | | | | |
|---|---|---|---|---|
| D117L | D117L CDS | Inner envelope | 49 | LGILILLIIILIIVA |
| D117L | D117L CDS | Inner envelope | 50 | GILILLIIILIIVAI |
| D117L | D117L CDS | Inner envelope | 51 | ILILLIIILIIVAIV |
| D117L | D117L CDS | Inner envelope | 52 | LILLIIILIIVAIVY |
| D117L | D117L CDS | Inner envelope | 53 | ILLIIILIIVAIVYY |
| D117L | D117L CDS | Inner envelope | 54 | LLIIILIIVAIVYYN |
| D117L | D117L CDS | Inner envelope | 55 | LIIILIIVAIVYYNR |
| D117L | D117L CDS | Inner envelope | 56 | IIILIIVAIVYYNRT |
| D117L | D117L CDS | Inner envelope | 57 | IILIIVAIVYYNRTI |
| D117L | D117L CDS | Inner envelope | 58 | ILIIVAIVYYNRTID |
| D117L | D117L CDS | Inner envelope | 59 | LIIVAIVYYNRTIDC |
| D117L | D117L CDS | Inner envelope | 60 | IIVAIVYYNRTIDCK |
| D117L | D117L CDS | Inner envelope | 61 | IVAIVYYNRTIDCKS |
| D117L | D117L CDS | Inner envelope | 62 | VAIVYYNRTIDCKSS |
| D117L | D117L CDS | Inner envelope | 63 | AIVYYNRTIDCKSSI |
| D117L | D117L CDS | Inner envelope | 64 | IVYYNRTIDCKSSIP |
| D117L | D117L CDS | Inner envelope | 65 | VYYNRTIDCKSSIPK |
| D117L | D117L CDS | Inner envelope | 66 | YYNRTIDCKSSIPKP |
| D117L | D117L CDS | Inner envelope | 67 | YNRTIDCKSSIPKPP |
| D117L | D117L CDS | Inner envelope | 68 | NRTIDCKSSIPKPPP |
| D117L | D117L CDS | Inner envelope | 69 | RTIDCKSSIPKPPPS |
| D117L | D117L CDS | Inner envelope | 70 | TIDCKSSIPKPPPSY |
| D117L | D117L CDS | Inner envelope | 71 | IDCKSSIPKPPPSYY |
| D117L | D117L CDS | Inner envelope | 72 | DCKSSIPKPPPSYYV |
| D117L | D117L CDS | Inner envelope | 73 | CKSSIPKPPPSYYVQ |
| D117L | D117L CDS | Inner envelope | 74 | KSSIPKPPPSYYVQQ |
| D117L | D117L CDS | Inner envelope | 75 | SSIPKPPPSYYVQQP |
| D117L | D117L CDS | Inner envelope | 76 | SIPKPPPSYYVQQPE |
| D117L | D117L CDS | Inner envelope | 77 | IPKPPPSYYVQQPEP |
| D117L | D117L CDS | Inner envelope | 78 | PKPPPSYYVQQPEPH |
| D117L | D117L CDS | Inner envelope | 79 | KPPPSYYVQQPEPHH |
| D117L | D117L CDS | Inner envelope | 80 | PPPSYYVQQPEPHHH |
| D117L | D117L CDS | Inner envelope | 81 | PPSYYVQQPEPHHHF |
| D117L | D117L CDS | Inner envelope | 82 | PSYYVQQPEPHHHFP |
| D117L | D117L CDS | Inner envelope | 83 | SYYVQQPEPHHHFPV |
| D117L | D117L CDS | Inner envelope | 84 | YYVQQPEPHHHFPVF |
| D117L | D117L CDS | Inner envelope | 85 | YVQQPEPHHHFPVFF |
| D117L | D117L CDS | Inner envelope | 86 | VQQPEPHHHFPVFFR |
| D117L | D117L CDS | Inner envelope | 87 | QQPEPHHHFPVFFRK |
| D117L | D117L CDS | Inner envelope | 88 | QPEPHHHFPVFFRKR |
| D117L | D117L CDS | Inner envelope | 89 | PEPHHHFPVFFRKRK |
| D117L | D117L CDS | Inner envelope | 90 | EPHHHFPVFFRKRKN |
| D117L | D117L CDS | Inner envelope | 91 | PHHHFPVFFRKRKNS |
| D117L | D117L CDS | Inner envelope | 92 | HHHFPVFFRKRKNST |
| D117L | D117L CDS | Inner envelope | 93 | HHFPVFFRKRKNSTS |
| D117L | D117L CDS | Inner envelope | 94 | HFPVFFRKRKNSTSL |
| D117L | D117L CDS | Inner envelope | 95 | FPVFFRKRKNSTSLQ |
| D117L | D117L CDS | Inner envelope | 96 | PVFFRKRKNSTSLQS |
| D117L | D117L CDS | Inner envelope | 97 | VFFRKRKNSTSLQSH |
| D117L | D117L CDS | Inner envelope | 98 | FFRKRKNSTSLQSHI |
| D117L | D117L CDS | Inner envelope | 99 | FRKRKNSTSLQSHIP |
| D117L | D117L CDS | Inner envelope | 100 | RKRKNSTSLQSHIPS |
| D117L | D117L CDS | Inner envelope | 101 | KRKNSTSLQSHIPSD |
| D117L | D117L CDS | Inner envelope | 102 | RKNSTSLQSHIPSDE |
| D117L | D117L CDS | Inner envelope | 103 | KNSTSLQSHIPSDEQ |
| D117L | D117L CDS | Inner envelope | 104 | NSTSLQSHIPSDEQL |
| D117L | D117L CDS | Inner envelope | 105 | STSLQSHIPSDEQLA |
| D117L | D117L CDS | Inner envelope | 106 | TSLQSHIPSDEQLAE |
| D117L | D117L CDS | Inner envelope | 107 | SLQSHIPSDEQLAEL |
| D117L | D117L CDS | Inner envelope | 108 | LQSHIPSDEQLAELA |
| D117L | D117L CDS | Inner envelope | 109 | QSHIPSDEQLAELAH |
| D117L | D117L CDS | Inner envelope | 110 | SHIPSDEQLAELAHS |
| D117L | D117L CDS | Inner envelope | 111 | HIPSDEQLAELAHSG |
| D117L | D117L CDS | Inner envelope | 112 | IPSDEQLAELAHSGS |
| D117L | D117L CDS | Inner envelope | 113 | PSDEQLAELAHSGSG |
| D117L | D117L CDS | Inner envelope | 114 | SDEQLAELAHSGSGS |
| D117L | D117L CDS | Inner envelope | 115 | DEQLAELAHSGSGSG |

| | | | | |
|---|---|---|---|---|
| D117L | D117L CDS | Inner envelope | 116 | EQLAELAHSGSGSGS |
| D117L | D117L CDS | Inner envelope | 117 | QLAELAHSGSGSGSG |
| Protein E248R | E248R CDS | Inner envelope | 1 | GSGSGSGMGGSTSKN |
| Protein E248R | E248R CDS | Inner envelope | 2 | SGSGSGMGGSTSKNS |
| Protein E248R | E248R CDS | Inner envelope | 3 | GSGSGMGGSTSKNSF |
| Protein E248R | E248R CDS | Inner envelope | 4 | SGSGMGGSTSKNSFK |
| Protein E248R | E248R CDS | Inner envelope | 5 | GSGMGGSTSKNSFKN |
| Protein E248R | E248R CDS | Inner envelope | 6 | SGMGGSTSKNSFKNT |
| Protein E248R | E248R CDS | Inner envelope | 7 | GMGGSTSKNSFKNTT |
| Protein E248R | E248R CDS | Inner envelope | 8 | MGGSTSKNSFKNTTN |
| Protein E248R | E248R CDS | Inner envelope | 9 | GGSTSKNSFKNTTNI |
| Protein E248R | E248R CDS | Inner envelope | 10 | GSTSKNSFKNTTNII |
| Protein E248R | E248R CDS | Inner envelope | 11 | STSKNSFKNTTNIIS |
| Protein E248R | E248R CDS | Inner envelope | 12 | TSKNSFKNTTNIISN |
| Protein E248R | E248R CDS | Inner envelope | 13 | SKNSFKNTTNIISNS |
| Protein E248R | E248R CDS | Inner envelope | 14 | KNSFKNTTNIISNSI |
| Protein E248R | E248R CDS | Inner envelope | 15 | NSFKNTTNIISNSIF |
| Protein E248R | E248R CDS | Inner envelope | 16 | SFKNTTNIISNSIFN |
| Protein E248R | E248R CDS | Inner envelope | 17 | FKNTTNIISNSIFNQ |
| Protein E248R | E248R CDS | Inner envelope | 18 | KNTTNIISNSIFNQM |
| Protein E248R | E248R CDS | Inner envelope | 19 | NTTNIISNSIFNQMQ |
| Protein E248R | E248R CDS | Inner envelope | 20 | TTNIISNSIFNQMQS |
| Protein E248R | E248R CDS | Inner envelope | 21 | TNIISNSIFNQMQSC |
| Protein E248R | E248R CDS | Inner envelope | 22 | NIISNSIFNQMQSCI |
| Protein E248R | E248R CDS | Inner envelope | 23 | IISNSIFNQMQSCIS |
| Protein E248R | E248R CDS | Inner envelope | 24 | ISNSIFNQMQSCISM |
| Protein E248R | E248R CDS | Inner envelope | 25 | SNSIFNQMQSCISML |
| Protein E248R | E248R CDS | Inner envelope | 26 | NSIFNQMQSCISMLD |
| Protein E248R | E248R CDS | Inner envelope | 27 | SIFNQMQSCISMLDG |
| Protein E248R | E248R CDS | Inner envelope | 28 | IFNQMQSCISMLDGK |
| Protein E248R | E248R CDS | Inner envelope | 29 | FNQMQSCISMLDGKN |
| Protein E248R | E248R CDS | Inner envelope | 30 | NQMQSCISMLDGKNY |
| Protein E248R | E248R CDS | Inner envelope | 31 | QMQSCISMLDGKNYI |
| Protein E248R | E248R CDS | Inner envelope | 32 | MQSCISMLDGKNYIG |
| Protein E248R | E248R CDS | Inner envelope | 33 | QSCISMLDGKNYIGV |
| Protein E248R | E248R CDS | Inner envelope | 34 | SCISMLDGKNYIGVF |
| Protein E248R | E248R CDS | Inner envelope | 35 | CISMLDGKNYIGVFG |
| Protein E248R | E248R CDS | Inner envelope | 36 | ISMLDGKNYIGVFGD |
| Protein E248R | E248R CDS | Inner envelope | 37 | SMLDGKNYIGVFGDG |
| Protein E248R | E248R CDS | Inner envelope | 38 | MLDGKNYIGVFGDGN |
| Protein E248R | E248R CDS | Inner envelope | 39 | LDGKNYIGVFGDGNI |
| Protein E248R | E248R CDS | Inner envelope | 40 | DGKNYIGVFGDGNIL |
| Protein E248R | E248R CDS | Inner envelope | 41 | GKNYIGVFGDGNILN |
| Protein E248R | E248R CDS | Inner envelope | 42 | KNYIGVFGDGNILNH |
| Protein E248R | E248R CDS | Inner envelope | 43 | NYIGVFGDGNILNHV |
| Protein E248R | E248R CDS | Inner envelope | 44 | YIGVFGDGNILNHVF |
| Protein E248R | E248R CDS | Inner envelope | 45 | IGVFGDGNILNHVFQ |
| Protein E248R | E248R CDS | Inner envelope | 46 | GVFGDGNILNHVFQD |
| Protein E248R | E248R CDS | Inner envelope | 47 | VFGDGNILNHVFQDL |
| Protein E248R | E248R CDS | Inner envelope | 48 | FGDGNILNHVFQDLN |
| Protein E248R | E248R CDS | Inner envelope | 49 | GDGNILNHVFQDLNL |
| Protein E248R | E248R CDS | Inner envelope | 50 | DGNILNHVFQDLNLS |
| Protein E248R | E248R CDS | Inner envelope | 51 | GNILNHVFQDLNLSL |
| Protein E248R | E248R CDS | Inner envelope | 52 | NILNHVFQDLNLSLN |
| Protein E248R | E248R CDS | Inner envelope | 53 | ILNHVFQDLNLSLNT |
| Protein E248R | E248R CDS | Inner envelope | 54 | LNHVFQDLNLSLNTS |
| Protein E248R | E248R CDS | Inner envelope | 55 | NHVFQDLNLSLNTSC |
| Protein E248R | E248R CDS | Inner envelope | 56 | HVFQDLNLSLNTSCV |
| Protein E248R | E248R CDS | Inner envelope | 57 | VFQDLNLSLNTSCVQ |
| Protein E248R | E248R CDS | Inner envelope | 58 | FQDLNLSLNTSCVQK |
| Protein E248R | E248R CDS | Inner envelope | 59 | QDLNLSLNTSCVQKH |
| Protein E248R | E248R CDS | Inner envelope | 60 | DLNLSLNTSCVQKHV |
| Protein E248R | E248R CDS | Inner envelope | 61 | LNLSLNTSCVQKHVN |
| Protein E248R | E248R CDS | Inner envelope | 62 | NLSLNTSCVQKHVNE |
| Protein E248R | E248R CDS | Inner envelope | 63 | LSLNTSCVQKHVNEE |
| Protein E248R | E248R CDS | Inner envelope | 64 | SLNTSCVQKHVNEEN |
| Protein E248R | E248R CDS | Inner envelope | 65 | LNTSCVQKHVNEENF |

| | | | | |
|---|---|---|---|---|
| Protein E248R | E248R CDS | Inner envelope | 66 | NTSCVQKHVNEENFI |
| Protein E248R | E248R CDS | Inner envelope | 67 | TSCVQKHVNEENFIT |
| Protein E248R | E248R CDS | Inner envelope | 68 | SCVQKHVNEENFITN |
| Protein E248R | E248R CDS | Inner envelope | 69 | CVQKHVNEENFITNL |
| Protein E248R | E248R CDS | Inner envelope | 70 | VQKHVNEENFITNLS |
| Protein E248R | E248R CDS | Inner envelope | 71 | QKHVNEENFITNLSN |
| Protein E248R | E248R CDS | Inner envelope | 72 | KHVNEENFITNLSNQ |
| Protein E248R | E248R CDS | Inner envelope | 73 | HVNEENFITNLSNQI |
| Protein E248R | E248R CDS | Inner envelope | 74 | VNEENFITNLSNQIT |
| Protein E248R | E248R CDS | Inner envelope | 75 | NEENFITNLSNQITQ |
| Protein E248R | E248R CDS | Inner envelope | 76 | EENFITNLSNQITQN |
| Protein E248R | E248R CDS | Inner envelope | 77 | ENFITNLSNQITQNL |
| Protein E248R | E248R CDS | Inner envelope | 78 | NFITNLSNQITQNLK |
| Protein E248R | E248R CDS | Inner envelope | 79 | FITNLSNQITQNLKD |
| Protein E248R | E248R CDS | Inner envelope | 80 | ITNLSNQITQNLKDQ |
| Protein E248R | E248R CDS | Inner envelope | 81 | TNLSNQITQNLKDQE |
| Protein E248R | E248R CDS | Inner envelope | 82 | NLSNQITQNLKDQEV |
| Protein E248R | E248R CDS | Inner envelope | 83 | LSNQITQNLKDQEVA |
| Protein E248R | E248R CDS | Inner envelope | 84 | SNQITQNLKDQEVAL |
| Protein E248R | E248R CDS | Inner envelope | 85 | NQITQNLKDQEVALT |
| Protein E248R | E248R CDS | Inner envelope | 86 | QITQNLKDQEVALTQ |
| Protein E248R | E248R CDS | Inner envelope | 87 | ITQNLKDQEVALTQW |
| Protein E248R | E248R CDS | Inner envelope | 88 | TQNLKDQEVALTQWM |
| Protein E248R | E248R CDS | Inner envelope | 89 | QNLKDQEVALTQWMD |
| Protein E248R | E248R CDS | Inner envelope | 90 | NLKDQEVALTQWMDA |
| Protein E248R | E248R CDS | Inner envelope | 91 | LKDQEVALTQWMDAG |
| Protein E248R | E248R CDS | Inner envelope | 92 | KDQEVALTQWMDAGT |
| Protein E248R | E248R CDS | Inner envelope | 93 | DQEVALTQWMDAGTH |
| Protein E248R | E248R CDS | Inner envelope | 94 | QEVALTQWMDAGTHD |
| Protein E248R | E248R CDS | Inner envelope | 95 | EVALTQWMDAGTHDQ |
| Protein E248R | E248R CDS | Inner envelope | 96 | VALTQWMDAGTHDQK |
| Protein E248R | E248R CDS | Inner envelope | 97 | ALTQWMDAGTHDQKT |
| Protein E248R | E248R CDS | Inner envelope | 98 | LTQWMDAGTHDQKTD |
| Protein E248R | E248R CDS | Inner envelope | 99 | TQWMDAGTHDQKTDI |
| Protein E248R | E248R CDS | Inner envelope | 100 | QWMDAGTHDQKTDIE |
| Protein E248R | E248R CDS | Inner envelope | 101 | WMDAGTHDQKTDIEE |
| Protein E248R | E248R CDS | Inner envelope | 102 | MDAGTHDQKTDIEEN |
| Protein E248R | E248R CDS | Inner envelope | 103 | DAGTHDQKTDIEENI |
| Protein E248R | E248R CDS | Inner envelope | 104 | AGTHDQKTDIEENIK |
| Protein E248R | E248R CDS | Inner envelope | 105 | GTHDQKTDIEENIKV |
| Protein E248R | E248R CDS | Inner envelope | 106 | THDQKTDIEENIKVN |
| Protein E248R | E248R CDS | Inner envelope | 107 | HDQKTDIEENIKVNL |
| Protein E248R | E248R CDS | Inner envelope | 108 | DQKTDIEENIKVNLT |
| Protein E248R | E248R CDS | Inner envelope | 109 | QKTDIEENIKVNLTT |
| Protein E248R | E248R CDS | Inner envelope | 110 | KTDIEENIKVNLTTT |
| Protein E248R | E248R CDS | Inner envelope | 111 | TDIEENIKVNLTTTL |
| Protein E248R | E248R CDS | Inner envelope | 112 | DIEENIKVNLTTTLI |
| Protein E248R | E248R CDS | Inner envelope | 113 | IEENIKVNLTTTLIQ |
| Protein E248R | E248R CDS | Inner envelope | 114 | EENIKVNLTTTLIQN |
| Protein E248R | E248R CDS | Inner envelope | 115 | ENIKVNLTTTLIQNC |
| Protein E248R | E248R CDS | Inner envelope | 116 | NIKVNLTTTLIQNCV |
| Protein E248R | E248R CDS | Inner envelope | 117 | IKVNLTTTLIQNCVS |
| Protein E248R | E248R CDS | Inner envelope | 118 | KVNLTTTLIQNCVSS |
| Protein E248R | E248R CDS | Inner envelope | 119 | VNLTTTLIQNCVSSL |
| Protein E248R | E248R CDS | Inner envelope | 120 | NLTTTLIQNCVSSLS |
| Protein E248R | E248R CDS | Inner envelope | 121 | LTTTLIQNCVSSLSG |
| Protein E248R | E248R CDS | Inner envelope | 122 | TTTLIQNCVSSLSGM |
| Protein E248R | E248R CDS | Inner envelope | 123 | TTLIQNCVSSLSGMN |
| Protein E248R | E248R CDS | Inner envelope | 124 | TLIQNCVSSLSGMNV |
| Protein E248R | E248R CDS | Inner envelope | 125 | LIQNCVSSLSGMNVL |
| Protein E248R | E248R CDS | Inner envelope | 126 | IQNCVSSLSGMNVLV |
| Protein E248R | E248R CDS | Inner envelope | 127 | QNCVSSLSGMNVLVV |
| Protein E248R | E248R CDS | Inner envelope | 128 | NCVSSLSGMNVLVVK |
| Protein E248R | E248R CDS | Inner envelope | 129 | CVSSLSGMNVLVVKG |
| Protein E248R | E248R CDS | Inner envelope | 130 | VSSLSGMNVLVVKGN |
| Protein E248R | E248R CDS | Inner envelope | 131 | SSLSGMNVLVVKGNG |
| Protein E248R | E248R CDS | Inner envelope | 132 | SLSGMNVLVVKGNGN |

| | | | |
|---|---|---|---|
| Protein E248R | E248R CDS | Inner envelope | 133 LSGMNVLVVKGNGNI |
| Protein E248R | E248R CDS | Inner envelope | 134 SGMNVLVVKGNGNIV |
| Protein E248R | E248R CDS | Inner envelope | 135 GMNVLVVKGNGNIVE |
| Protein E248R | E248R CDS | Inner envelope | 136 MNVLVVKGNGNIVEN |
| Protein E248R | E248R CDS | Inner envelope | 137 NVLVVKGNGNIVENA |
| Protein E248R | E248R CDS | Inner envelope | 138 VLVVKGNGNIVENAT |
| Protein E248R | E248R CDS | Inner envelope | 139 LVVKGNGNIVENATQ |
| Protein E248R | E248R CDS | Inner envelope | 140 VVKGNGNIVENATQK |
| Protein E248R | E248R CDS | Inner envelope | 141 VKGNGNIVENATQKQ |
| Protein E248R | E248R CDS | Inner envelope | 142 KGNGNIVENATQKQS |
| Protein E248R | E248R CDS | Inner envelope | 143 GNGNIVENATQKQSQ |
| Protein E248R | E248R CDS | Inner envelope | 144 NGNIVENATQKQSQQ |
| Protein E248R | E248R CDS | Inner envelope | 145 GNIVENATQKQSQQI |
| Protein E248R | E248R CDS | Inner envelope | 146 NIVENATQKQSQQII |
| Protein E248R | E248R CDS | Inner envelope | 147 IVENATQKQSQQIIS |
| Protein E248R | E248R CDS | Inner envelope | 148 VENATQKQSQQIISN |
| Protein E248R | E248R CDS | Inner envelope | 149 ENATQKQSQQIISNC |
| Protein E248R | E248R CDS | Inner envelope | 150 NATQKQSQQIISNCL |
| Protein E248R | E248R CDS | Inner envelope | 151 ATQKQSQQIISNCLQ |
| Protein E248R | E248R CDS | Inner envelope | 152 TQKQSQQIISNCLQG |
| Protein E248R | E248R CDS | Inner envelope | 153 QKQSQQIISNCLQGS |
| Protein E248R | E248R CDS | Inner envelope | 154 KQSQQIISNCLQGSK |
| Protein E248R | E248R CDS | Inner envelope | 155 QSQQIISNCLQGSKQ |
| Protein E248R | E248R CDS | Inner envelope | 156 SQQIISNCLQGSKQA |
| Protein E248R | E248R CDS | Inner envelope | 157 QQIISNCLQGSKQAI |
| Protein E248R | E248R CDS | Inner envelope | 158 QIISNCLQGSKQAID |
| Protein E248R | E248R CDS | Inner envelope | 159 IISNCLQGSKQAIDT |
| Protein E248R | E248R CDS | Inner envelope | 160 ISNCLQGSKQAIDTT |
| Protein E248R | E248R CDS | Inner envelope | 161 SNCLQGSKQAIDTTT |
| Protein E248R | E248R CDS | Inner envelope | 162 NCLQGSKQAIDTTTG |
| Protein E248R | E248R CDS | Inner envelope | 163 CLQGSKQAIDTTTGI |
| Protein E248R | E248R CDS | Inner envelope | 164 LQGSKQAIDTTTGIT |
| Protein E248R | E248R CDS | Inner envelope | 165 QGSKQAIDTTTGITN |
| Protein E248R | E248R CDS | Inner envelope | 166 GSKQAIDTTTGITNT |
| Protein E248R | E248R CDS | Inner envelope | 167 SKQAIDTTTGITNTV |
| Protein E248R | E248R CDS | Inner envelope | 168 KQAIDTTTGITNTVN |
| Protein E248R | E248R CDS | Inner envelope | 169 QAIDTTTGITNTVNQ |
| Protein E248R | E248R CDS | Inner envelope | 170 AIDTTTGITNTVNQY |
| Protein E248R | E248R CDS | Inner envelope | 171 IDTTTGITNTVNQYS |
| Protein E248R | E248R CDS | Inner envelope | 172 DTTTGITNTVNQYSH |
| Protein E248R | E248R CDS | Inner envelope | 173 TTTGITNTVNQYSHY |
| Protein E248R | E248R CDS | Inner envelope | 174 TTGITNTVNQYSHYT |
| Protein E248R | E248R CDS | Inner envelope | 175 TGITNTVNQYSHYTS |
| Protein E248R | E248R CDS | Inner envelope | 176 GITNTVNQYSHYTSK |
| Protein E248R | E248R CDS | Inner envelope | 177 ITNTVNQYSHYTSKN |
| Protein E248R | E248R CDS | Inner envelope | 178 TNTVNQYSHYTSKNF |
| Protein E248R | E248R CDS | Inner envelope | 179 NTVNQYSHYTSKNFF |
| Protein E248R | E248R CDS | Inner envelope | 180 TVNQYSHYTSKNFFD |
| Protein E248R | E248R CDS | Inner envelope | 181 VNQYSHYTSKNFFDF |
| Protein E248R | E248R CDS | Inner envelope | 182 NQYSHYTSKNFFDFI |
| Protein E248R | E248R CDS | Inner envelope | 183 QYSHYTSKNFFDFIA |
| Protein E248R | E248R CDS | Inner envelope | 184 YSHYTSKNFFDFIAD |
| Protein E248R | E248R CDS | Inner envelope | 185 SHYTSKNFFDFIADA |
| Protein E248R | E248R CDS | Inner envelope | 186 HYTSKNFFDFIADAI |
| Protein E248R | E248R CDS | Inner envelope | 187 YTSKNFFDFIADAIS |
| Protein E248R | E248R CDS | Inner envelope | 188 TSKNFFDFIADAISA |
| Protein E248R | E248R CDS | Inner envelope | 189 SKNFFDFIADAISAV |
| Protein E248R | E248R CDS | Inner envelope | 190 KNFFDFIADAISAVF |
| Protein E248R | E248R CDS | Inner envelope | 191 NFFDFIADAISAVFK |
| Protein E248R | E248R CDS | Inner envelope | 192 FFDFIADAISAVFKN |
| Protein E248R | E248R CDS | Inner envelope | 193 FDFIADAISAVFKNI |
| Protein E248R | E248R CDS | Inner envelope | 194 DFIADAISAVFKNIM |
| Protein E248R | E248R CDS | Inner envelope | 195 FIADAISAVFKNIMV |
| Protein E248R | E248R CDS | Inner envelope | 196 IADAISAVFKNIMVA |
| Protein E248R | E248R CDS | Inner envelope | 197 ADAISAVFKNIMVAA |
| Protein E248R | E248R CDS | Inner envelope | 198 DAISAVFKNIMVAAV |
| Protein E248R | E248R CDS | Inner envelope | 199 AISAVFKNIMVAAVV |

| | | | | |
|---|---|---|---|---|
| Protein E248R | E248R CDS | Inner envelope | 200 | ISAVFKNIMVAAVVI |
| Protein E248R | E248R CDS | Inner envelope | 201 | SAVFKNIMVAAVVIV |
| Protein E248R | E248R CDS | Inner envelope | 202 | AVFKNIMVAAVVIVL |
| Protein E248R | E248R CDS | Inner envelope | 203 | VFKNIMVAAVVIVLI |
| Protein E248R | E248R CDS | Inner envelope | 204 | FKNIMVAAVVIVLII |
| Protein E248R | E248R CDS | Inner envelope | 205 | KNIMVAAVVIVLIIV |
| Protein E248R | E248R CDS | Inner envelope | 206 | NIMVAAVVIVLIIVG |
| Protein E248R | E248R CDS | Inner envelope | 207 | IMVAAVVIVLIIVGF |
| Protein E248R | E248R CDS | Inner envelope | 208 | MVAAVVIVLIIVGFI |
| Protein E248R | E248R CDS | Inner envelope | 209 | VAAVVIVLIIVGFIA |
| Protein E248R | E248R CDS | Inner envelope | 210 | AAVVIVLIIVGFIAV |
| Protein E248R | E248R CDS | Inner envelope | 211 | AVVIVLIIVGFIAVF |
| Protein E248R | E248R CDS | Inner envelope | 212 | VVIVLIIVGFIAVFY |
| Protein E248R | E248R CDS | Inner envelope | 213 | VIVLIIVGFIAVFYF |
| Protein E248R | E248R CDS | Inner envelope | 214 | IVLIIVGFIAVFYFL |
| Protein E248R | E248R CDS | Inner envelope | 215 | VLIIVGFIAVFYFLH |
| Protein E248R | E248R CDS | Inner envelope | 216 | LIIVGFIAVFYFLHS |
| Protein E248R | E248R CDS | Inner envelope | 217 | IIVGFIAVFYFLHSR |
| Protein E248R | E248R CDS | Inner envelope | 218 | IVGFIAVFYFLHSRH |
| Protein E248R | E248R CDS | Inner envelope | 219 | VGFIAVFYFLHSRHR |
| Protein E248R | E248R CDS | Inner envelope | 220 | GFIAVFYFLHSRHRH |
| Protein E248R | E248R CDS | Inner envelope | 221 | FIAVFYFLHSRHRHE |
| Protein E248R | E248R CDS | Inner envelope | 222 | IAVFYFLHSRHRHEE |
| Protein E248R | E248R CDS | Inner envelope | 223 | AVFYFLHSRHRHEEE |
| Protein E248R | E248R CDS | Inner envelope | 224 | VFYFLHSRHRHEEEE |
| Protein E248R | E248R CDS | Inner envelope | 225 | FYFLHSRHRHEEEEE |
| Protein E248R | E248R CDS | Inner envelope | 226 | YFLHSRHRHEEEEEA |
| Protein E248R | E248R CDS | Inner envelope | 227 | FLHSRHRHEEEEEAE |
| Protein E248R | E248R CDS | Inner envelope | 228 | LHSRHRHEEEEEAEP |
| Protein E248R | E248R CDS | Inner envelope | 229 | HSRHRHEEEEEAEPL |
| Protein E248R | E248R CDS | Inner envelope | 230 | SRHRHEEEEEAEPLI |
| Protein E248R | E248R CDS | Inner envelope | 231 | RHRHEEEEEAEPLIS |
| Protein E248R | E248R CDS | Inner envelope | 232 | HRHEEEEEAEPLISN |
| Protein E248R | E248R CDS | Inner envelope | 233 | RHEEEEEAEPLISNK |
| Protein E248R | E248R CDS | Inner envelope | 234 | HEEEEEAEPLISNKV |
| Protein E248R | E248R CDS | Inner envelope | 235 | EEEEEAEPLISNKVL |
| Protein E248R | E248R CDS | Inner envelope | 236 | EEEEAEPLISNKVLK |
| Protein E248R | E248R CDS | Inner envelope | 237 | EEEAEPLISNKVLKN |
| Protein E248R | E248R CDS | Inner envelope | 238 | EEAEPLISNKVLKNA |
| Protein E248R | E248R CDS | Inner envelope | 239 | EAEPLISNKVLKNAA |
| Protein E248R | E248R CDS | Inner envelope | 240 | AEPLISNKVLKNAAV |
| Protein E248R | E248R CDS | Inner envelope | 241 | EPLISNKVLKNAAVS |
| Protein E248R | E248R CDS | Inner envelope | 242 | PLISNKVLKNAAVSG |
| Protein E248R | E248R CDS | Inner envelope | 243 | LISNKVLKNAAVSGS |
| Protein E248R | E248R CDS | Inner envelope | 244 | ISNKVLKNAAVSGSG |
| Protein E248R | E248R CDS | Inner envelope | 245 | SNKVLKNAAVSGSGS |
| Protein E248R | E248R CDS | Inner envelope | 246 | NKVLKNAAVSGSGSG |
| Protein E248R | E248R CDS | Inner envelope | 247 | KVLKNAAVSGSGSGS |
| Protein E248R | E248R CDS | Inner envelope | 248 | VLKNAAVSGSGSGSG |
| BA71V-CP2475L (P220) | BA71V-CP2475L (p220) | Core shell | 1 | GSGSGSGMGNRGSST |
| BA71V-CP2475L (P220) | BA71V-CP2475L (p220) | Core shell | 2 | SGSGSGMGNRGSSTS |
| BA71V-CP2475L (P220) | BA71V-CP2475L (p220) | Core shell | 3 | GSGSGMGNRGSSTSS |
| BA71V-CP2475L (P220) | BA71V-CP2475L (p220) | Core shell | 4 | SGSGMGNRGSSTSSR |
| BA71V-CP2475L (P220) | BA71V-CP2475L (p220) | Core shell | 5 | GSGMGNRGSSTSSRP |
| BA71V-CP2475L (P220) | BA71V-CP2475L (p220) | Core shell | 6 | SGMGNRGSSTSSRPP |
| BA71V-CP2475L (P220) | BA71V-CP2475L (p220) | Core shell | 7 | GMGNRGSSTSSRPPL |
| BA71V-CP2475L (P220) | BA71V-CP2475L (p220) | Core shell | 8 | MGNRGSSTSSRPPLS |
| BA71V-CP2475L (P220) | BA71V-CP2475L (p220) | Core shell | 9 | GNRGSSTSSRPPLSS |
| BA71V-CP2475L (P220) | BA71V-CP2475L (p220) | Core shell | 10 | NRGSSTSSRPPLSSE |
| BA71V-CP2475L (P220) | BA71V-CP2475L (p220) | Core shell | 11 | RGSSTSSRPPLSSEA |
| BA71V-CP2475L (P220) | BA71V-CP2475L (p220) | Core shell | 12 | GSSTSSRPPLSSEAN |
| BA71V-CP2475L (P220) | BA71V-CP2475L (p220) | Core shell | 13 | SSTSSRPPLSSEANL |
| BA71V-CP2475L (P220) | BA71V-CP2475L (p220) | Core shell | 14 | STSSRPPLSSEANLY |
| BA71V-CP2475L (P220) | BA71V-CP2475L (p220) | Core shell | 15 | TSSRPPLSSEANLYA |
| BA71V-CP2475L (P220) | BA71V-CP2475L (p220) | Core shell | 16 | SSRPPLSSEANLYAK |
| BA71V-CP2475L (P220) | BA71V-CP2475L (p220) | Core shell | 17 | SRPPLSSEANLYAKL |
| BA71V-CP2475L (P220) | BA71V-CP2475L (p220) | Core shell | 18 | RPPLSSEANLYAKLQ |

| | | | |
|---|---|---|---|
| BA71V-CP2475L (P220) | BA71V-CP2475L (p220) | Core shell | 19 PPLSSEANLYAKLQD |
| BA71V-CP2475L (P220) | BA71V-CP2475L (p220) | Core shell | 20 PLSSEANLYAKLQDH |
| BA71V-CP2475L (P220) | BA71V-CP2475L (p220) | Core shell | 21 LSSEANLYAKLQDHI |
| BA71V-CP2475L (P220) | BA71V-CP2475L (p220) | Core shell | 22 SSEANLYAKLQDHIQ |
| BA71V-CP2475L (P220) | BA71V-CP2475L (p220) | Core shell | 23 SEANLYAKLQDHIQR |
| BA71V-CP2475L (P220) | BA71V-CP2475L (p220) | Core shell | 24 EANLYAKLQDHIQRQ |
| BA71V-CP2475L (P220) | BA71V-CP2475L (p220) | Core shell | 25 ANLYAKLQDHIQRQT |
| BA71V-CP2475L (P220) | BA71V-CP2475L (p220) | Core shell | 26 NLYAKLQDHIQRQTR |
| BA71V-CP2475L (P220) | BA71V-CP2475L (p220) | Core shell | 27 LYAKLQDHIQRQTRP |
| BA71V-CP2475L (P220) | BA71V-CP2475L (p220) | Core shell | 28 YAKLQDHIQRQTRPF |
| BA71V-CP2475L (P220) | BA71V-CP2475L (p220) | Core shell | 29 AKLQDHIQRQTRPFA |
| BA71V-CP2475L (P220) | BA71V-CP2475L (p220) | Core shell | 30 KLQDHIQRQTRPFAG |
| BA71V-CP2475L (P220) | BA71V-CP2475L (p220) | Core shell | 31 LQDHIQRQTRPFAGG |
| BA71V-CP2475L (P220) | BA71V-CP2475L (p220) | Core shell | 32 QDHIQRQTRPFAGGG |
| BA71V-CP2475L (P220) | BA71V-CP2475L (p220) | Core shell | 33 DHIQRQTRPFAGGGY |
| BA71V-CP2475L (P220) | BA71V-CP2475L (p220) | Core shell | 34 HIQRQTRPFAGGGYF |
| BA71V-CP2475L (P220) | BA71V-CP2475L (p220) | Core shell | 35 IQRQTRPFAGGGYFN |
| BA71V-CP2475L (P220) | BA71V-CP2475L (p220) | Core shell | 36 QRQTRPFAGGGYFNG |
| BA71V-CP2475L (P220) | BA71V-CP2475L (p220) | Core shell | 37 RQTRPFAGGGYFNGG |
| BA71V-CP2475L (P220) | BA71V-CP2475L (p220) | Core shell | 38 QTRPFAGGGYFNGGG |
| BA71V-CP2475L (P220) | BA71V-CP2475L (p220) | Core shell | 39 TRPFAGGGYFNGGGD |
| BA71V-CP2475L (P220) | BA71V-CP2475L (p220) | Core shell | 40 RPFAGGGYFNGGGDK |
| BA71V-CP2475L (P220) | BA71V-CP2475L (p220) | Core shell | 41 PFAGGGYFNGGGDKN |
| BA71V-CP2475L (P220) | BA71V-CP2475L (p220) | Core shell | 42 FAGGGYFNGGGDKNP |
| BA71V-CP2475L (P220) | BA71V-CP2475L (p220) | Core shell | 43 AGGGYFNGGGDKNPV |
| BA71V-CP2475L (P220) | BA71V-CP2475L (p220) | Core shell | 44 GGGYFNGGGDKNPVQ |
| BA71V-CP2475L (P220) | BA71V-CP2475L (p220) | Core shell | 45 GGYFNGGGDKNPVQH |
| BA71V-CP2475L (P220) | BA71V-CP2475L (p220) | Core shell | 46 GYFNGGGDKNPVQHI |
| BA71V-CP2475L (P220) | BA71V-CP2475L (p220) | Core shell | 47 YFNGGGDKNPVQHIK |
| BA71V-CP2475L (P220) | BA71V-CP2475L (p220) | Core shell | 48 FNGGGDKNPVQHIKD |
| BA71V-CP2475L (P220) | BA71V-CP2475L (p220) | Core shell | 49 NGGGDKNPVQHIKDY |
| BA71V-CP2475L (P220) | BA71V-CP2475L (p220) | Core shell | 50 GGGDKNPVQHIKDYH |
| BA71V-CP2475L (P220) | BA71V-CP2475L (p220) | Core shell | 51 GGDKNPVQHIKDYHI |
| BA71V-CP2475L (P220) | BA71V-CP2475L (p220) | Core shell | 52 GDKNPVQHIKDYHID |
| BA71V-CP2475L (P220) | BA71V-CP2475L (p220) | Core shell | 53 DKNPVQHIKDYHIDS |
| BA71V-CP2475L (P220) | BA71V-CP2475L (p220) | Core shell | 54 KNPVQHIKDYHIDSV |
| BA71V-CP2475L (P220) | BA71V-CP2475L (p220) | Core shell | 55 NPVQHIKDYHIDSVS |
| BA71V-CP2475L (P220) | BA71V-CP2475L (p220) | Core shell | 56 PVQHIKDYHIDSVSS |
| BA71V-CP2475L (P220) | BA71V-CP2475L (p220) | Core shell | 57 VQHIKDYHIDSVSSK |
| BA71V-CP2475L (P220) | BA71V-CP2475L (p220) | Core shell | 58 QHIKDYHIDSVSSKA |
| BA71V-CP2475L (P220) | BA71V-CP2475L (p220) | Core shell | 59 HIKDYHIDSVSSKAK |
| BA71V-CP2475L (P220) | BA71V-CP2475L (p220) | Core shell | 60 IKDYHIDSVSSKAKL |
| BA71V-CP2475L (P220) | BA71V-CP2475L (p220) | Core shell | 61 KDYHIDSVSSKAKLR |
| BA71V-CP2475L (P220) | BA71V-CP2475L (p220) | Core shell | 62 DYHIDSVSSKAKLRI |
| BA71V-CP2475L (P220) | BA71V-CP2475L (p220) | Core shell | 63 YHIDSVSSKAKLRII |
| BA71V-CP2475L (P220) | BA71V-CP2475L (p220) | Core shell | 64 HIDSVSSKAKLRIIE |
| BA71V-CP2475L (P220) | BA71V-CP2475L (p220) | Core shell | 65 IDSVSSKAKLRIIEG |
| BA71V-CP2475L (P220) | BA71V-CP2475L (p220) | Core shell | 66 DSVSSKAKLRIIEGI |
| BA71V-CP2475L (P220) | BA71V-CP2475L (p220) | Core shell | 67 SVSSKAKLRIIEGII |
| BA71V-CP2475L (P220) | BA71V-CP2475L (p220) | Core shell | 68 VSSKAKLRIIEGIIK |
| BA71V-CP2475L (P220) | BA71V-CP2475L (p220) | Core shell | 69 SSKAKLRIIEGIIKA |
| BA71V-CP2475L (P220) | BA71V-CP2475L (p220) | Core shell | 70 SKAKLRIIEGIIKAI |
| BA71V-CP2475L (P220) | BA71V-CP2475L (p220) | Core shell | 71 KAKLRIIEGIIKAIS |
| BA71V-CP2475L (P220) | BA71V-CP2475L (p220) | Core shell | 72 AKLRIIEGIIKAISK |
| BA71V-CP2475L (P220) | BA71V-CP2475L (p220) | Core shell | 73 KLRIIEGIIKAISKI |
| BA71V-CP2475L (P220) | BA71V-CP2475L (p220) | Core shell | 74 LRIIEGIIKAISKIG |
| BA71V-CP2475L (P220) | BA71V-CP2475L (p220) | Core shell | 75 RIIEGIIKAISKIGF |
| BA71V-CP2475L (P220) | BA71V-CP2475L (p220) | Core shell | 76 IIEGIIKAISKIGFK |
| BA71V-CP2475L (P220) | BA71V-CP2475L (p220) | Core shell | 77 IEGIIKAISKIGFKV |
| BA71V-CP2475L (P220) | BA71V-CP2475L (p220) | Core shell | 78 EGIIKAISKIGFKVD |
| BA71V-CP2475L (P220) | BA71V-CP2475L (p220) | Core shell | 79 GIIKAISKIGFKVDT |
| BA71V-CP2475L (P220) | BA71V-CP2475L (p220) | Core shell | 80 IIKAISKIGFKVDTK |
| BA71V-CP2475L (P220) | BA71V-CP2475L (p220) | Core shell | 81 IKAISKIGFKVDTKQ |
| BA71V-CP2475L (P220) | BA71V-CP2475L (p220) | Core shell | 82 KAISKIGFKVDTKQP |
| BA71V-CP2475L (P220) | BA71V-CP2475L (p220) | Core shell | 83 AISKIGFKVDTKQPI |
| BA71V-CP2475L (P220) | BA71V-CP2475L (p220) | Core shell | 84 ISKIGFKVDTKQPIE |
| BA71V-CP2475L (P220) | BA71V-CP2475L (p220) | Core shell | 85 SKIGFKVDTKQPIED |

| | | | | |
|---|---|---|---|---|
| BA71V-CP2475L (P220) | BA71V-CP2475L (p220) | Core shell | 86 | KIGFKVDTKQPIEDI |
| BA71V-CP2475L (P220) | BA71V-CP2475L (p220) | Core shell | 87 | IGFKVDTKQPIEDIL |
| BA71V-CP2475L (P220) | BA71V-CP2475L (p220) | Core shell | 88 | GFKVDTKQPIEDILK |
| BA71V-CP2475L (P220) | BA71V-CP2475L (p220) | Core shell | 89 | FKVDTKQPIEDILKD |
| BA71V-CP2475L (P220) | BA71V-CP2475L (p220) | Core shell | 90 | KVDTKQPIEDILKDI |
| BA71V-CP2475L (P220) | BA71V-CP2475L (p220) | Core shell | 91 | VDTKQPIEDILKDIK |
| BA71V-CP2475L (P220) | BA71V-CP2475L (p220) | Core shell | 92 | DTKQPIEDILKDIKK |
| BA71V-CP2475L (P220) | BA71V-CP2475L (p220) | Core shell | 93 | TKQPIEDILKDIKKQ |
| BA71V-CP2475L (P220) | BA71V-CP2475L (p220) | Core shell | 94 | KQPIEDILKDIKKQL |
| BA71V-CP2475L (P220) | BA71V-CP2475L (p220) | Core shell | 95 | QPIEDILKDIKKQLP |
| BA71V-CP2475L (P220) | BA71V-CP2475L (p220) | Core shell | 96 | PIEDILKDIKKQLPD |
| BA71V-CP2475L (P220) | BA71V-CP2475L (p220) | Core shell | 97 | IEDILKDIKKQLPDP |
| BA71V-CP2475L (P220) | BA71V-CP2475L (p220) | Core shell | 98 | EDILKDIKKQLPDPR |
| BA71V-CP2475L (P220) | BA71V-CP2475L (p220) | Core shell | 99 | DILKDIKKQLPDPRA |
| BA71V-CP2475L (P220) | BA71V-CP2475L (p220) | Core shell | 100 | ILKDIKKQLPDPRAG |
| BA71V-CP2475L (P220) | BA71V-CP2475L (p220) | Core shell | 101 | LKDIKKQLPDPRAGS |
| BA71V-CP2475L (P220) | BA71V-CP2475L (p220) | Core shell | 102 | KDIKKQLPDPRAGST |
| BA71V-CP2475L (P220) | BA71V-CP2475L (p220) | Core shell | 103 | DIKKQLPDPRAGSTF |
| BA71V-CP2475L (P220) | BA71V-CP2475L (p220) | Core shell | 104 | IKKQLPDPRAGSTFV |
| BA71V-CP2475L (P220) | BA71V-CP2475L (p220) | Core shell | 105 | KKQLPDPRAGSTFVK |
| BA71V-CP2475L (P220) | BA71V-CP2475L (p220) | Core shell | 106 | KQLPDPRAGSTFVKN |
| BA71V-CP2475L (P220) | BA71V-CP2475L (p220) | Core shell | 107 | QLPDPRAGSTFVKNA |
| BA71V-CP2475L (P220) | BA71V-CP2475L (p220) | Core shell | 108 | LPDPRAGSTFVKNAE |
| BA71V-CP2475L (P220) | BA71V-CP2475L (p220) | Core shell | 109 | PDPRAGSTFVKNAEK |
| BA71V-CP2475L (P220) | BA71V-CP2475L (p220) | Core shell | 110 | DPRAGSTFVKNAEKQ |
| BA71V-CP2475L (P220) | BA71V-CP2475L (p220) | Core shell | 111 | PRAGSTFVKNAEKQE |
| BA71V-CP2475L (P220) | BA71V-CP2475L (p220) | Core shell | 112 | RAGSTFVKNAEKQET |
| BA71V-CP2475L (P220) | BA71V-CP2475L (p220) | Core shell | 113 | AGSTFVKNAEKQETI |
| BA71V-CP2475L (P220) | BA71V-CP2475L (p220) | Core shell | 114 | GSTFVKNAEKQETIC |
| BA71V-CP2475L (P220) | BA71V-CP2475L (p220) | Core shell | 115 | STFVKNAEKQETICK |
| BA71V-CP2475L (P220) | BA71V-CP2475L (p220) | Core shell | 116 | TFVKNAEKQETICKM |
| BA71V-CP2475L (P220) | BA71V-CP2475L (p220) | Core shell | 117 | FVKNAEKQETICKMI |
| BA71V-CP2475L (P220) | BA71V-CP2475L (p220) | Core shell | 118 | VKNAEKQETICKMIA |
| BA71V-CP2475L (P220) | BA71V-CP2475L (p220) | Core shell | 119 | KNAEKQETICKMIAD |
| BA71V-CP2475L (P220) | BA71V-CP2475L (p220) | Core shell | 120 | NAEKQETICKMIADA |
| BA71V-CP2475L (P220) | BA71V-CP2475L (p220) | Core shell | 121 | AEKQETICKMIADAI |
| BA71V-CP2475L (P220) | BA71V-CP2475L (p220) | Core shell | 122 | EKQETICKMIADAIN |
| BA71V-CP2475L (P220) | BA71V-CP2475L (p220) | Core shell | 123 | KQETICKMIADAINQ |
| BA71V-CP2475L (P220) | BA71V-CP2475L (p220) | Core shell | 124 | QETICKMIADAINQE |
| BA71V-CP2475L (P220) | BA71V-CP2475L (p220) | Core shell | 125 | ETICKMIADAINQEF |
| BA71V-CP2475L (P220) | BA71V-CP2475L (p220) | Core shell | 126 | TICKMIADAINQEFI |
| BA71V-CP2475L (P220) | BA71V-CP2475L (p220) | Core shell | 127 | ICKMIADAINQEFID |
| BA71V-CP2475L (P220) | BA71V-CP2475L (p220) | Core shell | 128 | CKMIADAINQEFIDL |
| BA71V-CP2475L (P220) | BA71V-CP2475L (p220) | Core shell | 129 | KMIADAINQEFIDLG |
| BA71V-CP2475L (P220) | BA71V-CP2475L (p220) | Core shell | 130 | MIADAINQEFIDLGQ |
| BA71V-CP2475L (P220) | BA71V-CP2475L (p220) | Core shell | 131 | IADAINQEFIDLGQD |
| BA71V-CP2475L (P220) | BA71V-CP2475L (p220) | Core shell | 132 | ADAINQEFIDLGQDK |
| BA71V-CP2475L (P220) | BA71V-CP2475L (p220) | Core shell | 133 | DAINQEFIDLGQDKL |
| BA71V-CP2475L (P220) | BA71V-CP2475L (p220) | Core shell | 134 | AINQEFIDLGQDKLI |
| BA71V-CP2475L (P220) | BA71V-CP2475L (p220) | Core shell | 135 | INQEFIDLGQDKLID |
| BA71V-CP2475L (P220) | BA71V-CP2475L (p220) | Core shell | 136 | NQEFIDLGQDKLIDT |
| BA71V-CP2475L (P220) | BA71V-CP2475L (p220) | Core shell | 137 | QEFIDLGQDKLIDTT |
| BA71V-CP2475L (P220) | BA71V-CP2475L (p220) | Core shell | 138 | EFIDLGQDKLIDTTE |
| BA71V-CP2475L (P220) | BA71V-CP2475L (p220) | Core shell | 139 | FIDLGQDKLIDTTEG |
| BA71V-CP2475L (P220) | BA71V-CP2475L (p220) | Core shell | 140 | IDLGQDKLIDTTEGA |
| BA71V-CP2475L (P220) | BA71V-CP2475L (p220) | Core shell | 141 | DLGQDKLIDTTEGAA |
| BA71V-CP2475L (P220) | BA71V-CP2475L (p220) | Core shell | 142 | LGQDKLIDTTEGAAS |
| BA71V-CP2475L (P220) | BA71V-CP2475L (p220) | Core shell | 143 | GQDKLIDTTEGAASI |
| BA71V-CP2475L (P220) | BA71V-CP2475L (p220) | Core shell | 144 | QDKLIDTTEGAASIC |
| BA71V-CP2475L (P220) | BA71V-CP2475L (p220) | Core shell | 145 | DKLIDTTEGAASICR |
| BA71V-CP2475L (P220) | BA71V-CP2475L (p220) | Core shell | 146 | KLIDTTEGAASICRQ |
| BA71V-CP2475L (P220) | BA71V-CP2475L (p220) | Core shell | 147 | LIDTTEGAASICRQI |
| BA71V-CP2475L (P220) | BA71V-CP2475L (p220) | Core shell | 148 | IDTTEGAASICRQIV |
| BA71V-CP2475L (P220) | BA71V-CP2475L (p220) | Core shell | 149 | DTTEGAASICRQIVL |
| BA71V-CP2475L (P220) | BA71V-CP2475L (p220) | Core shell | 150 | TTEGAASICRQIVLY |
| BA71V-CP2475L (P220) | BA71V-CP2475L (p220) | Core shell | 151 | TEGAASICRQIVLYI |
| BA71V-CP2475L (P220) | BA71V-CP2475L (p220) | Core shell | 152 | EGAASICRQIVLYIN |

| | | | | |
|---|---|---|---|---|
| BA71V-CP2475L (P220) | BA71V-CP2475L (p220) | Core shell | 153 | GAASICRQIVLYINS |
| BA71V-CP2475L (P220) | BA71V-CP2475L (p220) | Core shell | 154 | AASICRQIVLYINSL |
| BA71V-CP2475L (P220) | BA71V-CP2475L (p220) | Core shell | 155 | ASICRQIVLYINSLT |
| BA71V-CP2475L (P220) | BA71V-CP2475L (p220) | Core shell | 156 | SICRQIVLYINSLTH |
| BA71V-CP2475L (P220) | BA71V-CP2475L (p220) | Core shell | 157 | ICRQIVLYINSLTHG |
| BA71V-CP2475L (P220) | BA71V-CP2475L (p220) | Core shell | 158 | CRQIVLYINSLTHGL |
| BA71V-CP2475L (P220) | BA71V-CP2475L (p220) | Core shell | 159 | RQIVLYINSLTHGLR |
| BA71V-CP2475L (P220) | BA71V-CP2475L (p220) | Core shell | 160 | QIVLYINSLTHGLRA |
| BA71V-CP2475L (P220) | BA71V-CP2475L (p220) | Core shell | 161 | IVLYINSLTHGLRAE |
| BA71V-CP2475L (P220) | BA71V-CP2475L (p220) | Core shell | 162 | VLYINSLTHGLRAEY |
| BA71V-CP2475L (P220) | BA71V-CP2475L (p220) | Core shell | 163 | LYINSLTHGLRAEYL |
| BA71V-CP2475L (P220) | BA71V-CP2475L (p220) | Core shell | 164 | YINSLTHGLRAEYLD |
| BA71V-CP2475L (P220) | BA71V-CP2475L (p220) | Core shell | 165 | INSLTHGLRAEYLDV |
| BA71V-CP2475L (P220) | BA71V-CP2475L (p220) | Core shell | 166 | NSLTHGLRAEYLDVH |
| BA71V-CP2475L (P220) | BA71V-CP2475L (p220) | Core shell | 167 | SLTHGLRAEYLDVHG |
| BA71V-CP2475L (P220) | BA71V-CP2475L (p220) | Core shell | 168 | LTHGLRAEYLDVHGS |
| BA71V-CP2475L (P220) | BA71V-CP2475L (p220) | Core shell | 169 | THGLRAEYLDVHGSI |
| BA71V-CP2475L (P220) | BA71V-CP2475L (p220) | Core shell | 170 | HGLRAEYLDVHGSIE |
| BA71V-CP2475L (P220) | BA71V-CP2475L (p220) | Core shell | 171 | GLRAEYLDVHGSIEN |
| BA71V-CP2475L (P220) | BA71V-CP2475L (p220) | Core shell | 172 | LRAEYLDVHGSIENT |
| BA71V-CP2475L (P220) | BA71V-CP2475L (p220) | Core shell | 173 | RAEYLDVHGSIENTL |
| BA71V-CP2475L (P220) | BA71V-CP2475L (p220) | Core shell | 174 | AEYLDVHGSIENTLE |
| BA71V-CP2475L (P220) | BA71V-CP2475L (p220) | Core shell | 175 | EYLDVHGSIENTLEN |
| BA71V-CP2475L (P220) | BA71V-CP2475L (p220) | Core shell | 176 | YLDVHGSIENTLENI |
| BA71V-CP2475L (P220) | BA71V-CP2475L (p220) | Core shell | 177 | LDVHGSIENTLENIK |
| BA71V-CP2475L (P220) | BA71V-CP2475L (p220) | Core shell | 178 | DVHGSIENTLENIKL |
| BA71V-CP2475L (P220) | BA71V-CP2475L (p220) | Core shell | 179 | VHGSIENTLENIKLL |
| BA71V-CP2475L (P220) | BA71V-CP2475L (p220) | Core shell | 180 | HGSIENTLENIKLLS |
| BA71V-CP2475L (P220) | BA71V-CP2475L (p220) | Core shell | 181 | GSIENTLENIKLLSD |
| BA71V-CP2475L (P220) | BA71V-CP2475L (p220) | Core shell | 182 | SIENTLENIKLLSDA |
| BA71V-CP2475L (P220) | BA71V-CP2475L (p220) | Core shell | 183 | IENTLENIKLLSDAI |
| BA71V-CP2475L (P220) | BA71V-CP2475L (p220) | Core shell | 184 | ENTLENIKLLSDAIK |
| BA71V-CP2475L (P220) | BA71V-CP2475L (p220) | Core shell | 185 | NTLENIKLLSDAIKQ |
| BA71V-CP2475L (P220) | BA71V-CP2475L (p220) | Core shell | 186 | TLENIKLLSDAIKQL |
| BA71V-CP2475L (P220) | BA71V-CP2475L (p220) | Core shell | 187 | LENIKLLSDAIKQLH |
| BA71V-CP2475L (P220) | BA71V-CP2475L (p220) | Core shell | 188 | ENIKLLSDAIKQLHE |
| BA71V-CP2475L (P220) | BA71V-CP2475L (p220) | Core shell | 189 | NIKLLSDAIKQLHER |
| BA71V-CP2475L (P220) | BA71V-CP2475L (p220) | Core shell | 190 | IKLLSDAIKQLHERM |
| BA71V-CP2475L (P220) | BA71V-CP2475L (p220) | Core shell | 191 | KLLSDAIKQLHERMV |
| BA71V-CP2475L (P220) | BA71V-CP2475L (p220) | Core shell | 192 | LLSDAIKQLHERMVT |
| BA71V-CP2475L (P220) | BA71V-CP2475L (p220) | Core shell | 193 | LSDAIKQLHERMVTE |
| BA71V-CP2475L (P220) | BA71V-CP2475L (p220) | Core shell | 194 | SDAIKQLHERMVTEV |
| BA71V-CP2475L (P220) | BA71V-CP2475L (p220) | Core shell | 195 | DAIKQLHERMVTEVT |
| BA71V-CP2475L (P220) | BA71V-CP2475L (p220) | Core shell | 196 | AIKQLHERMVTEVTK |
| BA71V-CP2475L (P220) | BA71V-CP2475L (p220) | Core shell | 197 | IKQLHERMVTEVTKA |
| BA71V-CP2475L (P220) | BA71V-CP2475L (p220) | Core shell | 198 | KQLHERMVTEVTKAA |
| BA71V-CP2475L (P220) | BA71V-CP2475L (p220) | Core shell | 199 | QLHERMVTEVTKAAP |
| BA71V-CP2475L (P220) | BA71V-CP2475L (p220) | Core shell | 200 | LHERMVTEVTKAAPN |
| BA71V-CP2475L (P220) | BA71V-CP2475L (p220) | Core shell | 201 | HERMVTEVTKAAPNE |
| BA71V-CP2475L (P220) | BA71V-CP2475L (p220) | Core shell | 202 | ERMVTEVTKAAPNEE |
| BA71V-CP2475L (P220) | BA71V-CP2475L (p220) | Core shell | 203 | RMVTEVTKAAPNEEV |
| BA71V-CP2475L (P220) | BA71V-CP2475L (p220) | Core shell | 204 | MVTEVTKAAPNEEVI |
| BA71V-CP2475L (P220) | BA71V-CP2475L (p220) | Core shell | 205 | VTEVTKAAPNEEVIN |
| BA71V-CP2475L (P220) | BA71V-CP2475L (p220) | Core shell | 206 | TEVTKAAPNEEVINA |
| BA71V-CP2475L (P220) | BA71V-CP2475L (p220) | Core shell | 207 | EVTKAAPNEEVINAV |
| BA71V-CP2475L (P220) | BA71V-CP2475L (p220) | Core shell | 208 | VTKAAPNEEVINAVT |
| BA71V-CP2475L (P220) | BA71V-CP2475L (p220) | Core shell | 209 | TKAAPNEEVINAVTM |
| BA71V-CP2475L (P220) | BA71V-CP2475L (p220) | Core shell | 210 | KAAPNEEVINAVTMI |
| BA71V-CP2475L (P220) | BA71V-CP2475L (p220) | Core shell | 211 | AAPNEEVINAVTMIE |
| BA71V-CP2475L (P220) | BA71V-CP2475L (p220) | Core shell | 212 | APNEEVINAVTMIEA |
| BA71V-CP2475L (P220) | BA71V-CP2475L (p220) | Core shell | 213 | PNEEVINAVTMIEAV |
| BA71V-CP2475L (P220) | BA71V-CP2475L (p220) | Core shell | 214 | NEEVINAVTMIEAVY |
| BA71V-CP2475L (P220) | BA71V-CP2475L (p220) | Core shell | 215 | EEVINAVTMIEAVYR |
| BA71V-CP2475L (P220) | BA71V-CP2475L (p220) | Core shell | 216 | EVINAVTMIEAVYRR |
| BA71V-CP2475L (P220) | BA71V-CP2475L (p220) | Core shell | 217 | VINAVTMIEAVYRRL |
| BA71V-CP2475L (P220) | BA71V-CP2475L (p220) | Core shell | 218 | INAVTMIEAVYRRLL |
| BA71V-CP2475L (P220) | BA71V-CP2475L (p220) | Core shell | 219 | NAVTMIEAVYRRLLN |

| | | | |
|---|---|---|---|
| BA71V-CP2475L (P220) | BA71V-CP2475L (p220) | Core shell | 220 AVTMIEAVYRRLLNE |
| BA71V-CP2475L (P220) | BA71V-CP2475L (p220) | Core shell | 221 VTMIEAVYRRLLNEQ |
| BA71V-CP2475L (P220) | BA71V-CP2475L (p220) | Core shell | 222 TMIEAVYRRLLNEQN |
| BA71V-CP2475L (P220) | BA71V-CP2475L (p220) | Core shell | 223 MIEAVYRRLLNEQNL |
| BA71V-CP2475L (P220) | BA71V-CP2475L (p220) | Core shell | 224 IEAVYRRLLNEQNLQ |
| BA71V-CP2475L (P220) | BA71V-CP2475L (p220) | Core shell | 225 EAVYRRLLNEQNLQI |
| BA71V-CP2475L (P220) | BA71V-CP2475L (p220) | Core shell | 226 AVYRRLLNEQNLQIN |
| BA71V-CP2475L (P220) | BA71V-CP2475L (p220) | Core shell | 227 VYRRLLNEQNLQINI |
| BA71V-CP2475L (P220) | BA71V-CP2475L (p220) | Core shell | 228 YRRLLNEQNLQINIL |
| BA71V-CP2475L (P220) | BA71V-CP2475L (p220) | Core shell | 229 RRLLNEQNLQINILT |
| BA71V-CP2475L (P220) | BA71V-CP2475L (p220) | Core shell | 230 RLLNEQNLQINILTN |
| BA71V-CP2475L (P220) | BA71V-CP2475L (p220) | Core shell | 231 LLNEQNLQINILTNF |
| BA71V-CP2475L (P220) | BA71V-CP2475L (p220) | Core shell | 232 LNEQNLQINILTNFI |
| BA71V-CP2475L (P220) | BA71V-CP2475L (p220) | Core shell | 233 NEQNLQINILTNFID |
| BA71V-CP2475L (P220) | BA71V-CP2475L (p220) | Core shell | 234 EQNLQINILTNFIDN |
| BA71V-CP2475L (P220) | BA71V-CP2475L (p220) | Core shell | 235 QNLQINILTNFIDNI |
| BA71V-CP2475L (P220) | BA71V-CP2475L (p220) | Core shell | 236 NLQINILTNFIDNIL |
| BA71V-CP2475L (P220) | BA71V-CP2475L (p220) | Core shell | 237 LQINILTNFIDNILT |
| BA71V-CP2475L (P220) | BA71V-CP2475L (p220) | Core shell | 238 QINILTNFIDNILTP |
| BA71V-CP2475L (P220) | BA71V-CP2475L (p220) | Core shell | 239 INILTNFIDNILTPT |
| BA71V-CP2475L (P220) | BA71V-CP2475L (p220) | Core shell | 240 NILTNFIDNILTPTQ |
| BA71V-CP2475L (P220) | BA71V-CP2475L (p220) | Core shell | 241 ILTNFIDNILTPTQK |
| BA71V-CP2475L (P220) | BA71V-CP2475L (p220) | Core shell | 242 LTNFIDNILTPTQKE |
| BA71V-CP2475L (P220) | BA71V-CP2475L (p220) | Core shell | 243 TNFIDNILTPTQKEL |
| BA71V-CP2475L (P220) | BA71V-CP2475L (p220) | Core shell | 244 NFIDNILTPTQKELD |
| BA71V-CP2475L (P220) | BA71V-CP2475L (p220) | Core shell | 245 FIDNILTPTQKELDK |
| BA71V-CP2475L (P220) | BA71V-CP2475L (p220) | Core shell | 246 IDNILTPTQKELDKL |
| BA71V-CP2475L (P220) | BA71V-CP2475L (p220) | Core shell | 247 DNILTPTQKELDKLK |
| BA71V-CP2475L (P220) | BA71V-CP2475L (p220) | Core shell | 248 NILTPTQKELDKLKT |
| BA71V-CP2475L (P220) | BA71V-CP2475L (p220) | Core shell | 249 ILTPTQKELDKLKTD |
| BA71V-CP2475L (P220) | BA71V-CP2475L (p220) | Core shell | 250 LTPTQKELDKLKTDE |
| BA71V-CP2475L (P220) | BA71V-CP2475L (p220) | Core shell | 251 TPTQKELDKLKTDEV |
| BA71V-CP2475L (P220) | BA71V-CP2475L (p220) | Core shell | 252 PTQKELDKLKTDEVD |
| BA71V-CP2475L (P220) | BA71V-CP2475L (p220) | Core shell | 253 TQKELDKLKTDEVDI |
| BA71V-CP2475L (P220) | BA71V-CP2475L (p220) | Core shell | 254 QKELDKLKTDEVDII |
| BA71V-CP2475L (P220) | BA71V-CP2475L (p220) | Core shell | 255 KELDKLKTDEVDIIK |
| BA71V-CP2475L (P220) | BA71V-CP2475L (p220) | Core shell | 256 ELDKLKTDEVDIIKI |
| BA71V-CP2475L (P220) | BA71V-CP2475L (p220) | Core shell | 257 LDKLKTDEVDIIKIL |
| BA71V-CP2475L (P220) | BA71V-CP2475L (p220) | Core shell | 258 DKLKTDEVDIIKILN |
| BA71V-CP2475L (P220) | BA71V-CP2475L (p220) | Core shell | 259 KLKTDEVDIIKILND |
| BA71V-CP2475L (P220) | BA71V-CP2475L (p220) | Core shell | 260 LKTDEVDIIKILNDT |
| BA71V-CP2475L (P220) | BA71V-CP2475L (p220) | Core shell | 261 KTDEVDIIKILNDTN |
| BA71V-CP2475L (P220) | BA71V-CP2475L (p220) | Core shell | 262 TDEVDIIKILNDTNS |
| BA71V-CP2475L (P220) | BA71V-CP2475L (p220) | Core shell | 263 DEVDIIKILNDTNSV |
| BA71V-CP2475L (P220) | BA71V-CP2475L (p220) | Core shell | 264 EVDIIKILNDTNSVL |
| BA71V-CP2475L (P220) | BA71V-CP2475L (p220) | Core shell | 265 VDIIKILNDTNSVLG |
| BA71V-CP2475L (P220) | BA71V-CP2475L (p220) | Core shell | 266 DIIKILNDTNSVLGT |
| BA71V-CP2475L (P220) | BA71V-CP2475L (p220) | Core shell | 267 IIKILNDTNSVLGTK |
| BA71V-CP2475L (P220) | BA71V-CP2475L (p220) | Core shell | 268 IKILNDTNSVLGTKN |
| BA71V-CP2475L (P220) | BA71V-CP2475L (p220) | Core shell | 269 KILNDTNSVLGTKNF |
| BA71V-CP2475L (P220) | BA71V-CP2475L (p220) | Core shell | 270 ILNDTNSVLGTKNFG |
| BA71V-CP2475L (P220) | BA71V-CP2475L (p220) | Core shell | 271 LNDTNSVLGTKNFGK |
| BA71V-CP2475L (P220) | BA71V-CP2475L (p220) | Core shell | 272 NDTNSVLGTKNFGKV |
| BA71V-CP2475L (P220) | BA71V-CP2475L (p220) | Core shell | 273 DTNSVLGTKNFGKVL |
| BA71V-CP2475L (P220) | BA71V-CP2475L (p220) | Core shell | 274 TNSVLGTKNFGKVLS |
| BA71V-CP2475L (P220) | BA71V-CP2475L (p220) | Core shell | 275 NSVLGTKNFGKVLSY |
| BA71V-CP2475L (P220) | BA71V-CP2475L (p220) | Core shell | 276 SVLGTKNFGKVLSYT |
| BA71V-CP2475L (P220) | BA71V-CP2475L (p220) | Core shell | 277 VLGTKNFGKVLSYTL |
| BA71V-CP2475L (P220) | BA71V-CP2475L (p220) | Core shell | 278 LGTKNFGKVLSYTLC |
| BA71V-CP2475L (P220) | BA71V-CP2475L (p220) | Core shell | 279 GTKNFGKVLSYTLCN |
| BA71V-CP2475L (P220) | BA71V-CP2475L (p220) | Core shell | 280 TKNFGKVLSYTLCNL |
| BA71V-CP2475L (P220) | BA71V-CP2475L (p220) | Core shell | 281 KNFGKVLSYTLCNLG |
| BA71V-CP2475L (P220) | BA71V-CP2475L (p220) | Core shell | 282 NFGKVLSYTLCNLGI |
| BA71V-CP2475L (P220) | BA71V-CP2475L (p220) | Core shell | 283 FGKVLSYTLCNLGIA |
| BA71V-CP2475L (P220) | BA71V-CP2475L (p220) | Core shell | 284 GKVLSYTLCNLGIAA |
| BA71V-CP2475L (P220) | BA71V-CP2475L (p220) | Core shell | 285 KVLSYTLCNLGIAAT |
| BA71V-CP2475L (P220) | BA71V-CP2475L (p220) | Core shell | 286 VLSYTLCNLGIAATV |

| | | | | |
|---|---|---|---|---|
| BA71V-CP2475L (P220) | BA71V-CP2475L (p220) | Core shell | 287 | LSYTLCNLGIAATVA |
| BA71V-CP2475L (P220) | BA71V-CP2475L (p220) | Core shell | 288 | SYTLCNLGIAATVAN |
| BA71V-CP2475L (P220) | BA71V-CP2475L (p220) | Core shell | 289 | YTLCNLGIAATVANK |
| BA71V-CP2475L (P220) | BA71V-CP2475L (p220) | Core shell | 290 | TLCNLGIAATVANKI |
| BA71V-CP2475L (P220) | BA71V-CP2475L (p220) | Core shell | 291 | LCNLGIAATVANKIN |
| BA71V-CP2475L (P220) | BA71V-CP2475L (p220) | Core shell | 292 | CNLGIAATVANKINK |
| BA71V-CP2475L (P220) | BA71V-CP2475L (p220) | Core shell | 293 | NLGIAATVANKINKA |
| BA71V-CP2475L (P220) | BA71V-CP2475L (p220) | Core shell | 294 | LGIAATVANKINKAL |
| BA71V-CP2475L (P220) | BA71V-CP2475L (p220) | Core shell | 295 | GIAATVANKINKALQ |
| BA71V-CP2475L (P220) | BA71V-CP2475L (p220) | Core shell | 296 | IAATVANKINKALQR |
| BA71V-CP2475L (P220) | BA71V-CP2475L (p220) | Core shell | 297 | AATVANKINKALQRV |
| BA71V-CP2475L (P220) | BA71V-CP2475L (p220) | Core shell | 298 | ATVANKINKALQRVG |
| BA71V-CP2475L (P220) | BA71V-CP2475L (p220) | Core shell | 299 | TVANKINKALQRVGL |
| BA71V-CP2475L (P220) | BA71V-CP2475L (p220) | Core shell | 300 | VANKINKALQRVGLK |
| BA71V-CP2475L (P220) | BA71V-CP2475L (p220) | Core shell | 301 | ANKINKALQRVGLKV |
| BA71V-CP2475L (P220) | BA71V-CP2475L (p220) | Core shell | 302 | NKINKALQRVGLKVE |
| BA71V-CP2475L (P220) | BA71V-CP2475L (p220) | Core shell | 303 | KINKALQRVGLKVEQ |
| BA71V-CP2475L (P220) | BA71V-CP2475L (p220) | Core shell | 304 | INKALQRVGLKVEQY |
| BA71V-CP2475L (P220) | BA71V-CP2475L (p220) | Core shell | 305 | NKALQRVGLKVEQYL |
| BA71V-CP2475L (P220) | BA71V-CP2475L (p220) | Core shell | 306 | KALQRVGLKVEQYLH |
| BA71V-CP2475L (P220) | BA71V-CP2475L (p220) | Core shell | 307 | ALQRVGLKVEQYLHS |
| BA71V-CP2475L (P220) | BA71V-CP2475L (p220) | Core shell | 308 | LQRVGLKVEQYLHSK |
| BA71V-CP2475L (P220) | BA71V-CP2475L (p220) | Core shell | 309 | QRVGLKVEQYLHSKN |
| BA71V-CP2475L (P220) | BA71V-CP2475L (p220) | Core shell | 310 | RVGLKVEQYLHSKNW |
| BA71V-CP2475L (P220) | BA71V-CP2475L (p220) | Core shell | 311 | VGLKVEQYLHSKNWA |
| BA71V-CP2475L (P220) | BA71V-CP2475L (p220) | Core shell | 312 | GLKVEQYLHSKNWAE |
| BA71V-CP2475L (P220) | BA71V-CP2475L (p220) | Core shell | 313 | LKVEQYLHSKNWAEF |
| BA71V-CP2475L (P220) | BA71V-CP2475L (p220) | Core shell | 314 | KVEQYLHSKNWAEFD |
| BA71V-CP2475L (P220) | BA71V-CP2475L (p220) | Core shell | 315 | VEQYLHSKNWAEFDK |
| BA71V-CP2475L (P220) | BA71V-CP2475L (p220) | Core shell | 316 | EQYLHSKNWAEFDKE |
| BA71V-CP2475L (P220) | BA71V-CP2475L (p220) | Core shell | 317 | QYLHSKNWAEFDKEL |
| BA71V-CP2475L (P220) | BA71V-CP2475L (p220) | Core shell | 318 | YLHSKNWAEFDKELD |
| BA71V-CP2475L (P220) | BA71V-CP2475L (p220) | Core shell | 319 | LHSKNWAEFDKELDL |
| BA71V-CP2475L (P220) | BA71V-CP2475L (p220) | Core shell | 320 | HSKNWAEFDKELDLK |
| BA71V-CP2475L (P220) | BA71V-CP2475L (p220) | Core shell | 321 | SKNWAEFDKELDLKR |
| BA71V-CP2475L (P220) | BA71V-CP2475L (p220) | Core shell | 322 | KNWAEFDKELDLKRF |
| BA71V-CP2475L (P220) | BA71V-CP2475L (p220) | Core shell | 323 | NWAEFDKELDLKRFS |
| BA71V-CP2475L (P220) | BA71V-CP2475L (p220) | Core shell | 324 | WAEFDKELDLKRFSG |
| BA71V-CP2475L (P220) | BA71V-CP2475L (p220) | Core shell | 325 | AEFDKELDLKRFSGL |
| BA71V-CP2475L (P220) | BA71V-CP2475L (p220) | Core shell | 326 | EFDKELDLKRFSGLV |
| BA71V-CP2475L (P220) | BA71V-CP2475L (p220) | Core shell | 327 | FDKELDLKRFSGLVS |
| BA71V-CP2475L (P220) | BA71V-CP2475L (p220) | Core shell | 328 | DKELDLKRFSGLVSA |
| BA71V-CP2475L (P220) | BA71V-CP2475L (p220) | Core shell | 329 | KELDLKRFSGLVSAE |
| BA71V-CP2475L (P220) | BA71V-CP2475L (p220) | Core shell | 330 | ELDLKRFSGLVSAEN |
| BA71V-CP2475L (P220) | BA71V-CP2475L (p220) | Core shell | 331 | LDLKRFSGLVSAENI |
| BA71V-CP2475L (P220) | BA71V-CP2475L (p220) | Core shell | 332 | DLKRFSGLVSAENIA |
| BA71V-CP2475L (P220) | BA71V-CP2475L (p220) | Core shell | 333 | LKRFSGLVSAENIAE |
| BA71V-CP2475L (P220) | BA71V-CP2475L (p220) | Core shell | 334 | KRFSGLVSAENIAEF |
| BA71V-CP2475L (P220) | BA71V-CP2475L (p220) | Core shell | 335 | RFSGLVSAENIAEFE |
| BA71V-CP2475L (P220) | BA71V-CP2475L (p220) | Core shell | 336 | FSGLVSAENIAEFEK |
| BA71V-CP2475L (P220) | BA71V-CP2475L (p220) | Core shell | 337 | SGLVSAENIAEFEKA |
| BA71V-CP2475L (P220) | BA71V-CP2475L (p220) | Core shell | 338 | GLVSAENIAEFEKAV |
| BA71V-CP2475L (P220) | BA71V-CP2475L (p220) | Core shell | 339 | LVSAENIAEFEKAVN |
| BA71V-CP2475L (P220) | BA71V-CP2475L (p220) | Core shell | 340 | VSAENIAEFEKAVNL |
| BA71V-CP2475L (P220) | BA71V-CP2475L (p220) | Core shell | 341 | SAENIAEFEKAVNLL |
| BA71V-CP2475L (P220) | BA71V-CP2475L (p220) | Core shell | 342 | AENIAEFEKAVNLLR |
| BA71V-CP2475L (P220) | BA71V-CP2475L (p220) | Core shell | 343 | ENIAEFEKAVNLLRQ |
| BA71V-CP2475L (P220) | BA71V-CP2475L (p220) | Core shell | 344 | NIAEFEKAVNLLRQT |
| BA71V-CP2475L (P220) | BA71V-CP2475L (p220) | Core shell | 345 | IAEFEKAVNLLRQTF |
| BA71V-CP2475L (P220) | BA71V-CP2475L (p220) | Core shell | 346 | AEFEKAVNLLRQTFN |
| BA71V-CP2475L (P220) | BA71V-CP2475L (p220) | Core shell | 347 | EFEKAVNLLRQTFNE |
| BA71V-CP2475L (P220) | BA71V-CP2475L (p220) | Core shell | 348 | FEKAVNLLRQTFNER |
| BA71V-CP2475L (P220) | BA71V-CP2475L (p220) | Core shell | 349 | EKAVNLLRQTFNERH |
| BA71V-CP2475L (P220) | BA71V-CP2475L (p220) | Core shell | 350 | KAVNLLRQTFNERHK |
| BA71V-CP2475L (P220) | BA71V-CP2475L (p220) | Core shell | 351 | AVNLLRQTFNERHKI |
| BA71V-CP2475L (P220) | BA71V-CP2475L (p220) | Core shell | 352 | VNLLRQTFNERHKIL |
| BA71V-CP2475L (P220) | BA71V-CP2475L (p220) | Core shell | 353 | NLLRQTFNERHKILE |

| | | | | |
|---|---|---|---|---|
| BA71V-CP2475L (P220) | BA71V-CP2475L (p220) | Core shell | 354 | LLRQTFNERHKILEN |
| BA71V-CP2475L (P220) | BA71V-CP2475L (p220) | Core shell | 355 | LRQTFNERHKILENN |
| BA71V-CP2475L (P220) | BA71V-CP2475L (p220) | Core shell | 356 | RQTFNERHKILENNC |
| BA71V-CP2475L (P220) | BA71V-CP2475L (p220) | Core shell | 357 | QTFNERHKILENNCA |
| BA71V-CP2475L (P220) | BA71V-CP2475L (p220) | Core shell | 358 | TFNERHKILENNCAK |
| BA71V-CP2475L (P220) | BA71V-CP2475L (p220) | Core shell | 359 | FNERHKILENNCAKK |
| BA71V-CP2475L (P220) | BA71V-CP2475L (p220) | Core shell | 360 | NERHKILENNCAKKG |
| BA71V-CP2475L (P220) | BA71V-CP2475L (p220) | Core shell | 361 | ERHKILENNCAKKGG |
| BA71V-CP2475L (P220) | BA71V-CP2475L (p220) | Core shell | 362 | RHKILENNCAKKGGD |
| BA71V-CP2475L (P220) | BA71V-CP2475L (p220) | Core shell | 363 | HKILENNCAKKGGDE |
| BA71V-CP2475L (P220) | BA71V-CP2475L (p220) | Core shell | 364 | KILENNCAKKGGDEE |
| BA71V-CP2475L (P220) | BA71V-CP2475L (p220) | Core shell | 365 | ILENNCAKKGGDEEK |
| BA71V-CP2475L (P220) | BA71V-CP2475L (p220) | Core shell | 366 | LENNCAKKGGDEEKT |
| BA71V-CP2475L (P220) | BA71V-CP2475L (p220) | Core shell | 367 | ENNCAKKGGDEEKTP |
| BA71V-CP2475L (P220) | BA71V-CP2475L (p220) | Core shell | 368 | NNCAKKGGDEEKTPL |
| BA71V-CP2475L (P220) | BA71V-CP2475L (p220) | Core shell | 369 | NCAKKGGDEEKTPLD |
| BA71V-CP2475L (P220) | BA71V-CP2475L (p220) | Core shell | 370 | CAKKGGDEEKTPLDK |
| BA71V-CP2475L (P220) | BA71V-CP2475L (p220) | Core shell | 371 | AKKGGDEEKTPLDKR |
| BA71V-CP2475L (P220) | BA71V-CP2475L (p220) | Core shell | 372 | KKGGDEEKTPLDKRM |
| BA71V-CP2475L (P220) | BA71V-CP2475L (p220) | Core shell | 373 | KGGDEEKTPLDKRME |
| BA71V-CP2475L (P220) | BA71V-CP2475L (p220) | Core shell | 374 | GGDEEKTPLDKRMEA |
| BA71V-CP2475L (P220) | BA71V-CP2475L (p220) | Core shell | 375 | GDEEKTPLDKRMEAQ |
| BA71V-CP2475L (P220) | BA71V-CP2475L (p220) | Core shell | 376 | DEEKTPLDKRMEAQR |
| BA71V-CP2475L (P220) | BA71V-CP2475L (p220) | Core shell | 377 | EEKTPLDKRMEAQRL |
| BA71V-CP2475L (P220) | BA71V-CP2475L (p220) | Core shell | 378 | EKTPLDKRMEAQRLD |
| BA71V-CP2475L (P220) | BA71V-CP2475L (p220) | Core shell | 379 | KTPLDKRMEAQRLDR |
| BA71V-CP2475L (P220) | BA71V-CP2475L (p220) | Core shell | 380 | TPLDKRMEAQRLDRK |
| BA71V-CP2475L (P220) | BA71V-CP2475L (p220) | Core shell | 381 | PLDKRMEAQRLDRKH |
| BA71V-CP2475L (P220) | BA71V-CP2475L (p220) | Core shell | 382 | LDKRMEAQRLDRKHI |
| BA71V-CP2475L (P220) | BA71V-CP2475L (p220) | Core shell | 383 | DKRMEAQRLDRKHIL |
| BA71V-CP2475L (P220) | BA71V-CP2475L (p220) | Core shell | 384 | KRMEAQRLDRKHILM |
| BA71V-CP2475L (P220) | BA71V-CP2475L (p220) | Core shell | 385 | RMEAQRLDRKHILME |
| BA71V-CP2475L (P220) | BA71V-CP2475L (p220) | Core shell | 386 | MEAQRLDRKHILMEF |
| BA71V-CP2475L (P220) | BA71V-CP2475L (p220) | Core shell | 387 | EAQRLDRKHILMEFL |
| BA71V-CP2475L (P220) | BA71V-CP2475L (p220) | Core shell | 388 | AQRLDRKHILMEFLN |
| BA71V-CP2475L (P220) | BA71V-CP2475L (p220) | Core shell | 389 | QRLDRKHILMEFLNK |
| BA71V-CP2475L (P220) | BA71V-CP2475L (p220) | Core shell | 390 | RLDRKHILMEFLNKS |
| BA71V-CP2475L (P220) | BA71V-CP2475L (p220) | Core shell | 391 | LDRKHILMEFLNKST |
| BA71V-CP2475L (P220) | BA71V-CP2475L (p220) | Core shell | 392 | DRKHILMEFLNKSTQ |
| BA71V-CP2475L (P220) | BA71V-CP2475L (p220) | Core shell | 393 | RKHILMEFLNKSTQA |
| BA71V-CP2475L (P220) | BA71V-CP2475L (p220) | Core shell | 394 | KHILMEFLNKSTQAY |
| BA71V-CP2475L (P220) | BA71V-CP2475L (p220) | Core shell | 395 | HILMEFLNKSTQAYN |
| BA71V-CP2475L (P220) | BA71V-CP2475L (p220) | Core shell | 396 | ILMEFLNKSTQAYND |
| BA71V-CP2475L (P220) | BA71V-CP2475L (p220) | Core shell | 397 | LMEFLNKSTQAYNDF |
| BA71V-CP2475L (P220) | BA71V-CP2475L (p220) | Core shell | 398 | MEFLNKSTQAYNDFL |
| BA71V-CP2475L (P220) | BA71V-CP2475L (p220) | Core shell | 399 | EFLNKSTQAYNDFLE |
| BA71V-CP2475L (P220) | BA71V-CP2475L (p220) | Core shell | 400 | FLNKSTQAYNDFLEN |
| BA71V-CP2475L (P220) | BA71V-CP2475L (p220) | Core shell | 401 | LNKSTQAYNDFLENV |
| BA71V-CP2475L (P220) | BA71V-CP2475L (p220) | Core shell | 402 | NKSTQAYNDFLENVK |
| BA71V-CP2475L (P220) | BA71V-CP2475L (p220) | Core shell | 403 | KSTQAYNDFLENVKK |
| BA71V-CP2475L (P220) | BA71V-CP2475L (p220) | Core shell | 404 | STQAYNDFLENVKKI |
| BA71V-CP2475L (P220) | BA71V-CP2475L (p220) | Core shell | 405 | TQAYNDFLENVKKIG |
| BA71V-CP2475L (P220) | BA71V-CP2475L (p220) | Core shell | 406 | QAYNDFLENVKKIGM |
| BA71V-CP2475L (P220) | BA71V-CP2475L (p220) | Core shell | 407 | AYNDFLENVKKIGMK |
| BA71V-CP2475L (P220) | BA71V-CP2475L (p220) | Core shell | 408 | YNDFLENVKKIGMKL |
| BA71V-CP2475L (P220) | BA71V-CP2475L (p220) | Core shell | 409 | NDFLENVKKIGMKLV |
| BA71V-CP2475L (P220) | BA71V-CP2475L (p220) | Core shell | 410 | DFLENVKKIGMKLVK |
| BA71V-CP2475L (P220) | BA71V-CP2475L (p220) | Core shell | 411 | FLENVKKIGMKLVKE |
| BA71V-CP2475L (P220) | BA71V-CP2475L (p220) | Core shell | 412 | LENVKKIGMKLVKEI |
| BA71V-CP2475L (P220) | BA71V-CP2475L (p220) | Core shell | 413 | ENVKKIGMKLVKEIA |
| BA71V-CP2475L (P220) | BA71V-CP2475L (p220) | Core shell | 414 | NVKKIGMKLVKEIAL |
| BA71V-CP2475L (P220) | BA71V-CP2475L (p220) | Core shell | 415 | VKKIGMKLVKEIALT |
| BA71V-CP2475L (P220) | BA71V-CP2475L (p220) | Core shell | 416 | KKIGMKLVKEIALTP |
| BA71V-CP2475L (P220) | BA71V-CP2475L (p220) | Core shell | 417 | KIGMKLVKEIALTPN |
| BA71V-CP2475L (P220) | BA71V-CP2475L (p220) | Core shell | 418 | IGMKLVKEIALTPNI |
| BA71V-CP2475L (P220) | BA71V-CP2475L (p220) | Core shell | 419 | GMKLVKEIALTPNIT |
| BA71V-CP2475L (P220) | BA71V-CP2475L (p220) | Core shell | 420 | MKLVKEIALTPNITK |

| | | | |
|---|---|---|---|
| BA71V-CP2475L (P220) | BA71V-CP2475L (p220) | Core shell | 421 KLVKEIALTPNITKL |
| BA71V-CP2475L (P220) | BA71V-CP2475L (p220) | Core shell | 422 LVKEIALTPNITKLR |
| BA71V-CP2475L (P220) | BA71V-CP2475L (p220) | Core shell | 423 VKEIALTPNITKLRD |
| BA71V-CP2475L (P220) | BA71V-CP2475L (p220) | Core shell | 424 KEIALTPNITKLRDA |
| BA71V-CP2475L (P220) | BA71V-CP2475L (p220) | Core shell | 425 EIALTPNITKLRDAL |
| BA71V-CP2475L (P220) | BA71V-CP2475L (p220) | Core shell | 426 IALTPNITKLRDALS |
| BA71V-CP2475L (P220) | BA71V-CP2475L (p220) | Core shell | 427 ALTPNITKLRDALSR |
| BA71V-CP2475L (P220) | BA71V-CP2475L (p220) | Core shell | 428 LTPNITKLRDALSRI |
| BA71V-CP2475L (P220) | BA71V-CP2475L (p220) | Core shell | 429 TPNITKLRDALSRIN |
| BA71V-CP2475L (P220) | BA71V-CP2475L (p220) | Core shell | 430 PNITKLRDALSRIND |
| BA71V-CP2475L (P220) | BA71V-CP2475L (p220) | Core shell | 431 NITKLRDALSRINDM |
| BA71V-CP2475L (P220) | BA71V-CP2475L (p220) | Core shell | 432 ITKLRDALSRINDMG |
| BA71V-CP2475L (P220) | BA71V-CP2475L (p220) | Core shell | 433 TKLRDALSRINDMGT |
| BA71V-CP2475L (P220) | BA71V-CP2475L (p220) | Core shell | 434 KLRDALSRINDMGTI |
| BA71V-CP2475L (P220) | BA71V-CP2475L (p220) | Core shell | 435 LRDALSRINDMGTIA |
| BA71V-CP2475L (P220) | BA71V-CP2475L (p220) | Core shell | 436 RDALSRINDMGTIAL |
| BA71V-CP2475L (P220) | BA71V-CP2475L (p220) | Core shell | 437 DALSRINDMGTIALD |
| BA71V-CP2475L (P220) | BA71V-CP2475L (p220) | Core shell | 438 ALSRINDMGTIALDL |
| BA71V-CP2475L (P220) | BA71V-CP2475L (p220) | Core shell | 439 LSRINDMGTIALDLS |
| BA71V-CP2475L (P220) | BA71V-CP2475L (p220) | Core shell | 440 SRINDMGTIALDLSL |
| BA71V-CP2475L (P220) | BA71V-CP2475L (p220) | Core shell | 441 RINDMGTIALDLSLI |
| BA71V-CP2475L (P220) | BA71V-CP2475L (p220) | Core shell | 442 INDMGTIALDLSLIG |
| BA71V-CP2475L (P220) | BA71V-CP2475L (p220) | Core shell | 443 NDMGTIALDLSLIGF |
| BA71V-CP2475L (P220) | BA71V-CP2475L (p220) | Core shell | 444 DMGTIALDLSLIGFY |
| BA71V-CP2475L (P220) | BA71V-CP2475L (p220) | Core shell | 445 MGTIALDLSLIGFYN |
| BA71V-CP2475L (P220) | BA71V-CP2475L (p220) | Core shell | 446 GTIALDLSLIGFYNN |
| BA71V-CP2475L (P220) | BA71V-CP2475L (p220) | Core shell | 447 TIALDLSLIGFYNNA |
| BA71V-CP2475L (P220) | BA71V-CP2475L (p220) | Core shell | 448 IALDLSLIGFYNNAA |
| BA71V-CP2475L (P220) | BA71V-CP2475L (p220) | Core shell | 449 ALDLSLIGFYNNAAA |
| BA71V-CP2475L (P220) | BA71V-CP2475L (p220) | Core shell | 450 LDLSLIGFYNNAAAR |
| BA71V-CP2475L (P220) | BA71V-CP2475L (p220) | Core shell | 451 DLSLIGFYNNAAARE |
| BA71V-CP2475L (P220) | BA71V-CP2475L (p220) | Core shell | 452 LSLIGFYNNAAAREE |
| BA71V-CP2475L (P220) | BA71V-CP2475L (p220) | Core shell | 453 SLIGFYNNAAAREER |
| BA71V-CP2475L (P220) | BA71V-CP2475L (p220) | Core shell | 454 LIGFYNNAAAREERE |
| BA71V-CP2475L (P220) | BA71V-CP2475L (p220) | Core shell | 455 IGFYNNAAAREERET |
| BA71V-CP2475L (P220) | BA71V-CP2475L (p220) | Core shell | 456 GFYNNAAAREERETF |
| BA71V-CP2475L (P220) | BA71V-CP2475L (p220) | Core shell | 457 FYNNAAAREERETFL |
| BA71V-CP2475L (P220) | BA71V-CP2475L (p220) | Core shell | 458 YNNAAAREERETFLI |
| BA71V-CP2475L (P220) | BA71V-CP2475L (p220) | Core shell | 459 NNAAAREERETFLIQ |
| BA71V-CP2475L (P220) | BA71V-CP2475L (p220) | Core shell | 460 NAAAREERETFLIQL |
| BA71V-CP2475L (P220) | BA71V-CP2475L (p220) | Core shell | 461 AAAREERETFLIQLT |
| BA71V-CP2475L (P220) | BA71V-CP2475L (p220) | Core shell | 462 AAREERETFLIQLTL |
| BA71V-CP2475L (P220) | BA71V-CP2475L (p220) | Core shell | 463 AREERETFLIQLTLV |
| BA71V-CP2475L (P220) | BA71V-CP2475L (p220) | Core shell | 464 REERETFLIQLTLVK |
| BA71V-CP2475L (P220) | BA71V-CP2475L (p220) | Core shell | 465 EERETFLIQLTLVKN |
| BA71V-CP2475L (P220) | BA71V-CP2475L (p220) | Core shell | 466 ERETFLIQLTLVKNV |
| BA71V-CP2475L (P220) | BA71V-CP2475L (p220) | Core shell | 467 RETFLIQLTLVKNVL |
| BA71V-CP2475L (P220) | BA71V-CP2475L (p220) | Core shell | 468 ETFLIQLTLVKNVLE |
| BA71V-CP2475L (P220) | BA71V-CP2475L (p220) | Core shell | 469 TFLIQLTLVKNVLEE |
| BA71V-CP2475L (P220) | BA71V-CP2475L (p220) | Core shell | 470 FLIQLTLVKNVLEEL |
| BA71V-CP2475L (P220) | BA71V-CP2475L (p220) | Core shell | 471 LIQLTLVKNVLEELA |
| BA71V-CP2475L (P220) | BA71V-CP2475L (p220) | Core shell | 472 IQLTLVKNVLEELAK |
| BA71V-CP2475L (P220) | BA71V-CP2475L (p220) | Core shell | 473 QLTLVKNVLEELAKT |
| BA71V-CP2475L (P220) | BA71V-CP2475L (p220) | Core shell | 474 LTLVKNVLEELAKTD |
| BA71V-CP2475L (P220) | BA71V-CP2475L (p220) | Core shell | 475 TLVKNVLEELAKTDP |
| BA71V-CP2475L (P220) | BA71V-CP2475L (p220) | Core shell | 476 LVKNVLEELAKTDPN |
| BA71V-CP2475L (P220) | BA71V-CP2475L (p220) | Core shell | 477 VKNVLEELAKTDPNF |
| BA71V-CP2475L (P220) | BA71V-CP2475L (p220) | Core shell | 478 KNVLEELAKTDPNFK |
| BA71V-CP2475L (P220) | BA71V-CP2475L (p220) | Core shell | 479 NVLEELAKTDPNFKN |
| BA71V-CP2475L (P220) | BA71V-CP2475L (p220) | Core shell | 480 VLEELAKTDPNFKNL |
| BA71V-CP2475L (P220) | BA71V-CP2475L (p220) | Core shell | 481 LEELAKTDPNFKNLY |
| BA71V-CP2475L (P220) | BA71V-CP2475L (p220) | Core shell | 482 EELAKTDPNFKNLYD |
| BA71V-CP2475L (P220) | BA71V-CP2475L (p220) | Core shell | 483 ELAKTDPNFKNLYDS |
| BA71V-CP2475L (P220) | BA71V-CP2475L (p220) | Core shell | 484 LAKTDPNFKNLYDSC |
| BA71V-CP2475L (P220) | BA71V-CP2475L (p220) | Core shell | 485 AKTDPNFKNLYDSCF |
| BA71V-CP2475L (P220) | BA71V-CP2475L (p220) | Core shell | 486 KTDPNFKNLYDSCFR |
| BA71V-CP2475L (P220) | BA71V-CP2475L (p220) | Core shell | 487 TDPNFKNLYDSCFRL |

| | | | |
|---|---|---|---|
| BA71V-CP2475L (P220) | BA71V-CP2475L (p220) | Core shell | 488 DPNFKNLYDSCFRLL |
| BA71V-CP2475L (P220) | BA71V-CP2475L (p220) | Core shell | 489 PNFKNLYDSCFRLLQ |
| BA71V-CP2475L (P220) | BA71V-CP2475L (p220) | Core shell | 490 NFKNLYDSCFRLLQI |
| BA71V-CP2475L (P220) | BA71V-CP2475L (p220) | Core shell | 491 FKNLYDSCFRLLQII |
| BA71V-CP2475L (P220) | BA71V-CP2475L (p220) | Core shell | 492 KNLYDSCFRLLQIID |
| BA71V-CP2475L (P220) | BA71V-CP2475L (p220) | Core shell | 493 NLYDSCFRLLQIIDF |
| BA71V-CP2475L (P220) | BA71V-CP2475L (p220) | Core shell | 494 LYDSCFRLLQIIDFY |
| BA71V-CP2475L (P220) | BA71V-CP2475L (p220) | Core shell | 495 YDSCFRLLQIIDFYT |
| BA71V-CP2475L (P220) | BA71V-CP2475L (p220) | Core shell | 496 DSCFRLLQIIDFYTD |
| BA71V-CP2475L (P220) | BA71V-CP2475L (p220) | Core shell | 497 SCFRLLQIIDFYTDI |
| BA71V-CP2475L (P220) | BA71V-CP2475L (p220) | Core shell | 498 CFRLLQIIDFYTDIV |
| BA71V-CP2475L (P220) | BA71V-CP2475L (p220) | Core shell | 499 FRLLQIIDFYTDIVQ |
| BA71V-CP2475L (P220) | BA71V-CP2475L (p220) | Core shell | 500 RLLQIIDFYTDIVQK |
| BA71V-CP2475L (P220) | BA71V-CP2475L (p220) | Core shell | 501 LLQIIDFYTDIVQKK |
| BA71V-CP2475L (P220) | BA71V-CP2475L (p220) | Core shell | 502 LQIIDFYTDIVQKKY |
| BA71V-CP2475L (P220) | BA71V-CP2475L (p220) | Core shell | 503 QIIDFYTDIVQKKYG |
| BA71V-CP2475L (P220) | BA71V-CP2475L (p220) | Core shell | 504 IIDFYTDIVQKKYGG |
| BA71V-CP2475L (P220) | BA71V-CP2475L (p220) | Core shell | 505 IDFYTDIVQKKYGGG |
| BA71V-CP2475L (P220) | BA71V-CP2475L (p220) | Core shell | 506 DFYTDIVQKKYGGGE |
| BA71V-CP2475L (P220) | BA71V-CP2475L (p220) | Core shell | 507 FYTDIVQKKYGGGED |
| BA71V-CP2475L (P220) | BA71V-CP2475L (p220) | Core shell | 508 YTDIVQKKYGGGEDC |
| BA71V-CP2475L (P220) | BA71V-CP2475L (p220) | Core shell | 509 TDIVQKKYGGGEDCE |
| BA71V-CP2475L (P220) | BA71V-CP2475L (p220) | Core shell | 510 DIVQKKYGGGEDCEC |
| BA71V-CP2475L (P220) | BA71V-CP2475L (p220) | Core shell | 511 IVQKKYGGGEDCECT |
| BA71V-CP2475L (P220) | BA71V-CP2475L (p220) | Core shell | 512 VQKKYGGGEDCECTK |
| BA71V-CP2475L (P220) | BA71V-CP2475L (p220) | Core shell | 513 QKKYGGGEDCECTKV |
| BA71V-CP2475L (P220) | BA71V-CP2475L (p220) | Core shell | 514 KKYGGGEDCECTKVG |
| BA71V-CP2475L (P220) | BA71V-CP2475L (p220) | Core shell | 515 KYGGGEDCECTKVGG |
| BA71V-CP2475L (P220) | BA71V-CP2475L (p220) | Core shell | 516 YGGGEDCECTKVGGA |
| BA71V-CP2475L (P220) | BA71V-CP2475L (p220) | Core shell | 517 GGGEDCECTKVGGAA |
| BA71V-CP2475L (P220) | BA71V-CP2475L (p220) | Core shell | 518 GGEDCECTKVGGAAL |
| BA71V-CP2475L (P220) | BA71V-CP2475L (p220) | Core shell | 519 GEDCECTKVGGAALT |
| BA71V-CP2475L (P220) | BA71V-CP2475L (p220) | Core shell | 520 EDCECTKVGGAALTV |
| BA71V-CP2475L (P220) | BA71V-CP2475L (p220) | Core shell | 521 DCECTKVGGAALTVE |
| BA71V-CP2475L (P220) | BA71V-CP2475L (p220) | Core shell | 522 CECTKVGGAALTVEE |
| BA71V-CP2475L (P220) | BA71V-CP2475L (p220) | Core shell | 523 ECTKVGGAALTVEEL |
| BA71V-CP2475L (P220) | BA71V-CP2475L (p220) | Core shell | 524 CTKVGGAALTVEELG |
| BA71V-CP2475L (P220) | BA71V-CP2475L (p220) | Core shell | 525 TKVGGAALTVEELGL |
| BA71V-CP2475L (P220) | BA71V-CP2475L (p220) | Core shell | 526 KVGGAALTVEELGLS |
| BA71V-CP2475L (P220) | BA71V-CP2475L (p220) | Core shell | 527 VGGAALTVEELGLSK |
| BA71V-CP2475L (P220) | BA71V-CP2475L (p220) | Core shell | 528 GGAALTVEELGLSKA |
| BA71V-CP2475L (P220) | BA71V-CP2475L (p220) | Core shell | 529 GAALTVEELGLSKAA |
| BA71V-CP2475L (P220) | BA71V-CP2475L (p220) | Core shell | 530 AALTVEELGLSKAAR |
| BA71V-CP2475L (P220) | BA71V-CP2475L (p220) | Core shell | 531 ALTVEELGLSKAARS |
| BA71V-CP2475L (P220) | BA71V-CP2475L (p220) | Core shell | 532 LTVEELGLSKAARSQ |
| BA71V-CP2475L (P220) | BA71V-CP2475L (p220) | Core shell | 533 TVEELGLSKAARSQV |
| BA71V-CP2475L (P220) | BA71V-CP2475L (p220) | Core shell | 534 VEELGLSKAARSQVD |
| BA71V-CP2475L (P220) | BA71V-CP2475L (p220) | Core shell | 535 EELGLSKAARSQVDL |
| BA71V-CP2475L (P220) | BA71V-CP2475L (p220) | Core shell | 536 ELGLSKAARSQVDLN |
| BA71V-CP2475L (P220) | BA71V-CP2475L (p220) | Core shell | 537 LGLSKAARSQVDLNQ |
| BA71V-CP2475L (P220) | BA71V-CP2475L (p220) | Core shell | 538 GLSKAARSQVDLNQA |
| BA71V-CP2475L (P220) | BA71V-CP2475L (p220) | Core shell | 539 LSKAARSQVDLNQAI |
| BA71V-CP2475L (P220) | BA71V-CP2475L (p220) | Core shell | 540 SKAARSQVDLNQAIN |
| BA71V-CP2475L (P220) | BA71V-CP2475L (p220) | Core shell | 541 KAARSQVDLNQAINT |
| BA71V-CP2475L (P220) | BA71V-CP2475L (p220) | Core shell | 542 AARSQVDLNQAINTF |
| BA71V-CP2475L (P220) | BA71V-CP2475L (p220) | Core shell | 543 ARSQVDLNQAINTFM |
| BA71V-CP2475L (P220) | BA71V-CP2475L (p220) | Core shell | 544 RSQVDLNQAINTFMY |
| BA71V-CP2475L (P220) | BA71V-CP2475L (p220) | Core shell | 545 SQVDLNQAINTFMYY |
| BA71V-CP2475L (P220) | BA71V-CP2475L (p220) | Core shell | 546 QVDLNQAINTFMYYY |
| BA71V-CP2475L (P220) | BA71V-CP2475L (p220) | Core shell | 547 VDLNQAINTFMYYYY |
| BA71V-CP2475L (P220) | BA71V-CP2475L (p220) | Core shell | 548 DLNQAINTFMYYYYV |
| BA71V-CP2475L (P220) | BA71V-CP2475L (p220) | Core shell | 549 LNQAINTFMYYYYVA |
| BA71V-CP2475L (P220) | BA71V-CP2475L (p220) | Core shell | 550 NQAINTFMYYYYVAQ |
| BA71V-CP2475L (P220) | BA71V-CP2475L (p220) | Core shell | 551 QAINTFMYYYYVAQI |
| BA71V-CP2475L (P220) | BA71V-CP2475L (p220) | Core shell | 552 AINTFMYYYYVAQIY |
| BA71V-CP2475L (P220) | BA71V-CP2475L (p220) | Core shell | 553 INTFMYYYYVAQIYS |
| BA71V-CP2475L (P220) | BA71V-CP2475L (p220) | Core shell | 554 NTFMYYYYVAQIYSN |

| | | | | |
|---|---|---|---|---|
| BA71V-CP2475L (P220) | BA71V-CP2475L (p220) | Core shell | 555 | TFMYYYYVAQIYSNL |
| BA71V-CP2475L (P220) | BA71V-CP2475L (p220) | Core shell | 556 | FMYYYYVAQIYSNLT |
| BA71V-CP2475L (P220) | BA71V-CP2475L (p220) | Core shell | 557 | MYYYYVAQIYSNLTH |
| BA71V-CP2475L (P220) | BA71V-CP2475L (p220) | Core shell | 558 | YYYYVAQIYSNLTHN |
| BA71V-CP2475L (P220) | BA71V-CP2475L (p220) | Core shell | 559 | YYYVAQIYSNLTHNK |
| BA71V-CP2475L (P220) | BA71V-CP2475L (p220) | Core shell | 560 | YYVAQIYSNLTHNKQ |
| BA71V-CP2475L (P220) | BA71V-CP2475L (p220) | Core shell | 561 | YVAQIYSNLTHNKQE |
| BA71V-CP2475L (P220) | BA71V-CP2475L (p220) | Core shell | 562 | VAQIYSNLTHNKQEF |
| BA71V-CP2475L (P220) | BA71V-CP2475L (p220) | Core shell | 563 | AQIYSNLTHNKQEFQ |
| BA71V-CP2475L (P220) | BA71V-CP2475L (p220) | Core shell | 564 | QIYSNLTHNKQEFQS |
| BA71V-CP2475L (P220) | BA71V-CP2475L (p220) | Core shell | 565 | IYSNLTHNKQEFQSY |
| BA71V-CP2475L (P220) | BA71V-CP2475L (p220) | Core shell | 566 | YSNLTHNKQEFQSYE |
| BA71V-CP2475L (P220) | BA71V-CP2475L (p220) | Core shell | 567 | SNLTHNKQEFQSYEE |
| BA71V-CP2475L (P220) | BA71V-CP2475L (p220) | Core shell | 568 | NLTHNKQEFQSYEEN |
| BA71V-CP2475L (P220) | BA71V-CP2475L (p220) | Core shell | 569 | LTHNKQEFQSYEENY |
| BA71V-CP2475L (P220) | BA71V-CP2475L (p220) | Core shell | 570 | THNKQEFQSYEENYA |
| BA71V-CP2475L (P220) | BA71V-CP2475L (p220) | Core shell | 571 | HNKQEFQSYEENYAT |
| BA71V-CP2475L (P220) | BA71V-CP2475L (p220) | Core shell | 572 | NKQEFQSYEENYATI |
| BA71V-CP2475L (P220) | BA71V-CP2475L (p220) | Core shell | 573 | KQEFQSYEENYATIL |
| BA71V-CP2475L (P220) | BA71V-CP2475L (p220) | Core shell | 574 | QEFQSYEENYATILG |
| BA71V-CP2475L (P220) | BA71V-CP2475L (p220) | Core shell | 575 | EFQSYEENYATILGD |
| BA71V-CP2475L (P220) | BA71V-CP2475L (p220) | Core shell | 576 | FQSYEENYATILGDA |
| BA71V-CP2475L (P220) | BA71V-CP2475L (p220) | Core shell | 577 | QSYEENYATILGDAI |
| BA71V-CP2475L (P220) | BA71V-CP2475L (p220) | Core shell | 578 | SYEENYATILGDAIA |
| BA71V-CP2475L (P220) | BA71V-CP2475L (p220) | Core shell | 579 | YEENYATILGDAIAG |
| BA71V-CP2475L (P220) | BA71V-CP2475L (p220) | Core shell | 580 | EENYATILGDAIAGR |
| BA71V-CP2475L (P220) | BA71V-CP2475L (p220) | Core shell | 581 | ENYATILGDAIAGRL |
| BA71V-CP2475L (P220) | BA71V-CP2475L (p220) | Core shell | 582 | NYATILGDAIAGRLM |
| BA71V-CP2475L (P220) | BA71V-CP2475L (p220) | Core shell | 583 | YATILGDAIAGRLMQ |
| BA71V-CP2475L (P220) | BA71V-CP2475L (p220) | Core shell | 584 | ATILGDAIAGRLMQL |
| BA71V-CP2475L (P220) | BA71V-CP2475L (p220) | Core shell | 585 | TILGDAIAGRLMQLD |
| BA71V-CP2475L (P220) | BA71V-CP2475L (p220) | Core shell | 586 | ILGDAIAGRLMQLDT |
| BA71V-CP2475L (P220) | BA71V-CP2475L (p220) | Core shell | 587 | LGDAIAGRLMQLDTE |
| BA71V-CP2475L (P220) | BA71V-CP2475L (p220) | Core shell | 588 | GDAIAGRLMQLDTEK |
| BA71V-CP2475L (P220) | BA71V-CP2475L (p220) | Core shell | 589 | DAIAGRLMQLDTEKN |
| BA71V-CP2475L (P220) | BA71V-CP2475L (p220) | Core shell | 590 | AIAGRLMQLDTEKNA |
| BA71V-CP2475L (P220) | BA71V-CP2475L (p220) | Core shell | 591 | IAGRLMQLDTEKNAR |
| BA71V-CP2475L (P220) | BA71V-CP2475L (p220) | Core shell | 592 | AGRLMQLDTEKNARI |
| BA71V-CP2475L (P220) | BA71V-CP2475L (p220) | Core shell | 593 | GRLMQLDTEKNARIN |
| BA71V-CP2475L (P220) | BA71V-CP2475L (p220) | Core shell | 594 | RLMQLDTEKNARINS |
| BA71V-CP2475L (P220) | BA71V-CP2475L (p220) | Core shell | 595 | LMQLDTEKNARINSP |
| BA71V-CP2475L (P220) | BA71V-CP2475L (p220) | Core shell | 596 | MQLDTEKNARINSPA |
| BA71V-CP2475L (P220) | BA71V-CP2475L (p220) | Core shell | 597 | QLDTEKNARINSPAV |
| BA71V-CP2475L (P220) | BA71V-CP2475L (p220) | Core shell | 598 | LDTEKNARINSPAVD |
| BA71V-CP2475L (P220) | BA71V-CP2475L (p220) | Core shell | 599 | DTEKNARINSPAVDL |
| BA71V-CP2475L (P220) | BA71V-CP2475L (p220) | Core shell | 600 | TEKNARINSPAVDLA |
| BA71V-CP2475L (P220) | BA71V-CP2475L (p220) | Core shell | 601 | EKNARINSPAVDLAR |
| BA71V-CP2475L (P220) | BA71V-CP2475L (p220) | Core shell | 602 | KNARINSPAVDLARG |
| BA71V-CP2475L (P220) | BA71V-CP2475L (p220) | Core shell | 603 | NARINSPAVDLARGH |
| BA71V-CP2475L (P220) | BA71V-CP2475L (p220) | Core shell | 604 | ARINSPAVDLARGHV |
| BA71V-CP2475L (P220) | BA71V-CP2475L (p220) | Core shell | 605 | RINSPAVDLARGHVG |
| BA71V-CP2475L (P220) | BA71V-CP2475L (p220) | Core shell | 606 | INSPAVDLARGHVGP |
| BA71V-CP2475L (P220) | BA71V-CP2475L (p220) | Core shell | 607 | NSPAVDLARGHVGPN |
| BA71V-CP2475L (P220) | BA71V-CP2475L (p220) | Core shell | 608 | SPAVDLARGHVGPNP |
| BA71V-CP2475L (P220) | BA71V-CP2475L (p220) | Core shell | 609 | PAVDLARGHVGPNPG |
| BA71V-CP2475L (P220) | BA71V-CP2475L (p220) | Core shell | 610 | AVDLARGHVGPNPGG |
| BA71V-CP2475L (P220) | BA71V-CP2475L (p220) | Core shell | 611 | VDLARGHVGPNPGGA |
| BA71V-CP2475L (P220) | BA71V-CP2475L (p220) | Core shell | 612 | DLARGHVGPNPGGAQ |
| BA71V-CP2475L (P220) | BA71V-CP2475L (p220) | Core shell | 613 | LARGHVGPNPGGAQE |
| BA71V-CP2475L (P220) | BA71V-CP2475L (p220) | Core shell | 614 | ARGHVGPNPGGAQEV |
| BA71V-CP2475L (P220) | BA71V-CP2475L (p220) | Core shell | 615 | RGHVGPNPGGAQEVD |
| BA71V-CP2475L (P220) | BA71V-CP2475L (p220) | Core shell | 616 | GHVGPNPGGAQEVDW |
| BA71V-CP2475L (P220) | BA71V-CP2475L (p220) | Core shell | 617 | HVGPNPGGAQEVDWK |
| BA71V-CP2475L (P220) | BA71V-CP2475L (p220) | Core shell | 618 | VGPNPGGAQEVDWKA |
| BA71V-CP2475L (P220) | BA71V-CP2475L (p220) | Core shell | 619 | GPNPGGAQEVDWKAT |
| BA71V-CP2475L (P220) | BA71V-CP2475L (p220) | Core shell | 620 | PNPGGAQEVDWKATI |
| BA71V-CP2475L (P220) | BA71V-CP2475L (p220) | Core shell | 621 | NPGGAQEVDWKATIS |

| | | | | |
|---|---|---|---|---|
| BA71V-CP2475L (P220) | BA71V-CP2475L (p220) | Core shell | 622 | PGGAQEVDWKATISA |
| BA71V-CP2475L (P220) | BA71V-CP2475L (p220) | Core shell | 623 | GGAQEVDWKATISAI |
| BA71V-CP2475L (P220) | BA71V-CP2475L (p220) | Core shell | 624 | GAQEVDWKATISAIE |
| BA71V-CP2475L (P220) | BA71V-CP2475L (p220) | Core shell | 625 | AQEVDWKATISAIEL |
| BA71V-CP2475L (P220) | BA71V-CP2475L (p220) | Core shell | 626 | QEVDWKATISAIELE |
| BA71V-CP2475L (P220) | BA71V-CP2475L (p220) | Core shell | 627 | EVDWKATISAIELEY |
| BA71V-CP2475L (P220) | BA71V-CP2475L (p220) | Core shell | 628 | VDWKATISAIELEYD |
| BA71V-CP2475L (P220) | BA71V-CP2475L (p220) | Core shell | 629 | DWKATISAIELEYDV |
| BA71V-CP2475L (P220) | BA71V-CP2475L (p220) | Core shell | 630 | WKATISAIELEYDVK |
| BA71V-CP2475L (P220) | BA71V-CP2475L (p220) | Core shell | 631 | KATISAIELEYDVKR |
| BA71V-CP2475L (P220) | BA71V-CP2475L (p220) | Core shell | 632 | ATISAIELEYDVKRR |
| BA71V-CP2475L (P220) | BA71V-CP2475L (p220) | Core shell | 633 | TISAIELEYDVKRRF |
| BA71V-CP2475L (P220) | BA71V-CP2475L (p220) | Core shell | 634 | ISAIELEYDVKRRFY |
| BA71V-CP2475L (P220) | BA71V-CP2475L (p220) | Core shell | 635 | SAIELEYDVKRRFYR |
| BA71V-CP2475L (P220) | BA71V-CP2475L (p220) | Core shell | 636 | AIELEYDVKRRFYRA |
| BA71V-CP2475L (P220) | BA71V-CP2475L (p220) | Core shell | 637 | IELEYDVKRRFYRAL |
| BA71V-CP2475L (P220) | BA71V-CP2475L (p220) | Core shell | 638 | ELEYDVKRRFYRALE |
| BA71V-CP2475L (P220) | BA71V-CP2475L (p220) | Core shell | 639 | LEYDVKRRFYRALEG |
| BA71V-CP2475L (P220) | BA71V-CP2475L (p220) | Core shell | 640 | EYDVKRRFYRALEGL |
| BA71V-CP2475L (P220) | BA71V-CP2475L (p220) | Core shell | 641 | YDVKRRFYRALEGLD |
| BA71V-CP2475L (P220) | BA71V-CP2475L (p220) | Core shell | 642 | DVKRRFYRALEGLDL |
| BA71V-CP2475L (P220) | BA71V-CP2475L (p220) | Core shell | 643 | VKRRFYRALEGLDLY |
| BA71V-CP2475L (P220) | BA71V-CP2475L (p220) | Core shell | 644 | KRRFYRALEGLDLYL |
| BA71V-CP2475L (P220) | BA71V-CP2475L (p220) | Core shell | 645 | RRFYRALEGLDLYLK |
| BA71V-CP2475L (P220) | BA71V-CP2475L (p220) | Core shell | 646 | RFYRALEGLDLYLKN |
| BA71V-CP2475L (P220) | BA71V-CP2475L (p220) | Core shell | 647 | FYRALEGLDLYLKNI |
| BA71V-CP2475L (P220) | BA71V-CP2475L (p220) | Core shell | 648 | YRALEGLDLYLKNIT |
| BA71V-CP2475L (P220) | BA71V-CP2475L (p220) | Core shell | 649 | RALEGLDLYLKNITK |
| BA71V-CP2475L (P220) | BA71V-CP2475L (p220) | Core shell | 650 | ALEGLDLYLKNITKT |
| BA71V-CP2475L (P220) | BA71V-CP2475L (p220) | Core shell | 651 | LEGLDLYLKNITKTF |
| BA71V-CP2475L (P220) | BA71V-CP2475L (p220) | Core shell | 652 | EGLDLYLKNITKTFV |
| BA71V-CP2475L (P220) | BA71V-CP2475L (p220) | Core shell | 653 | GLDLYLKNITKTFVN |
| BA71V-CP2475L (P220) | BA71V-CP2475L (p220) | Core shell | 654 | LDLYLKNITKTFVNN |
| BA71V-CP2475L (P220) | BA71V-CP2475L (p220) | Core shell | 655 | DLYLKNITKTFVNNI |
| BA71V-CP2475L (P220) | BA71V-CP2475L (p220) | Core shell | 656 | LYLKNITKTFVNNID |
| BA71V-CP2475L (P220) | BA71V-CP2475L (p220) | Core shell | 657 | YLKNITKTFVNNIDS |
| BA71V-CP2475L (P220) | BA71V-CP2475L (p220) | Core shell | 658 | LKNITKTFVNNIDSI |
| BA71V-CP2475L (P220) | BA71V-CP2475L (p220) | Core shell | 659 | KNITKTFVNNIDSIQ |
| BA71V-CP2475L (P220) | BA71V-CP2475L (p220) | Core shell | 660 | NITKTFVNNIDSIQT |
| BA71V-CP2475L (P220) | BA71V-CP2475L (p220) | Core shell | 661 | ITKTFVNNIDSIQTV |
| BA71V-CP2475L (P220) | BA71V-CP2475L (p220) | Core shell | 662 | TKTFVNNIDSIQTVQ |
| BA71V-CP2475L (P220) | BA71V-CP2475L (p220) | Core shell | 663 | KTFVNNIDSIQTVQQ |
| BA71V-CP2475L (P220) | BA71V-CP2475L (p220) | Core shell | 664 | TFVNNIDSIQTVQQM |
| BA71V-CP2475L (P220) | BA71V-CP2475L (p220) | Core shell | 665 | FVNNIDSIQTVQQML |
| BA71V-CP2475L (P220) | BA71V-CP2475L (p220) | Core shell | 666 | VNNIDSIQTVQQMLD |
| BA71V-CP2475L (P220) | BA71V-CP2475L (p220) | Core shell | 667 | NNIDSIQTVQQMLDG |
| BA71V-CP2475L (P220) | BA71V-CP2475L (p220) | Core shell | 668 | NIDSIQTVQQMLDGV |
| BA71V-CP2475L (P220) | BA71V-CP2475L (p220) | Core shell | 669 | IDSIQTVQQMLDGVR |
| BA71V-CP2475L (P220) | BA71V-CP2475L (p220) | Core shell | 670 | DSIQTVQQMLDGVRI |
| BA71V-CP2475L (P220) | BA71V-CP2475L (p220) | Core shell | 671 | SIQTVQQMLDGVRII |
| BA71V-CP2475L (P220) | BA71V-CP2475L (p220) | Core shell | 672 | IQTVQQMLDGVRIIG |
| BA71V-CP2475L (P220) | BA71V-CP2475L (p220) | Core shell | 673 | QTVQQMLDGVRIIGR |
| BA71V-CP2475L (P220) | BA71V-CP2475L (p220) | Core shell | 674 | TVQQMLDGVRIIGRW |
| BA71V-CP2475L (P220) | BA71V-CP2475L (p220) | Core shell | 675 | VQQMLDGVRIIGRWF |
| BA71V-CP2475L (P220) | BA71V-CP2475L (p220) | Core shell | 676 | QQMLDGVRIIGRWFT |
| BA71V-CP2475L (P220) | BA71V-CP2475L (p220) | Core shell | 677 | QMLDGVRIIGRWFTE |
| BA71V-CP2475L (P220) | BA71V-CP2475L (p220) | Core shell | 678 | MLDGVRIIGRWFTEA |
| BA71V-CP2475L (P220) | BA71V-CP2475L (p220) | Core shell | 679 | LDGVRIIGRWFTEAT |
| BA71V-CP2475L (P220) | BA71V-CP2475L (p220) | Core shell | 680 | DGVRIIGRWFTEATG |
| BA71V-CP2475L (P220) | BA71V-CP2475L (p220) | Core shell | 681 | GVRIIGRWFTEATGD |
| BA71V-CP2475L (P220) | BA71V-CP2475L (p220) | Core shell | 682 | VRIIGRWFTEATGDT |
| BA71V-CP2475L (P220) | BA71V-CP2475L (p220) | Core shell | 683 | RIIGRWFTEATGDTL |
| BA71V-CP2475L (P220) | BA71V-CP2475L (p220) | Core shell | 684 | IIGRWFTEATGDTLA |
| BA71V-CP2475L (P220) | BA71V-CP2475L (p220) | Core shell | 685 | IGRWFTEATGDTLAQ |
| BA71V-CP2475L (P220) | BA71V-CP2475L (p220) | Core shell | 686 | GRWFTEATGDTLAQV |
| BA71V-CP2475L (P220) | BA71V-CP2475L (p220) | Core shell | 687 | RWFTEATGDTLAQVF |
| BA71V-CP2475L (P220) | BA71V-CP2475L (p220) | Core shell | 688 | WFTEATGDTLAQVFE |

| | | | |
|---|---|---|---|
| BA71V-CP2475L (P220) | BA71V-CP2475L (p220) | Core shell | 689 FTEATGDTLAQVFES |
| BA71V-CP2475L (P220) | BA71V-CP2475L (p220) | Core shell | 690 TEATGDTLAQVFESF |
| BA71V-CP2475L (P220) | BA71V-CP2475L (p220) | Core shell | 691 EATGDTLAQVFESFP |
| BA71V-CP2475L (P220) | BA71V-CP2475L (p220) | Core shell | 692 ATGDTLAQVFESFPT |
| BA71V-CP2475L (P220) | BA71V-CP2475L (p220) | Core shell | 693 TGDTLAQVFESFPTS |
| BA71V-CP2475L (P220) | BA71V-CP2475L (p220) | Core shell | 694 GDTLAQVFESFPTSA |
| BA71V-CP2475L (P220) | BA71V-CP2475L (p220) | Core shell | 695 DTLAQVFESFPTSAG |
| BA71V-CP2475L (P220) | BA71V-CP2475L (p220) | Core shell | 696 TLAQVFESFPTSAGN |
| BA71V-CP2475L (P220) | BA71V-CP2475L (p220) | Core shell | 697 LAQVFESFPTSAGND |
| BA71V-CP2475L (P220) | BA71V-CP2475L (p220) | Core shell | 698 AQVFESFPTSAGNDS |
| BA71V-CP2475L (P220) | BA71V-CP2475L (p220) | Core shell | 699 QVFESFPTSAGNDSN |
| BA71V-CP2475L (P220) | BA71V-CP2475L (p220) | Core shell | 700 VFESFPTSAGNDSNV |
| BA71V-CP2475L (P220) | BA71V-CP2475L (p220) | Core shell | 701 FESFPTSAGNDSNVF |
| BA71V-CP2475L (P220) | BA71V-CP2475L (p220) | Core shell | 702 ESFPTSAGNDSNVFT |
| BA71V-CP2475L (P220) | BA71V-CP2475L (p220) | Core shell | 703 SFPTSAGNDSNVFTD |
| BA71V-CP2475L (P220) | BA71V-CP2475L (p220) | Core shell | 704 FPTSAGNDSNVFTDN |
| BA71V-CP2475L (P220) | BA71V-CP2475L (p220) | Core shell | 705 PTSAGNDSNVFTDNA |
| BA71V-CP2475L (P220) | BA71V-CP2475L (p220) | Core shell | 706 TSAGNDSNVFTDNAP |
| BA71V-CP2475L (P220) | BA71V-CP2475L (p220) | Core shell | 707 SAGNDSNVFTDNAPA |
| BA71V-CP2475L (P220) | BA71V-CP2475L (p220) | Core shell | 708 AGNDSNVFTDNAPAG |
| BA71V-CP2475L (P220) | BA71V-CP2475L (p220) | Core shell | 709 GNDSNVFTDNAPAGH |
| BA71V-CP2475L (P220) | BA71V-CP2475L (p220) | Core shell | 710 NDSNVFTDNAPAGHY |
| BA71V-CP2475L (P220) | BA71V-CP2475L (p220) | Core shell | 711 DSNVFTDNAPAGHYY |
| BA71V-CP2475L (P220) | BA71V-CP2475L (p220) | Core shell | 712 SNVFTDNAPAGHYYE |
| BA71V-CP2475L (P220) | BA71V-CP2475L (p220) | Core shell | 713 NVFTDNAPAGHYYEK |
| BA71V-CP2475L (P220) | BA71V-CP2475L (p220) | Core shell | 714 VFTDNAPAGHYYEKV |
| BA71V-CP2475L (P220) | BA71V-CP2475L (p220) | Core shell | 715 FTDNAPAGHYYEKVA |
| BA71V-CP2475L (P220) | BA71V-CP2475L (p220) | Core shell | 716 TDNAPAGHYYEKVAA |
| BA71V-CP2475L (P220) | BA71V-CP2475L (p220) | Core shell | 717 DNAPAGHYYEKVAAE |
| BA71V-CP2475L (P220) | BA71V-CP2475L (p220) | Core shell | 718 NAPAGHYYEKVAAEI |
| BA71V-CP2475L (P220) | BA71V-CP2475L (p220) | Core shell | 719 APAGHYYEKVAAEIQ |
| BA71V-CP2475L (P220) | BA71V-CP2475L (p220) | Core shell | 720 PAGHYYEKVAAEIQQ |
| BA71V-CP2475L (P220) | BA71V-CP2475L (p220) | Core shell | 721 AGHYYEKVAAEIQQG |
| BA71V-CP2475L (P220) | BA71V-CP2475L (p220) | Core shell | 722 GHYYEKVAAEIQQGR |
| BA71V-CP2475L (P220) | BA71V-CP2475L (p220) | Core shell | 723 HYYEKVAAEIQQGRG |
| BA71V-CP2475L (P220) | BA71V-CP2475L (p220) | Core shell | 724 YYEKVAAEIQQGRGV |
| BA71V-CP2475L (P220) | BA71V-CP2475L (p220) | Core shell | 725 YEKVAAEIQQGRGVG |
| BA71V-CP2475L (P220) | BA71V-CP2475L (p220) | Core shell | 726 EKVAAEIQQGRGVGT |
| BA71V-CP2475L (P220) | BA71V-CP2475L (p220) | Core shell | 727 KVAAEIQQGRGVGTL |
| BA71V-CP2475L (P220) | BA71V-CP2475L (p220) | Core shell | 728 VAAEIQQGRGVGTLR |
| BA71V-CP2475L (P220) | BA71V-CP2475L (p220) | Core shell | 729 AAEIQQGRGVGTLRP |
| BA71V-CP2475L (P220) | BA71V-CP2475L (p220) | Core shell | 730 AEIQQGRGVGTLRPV |
| BA71V-CP2475L (P220) | BA71V-CP2475L (p220) | Core shell | 731 EIQQGRGVGTLRPVR |
| BA71V-CP2475L (P220) | BA71V-CP2475L (p220) | Core shell | 732 IQQGRGVGTLRPVRA |
| BA71V-CP2475L (P220) | BA71V-CP2475L (p220) | Core shell | 733 QQGRGVGTLRPVRAS |
| BA71V-CP2475L (P220) | BA71V-CP2475L (p220) | Core shell | 734 QGRGVGTLRPVRASQ |
| BA71V-CP2475L (P220) | BA71V-CP2475L (p220) | Core shell | 735 GRGVGTLRPVRASQA |
| BA71V-CP2475L (P220) | BA71V-CP2475L (p220) | Core shell | 736 RGVGTLRPVRASQAK |
| BA71V-CP2475L (P220) | BA71V-CP2475L (p220) | Core shell | 737 GVGTLRPVRASQAKN |
| BA71V-CP2475L (P220) | BA71V-CP2475L (p220) | Core shell | 738 VGTLRPVRASQAKNI |
| BA71V-CP2475L (P220) | BA71V-CP2475L (p220) | Core shell | 739 GTLRPVRASQAKNIR |
| BA71V-CP2475L (P220) | BA71V-CP2475L (p220) | Core shell | 740 TLRPVRASQAKNIRD |
| BA71V-CP2475L (P220) | BA71V-CP2475L (p220) | Core shell | 741 LRPVRASQAKNIRDL |
| BA71V-CP2475L (P220) | BA71V-CP2475L (p220) | Core shell | 742 RPVRASQAKNIRDLI |
| BA71V-CP2475L (P220) | BA71V-CP2475L (p220) | Core shell | 743 PVRASQAKNIRDLIG |
| BA71V-CP2475L (P220) | BA71V-CP2475L (p220) | Core shell | 744 VRASQAKNIRDLIGR |
| BA71V-CP2475L (P220) | BA71V-CP2475L (p220) | Core shell | 745 RASQAKNIRDLIGRS |
| BA71V-CP2475L (P220) | BA71V-CP2475L (p220) | Core shell | 746 ASQAKNIRDLIGRSL |
| BA71V-CP2475L (P220) | BA71V-CP2475L (p220) | Core shell | 747 SQAKNIRDLIGRSLS |
| BA71V-CP2475L (P220) | BA71V-CP2475L (p220) | Core shell | 748 QAKNIRDLIGRSLSN |
| BA71V-CP2475L (P220) | BA71V-CP2475L (p220) | Core shell | 749 AKNIRDLIGRSLSNF |
| BA71V-CP2475L (P220) | BA71V-CP2475L (p220) | Core shell | 750 KNIRDLIGRSLSNFQ |
| BA71V-CP2475L (P220) | BA71V-CP2475L (p220) | Core shell | 751 NIRDLIGRSLSNFQA |
| BA71V-CP2475L (P220) | BA71V-CP2475L (p220) | Core shell | 752 IRDLIGRSLSNFQAL |
| BA71V-CP2475L (P220) | BA71V-CP2475L (p220) | Core shell | 753 RDLIGRSLSNFQALK |
| BA71V-CP2475L (P220) | BA71V-CP2475L (p220) | Core shell | 754 DLIGRSLSNFQALKN |
| BA71V-CP2475L (P220) | BA71V-CP2475L (p220) | Core shell | 755 LIGRSLSNFQALKNI |

| | | | | |
|---|---|---|---|---|
| BA71V-CP2475L (P220) | BA71V-CP2475L (p220) | Core shell | 756 | IGRSLSNFQALKNII |
| BA71V-CP2475L (P220) | BA71V-CP2475L (p220) | Core shell | 757 | GRSLSNFQALKNIIN |
| BA71V-CP2475L (P220) | BA71V-CP2475L (p220) | Core shell | 758 | RSLSNFQALKNIINA |
| BA71V-CP2475L (P220) | BA71V-CP2475L (p220) | Core shell | 759 | SLSNFQALKNIINAF |
| BA71V-CP2475L (P220) | BA71V-CP2475L (p220) | Core shell | 760 | LSNFQALKNIINAFA |
| BA71V-CP2475L (P220) | BA71V-CP2475L (p220) | Core shell | 761 | SNFQALKNIINAFAR |
| BA71V-CP2475L (P220) | BA71V-CP2475L (p220) | Core shell | 762 | NFQALKNIINAFARI |
| BA71V-CP2475L (P220) | BA71V-CP2475L (p220) | Core shell | 763 | FQALKNIINAFARIG |
| BA71V-CP2475L (P220) | BA71V-CP2475L (p220) | Core shell | 764 | QALKNIINAFARIGD |
| BA71V-CP2475L (P220) | BA71V-CP2475L (p220) | Core shell | 765 | ALKNIINAFARIGDM |
| BA71V-CP2475L (P220) | BA71V-CP2475L (p220) | Core shell | 766 | LKNIINAFARIGDML |
| BA71V-CP2475L (P220) | BA71V-CP2475L (p220) | Core shell | 767 | KNIINAFARIGDMLG |
| BA71V-CP2475L (P220) | BA71V-CP2475L (p220) | Core shell | 768 | NIINAFARIGDMLGG |
| BA71V-CP2475L (P220) | BA71V-CP2475L (p220) | Core shell | 769 | IINAFARIGDMLGGE |
| BA71V-CP2475L (P220) | BA71V-CP2475L (p220) | Core shell | 770 | INAFARIGDMLGGEE |
| BA71V-CP2475L (P220) | BA71V-CP2475L (p220) | Core shell | 771 | NAFARIGDMLGGEEL |
| BA71V-CP2475L (P220) | BA71V-CP2475L (p220) | Core shell | 772 | AFARIGDMLGGEELR |
| BA71V-CP2475L (P220) | BA71V-CP2475L (p220) | Core shell | 773 | FARIGDMLGGEELRQ |
| BA71V-CP2475L (P220) | BA71V-CP2475L (p220) | Core shell | 774 | ARIGDMLGGEELRQT |
| BA71V-CP2475L (P220) | BA71V-CP2475L (p220) | Core shell | 775 | RIGDMLGGEELRQTV |
| BA71V-CP2475L (P220) | BA71V-CP2475L (p220) | Core shell | 776 | IGDMLGGEELRQTVP |
| BA71V-CP2475L (P220) | BA71V-CP2475L (p220) | Core shell | 777 | GDMLGGEELRQTVPM |
| BA71V-CP2475L (P220) | BA71V-CP2475L (p220) | Core shell | 778 | DMLGGEELRQTVPMS |
| BA71V-CP2475L (P220) | BA71V-CP2475L (p220) | Core shell | 779 | MLGGEELRQTVPMSP |
| BA71V-CP2475L (P220) | BA71V-CP2475L (p220) | Core shell | 780 | LGGEELRQTVPMSPL |
| BA71V-CP2475L (P220) | BA71V-CP2475L (p220) | Core shell | 781 | GGEELRQTVPMSPLQ |
| BA71V-CP2475L (P220) | BA71V-CP2475L (p220) | Core shell | 782 | GEELRQTVPMSPLQI |
| BA71V-CP2475L (P220) | BA71V-CP2475L (p220) | Core shell | 783 | EELRQTVPMSPLQIY |
| BA71V-CP2475L (P220) | BA71V-CP2475L (p220) | Core shell | 784 | ELRQTVPMSPLQIYK |
| BA71V-CP2475L (P220) | BA71V-CP2475L (p220) | Core shell | 785 | LRQTVPMSPLQIYKT |
| BA71V-CP2475L (P220) | BA71V-CP2475L (p220) | Core shell | 786 | RQTVPMSPLQIYKTL |
| BA71V-CP2475L (P220) | BA71V-CP2475L (p220) | Core shell | 787 | QTVPMSPLQIYKTLL |
| BA71V-CP2475L (P220) | BA71V-CP2475L (p220) | Core shell | 788 | TVPMSPLQIYKTLLE |
| BA71V-CP2475L (P220) | BA71V-CP2475L (p220) | Core shell | 789 | VPMSPLQIYKTLLEY |
| BA71V-CP2475L (P220) | BA71V-CP2475L (p220) | Core shell | 790 | PMSPLQIYKTLLEYI |
| BA71V-CP2475L (P220) | BA71V-CP2475L (p220) | Core shell | 791 | MSPLQIYKTLLEYIQ |
| BA71V-CP2475L (P220) | BA71V-CP2475L (p220) | Core shell | 792 | SPLQIYKTLLEYIQH |
| BA71V-CP2475L (P220) | BA71V-CP2475L (p220) | Core shell | 793 | PLQIYKTLLEYIQHS |
| BA71V-CP2475L (P220) | BA71V-CP2475L (p220) | Core shell | 794 | LQIYKTLLEYIQHSA |
| BA71V-CP2475L (P220) | BA71V-CP2475L (p220) | Core shell | 795 | QIYKTLLEYIQHSAL |
| BA71V-CP2475L (P220) | BA71V-CP2475L (p220) | Core shell | 796 | IYKTLLEYIQHSALS |
| BA71V-CP2475L (P220) | BA71V-CP2475L (p220) | Core shell | 797 | YKTLLEYIQHSALSV |
| BA71V-CP2475L (P220) | BA71V-CP2475L (p220) | Core shell | 798 | KTLLEYIQHSALSVG |
| BA71V-CP2475L (P220) | BA71V-CP2475L (p220) | Core shell | 799 | TLLEYIQHSALSVGL |
| BA71V-CP2475L (P220) | BA71V-CP2475L (p220) | Core shell | 800 | LLEYIQHSALSVGLK |
| BA71V-CP2475L (P220) | BA71V-CP2475L (p220) | Core shell | 801 | LEYIQHSALSVGLKN |
| BA71V-CP2475L (P220) | BA71V-CP2475L (p220) | Core shell | 802 | EYIQHSALSVGLKNL |
| BA71V-CP2475L (P220) | BA71V-CP2475L (p220) | Core shell | 803 | YIQHSALSVGLKNLN |
| BA71V-CP2475L (P220) | BA71V-CP2475L (p220) | Core shell | 804 | IQHSALSVGLKNLNQ |
| BA71V-CP2475L (P220) | BA71V-CP2475L (p220) | Core shell | 805 | QHSALSVGLKNLNQT |
| BA71V-CP2475L (P220) | BA71V-CP2475L (p220) | Core shell | 806 | HSALSVGLKNLNQTQ |
| BA71V-CP2475L (P220) | BA71V-CP2475L (p220) | Core shell | 807 | SALSVGLKNLNQTQI |
| BA71V-CP2475L (P220) | BA71V-CP2475L (p220) | Core shell | 808 | ALSVGLKNLNQTQIG |
| BA71V-CP2475L (P220) | BA71V-CP2475L (p220) | Core shell | 809 | LSVGLKNLNQTQIGG |
| BA71V-CP2475L (P220) | BA71V-CP2475L (p220) | Core shell | 810 | SVGLKNLNQTQIGGQ |
| BA71V-CP2475L (P220) | BA71V-CP2475L (p220) | Core shell | 811 | VGLKNLNQTQIGGQR |
| BA71V-CP2475L (P220) | BA71V-CP2475L (p220) | Core shell | 812 | GLKNLNQTQIGGQRV |
| BA71V-CP2475L (P220) | BA71V-CP2475L (p220) | Core shell | 813 | LKNLNQTQIGGQRVA |
| BA71V-CP2475L (P220) | BA71V-CP2475L (p220) | Core shell | 814 | KNLNQTQIGGQRVAL |
| BA71V-CP2475L (P220) | BA71V-CP2475L (p220) | Core shell | 815 | NLNQTQIGGQRVALA |
| BA71V-CP2475L (P220) | BA71V-CP2475L (p220) | Core shell | 816 | LNQTQIGGQRVALAQ |
| BA71V-CP2475L (P220) | BA71V-CP2475L (p220) | Core shell | 817 | NQTQIGGQRVALAQT |
| BA71V-CP2475L (P220) | BA71V-CP2475L (p220) | Core shell | 818 | QTQIGGQRVALAQTA |
| BA71V-CP2475L (P220) | BA71V-CP2475L (p220) | Core shell | 819 | TQIGGQRVALAQTAE |
| BA71V-CP2475L (P220) | BA71V-CP2475L (p220) | Core shell | 820 | QIGGQRVALAQTAEE |
| BA71V-CP2475L (P220) | BA71V-CP2475L (p220) | Core shell | 821 | IGGQRVALAQTAEEA |
| BA71V-CP2475L (P220) | BA71V-CP2475L (p220) | Core shell | 822 | GGQRVALAQTAEEAS |

| | | | | |
|---|---|---|---|---|
| BA71V-CP2475L (P220) | BA71V-CP2475L (p220) | Core shell | 823 | GQRVALAQTAEEASQ |
| BA71V-CP2475L (P220) | BA71V-CP2475L (p220) | Core shell | 824 | QRVALAQTAEEASQR |
| BA71V-CP2475L (P220) | BA71V-CP2475L (p220) | Core shell | 825 | RVALAQTAEEASQRV |
| BA71V-CP2475L (P220) | BA71V-CP2475L (p220) | Core shell | 826 | VALAQTAEEASQRVY |
| BA71V-CP2475L (P220) | BA71V-CP2475L (p220) | Core shell | 827 | ALAQTAEEASQRVYL |
| BA71V-CP2475L (P220) | BA71V-CP2475L (p220) | Core shell | 828 | LAQTAEEASQRVYLS |
| BA71V-CP2475L (P220) | BA71V-CP2475L (p220) | Core shell | 829 | AQTAEEASQRVYLST |
| BA71V-CP2475L (P220) | BA71V-CP2475L (p220) | Core shell | 830 | QTAEEASQRVYLSTV |
| BA71V-CP2475L (P220) | BA71V-CP2475L (p220) | Core shell | 831 | TAEEASQRVYLSTVR |
| BA71V-CP2475L (P220) | BA71V-CP2475L (p220) | Core shell | 832 | AEEASQRVYLSTVRV |
| BA71V-CP2475L (P220) | BA71V-CP2475L (p220) | Core shell | 833 | EEASQRVYLSTVRVN |
| BA71V-CP2475L (P220) | BA71V-CP2475L (p220) | Core shell | 834 | EASQRVYLSTVRVND |
| BA71V-CP2475L (P220) | BA71V-CP2475L (p220) | Core shell | 835 | ASQRVYLSTVRVNDA |
| BA71V-CP2475L (P220) | BA71V-CP2475L (p220) | Core shell | 836 | SQRVYLSTVRVNDAL |
| BA71V-CP2475L (P220) | BA71V-CP2475L (p220) | Core shell | 837 | QRVYLSTVRVNDALS |
| BA71V-CP2475L (P220) | BA71V-CP2475L (p220) | Core shell | 838 | RVYLSTVRVNDALST |
| BA71V-CP2475L (P220) | BA71V-CP2475L (p220) | Core shell | 839 | VYLSTVRVNDALSTR |
| BA71V-CP2475L (P220) | BA71V-CP2475L (p220) | Core shell | 840 | YLSTVRVNDALSTRW |
| BA71V-CP2475L (P220) | BA71V-CP2475L (p220) | Core shell | 841 | LSTVRVNDALSTRWE |
| BA71V-CP2475L (P220) | BA71V-CP2475L (p220) | Core shell | 842 | STVRVNDALSTRWET |
| BA71V-CP2475L (P220) | BA71V-CP2475L (p220) | Core shell | 843 | TVRVNDALSTRWETE |
| BA71V-CP2475L (P220) | BA71V-CP2475L (p220) | Core shell | 844 | VRVNDALSTRWETED |
| BA71V-CP2475L (P220) | BA71V-CP2475L (p220) | Core shell | 845 | RVNDALSTRWETEDV |
| BA71V-CP2475L (P220) | BA71V-CP2475L (p220) | Core shell | 846 | VNDALSTRWETEDVF |
| BA71V-CP2475L (P220) | BA71V-CP2475L (p220) | Core shell | 847 | NDALSTRWETEDVFF |
| BA71V-CP2475L (P220) | BA71V-CP2475L (p220) | Core shell | 848 | DALSTRWETEDVFFT |
| BA71V-CP2475L (P220) | BA71V-CP2475L (p220) | Core shell | 849 | ALSTRWETEDVFFTF |
| BA71V-CP2475L (P220) | BA71V-CP2475L (p220) | Core shell | 850 | LSTRWETEDVFFTFM |
| BA71V-CP2475L (P220) | BA71V-CP2475L (p220) | Core shell | 851 | STRWETEDVFFTFML |
| BA71V-CP2475L (P220) | BA71V-CP2475L (p220) | Core shell | 852 | TRWETEDVFFTFMLK |
| BA71V-CP2475L (P220) | BA71V-CP2475L (p220) | Core shell | 853 | RWETEDVFFTFMLKS |
| BA71V-CP2475L (P220) | BA71V-CP2475L (p220) | Core shell | 854 | WETEDVFFTFMLKSM |
| BA71V-CP2475L (P220) | BA71V-CP2475L (p220) | Core shell | 855 | ETEDVFFTFMLKSMA |
| BA71V-CP2475L (P220) | BA71V-CP2475L (p220) | Core shell | 856 | TEDVFFTFMLKSMAA |
| BA71V-CP2475L (P220) | BA71V-CP2475L (p220) | Core shell | 857 | EDVFFTFMLKSMAAK |
| BA71V-CP2475L (P220) | BA71V-CP2475L (p220) | Core shell | 858 | DVFFTFMLKSMAAKI |
| BA71V-CP2475L (P220) | BA71V-CP2475L (p220) | Core shell | 859 | VFFTFMLKSMAAKIF |
| BA71V-CP2475L (P220) | BA71V-CP2475L (p220) | Core shell | 860 | FFTFMLKSMAAKIFI |
| BA71V-CP2475L (P220) | BA71V-CP2475L (p220) | Core shell | 861 | FTFMLKSMAAKIFIV |
| BA71V-CP2475L (P220) | BA71V-CP2475L (p220) | Core shell | 862 | TFMLKSMAAKIFIVL |
| BA71V-CP2475L (P220) | BA71V-CP2475L (p220) | Core shell | 863 | FMLKSMAAKIFIVLG |
| BA71V-CP2475L (P220) | BA71V-CP2475L (p220) | Core shell | 864 | MLKSMAAKIFIVLGI |
| BA71V-CP2475L (P220) | BA71V-CP2475L (p220) | Core shell | 865 | LKSMAAKIFIVLGIY |
| BA71V-CP2475L (P220) | BA71V-CP2475L (p220) | Core shell | 866 | KSMAAKIFIVLGIYD |
| BA71V-CP2475L (P220) | BA71V-CP2475L (p220) | Core shell | 867 | SMAAKIFIVLGIYDM |
| BA71V-CP2475L (P220) | BA71V-CP2475L (p220) | Core shell | 868 | MAAKIFIVLGIYDMF |
| BA71V-CP2475L (P220) | BA71V-CP2475L (p220) | Core shell | 869 | AAKIFIVLGIYDMFE |
| BA71V-CP2475L (P220) | BA71V-CP2475L (p220) | Core shell | 870 | AKIFIVLGIYDMFER |
| BA71V-CP2475L (P220) | BA71V-CP2475L (p220) | Core shell | 871 | KIFIVLGIYDMFERP |
| BA71V-CP2475L (P220) | BA71V-CP2475L (p220) | Core shell | 872 | IFIVLGIYDMFERPE |
| BA71V-CP2475L (P220) | BA71V-CP2475L (p220) | Core shell | 873 | FIVLGIYDMFERPEP |
| BA71V-CP2475L (P220) | BA71V-CP2475L (p220) | Core shell | 874 | IVLGIYDMFERPEPV |
| BA71V-CP2475L (P220) | BA71V-CP2475L (p220) | Core shell | 875 | VLGIYDMFERPEPVY |
| BA71V-CP2475L (P220) | BA71V-CP2475L (p220) | Core shell | 876 | LGIYDMFERPEPVYK |
| BA71V-CP2475L (P220) | BA71V-CP2475L (p220) | Core shell | 877 | GIYDMFERPEPVYKL |
| BA71V-CP2475L (P220) | BA71V-CP2475L (p220) | Core shell | 878 | IYDMFERPEPVYKLI |
| BA71V-CP2475L (P220) | BA71V-CP2475L (p220) | Core shell | 879 | YDMFERPEPVYKLIP |
| BA71V-CP2475L (P220) | BA71V-CP2475L (p220) | Core shell | 880 | DMFERPEPVYKLIPT |
| BA71V-CP2475L (P220) | BA71V-CP2475L (p220) | Core shell | 881 | MFERPEPVYKLIPTR |
| BA71V-CP2475L (P220) | BA71V-CP2475L (p220) | Core shell | 882 | FERPEPVYKLIPTRM |
| BA71V-CP2475L (P220) | BA71V-CP2475L (p220) | Core shell | 883 | ERPEPVYKLIPTRMI |
| BA71V-CP2475L (P220) | BA71V-CP2475L (p220) | Core shell | 884 | RPEPVYKLIPTRMIL |
| BA71V-CP2475L (P220) | BA71V-CP2475L (p220) | Core shell | 885 | PEPVYKLIPTRMILG |
| BA71V-CP2475L (P220) | BA71V-CP2475L (p220) | Core shell | 886 | EPVYKLIPTRMILGG |
| BA71V-CP2475L (P220) | BA71V-CP2475L (p220) | Core shell | 887 | PVYKLIPTRMILGGA |
| BA71V-CP2475L (P220) | BA71V-CP2475L (p220) | Core shell | 888 | VYKLIPTRMILGGAD |
| BA71V-CP2475L (P220) | BA71V-CP2475L (p220) | Core shell | 889 | YKLIPTRMILGGADE |

| | | | | |
|---|---|---|---|---|
| BA71V-CP2475L (P220) | BA71V-CP2475L (p220) | Core shell | 890 | KLIPTRMILGGADEL |
| BA71V-CP2475L (P220) | BA71V-CP2475L (p220) | Core shell | 891 | LIPTRMILGGADELE |
| BA71V-CP2475L (P220) | BA71V-CP2475L (p220) | Core shell | 892 | IPTRMILGGADELEP |
| BA71V-CP2475L (P220) | BA71V-CP2475L (p220) | Core shell | 893 | PTRMILGGADELEPE |
| BA71V-CP2475L (P220) | BA71V-CP2475L (p220) | Core shell | 894 | TRMILGGADELEPEV |
| BA71V-CP2475L (P220) | BA71V-CP2475L (p220) | Core shell | 895 | RMILGGADELEPEVI |
| BA71V-CP2475L (P220) | BA71V-CP2475L (p220) | Core shell | 896 | MILGGADELEPEVIP |
| BA71V-CP2475L (P220) | BA71V-CP2475L (p220) | Core shell | 897 | ILGGADELEPEVIPE |
| BA71V-CP2475L (P220) | BA71V-CP2475L (p220) | Core shell | 898 | LGGADELEPEVIPEA |
| BA71V-CP2475L (P220) | BA71V-CP2475L (p220) | Core shell | 899 | GGADELEPEVIPEAA |
| BA71V-CP2475L (P220) | BA71V-CP2475L (p220) | Core shell | 900 | GADELEPEVIPEAAG |
| BA71V-CP2475L (P220) | BA71V-CP2475L (p220) | Core shell | 901 | ADELEPEVIPEAAGL |
| BA71V-CP2475L (P220) | BA71V-CP2475L (p220) | Core shell | 902 | DELEPEVIPEAAGLY |
| BA71V-CP2475L (P220) | BA71V-CP2475L (p220) | Core shell | 903 | ELEPEVIPEAAGLYF |
| BA71V-CP2475L (P220) | BA71V-CP2475L (p220) | Core shell | 904 | LEPEVIPEAAGLYFR |
| BA71V-CP2475L (P220) | BA71V-CP2475L (p220) | Core shell | 905 | EPEVIPEAAGLYFRL |
| BA71V-CP2475L (P220) | BA71V-CP2475L (p220) | Core shell | 906 | PEVIPEAAGLYFRLP |
| BA71V-CP2475L (P220) | BA71V-CP2475L (p220) | Core shell | 907 | EVIPEAAGLYFRLPR |
| BA71V-CP2475L (P220) | BA71V-CP2475L (p220) | Core shell | 908 | VIPEAAGLYFRLPRL |
| BA71V-CP2475L (P220) | BA71V-CP2475L (p220) | Core shell | 909 | IPEAAGLYFRLPRLA |
| BA71V-CP2475L (P220) | BA71V-CP2475L (p220) | Core shell | 910 | PEAAGLYFRLPRLAE |
| BA71V-CP2475L (P220) | BA71V-CP2475L (p220) | Core shell | 911 | EAAGLYFRLPRLAEF |
| BA71V-CP2475L (P220) | BA71V-CP2475L (p220) | Core shell | 912 | AAGLYFRLPRLAEFY |
| BA71V-CP2475L (P220) | BA71V-CP2475L (p220) | Core shell | 913 | AGLYFRLPRLAEFYQ |
| BA71V-CP2475L (P220) | BA71V-CP2475L (p220) | Core shell | 914 | GLYFRLPRLAEFYQK |
| BA71V-CP2475L (P220) | BA71V-CP2475L (p220) | Core shell | 915 | LYFRLPRLAEFYQKL |
| BA71V-CP2475L (P220) | BA71V-CP2475L (p220) | Core shell | 916 | YFRLPRLAEFYQKLF |
| BA71V-CP2475L (P220) | BA71V-CP2475L (p220) | Core shell | 917 | FRLPRLAEFYQKLFS |
| BA71V-CP2475L (P220) | BA71V-CP2475L (p220) | Core shell | 918 | RLPRLAEFYQKLFSF |
| BA71V-CP2475L (P220) | BA71V-CP2475L (p220) | Core shell | 919 | LPRLAEFYQKLFSFR |
| BA71V-CP2475L (P220) | BA71V-CP2475L (p220) | Core shell | 920 | PRLAEFYQKLFSFRD |
| BA71V-CP2475L (P220) | BA71V-CP2475L (p220) | Core shell | 921 | RLAEFYQKLFSFRDE |
| BA71V-CP2475L (P220) | BA71V-CP2475L (p220) | Core shell | 922 | LAEFYQKLFSFRDEN |
| BA71V-CP2475L (P220) | BA71V-CP2475L (p220) | Core shell | 923 | AEFYQKLFSFRDENV |
| BA71V-CP2475L (P220) | BA71V-CP2475L (p220) | Core shell | 924 | EFYQKLFSFRDENVQ |
| BA71V-CP2475L (P220) | BA71V-CP2475L (p220) | Core shell | 925 | FYQKLFSFRDENVQI |
| BA71V-CP2475L (P220) | BA71V-CP2475L (p220) | Core shell | 926 | YQKLFSFRDENVQIS |
| BA71V-CP2475L (P220) | BA71V-CP2475L (p220) | Core shell | 927 | QKLFSFRDENVQISM |
| BA71V-CP2475L (P220) | BA71V-CP2475L (p220) | Core shell | 928 | KLFSFRDENVQISML |
| BA71V-CP2475L (P220) | BA71V-CP2475L (p220) | Core shell | 929 | LFSFRDENVQISMLP |
| BA71V-CP2475L (P220) | BA71V-CP2475L (p220) | Core shell | 930 | FSFRDENVQISMLPE |
| BA71V-CP2475L (P220) | BA71V-CP2475L (p220) | Core shell | 931 | SFRDENVQISMLPEL |
| BA71V-CP2475L (P220) | BA71V-CP2475L (p220) | Core shell | 932 | FRDENVQISMLPELE |
| BA71V-CP2475L (P220) | BA71V-CP2475L (p220) | Core shell | 933 | RDENVQISMLPELEG |
| BA71V-CP2475L (P220) | BA71V-CP2475L (p220) | Core shell | 934 | DENVQISMLPELEGI |
| BA71V-CP2475L (P220) | BA71V-CP2475L (p220) | Core shell | 935 | ENVQISMLPELEGIF |
| BA71V-CP2475L (P220) | BA71V-CP2475L (p220) | Core shell | 936 | NVQISMLPELEGIFS |
| BA71V-CP2475L (P220) | BA71V-CP2475L (p220) | Core shell | 937 | VQISMLPELEGIFSG |
| BA71V-CP2475L (P220) | BA71V-CP2475L (p220) | Core shell | 938 | QISMLPELEGIFSGL |
| BA71V-CP2475L (P220) | BA71V-CP2475L (p220) | Core shell | 939 | ISMLPELEGIFSGLI |
| BA71V-CP2475L (P220) | BA71V-CP2475L (p220) | Core shell | 940 | SMLPELEGIFSGLIR |
| BA71V-CP2475L (P220) | BA71V-CP2475L (p220) | Core shell | 941 | MLPELEGIFSGLIRV |
| BA71V-CP2475L (P220) | BA71V-CP2475L (p220) | Core shell | 942 | LPELEGIFSGLIRVI |
| BA71V-CP2475L (P220) | BA71V-CP2475L (p220) | Core shell | 943 | PELEGIFSGLIRVIF |
| BA71V-CP2475L (P220) | BA71V-CP2475L (p220) | Core shell | 944 | ELEGIFSGLIRVIFM |
| BA71V-CP2475L (P220) | BA71V-CP2475L (p220) | Core shell | 945 | LEGIFSGLIRVIFMR |
| BA71V-CP2475L (P220) | BA71V-CP2475L (p220) | Core shell | 946 | EGIFSGLIRVIFMRP |
| BA71V-CP2475L (P220) | BA71V-CP2475L (p220) | Core shell | 947 | GIFSGLIRVIFMRPI |
| BA71V-CP2475L (P220) | BA71V-CP2475L (p220) | Core shell | 948 | IFSGLIRVIFMRPIE |
| BA71V-CP2475L (P220) | BA71V-CP2475L (p220) | Core shell | 949 | FSGLIRVIFMRPIEL |
| BA71V-CP2475L (P220) | BA71V-CP2475L (p220) | Core shell | 950 | SGLIRVIFMRPIELI |
| BA71V-CP2475L (P220) | BA71V-CP2475L (p220) | Core shell | 951 | GLIRVIFMRPIELIN |
| BA71V-CP2475L (P220) | BA71V-CP2475L (p220) | Core shell | 952 | LIRVIFMRPIELINI |
| BA71V-CP2475L (P220) | BA71V-CP2475L (p220) | Core shell | 953 | IRVIFMRPIELINIG |
| BA71V-CP2475L (P220) | BA71V-CP2475L (p220) | Core shell | 954 | RVIFMRPIELINIGD |
| BA71V-CP2475L (P220) | BA71V-CP2475L (p220) | Core shell | 955 | VIFMRPIELINIGDY |
| BA71V-CP2475L (P220) | BA71V-CP2475L (p220) | Core shell | 956 | IFMRPIELINIGDYS |

| | | | | |
|---|---|---|---|---|
| BA71V-CP2475L (P220) | BA71V-CP2475L (p220) | Core shell | 957 | FMRPIELINIGDYSE |
| BA71V-CP2475L (P220) | BA71V-CP2475L (p220) | Core shell | 958 | MRPIELINIGDYSET |
| BA71V-CP2475L (P220) | BA71V-CP2475L (p220) | Core shell | 959 | RPIELINIGDYSETE |
| BA71V-CP2475L (P220) | BA71V-CP2475L (p220) | Core shell | 960 | PIELINIGDYSETEI |
| BA71V-CP2475L (P220) | BA71V-CP2475L (p220) | Core shell | 961 | IELINIGDYSETEIR |
| BA71V-CP2475L (P220) | BA71V-CP2475L (p220) | Core shell | 962 | ELINIGDYSETEIRQ |
| BA71V-CP2475L (P220) | BA71V-CP2475L (p220) | Core shell | 963 | LINIGDYSETEIRQL |
| BA71V-CP2475L (P220) | BA71V-CP2475L (p220) | Core shell | 964 | INIGDYSETEIRQLI |
| BA71V-CP2475L (P220) | BA71V-CP2475L (p220) | Core shell | 965 | NIGDYSETEIRQLIK |
| BA71V-CP2475L (P220) | BA71V-CP2475L (p220) | Core shell | 966 | IGDYSETEIRQLIKE |
| BA71V-CP2475L (P220) | BA71V-CP2475L (p220) | Core shell | 967 | GDYSETEIRQLIKEI |
| BA71V-CP2475L (P220) | BA71V-CP2475L (p220) | Core shell | 968 | DYSETEIRQLIKEIN |
| BA71V-CP2475L (P220) | BA71V-CP2475L (p220) | Core shell | 969 | YSETEIRQLIKEINV |
| BA71V-CP2475L (P220) | BA71V-CP2475L (p220) | Core shell | 970 | SETEIRQLIKEINVI |
| BA71V-CP2475L (P220) | BA71V-CP2475L (p220) | Core shell | 971 | ETEIRQLIKEINVIY |
| BA71V-CP2475L (P220) | BA71V-CP2475L (p220) | Core shell | 972 | TEIRQLIKEINVIYQ |
| BA71V-CP2475L (P220) | BA71V-CP2475L (p220) | Core shell | 973 | EIRQLIKEINVIYQH |
| BA71V-CP2475L (P220) | BA71V-CP2475L (p220) | Core shell | 974 | IRQLIKEINVIYQHF |
| BA71V-CP2475L (P220) | BA71V-CP2475L (p220) | Core shell | 975 | RQLIKEINVIYQHFN |
| BA71V-CP2475L (P220) | BA71V-CP2475L (p220) | Core shell | 976 | QLIKEINVIYQHFNL |
| BA71V-CP2475L (P220) | BA71V-CP2475L (p220) | Core shell | 977 | LIKEINVIYQHFNLE |
| BA71V-CP2475L (P220) | BA71V-CP2475L (p220) | Core shell | 978 | IKEINVIYQHFNLEY |
| BA71V-CP2475L (P220) | BA71V-CP2475L (p220) | Core shell | 979 | KEINVIYQHFNLEYG |
| BA71V-CP2475L (P220) | BA71V-CP2475L (p220) | Core shell | 980 | EINVIYQHFNLEYGE |
| BA71V-CP2475L (P220) | BA71V-CP2475L (p220) | Core shell | 981 | INVIYQHFNLEYGEQ |
| BA71V-CP2475L (P220) | BA71V-CP2475L (p220) | Core shell | 982 | NVIYQHFNLEYGEQE |
| BA71V-CP2475L (P220) | BA71V-CP2475L (p220) | Core shell | 983 | VIYQHFNLEYGEQEA |
| BA71V-CP2475L (P220) | BA71V-CP2475L (p220) | Core shell | 984 | IYQHFNLEYGEQEAV |
| BA71V-CP2475L (P220) | BA71V-CP2475L (p220) | Core shell | 985 | YQHFNLEYGEQEAVK |
| BA71V-CP2475L (P220) | BA71V-CP2475L (p220) | Core shell | 986 | QHFNLEYGEQEAVKK |
| BA71V-CP2475L (P220) | BA71V-CP2475L (p220) | Core shell | 987 | HFNLEYGEQEAVKKA |
| BA71V-CP2475L (P220) | BA71V-CP2475L (p220) | Core shell | 988 | FNLEYGEQEAVKKAL |
| BA71V-CP2475L (P220) | BA71V-CP2475L (p220) | Core shell | 989 | NLEYGEQEAVKKALI |
| BA71V-CP2475L (P220) | BA71V-CP2475L (p220) | Core shell | 990 | LEYGEQEAVKKALIH |
| BA71V-CP2475L (P220) | BA71V-CP2475L (p220) | Core shell | 991 | EYGEQEAVKKALIHF |
| BA71V-CP2475L (P220) | BA71V-CP2475L (p220) | Core shell | 992 | YGEQEAVKKALIHFV |
| BA71V-CP2475L (P220) | BA71V-CP2475L (p220) | Core shell | 993 | GEQEAVKKALIHFVN |
| BA71V-CP2475L (P220) | BA71V-CP2475L (p220) | Core shell | 994 | EQEAVKKALIHFVNE |
| BA71V-CP2475L (P220) | BA71V-CP2475L (p220) | Core shell | 995 | QEAVKKALIHFVNEI |
| BA71V-CP2475L (P220) | BA71V-CP2475L (p220) | Core shell | 996 | EAVKKALIHFVNEIN |
| BA71V-CP2475L (P220) | BA71V-CP2475L (p220) | Core shell | 997 | AVKKALIHFVNEINR |
| BA71V-CP2475L (P220) | BA71V-CP2475L (p220) | Core shell | 998 | VKKALIHFVNEINRR |
| BA71V-CP2475L (P220) | BA71V-CP2475L (p220) | Core shell | 999 | KKALIHFVNEINRRF |
| BA71V-CP2475L (P220) | BA71V-CP2475L (p220) | Core shell | 1000 | KALIHFVNEINRRFG |
| BA71V-CP2475L (P220) | BA71V-CP2475L (p220) | Core shell | 1001 | ALIHFVNEINRRFGV |
| BA71V-CP2475L (P220) | BA71V-CP2475L (p220) | Core shell | 1002 | LIHFVNEINRRFGVI |
| BA71V-CP2475L (P220) | BA71V-CP2475L (p220) | Core shell | 1003 | IHFVNEINRRFGVIT |
| BA71V-CP2475L (P220) | BA71V-CP2475L (p220) | Core shell | 1004 | HFVNEINRRFGVITR |
| BA71V-CP2475L (P220) | BA71V-CP2475L (p220) | Core shell | 1005 | FVNEINRRFGVITRT |
| BA71V-CP2475L (P220) | BA71V-CP2475L (p220) | Core shell | 1006 | VNEINRRFGVITRTE |
| BA71V-CP2475L (P220) | BA71V-CP2475L (p220) | Core shell | 1007 | NEINRRFGVITRTEW |
| BA71V-CP2475L (P220) | BA71V-CP2475L (p220) | Core shell | 1008 | EINRRFGVITRTEWE |
| BA71V-CP2475L (P220) | BA71V-CP2475L (p220) | Core shell | 1009 | INRRFGVITRTEWEK |
| BA71V-CP2475L (P220) | BA71V-CP2475L (p220) | Core shell | 1010 | NRRFGVITRTEWEKF |
| BA71V-CP2475L (P220) | BA71V-CP2475L (p220) | Core shell | 1011 | RRFGVITRTEWEKFQ |
| BA71V-CP2475L (P220) | BA71V-CP2475L (p220) | Core shell | 1012 | RFGVITRTEWEKFQR |
| BA71V-CP2475L (P220) | BA71V-CP2475L (p220) | Core shell | 1013 | FGVITRTEWEKFQRI |
| BA71V-CP2475L (P220) | BA71V-CP2475L (p220) | Core shell | 1014 | GVITRTEWEKFQRIV |
| BA71V-CP2475L (P220) | BA71V-CP2475L (p220) | Core shell | 1015 | VITRTEWEKFQRIVQ |
| BA71V-CP2475L (P220) | BA71V-CP2475L (p220) | Core shell | 1016 | ITRTEWEKFQRIVQE |
| BA71V-CP2475L (P220) | BA71V-CP2475L (p220) | Core shell | 1017 | TRTEWEKFQRIVQEA |
| BA71V-CP2475L (P220) | BA71V-CP2475L (p220) | Core shell | 1018 | RTEWEKFQRIVQEAR |
| BA71V-CP2475L (P220) | BA71V-CP2475L (p220) | Core shell | 1019 | TEWEKFQRIVQEART |
| BA71V-CP2475L (P220) | BA71V-CP2475L (p220) | Core shell | 1020 | EWEKFQRIVQEARTM |
| BA71V-CP2475L (P220) | BA71V-CP2475L (p220) | Core shell | 1021 | WEKFQRIVQEARTMN |
| BA71V-CP2475L (P220) | BA71V-CP2475L (p220) | Core shell | 1022 | EKFQRIVQEARTMND |
| BA71V-CP2475L (P220) | BA71V-CP2475L (p220) | Core shell | 1023 | KFQRIVQEARTMNDF |

| | | | |
|---|---|---|---|
| BA71V-CP2475L (P220) | BA71V-CP2475L (p220) | Core shell | 1024 FQRIVQEARTMNDFG |
| BA71V-CP2475L (P220) | BA71V-CP2475L (p220) | Core shell | 1025 QRIVQEARTMNDFGM |
| BA71V-CP2475L (P220) | BA71V-CP2475L (p220) | Core shell | 1026 RIVQEARTMNDFGMM |
| BA71V-CP2475L (P220) | BA71V-CP2475L (p220) | Core shell | 1027 IVQEARTMNDFGMMN |
| BA71V-CP2475L (P220) | BA71V-CP2475L (p220) | Core shell | 1028 VQEARTMNDFGMMNQ |
| BA71V-CP2475L (P220) | BA71V-CP2475L (p220) | Core shell | 1029 QEARTMNDFGMMNQT |
| BA71V-CP2475L (P220) | BA71V-CP2475L (p220) | Core shell | 1030 EARTMNDFGMMNQTN |
| BA71V-CP2475L (P220) | BA71V-CP2475L (p220) | Core shell | 1031 ARTMNDFGMMNQTNY |
| BA71V-CP2475L (P220) | BA71V-CP2475L (p220) | Core shell | 1032 RTMNDFGMMNQTNYS |
| BA71V-CP2475L (P220) | BA71V-CP2475L (p220) | Core shell | 1033 TMNDFGMMNQTNYSI |
| BA71V-CP2475L (P220) | BA71V-CP2475L (p220) | Core shell | 1034 MNDFGMMNQTNYSIL |
| BA71V-CP2475L (P220) | BA71V-CP2475L (p220) | Core shell | 1035 NDFGMMNQTNYSILP |
| BA71V-CP2475L (P220) | BA71V-CP2475L (p220) | Core shell | 1036 DFGMMNQTNYSILPD |
| BA71V-CP2475L (P220) | BA71V-CP2475L (p220) | Core shell | 1037 FGMMNQTNYSILPDE |
| BA71V-CP2475L (P220) | BA71V-CP2475L (p220) | Core shell | 1038 GMMNQTNYSILPDED |
| BA71V-CP2475L (P220) | BA71V-CP2475L (p220) | Core shell | 1039 MMNQTNYSILPDEDG |
| BA71V-CP2475L (P220) | BA71V-CP2475L (p220) | Core shell | 1040 MNQTNYSILPDEDGY |
| BA71V-CP2475L (P220) | BA71V-CP2475L (p220) | Core shell | 1041 NQTNYSILPDEDGYT |
| BA71V-CP2475L (P220) | BA71V-CP2475L (p220) | Core shell | 1042 QTNYSILPDEDGYTQ |
| BA71V-CP2475L (P220) | BA71V-CP2475L (p220) | Core shell | 1043 TNYSILPDEDGYTQS |
| BA71V-CP2475L (P220) | BA71V-CP2475L (p220) | Core shell | 1044 NYSILPDEDGYTQSS |
| BA71V-CP2475L (P220) | BA71V-CP2475L (p220) | Core shell | 1045 YSILPDEDGYTQSSQ |
| BA71V-CP2475L (P220) | BA71V-CP2475L (p220) | Core shell | 1046 SILPDEDGYTQSSQL |
| BA71V-CP2475L (P220) | BA71V-CP2475L (p220) | Core shell | 1047 ILPDEDGYTQSSQLL |
| BA71V-CP2475L (P220) | BA71V-CP2475L (p220) | Core shell | 1048 LPDEDGYTQSSQLLP |
| BA71V-CP2475L (P220) | BA71V-CP2475L (p220) | Core shell | 1049 PDEDGYTQSSQLLPS |
| BA71V-CP2475L (P220) | BA71V-CP2475L (p220) | Core shell | 1050 DEDGYTQSSQLLPSD |
| BA71V-CP2475L (P220) | BA71V-CP2475L (p220) | Core shell | 1051 EDGYTQSSQLLPSDR |
| BA71V-CP2475L (P220) | BA71V-CP2475L (p220) | Core shell | 1052 DGYTQSSQLLPSDRF |
| BA71V-CP2475L (P220) | BA71V-CP2475L (p220) | Core shell | 1053 GYTQSSQLLPSDRFI |
| BA71V-CP2475L (P220) | BA71V-CP2475L (p220) | Core shell | 1054 YTQSSQLLPSDRFIG |
| BA71V-CP2475L (P220) | BA71V-CP2475L (p220) | Core shell | 1055 TQSSQLLPSDRFIGP |
| BA71V-CP2475L (P220) | BA71V-CP2475L (p220) | Core shell | 1056 QSSQLLPSDRFIGPS |
| BA71V-CP2475L (P220) | BA71V-CP2475L (p220) | Core shell | 1057 SSQLLPSDRFIGPSS |
| BA71V-CP2475L (P220) | BA71V-CP2475L (p220) | Core shell | 1058 SQLLPSDRFIGPSSQ |
| BA71V-CP2475L (P220) | BA71V-CP2475L (p220) | Core shell | 1059 QLLPSDRFIGPSSQP |
| BA71V-CP2475L (P220) | BA71V-CP2475L (p220) | Core shell | 1060 LLPSDRFIGPSSQPT |
| BA71V-CP2475L (P220) | BA71V-CP2475L (p220) | Core shell | 1061 LPSDRFIGPSSQPTP |
| BA71V-CP2475L (P220) | BA71V-CP2475L (p220) | Core shell | 1062 PSDRFIGPSSQPTPK |
| BA71V-CP2475L (P220) | BA71V-CP2475L (p220) | Core shell | 1063 SDRFIGPSSQPTPKW |
| BA71V-CP2475L (P220) | BA71V-CP2475L (p220) | Core shell | 1064 DRFIGPSSQPTPKWR |
| BA71V-CP2475L (P220) | BA71V-CP2475L (p220) | Core shell | 1065 RFIGPSSQPTPKWRP |
| BA71V-CP2475L (P220) | BA71V-CP2475L (p220) | Core shell | 1066 FIGPSSQPTPKWRPA |
| BA71V-CP2475L (P220) | BA71V-CP2475L (p220) | Core shell | 1067 IGPSSQPTPKWRPAL |
| BA71V-CP2475L (P220) | BA71V-CP2475L (p220) | Core shell | 1068 GPSSQPTPKWRPALY |
| BA71V-CP2475L (P220) | BA71V-CP2475L (p220) | Core shell | 1069 PSSQPTPKWRPALYN |
| BA71V-CP2475L (P220) | BA71V-CP2475L (p220) | Core shell | 1070 SSQPTPKWRPALYNI |
| BA71V-CP2475L (P220) | BA71V-CP2475L (p220) | Core shell | 1071 SQPTPKWRPALYNID |
| BA71V-CP2475L (P220) | BA71V-CP2475L (p220) | Core shell | 1072 QPTPKWRPALYNIDS |
| BA71V-CP2475L (P220) | BA71V-CP2475L (p220) | Core shell | 1073 PTPKWRPALYNIDSV |
| BA71V-CP2475L (P220) | BA71V-CP2475L (p220) | Core shell | 1074 TPKWRPALYNIDSVD |
| BA71V-CP2475L (P220) | BA71V-CP2475L (p220) | Core shell | 1075 PKWRPALYNIDSVDV |
| BA71V-CP2475L (P220) | BA71V-CP2475L (p220) | Core shell | 1076 KWRPALYNIDSVDVQ |
| BA71V-CP2475L (P220) | BA71V-CP2475L (p220) | Core shell | 1077 WRPALYNIDSVDVQT |
| BA71V-CP2475L (P220) | BA71V-CP2475L (p220) | Core shell | 1078 RPALYNIDSVDVQTG |
| BA71V-CP2475L (P220) | BA71V-CP2475L (p220) | Core shell | 1079 PALYNIDSVDVQTGM |
| BA71V-CP2475L (P220) | BA71V-CP2475L (p220) | Core shell | 1080 ALYNIDSVDVQTGML |
| BA71V-CP2475L (P220) | BA71V-CP2475L (p220) | Core shell | 1081 LYNIDSVDVQTGMLQ |
| BA71V-CP2475L (P220) | BA71V-CP2475L (p220) | Core shell | 1082 YNIDSVDVQTGMLQP |
| BA71V-CP2475L (P220) | BA71V-CP2475L (p220) | Core shell | 1083 NIDSVDVQTGMLQPN |
| BA71V-CP2475L (P220) | BA71V-CP2475L (p220) | Core shell | 1084 IDSVDVQTGMLQPNS |
| BA71V-CP2475L (P220) | BA71V-CP2475L (p220) | Core shell | 1085 DSVDVQTGMLQPNSQ |
| BA71V-CP2475L (P220) | BA71V-CP2475L (p220) | Core shell | 1086 SVDVQTGMLQPNSQW |
| BA71V-CP2475L (P220) | BA71V-CP2475L (p220) | Core shell | 1087 VDVQTGMLQPNSQWD |
| BA71V-CP2475L (P220) | BA71V-CP2475L (p220) | Core shell | 1088 DVQTGMLQPNSQWDL |
| BA71V-CP2475L (P220) | BA71V-CP2475L (p220) | Core shell | 1089 VQTGMLQPNSQWDLV |
| BA71V-CP2475L (P220) | BA71V-CP2475L (p220) | Core shell | 1090 QTGMLQPNSQWDLVQ |

| | | | | |
|---|---|---|---|---|
| BA71V-CP2475L (P220) | BA71V-CP2475L (p220) | Core shell | 1091 | TGMLQPNSQWDLVQK |
| BA71V-CP2475L (P220) | BA71V-CP2475L (p220) | Core shell | 1092 | GMLQPNSQWDLVQKF |
| BA71V-CP2475L (P220) | BA71V-CP2475L (p220) | Core shell | 1093 | MLQPNSQWDLVQKFR |
| BA71V-CP2475L (P220) | BA71V-CP2475L (p220) | Core shell | 1094 | LQPNSQWDLVQKFRK |
| BA71V-CP2475L (P220) | BA71V-CP2475L (p220) | Core shell | 1095 | QPNSQWDLVQKFRKQ |
| BA71V-CP2475L (P220) | BA71V-CP2475L (p220) | Core shell | 1096 | PNSQWDLVQKFRKQL |
| BA71V-CP2475L (P220) | BA71V-CP2475L (p220) | Core shell | 1097 | NSQWDLVQKFRKQLS |
| BA71V-CP2475L (P220) | BA71V-CP2475L (p220) | Core shell | 1098 | SQWDLVQKFRKQLSE |
| BA71V-CP2475L (P220) | BA71V-CP2475L (p220) | Core shell | 1099 | QWDLVQKFRKQLSEM |
| BA71V-CP2475L (P220) | BA71V-CP2475L (p220) | Core shell | 1100 | WDLVQKFRKQLSEMF |
| BA71V-CP2475L (P220) | BA71V-CP2475L (p220) | Core shell | 1101 | DLVQKFRKQLSEMFE |
| BA71V-CP2475L (P220) | BA71V-CP2475L (p220) | Core shell | 1102 | LVQKFRKQLSEMFED |
| BA71V-CP2475L (P220) | BA71V-CP2475L (p220) | Core shell | 1103 | VQKFRKQLSEMFEDP |
| BA71V-CP2475L (P220) | BA71V-CP2475L (p220) | Core shell | 1104 | QKFRKQLSEMFEDPS |
| BA71V-CP2475L (P220) | BA71V-CP2475L (p220) | Core shell | 1105 | KFRKQLSEMFEDPSL |
| BA71V-CP2475L (P220) | BA71V-CP2475L (p220) | Core shell | 1106 | FRKQLSEMFEDPSLQ |
| BA71V-CP2475L (P220) | BA71V-CP2475L (p220) | Core shell | 1107 | RKQLSEMFEDPSLQQ |
| BA71V-CP2475L (P220) | BA71V-CP2475L (p220) | Core shell | 1108 | KQLSEMFEDPSLQQE |
| BA71V-CP2475L (P220) | BA71V-CP2475L (p220) | Core shell | 1109 | QLSEMFEDPSLQQEL |
| BA71V-CP2475L (P220) | BA71V-CP2475L (p220) | Core shell | 1110 | LSEMFEDPSLQQELG |
| BA71V-CP2475L (P220) | BA71V-CP2475L (p220) | Core shell | 1111 | SEMFEDPSLQQELGK |
| BA71V-CP2475L (P220) | BA71V-CP2475L (p220) | Core shell | 1112 | EMFEDPSLQQELGKV |
| BA71V-CP2475L (P220) | BA71V-CP2475L (p220) | Core shell | 1113 | MFEDPSLQQELGKVS |
| BA71V-CP2475L (P220) | BA71V-CP2475L (p220) | Core shell | 1114 | FEDPSLQQELGKVSY |
| BA71V-CP2475L (P220) | BA71V-CP2475L (p220) | Core shell | 1115 | EDPSLQQELGKVSYQ |
| BA71V-CP2475L (P220) | BA71V-CP2475L (p220) | Core shell | 1116 | DPSLQQELGKVSYQE |
| BA71V-CP2475L (P220) | BA71V-CP2475L (p220) | Core shell | 1117 | PSLQQELGKVSYQEL |
| BA71V-CP2475L (P220) | BA71V-CP2475L (p220) | Core shell | 1118 | SLQQELGKVSYQELI |
| BA71V-CP2475L (P220) | BA71V-CP2475L (p220) | Core shell | 1119 | LQQELGKVSYQELIQ |
| BA71V-CP2475L (P220) | BA71V-CP2475L (p220) | Core shell | 1120 | QQELGKVSYQELIQQ |
| BA71V-CP2475L (P220) | BA71V-CP2475L (p220) | Core shell | 1121 | QELGKVSYQELIQQA |
| BA71V-CP2475L (P220) | BA71V-CP2475L (p220) | Core shell | 1122 | ELGKVSYQELIQQAT |
| BA71V-CP2475L (P220) | BA71V-CP2475L (p220) | Core shell | 1123 | LGKVSYQELIQQATN |
| BA71V-CP2475L (P220) | BA71V-CP2475L (p220) | Core shell | 1124 | GKVSYQELIQQATNE |
| BA71V-CP2475L (P220) | BA71V-CP2475L (p220) | Core shell | 1125 | KVSYQELIQQATNEL |
| BA71V-CP2475L (P220) | BA71V-CP2475L (p220) | Core shell | 1126 | VSYQELIQQATNELK |
| BA71V-CP2475L (P220) | BA71V-CP2475L (p220) | Core shell | 1127 | SYQELIQQATNELKK |
| BA71V-CP2475L (P220) | BA71V-CP2475L (p220) | Core shell | 1128 | YQELIQQATNELKKE |
| BA71V-CP2475L (P220) | BA71V-CP2475L (p220) | Core shell | 1129 | QELIQQATNELKKEH |
| BA71V-CP2475L (P220) | BA71V-CP2475L (p220) | Core shell | 1130 | ELIQQATNELKKEHT |
| BA71V-CP2475L (P220) | BA71V-CP2475L (p220) | Core shell | 1131 | LIQQATNELKKEHTD |
| BA71V-CP2475L (P220) | BA71V-CP2475L (p220) | Core shell | 1132 | IQQATNELKKEHTDK |
| BA71V-CP2475L (P220) | BA71V-CP2475L (p220) | Core shell | 1133 | QQATNELKKEHTDKI |
| BA71V-CP2475L (P220) | BA71V-CP2475L (p220) | Core shell | 1134 | QATNELKKEHTDKIQ |
| BA71V-CP2475L (P220) | BA71V-CP2475L (p220) | Core shell | 1135 | ATNELKKEHTDKIQI |
| BA71V-CP2475L (P220) | BA71V-CP2475L (p220) | Core shell | 1136 | TNELKKEHTDKIQIV |
| BA71V-CP2475L (P220) | BA71V-CP2475L (p220) | Core shell | 1137 | NELKKEHTDKIQIVS |
| BA71V-CP2475L (P220) | BA71V-CP2475L (p220) | Core shell | 1138 | ELKKEHTDKIQIVSK |
| BA71V-CP2475L (P220) | BA71V-CP2475L (p220) | Core shell | 1139 | LKKEHTDKIQIVSKL |
| BA71V-CP2475L (P220) | BA71V-CP2475L (p220) | Core shell | 1140 | KKEHTDKIQIVSKLI |
| BA71V-CP2475L (P220) | BA71V-CP2475L (p220) | Core shell | 1141 | KEHTDKIQIVSKLIQ |
| BA71V-CP2475L (P220) | BA71V-CP2475L (p220) | Core shell | 1142 | EHTDKIQIVSKLIQG |
| BA71V-CP2475L (P220) | BA71V-CP2475L (p220) | Core shell | 1143 | HTDKIQIVSKLIQGS |
| BA71V-CP2475L (P220) | BA71V-CP2475L (p220) | Core shell | 1144 | TDKIQIVSKLIQGSE |
| BA71V-CP2475L (P220) | BA71V-CP2475L (p220) | Core shell | 1145 | DKIQIVSKLIQGSES |
| BA71V-CP2475L (P220) | BA71V-CP2475L (p220) | Core shell | 1146 | KIQIVSKLIQGSESL |
| BA71V-CP2475L (P220) | BA71V-CP2475L (p220) | Core shell | 1147 | IQIVSKLIQGSESLA |
| BA71V-CP2475L (P220) | BA71V-CP2475L (p220) | Core shell | 1148 | QIVSKLIQGSESLAD |
| BA71V-CP2475L (P220) | BA71V-CP2475L (p220) | Core shell | 1149 | IVSKLIQGSESLADT |
| BA71V-CP2475L (P220) | BA71V-CP2475L (p220) | Core shell | 1150 | VSKLIQGSESLADTD |
| BA71V-CP2475L (P220) | BA71V-CP2475L (p220) | Core shell | 1151 | SKLIQGSESLADTDV |
| BA71V-CP2475L (P220) | BA71V-CP2475L (p220) | Core shell | 1152 | KLIQGSESLADTDVN |
| BA71V-CP2475L (P220) | BA71V-CP2475L (p220) | Core shell | 1153 | LIQGSESLADTDVNK |
| BA71V-CP2475L (P220) | BA71V-CP2475L (p220) | Core shell | 1154 | IQGSESLADTDVNKI |
| BA71V-CP2475L (P220) | BA71V-CP2475L (p220) | Core shell | 1155 | QGSESLADTDVNKIF |
| BA71V-CP2475L (P220) | BA71V-CP2475L (p220) | Core shell | 1156 | GSESLADTDVNKIFL |
| BA71V-CP2475L (P220) | BA71V-CP2475L (p220) | Core shell | 1157 | SESLADTDVNKIFLF |

| | | | | |
|---|---|---|---|---|
| BA71V-CP2475L (P220) | BA71V-CP2475L (p220) | Core shell | 1158 | ESLADTDVNKIFLFH |
| BA71V-CP2475L (P220) | BA71V-CP2475L (p220) | Core shell | 1159 | SLADTDVNKIFLFHE |
| BA71V-CP2475L (P220) | BA71V-CP2475L (p220) | Core shell | 1160 | LADTDVNKIFLFHET |
| BA71V-CP2475L (P220) | BA71V-CP2475L (p220) | Core shell | 1161 | ADTDVNKIFLFHETV |
| BA71V-CP2475L (P220) | BA71V-CP2475L (p220) | Core shell | 1162 | DTDVNKIFLFHETVI |
| BA71V-CP2475L (P220) | BA71V-CP2475L (p220) | Core shell | 1163 | TDVNKIFLFHETVIT |
| BA71V-CP2475L (P220) | BA71V-CP2475L (p220) | Core shell | 1164 | DVNKIFLFHETVITG |
| BA71V-CP2475L (P220) | BA71V-CP2475L (p220) | Core shell | 1165 | VNKIFLFHETVITGL |
| BA71V-CP2475L (P220) | BA71V-CP2475L (p220) | Core shell | 1166 | NKIFLFHETVITGLN |
| BA71V-CP2475L (P220) | BA71V-CP2475L (p220) | Core shell | 1167 | KIFLFHETVITGLNL |
| BA71V-CP2475L (P220) | BA71V-CP2475L (p220) | Core shell | 1168 | IFLFHETVITGLNLL |
| BA71V-CP2475L (P220) | BA71V-CP2475L (p220) | Core shell | 1169 | FLFHETVITGLNLLS |
| BA71V-CP2475L (P220) | BA71V-CP2475L (p220) | Core shell | 1170 | LFHETVITGLNLLSA |
| BA71V-CP2475L (P220) | BA71V-CP2475L (p220) | Core shell | 1171 | FHETVITGLNLLSAI |
| BA71V-CP2475L (P220) | BA71V-CP2475L (p220) | Core shell | 1172 | HETVITGLNLLSAIY |
| BA71V-CP2475L (P220) | BA71V-CP2475L (p220) | Core shell | 1173 | ETVITGLNLLSAIYV |
| BA71V-CP2475L (P220) | BA71V-CP2475L (p220) | Core shell | 1174 | TVITGLNLLSAIYVL |
| BA71V-CP2475L (P220) | BA71V-CP2475L (p220) | Core shell | 1175 | VITGLNLLSAIYVLL |
| BA71V-CP2475L (P220) | BA71V-CP2475L (p220) | Core shell | 1176 | ITGLNLLSAIYVLLN |
| BA71V-CP2475L (P220) | BA71V-CP2475L (p220) | Core shell | 1177 | TGLNLLSAIYVLLNT |
| BA71V-CP2475L (P220) | BA71V-CP2475L (p220) | Core shell | 1178 | GLNLLSAIYVLLNTF |
| BA71V-CP2475L (P220) | BA71V-CP2475L (p220) | Core shell | 1179 | LNLLSAIYVLLNTFR |
| BA71V-CP2475L (P220) | BA71V-CP2475L (p220) | Core shell | 1180 | NLLSAIYVLLNTFRN |
| BA71V-CP2475L (P220) | BA71V-CP2475L (p220) | Core shell | 1181 | LLSAIYVLLNTFRNN |
| BA71V-CP2475L (P220) | BA71V-CP2475L (p220) | Core shell | 1182 | LSAIYVLLNTFRNNI |
| BA71V-CP2475L (P220) | BA71V-CP2475L (p220) | Core shell | 1183 | SAIYVLLNTFRNNIK |
| BA71V-CP2475L (P220) | BA71V-CP2475L (p220) | Core shell | 1184 | AIYVLLNTFRNNIKA |
| BA71V-CP2475L (P220) | BA71V-CP2475L (p220) | Core shell | 1185 | IYVLLNTFRNNIKAL |
| BA71V-CP2475L (P220) | BA71V-CP2475L (p220) | Core shell | 1186 | YVLLNTFRNNIKALD |
| BA71V-CP2475L (P220) | BA71V-CP2475L (p220) | Core shell | 1187 | VLLNTFRNNIKALDL |
| BA71V-CP2475L (P220) | BA71V-CP2475L (p220) | Core shell | 1188 | LLNTFRNNIKALDLD |
| BA71V-CP2475L (P220) | BA71V-CP2475L (p220) | Core shell | 1189 | LNTFRNNIKALDLDT |
| BA71V-CP2475L (P220) | BA71V-CP2475L (p220) | Core shell | 1190 | NTFRNNIKALDLDTI |
| BA71V-CP2475L (P220) | BA71V-CP2475L (p220) | Core shell | 1191 | TFRNNIKALDLDTIQ |
| BA71V-CP2475L (P220) | BA71V-CP2475L (p220) | Core shell | 1192 | FRNNIKALDLDTIQK |
| BA71V-CP2475L (P220) | BA71V-CP2475L (p220) | Core shell | 1193 | RNNIKALDLDTIQKS |
| BA71V-CP2475L (P220) | BA71V-CP2475L (p220) | Core shell | 1194 | NNIKALDLDTIQKSI |
| BA71V-CP2475L (P220) | BA71V-CP2475L (p220) | Core shell | 1195 | NIKALDLDTIQKSII |
| BA71V-CP2475L (P220) | BA71V-CP2475L (p220) | Core shell | 1196 | IKALDLDTIQKSIIE |
| BA71V-CP2475L (P220) | BA71V-CP2475L (p220) | Core shell | 1197 | KALDLDTIQKSIIEW |
| BA71V-CP2475L (P220) | BA71V-CP2475L (p220) | Core shell | 1198 | ALDLDTIQKSIIEWL |
| BA71V-CP2475L (P220) | BA71V-CP2475L (p220) | Core shell | 1199 | LDLDTIQKSIIEWLR |
| BA71V-CP2475L (P220) | BA71V-CP2475L (p220) | Core shell | 1200 | DLDTIQKSIIEWLRE |
| BA71V-CP2475L (P220) | BA71V-CP2475L (p220) | Core shell | 1201 | LDTIQKSIIEWLRET |
| BA71V-CP2475L (P220) | BA71V-CP2475L (p220) | Core shell | 1202 | DTIQKSIIEWLRETQ |
| BA71V-CP2475L (P220) | BA71V-CP2475L (p220) | Core shell | 1203 | TIQKSIIEWLRETQA |
| BA71V-CP2475L (P220) | BA71V-CP2475L (p220) | Core shell | 1204 | IQKSIIEWLRETQAA |
| BA71V-CP2475L (P220) | BA71V-CP2475L (p220) | Core shell | 1205 | QKSIIEWLRETQAAN |
| BA71V-CP2475L (P220) | BA71V-CP2475L (p220) | Core shell | 1206 | KSIIEWLRETQAANV |
| BA71V-CP2475L (P220) | BA71V-CP2475L (p220) | Core shell | 1207 | SIIEWLRETQAANVN |
| BA71V-CP2475L (P220) | BA71V-CP2475L (p220) | Core shell | 1208 | IIEWLRETQAANVNR |
| BA71V-CP2475L (P220) | BA71V-CP2475L (p220) | Core shell | 1209 | IEWLRETQAANVNRA |
| BA71V-CP2475L (P220) | BA71V-CP2475L (p220) | Core shell | 1210 | EWLRETQAANVNRAN |
| BA71V-CP2475L (P220) | BA71V-CP2475L (p220) | Core shell | 1211 | WLRETQAANVNRANL |
| BA71V-CP2475L (P220) | BA71V-CP2475L (p220) | Core shell | 1212 | LRETQAANVNRANLI |
| BA71V-CP2475L (P220) | BA71V-CP2475L (p220) | Core shell | 1213 | RETQAANVNRANLID |
| BA71V-CP2475L (P220) | BA71V-CP2475L (p220) | Core shell | 1214 | ETQAANVNRANLIDW |
| BA71V-CP2475L (P220) | BA71V-CP2475L (p220) | Core shell | 1215 | TQAANVNRANLIDWL |
| BA71V-CP2475L (P220) | BA71V-CP2475L (p220) | Core shell | 1216 | QAANVNRANLIDWLG |
| BA71V-CP2475L (P220) | BA71V-CP2475L (p220) | Core shell | 1217 | AANVNRANLIDWLGR |
| BA71V-CP2475L (P220) | BA71V-CP2475L (p220) | Core shell | 1218 | ANVNRANLIDWLGRR |
| BA71V-CP2475L (P220) | BA71V-CP2475L (p220) | Core shell | 1219 | NVNRANLIDWLGRRH |
| BA71V-CP2475L (P220) | BA71V-CP2475L (p220) | Core shell | 1220 | VNRANLIDWLGRRHG |
| BA71V-CP2475L (P220) | BA71V-CP2475L (p220) | Core shell | 1221 | NRANLIDWLGRRHGD |
| BA71V-CP2475L (P220) | BA71V-CP2475L (p220) | Core shell | 1222 | RANLIDWLGRRHGDI |
| BA71V-CP2475L (P220) | BA71V-CP2475L (p220) | Core shell | 1223 | ANLIDWLGRRHGDIS |
| BA71V-CP2475L (P220) | BA71V-CP2475L (p220) | Core shell | 1224 | NLIDWLGRRHGDISE |

| | | | | |
|---|---|---|---|---|
| BA71V-CP2475L (P220) | BA71V-CP2475L (p220) | Core shell | 1225 | LIDWLGRRHGDISEI |
| BA71V-CP2475L (P220) | BA71V-CP2475L (p220) | Core shell | 1226 | IDWLGRRHGDISEIR |
| BA71V-CP2475L (P220) | BA71V-CP2475L (p220) | Core shell | 1227 | DWLGRRHGDISEIRN |
| BA71V-CP2475L (P220) | BA71V-CP2475L (p220) | Core shell | 1228 | WLGRRHGDISEIRNP |
| BA71V-CP2475L (P220) | BA71V-CP2475L (p220) | Core shell | 1229 | LGRRHGDISEIRNPG |
| BA71V-CP2475L (P220) | BA71V-CP2475L (p220) | Core shell | 1230 | GRRHGDISEIRNPGL |
| BA71V-CP2475L (P220) | BA71V-CP2475L (p220) | Core shell | 1231 | RRHGDISEIRNPGLV |
| BA71V-CP2475L (P220) | BA71V-CP2475L (p220) | Core shell | 1232 | RHGDISEIRNPGLVI |
| BA71V-CP2475L (P220) | BA71V-CP2475L (p220) | Core shell | 1233 | HGDISEIRNPGLVIK |
| BA71V-CP2475L (P220) | BA71V-CP2475L (p220) | Core shell | 1234 | GDISEIRNPGLVIKA |
| BA71V-CP2475L (P220) | BA71V-CP2475L (p220) | Core shell | 1235 | DISEIRNPGLVIKAN |
| BA71V-CP2475L (P220) | BA71V-CP2475L (p220) | Core shell | 1236 | ISEIRNPGLVIKAND |
| BA71V-CP2475L (P220) | BA71V-CP2475L (p220) | Core shell | 1237 | SEIRNPGLVIKANDA |
| BA71V-CP2475L (P220) | BA71V-CP2475L (p220) | Core shell | 1238 | EIRNPGLVIKANDAR |
| BA71V-CP2475L (P220) | BA71V-CP2475L (p220) | Core shell | 1239 | IRNPGLVIKANDARL |
| BA71V-CP2475L (P220) | BA71V-CP2475L (p220) | Core shell | 1240 | RNPGLVIKANDARLS |
| BA71V-CP2475L (P220) | BA71V-CP2475L (p220) | Core shell | 1241 | NPGLVIKANDARLSE |
| BA71V-CP2475L (P220) | BA71V-CP2475L (p220) | Core shell | 1242 | PGLVIKANDARLSEV |
| BA71V-CP2475L (P220) | BA71V-CP2475L (p220) | Core shell | 1243 | GLVIKANDARLSEVY |
| BA71V-CP2475L (P220) | BA71V-CP2475L (p220) | Core shell | 1244 | LVIKANDARLSEVYP |
| BA71V-CP2475L (P220) | BA71V-CP2475L (p220) | Core shell | 1245 | VIKANDARLSEVYPD |
| BA71V-CP2475L (P220) | BA71V-CP2475L (p220) | Core shell | 1246 | IKANDARLSEVYPDP |
| BA71V-CP2475L (P220) | BA71V-CP2475L (p220) | Core shell | 1247 | KANDARLSEVYPDPT |
| BA71V-CP2475L (P220) | BA71V-CP2475L (p220) | Core shell | 1248 | ANDARLSEVYPDPTT |
| BA71V-CP2475L (P220) | BA71V-CP2475L (p220) | Core shell | 1249 | NDARLSEVYPDPTTD |
| BA71V-CP2475L (P220) | BA71V-CP2475L (p220) | Core shell | 1250 | DARLSEVYPDPTTDA |
| BA71V-CP2475L (P220) | BA71V-CP2475L (p220) | Core shell | 1251 | ARLSEVYPDPTTDAT |
| BA71V-CP2475L (P220) | BA71V-CP2475L (p220) | Core shell | 1252 | RLSEVYPDPTTDATA |
| BA71V-CP2475L (P220) | BA71V-CP2475L (p220) | Core shell | 1253 | LSEVYPDPTTDATAP |
| BA71V-CP2475L (P220) | BA71V-CP2475L (p220) | Core shell | 1254 | SEVYPDPTTDATAPL |
| BA71V-CP2475L (P220) | BA71V-CP2475L (p220) | Core shell | 1255 | EVYPDPTTDATAPLD |
| BA71V-CP2475L (P220) | BA71V-CP2475L (p220) | Core shell | 1256 | VYPDPTTDATAPLDR |
| BA71V-CP2475L (P220) | BA71V-CP2475L (p220) | Core shell | 1257 | YPDPTTDATAPLDRN |
| BA71V-CP2475L (P220) | BA71V-CP2475L (p220) | Core shell | 1258 | PDPTTDATAPLDRNL |
| BA71V-CP2475L (P220) | BA71V-CP2475L (p220) | Core shell | 1259 | DPTTDATAPLDRNLV |
| BA71V-CP2475L (P220) | BA71V-CP2475L (p220) | Core shell | 1260 | PTTDATAPLDRNLVT |
| BA71V-CP2475L (P220) | BA71V-CP2475L (p220) | Core shell | 1261 | TTDATAPLDRNLVTE |
| BA71V-CP2475L (P220) | BA71V-CP2475L (p220) | Core shell | 1262 | TDATAPLDRNLVTET |
| BA71V-CP2475L (P220) | BA71V-CP2475L (p220) | Core shell | 1263 | DATAPLDRNLVTETL |
| BA71V-CP2475L (P220) | BA71V-CP2475L (p220) | Core shell | 1264 | ATAPLDRNLVTETLF |
| BA71V-CP2475L (P220) | BA71V-CP2475L (p220) | Core shell | 1265 | TAPLDRNLVTETLFA |
| BA71V-CP2475L (P220) | BA71V-CP2475L (p220) | Core shell | 1266 | APLDRNLVTETLFAW |
| BA71V-CP2475L (P220) | BA71V-CP2475L (p220) | Core shell | 1267 | PLDRNLVTETLFAWF |
| BA71V-CP2475L (P220) | BA71V-CP2475L (p220) | Core shell | 1268 | LDRNLVTETLFAWFT |
| BA71V-CP2475L (P220) | BA71V-CP2475L (p220) | Core shell | 1269 | DRNLVTETLFAWFTR |
| BA71V-CP2475L (P220) | BA71V-CP2475L (p220) | Core shell | 1270 | RNLVTETLFAWFTRF |
| BA71V-CP2475L (P220) | BA71V-CP2475L (p220) | Core shell | 1271 | NLVTETLFAWFTRFV |
| BA71V-CP2475L (P220) | BA71V-CP2475L (p220) | Core shell | 1272 | LVTETLFAWFTRFVG |
| BA71V-CP2475L (P220) | BA71V-CP2475L (p220) | Core shell | 1273 | VTETLFAWFTRFVGI |
| BA71V-CP2475L (P220) | BA71V-CP2475L (p220) | Core shell | 1274 | TETLFAWFTRFVGIP |
| BA71V-CP2475L (P220) | BA71V-CP2475L (p220) | Core shell | 1275 | ETLFAWFTRFVGIPA |
| BA71V-CP2475L (P220) | BA71V-CP2475L (p220) | Core shell | 1276 | TLFAWFTRFVGIPAD |
| BA71V-CP2475L (P220) | BA71V-CP2475L (p220) | Core shell | 1277 | LFAWFTRFVGIPADG |
| BA71V-CP2475L (P220) | BA71V-CP2475L (p220) | Core shell | 1278 | FAWFTRFVGIPADGG |
| BA71V-CP2475L (P220) | BA71V-CP2475L (p220) | Core shell | 1279 | AWFTRFVGIPADGGV |
| BA71V-CP2475L (P220) | BA71V-CP2475L (p220) | Core shell | 1280 | WFTRFVGIPADGGVR |
| BA71V-CP2475L (P220) | BA71V-CP2475L (p220) | Core shell | 1281 | FTRFVGIPADGGVRP |
| BA71V-CP2475L (P220) | BA71V-CP2475L (p220) | Core shell | 1282 | TRFVGIPADGGVRPE |
| BA71V-CP2475L (P220) | BA71V-CP2475L (p220) | Core shell | 1283 | RFVGIPADGGVRPEQ |
| BA71V-CP2475L (P220) | BA71V-CP2475L (p220) | Core shell | 1284 | FVGIPADGGVRPEQE |
| BA71V-CP2475L (P220) | BA71V-CP2475L (p220) | Core shell | 1285 | VGIPADGGVRPEQEL |
| BA71V-CP2475L (P220) | BA71V-CP2475L (p220) | Core shell | 1286 | GIPADGGVRPEQELA |
| BA71V-CP2475L (P220) | BA71V-CP2475L (p220) | Core shell | 1287 | IPADGGVRPEQELAA |
| BA71V-CP2475L (P220) | BA71V-CP2475L (p220) | Core shell | 1288 | PADGGVRPEQELAAR |
| BA71V-CP2475L (P220) | BA71V-CP2475L (p220) | Core shell | 1289 | ADGGVRPEQELAARY |
| BA71V-CP2475L (P220) | BA71V-CP2475L (p220) | Core shell | 1290 | DGGVRPEQELAARYL |
| BA71V-CP2475L (P220) | BA71V-CP2475L (p220) | Core shell | 1291 | GGVRPEQELAARYLV |

| | | | |
|---|---|---|---|
| BA71V-CP2475L (P220) | BA71V-CP2475L (p220) | Core shell | 1292 GVRPEQELAARYLVD |
| BA71V-CP2475L (P220) | BA71V-CP2475L (p220) | Core shell | 1293 VRPEQELAARYLVDN |
| BA71V-CP2475L (P220) | BA71V-CP2475L (p220) | Core shell | 1294 RPEQELAARYLVDNQ |
| BA71V-CP2475L (P220) | BA71V-CP2475L (p220) | Core shell | 1295 PEQELAARYLVDNQR |
| BA71V-CP2475L (P220) | BA71V-CP2475L (p220) | Core shell | 1296 EQELAARYLVDNQRI |
| BA71V-CP2475L (P220) | BA71V-CP2475L (p220) | Core shell | 1297 QELAARYLVDNQRIM |
| BA71V-CP2475L (P220) | BA71V-CP2475L (p220) | Core shell | 1298 ELAARYLVDNQRIMQ |
| BA71V-CP2475L (P220) | BA71V-CP2475L (p220) | Core shell | 1299 LAARYLVDNQRIMQL |
| BA71V-CP2475L (P220) | BA71V-CP2475L (p220) | Core shell | 1300 AARYLVDNQRIMQLL |
| BA71V-CP2475L (P220) | BA71V-CP2475L (p220) | Core shell | 1301 ARYLVDNQRIMQLLL |
| BA71V-CP2475L (P220) | BA71V-CP2475L (p220) | Core shell | 1302 RYLVDNQRIMQLLLT |
| BA71V-CP2475L (P220) | BA71V-CP2475L (p220) | Core shell | 1303 YLVDNQRIMQLLLTN |
| BA71V-CP2475L (P220) | BA71V-CP2475L (p220) | Core shell | 1304 LVDNQRIMQLLLTNI |
| BA71V-CP2475L (P220) | BA71V-CP2475L (p220) | Core shell | 1305 VDNQRIMQLLLTNIF |
| BA71V-CP2475L (P220) | BA71V-CP2475L (p220) | Core shell | 1306 DNQRIMQLLLTNIFE |
| BA71V-CP2475L (P220) | BA71V-CP2475L (p220) | Core shell | 1307 NQRIMQLLLTNIFEM |
| BA71V-CP2475L (P220) | BA71V-CP2475L (p220) | Core shell | 1308 QRIMQLLLTNIFEMT |
| BA71V-CP2475L (P220) | BA71V-CP2475L (p220) | Core shell | 1309 RIMQLLLTNIFEMTS |
| BA71V-CP2475L (P220) | BA71V-CP2475L (p220) | Core shell | 1310 IMQLLLTNIFEMTSS |
| BA71V-CP2475L (P220) | BA71V-CP2475L (p220) | Core shell | 1311 MQLLLTNIFEMTSSF |
| BA71V-CP2475L (P220) | BA71V-CP2475L (p220) | Core shell | 1312 QLLLTNIFEMTSSFN |
| BA71V-CP2475L (P220) | BA71V-CP2475L (p220) | Core shell | 1313 LLLTNIFEMTSSFNK |
| BA71V-CP2475L (P220) | BA71V-CP2475L (p220) | Core shell | 1314 LLTNIFEMTSSFNKL |
| BA71V-CP2475L (P220) | BA71V-CP2475L (p220) | Core shell | 1315 LTNIFEMTSSFNKLV |
| BA71V-CP2475L (P220) | BA71V-CP2475L (p220) | Core shell | 1316 TNIFEMTSSFNKLVQ |
| BA71V-CP2475L (P220) | BA71V-CP2475L (p220) | Core shell | 1317 NIFEMTSSFNKLVQV |
| BA71V-CP2475L (P220) | BA71V-CP2475L (p220) | Core shell | 1318 IFEMTSSFNKLVQVR |
| BA71V-CP2475L (P220) | BA71V-CP2475L (p220) | Core shell | 1319 FEMTSSFNKLVQVRF |
| BA71V-CP2475L (P220) | BA71V-CP2475L (p220) | Core shell | 1320 EMTSSFNKLVQVRFP |
| BA71V-CP2475L (P220) | BA71V-CP2475L (p220) | Core shell | 1321 MTSSFNKLVQVRFPE |
| BA71V-CP2475L (P220) | BA71V-CP2475L (p220) | Core shell | 1322 TSSFNKLVQVRFPET |
| BA71V-CP2475L (P220) | BA71V-CP2475L (p220) | Core shell | 1323 SSFNKLVQVRFPETS |
| BA71V-CP2475L (P220) | BA71V-CP2475L (p220) | Core shell | 1324 SFNKLVQVRFPETST |
| BA71V-CP2475L (P220) | BA71V-CP2475L (p220) | Core shell | 1325 FNKLVQVRFPETSTA |
| BA71V-CP2475L (P220) | BA71V-CP2475L (p220) | Core shell | 1326 NKLVQVRFPETSTAH |
| BA71V-CP2475L (P220) | BA71V-CP2475L (p220) | Core shell | 1327 KLVQVRFPETSTAHV |
| BA71V-CP2475L (P220) | BA71V-CP2475L (p220) | Core shell | 1328 LVQVRFPETSTAHVH |
| BA71V-CP2475L (P220) | BA71V-CP2475L (p220) | Core shell | 1329 VQVRFPETSTAHVHL |
| BA71V-CP2475L (P220) | BA71V-CP2475L (p220) | Core shell | 1330 QVRFPETSTAHVHLD |
| BA71V-CP2475L (P220) | BA71V-CP2475L (p220) | Core shell | 1331 VRFPETSTAHVHLDF |
| BA71V-CP2475L (P220) | BA71V-CP2475L (p220) | Core shell | 1332 RFPETSTAHVHLDFT |
| BA71V-CP2475L (P220) | BA71V-CP2475L (p220) | Core shell | 1333 FPETSTAHVHLDFTG |
| BA71V-CP2475L (P220) | BA71V-CP2475L (p220) | Core shell | 1334 PETSTAHVHLDFTGL |
| BA71V-CP2475L (P220) | BA71V-CP2475L (p220) | Core shell | 1335 ETSTAHVHLDFTGLI |
| BA71V-CP2475L (P220) | BA71V-CP2475L (p220) | Core shell | 1336 TSTAHVHLDFTGLIS |
| BA71V-CP2475L (P220) | BA71V-CP2475L (p220) | Core shell | 1337 STAHVHLDFTGLISL |
| BA71V-CP2475L (P220) | BA71V-CP2475L (p220) | Core shell | 1338 TAHVHLDFTGLISLI |
| BA71V-CP2475L (P220) | BA71V-CP2475L (p220) | Core shell | 1339 AHVHLDFTGLISLID |
| BA71V-CP2475L (P220) | BA71V-CP2475L (p220) | Core shell | 1340 HVHLDFTGLISLIDS |
| BA71V-CP2475L (P220) | BA71V-CP2475L (p220) | Core shell | 1341 VHLDFTGLISLIDSL |
| BA71V-CP2475L (P220) | BA71V-CP2475L (p220) | Core shell | 1342 HLDFTGLISLIDSLM |
| BA71V-CP2475L (P220) | BA71V-CP2475L (p220) | Core shell | 1343 LDFTGLISLIDSLMA |
| BA71V-CP2475L (P220) | BA71V-CP2475L (p220) | Core shell | 1344 DFTGLISLIDSLMAD |
| BA71V-CP2475L (P220) | BA71V-CP2475L (p220) | Core shell | 1345 FTGLISLIDSLMADT |
| BA71V-CP2475L (P220) | BA71V-CP2475L (p220) | Core shell | 1346 TGLISLIDSLMADTK |
| BA71V-CP2475L (P220) | BA71V-CP2475L (p220) | Core shell | 1347 GLISLIDSLMADTKY |
| BA71V-CP2475L (P220) | BA71V-CP2475L (p220) | Core shell | 1348 LISLIDSLMADTKYF |
| BA71V-CP2475L (P220) | BA71V-CP2475L (p220) | Core shell | 1349 ISLIDSLMADTKYFL |
| BA71V-CP2475L (P220) | BA71V-CP2475L (p220) | Core shell | 1350 SLIDSLMADTKYFLD |
| BA71V-CP2475L (P220) | BA71V-CP2475L (p220) | Core shell | 1351 LIDSLMADTKYFLDL |
| BA71V-CP2475L (P220) | BA71V-CP2475L (p220) | Core shell | 1352 IDSLMADTKYFLDLL |
| BA71V-CP2475L (P220) | BA71V-CP2475L (p220) | Core shell | 1353 DSLMADTKYFLDLLR |
| BA71V-CP2475L (P220) | BA71V-CP2475L (p220) | Core shell | 1354 SLMADTKYFLDLLRP |
| BA71V-CP2475L (P220) | BA71V-CP2475L (p220) | Core shell | 1355 LMADTKYFLDLLRPH |
| BA71V-CP2475L (P220) | BA71V-CP2475L (p220) | Core shell | 1356 MADTKYFLDLLRPHI |
| BA71V-CP2475L (P220) | BA71V-CP2475L (p220) | Core shell | 1357 ADTKYFLDLLRPHID |
| BA71V-CP2475L (P220) | BA71V-CP2475L (p220) | Core shell | 1358 DTKYFLDLLRPHIDK |

| | | | | |
|---|---|---|---|---|
| BA71V-CP2475L (P220) | BA71V-CP2475L (p220) | Core shell | 1359 | TKYFLDLLRPHIDKN |
| BA71V-CP2475L (P220) | BA71V-CP2475L (p220) | Core shell | 1360 | KYFLDLLRPHIDKNI |
| BA71V-CP2475L (P220) | BA71V-CP2475L (p220) | Core shell | 1361 | YFLDLLRPHIDKNII |
| BA71V-CP2475L (P220) | BA71V-CP2475L (p220) | Core shell | 1362 | FLDLLRPHIDKNIIQ |
| BA71V-CP2475L (P220) | BA71V-CP2475L (p220) | Core shell | 1363 | LDLLRPHIDKNIIQY |
| BA71V-CP2475L (P220) | BA71V-CP2475L (p220) | Core shell | 1364 | DLLRPHIDKNIIQYY |
| BA71V-CP2475L (P220) | BA71V-CP2475L (p220) | Core shell | 1365 | LLRPHIDKNIIQYYE |
| BA71V-CP2475L (P220) | BA71V-CP2475L (p220) | Core shell | 1366 | LRPHIDKNIIQYYEN |
| BA71V-CP2475L (P220) | BA71V-CP2475L (p220) | Core shell | 1367 | RPHIDKNIIQYYENR |
| BA71V-CP2475L (P220) | BA71V-CP2475L (p220) | Core shell | 1368 | PHIDKNIIQYYENRS |
| BA71V-CP2475L (P220) | BA71V-CP2475L (p220) | Core shell | 1369 | HIDKNIIQYYENRSN |
| BA71V-CP2475L (P220) | BA71V-CP2475L (p220) | Core shell | 1370 | IDKNIIQYYENRSNP |
| BA71V-CP2475L (P220) | BA71V-CP2475L (p220) | Core shell | 1371 | DKNIIQYYENRSNPG |
| BA71V-CP2475L (P220) | BA71V-CP2475L (p220) | Core shell | 1372 | KNIIQYYENRSNPGS |
| BA71V-CP2475L (P220) | BA71V-CP2475L (p220) | Core shell | 1373 | NIIQYYENRSNPGSF |
| BA71V-CP2475L (P220) | BA71V-CP2475L (p220) | Core shell | 1374 | IIQYYENRSNPGSFY |
| BA71V-CP2475L (P220) | BA71V-CP2475L (p220) | Core shell | 1375 | IQYYENRSNPGSFYW |
| BA71V-CP2475L (P220) | BA71V-CP2475L (p220) | Core shell | 1376 | QYYENRSNPGSFYWL |
| BA71V-CP2475L (P220) | BA71V-CP2475L (p220) | Core shell | 1377 | YYENRSNPGSFYWLE |
| BA71V-CP2475L (P220) | BA71V-CP2475L (p220) | Core shell | 1378 | YENRSNPGSFYWLEE |
| BA71V-CP2475L (P220) | BA71V-CP2475L (p220) | Core shell | 1379 | ENRSNPGSFYWLEEH |
| BA71V-CP2475L (P220) | BA71V-CP2475L (p220) | Core shell | 1380 | NRSNPGSFYWLEEHL |
| BA71V-CP2475L (P220) | BA71V-CP2475L (p220) | Core shell | 1381 | RSNPGSFYWLEEHLI |
| BA71V-CP2475L (P220) | BA71V-CP2475L (p220) | Core shell | 1382 | SNPGSFYWLEEHLID |
| BA71V-CP2475L (P220) | BA71V-CP2475L (p220) | Core shell | 1383 | NPGSFYWLEEHLIDK |
| BA71V-CP2475L (P220) | BA71V-CP2475L (p220) | Core shell | 1384 | PGSFYWLEEHLIDKL |
| BA71V-CP2475L (P220) | BA71V-CP2475L (p220) | Core shell | 1385 | GSFYWLEEHLIDKLI |
| BA71V-CP2475L (P220) | BA71V-CP2475L (p220) | Core shell | 1386 | SFYWLEEHLIDKLIK |
| BA71V-CP2475L (P220) | BA71V-CP2475L (p220) | Core shell | 1387 | FYWLEEHLIDKLIKP |
| BA71V-CP2475L (P220) | BA71V-CP2475L (p220) | Core shell | 1388 | YWLEEHLIDKLIKPP |
| BA71V-CP2475L (P220) | BA71V-CP2475L (p220) | Core shell | 1389 | WLEEHLIDKLIKPPT |
| BA71V-CP2475L (P220) | BA71V-CP2475L (p220) | Core shell | 1390 | LEEHLIDKLIKPPTD |
| BA71V-CP2475L (P220) | BA71V-CP2475L (p220) | Core shell | 1391 | EEHLIDKLIKPPTDA |
| BA71V-CP2475L (P220) | BA71V-CP2475L (p220) | Core shell | 1392 | EHLIDKLIKPPTDAG |
| BA71V-CP2475L (P220) | BA71V-CP2475L (p220) | Core shell | 1393 | HLIDKLIKPPTDAGG |
| BA71V-CP2475L (P220) | BA71V-CP2475L (p220) | Core shell | 1394 | LIDKLIKPPTDAGGR |
| BA71V-CP2475L (P220) | BA71V-CP2475L (p220) | Core shell | 1395 | IDKLIKPPTDAGGRP |
| BA71V-CP2475L (P220) | BA71V-CP2475L (p220) | Core shell | 1396 | DKLIKPPTDAGGRPL |
| BA71V-CP2475L (P220) | BA71V-CP2475L (p220) | Core shell | 1397 | KLIKPPTDAGGRPLP |
| BA71V-CP2475L (P220) | BA71V-CP2475L (p220) | Core shell | 1398 | LIKPPTDAGGRPLPG |
| BA71V-CP2475L (P220) | BA71V-CP2475L (p220) | Core shell | 1399 | IKPPTDAGGRPLPGG |
| BA71V-CP2475L (P220) | BA71V-CP2475L (p220) | Core shell | 1400 | KPPTDAGGRPLPGGE |
| BA71V-CP2475L (P220) | BA71V-CP2475L (p220) | Core shell | 1401 | PPTDAGGRPLPGGEL |
| BA71V-CP2475L (P220) | BA71V-CP2475L (p220) | Core shell | 1402 | PTDAGGRPLPGGELG |
| BA71V-CP2475L (P220) | BA71V-CP2475L (p220) | Core shell | 1403 | TDAGGRPLPGGELGL |
| BA71V-CP2475L (P220) | BA71V-CP2475L (p220) | Core shell | 1404 | DAGGRPLPGGELGLE |
| BA71V-CP2475L (P220) | BA71V-CP2475L (p220) | Core shell | 1405 | AGGRPLPGGELGLEG |
| BA71V-CP2475L (P220) | BA71V-CP2475L (p220) | Core shell | 1406 | GGRPLPGGELGLEGV |
| BA71V-CP2475L (P220) | BA71V-CP2475L (p220) | Core shell | 1407 | GRPLPGGELGLEGVN |
| BA71V-CP2475L (P220) | BA71V-CP2475L (p220) | Core shell | 1408 | RPLPGGELGLEGVNQ |
| BA71V-CP2475L (P220) | BA71V-CP2475L (p220) | Core shell | 1409 | PLPGGELGLEGVNQI |
| BA71V-CP2475L (P220) | BA71V-CP2475L (p220) | Core shell | 1410 | LPGGELGLEGVNQII |
| BA71V-CP2475L (P220) | BA71V-CP2475L (p220) | Core shell | 1411 | PGGELGLEGVNQIIN |
| BA71V-CP2475L (P220) | BA71V-CP2475L (p220) | Core shell | 1412 | GGELGLEGVNQIINK |
| BA71V-CP2475L (P220) | BA71V-CP2475L (p220) | Core shell | 1413 | GELGLEGVNQIINKT |
| BA71V-CP2475L (P220) | BA71V-CP2475L (p220) | Core shell | 1414 | ELGLEGVNQIINKTY |
| BA71V-CP2475L (P220) | BA71V-CP2475L (p220) | Core shell | 1415 | LGLEGVNQIINKTYI |
| BA71V-CP2475L (P220) | BA71V-CP2475L (p220) | Core shell | 1416 | GLEGVNQIINKTYIL |
| BA71V-CP2475L (P220) | BA71V-CP2475L (p220) | Core shell | 1417 | LEGVNQIINKTYILL |
| BA71V-CP2475L (P220) | BA71V-CP2475L (p220) | Core shell | 1418 | EGVNQIINKTYILLT |
| BA71V-CP2475L (P220) | BA71V-CP2475L (p220) | Core shell | 1419 | GVNQIINKTYILLTK |
| BA71V-CP2475L (P220) | BA71V-CP2475L (p220) | Core shell | 1420 | VNQIINKTYILLTKP |
| BA71V-CP2475L (P220) | BA71V-CP2475L (p220) | Core shell | 1421 | NQIINKTYILLTKPY |
| BA71V-CP2475L (P220) | BA71V-CP2475L (p220) | Core shell | 1422 | QIINKTYILLTKPYN |
| BA71V-CP2475L (P220) | BA71V-CP2475L (p220) | Core shell | 1423 | IINKTYILLTKPYNV |
| BA71V-CP2475L (P220) | BA71V-CP2475L (p220) | Core shell | 1424 | INKTYILLTKPYNVL |
| BA71V-CP2475L (P220) | BA71V-CP2475L (p220) | Core shell | 1425 | NKTYILLTKPYNVLQ |

| | | | | |
|---|---|---|---|---|
| BA71V-CP2475L (P220) | BA71V-CP2475L (p220) | Core shell | 1426 | KTYILLTKPYNVLQL |
| BA71V-CP2475L (P220) | BA71V-CP2475L (p220) | Core shell | 1427 | TYILLTKPYNVLQLR |
| BA71V-CP2475L (P220) | BA71V-CP2475L (p220) | Core shell | 1428 | YILLTKPYNVLQLRG |
| BA71V-CP2475L (P220) | BA71V-CP2475L (p220) | Core shell | 1429 | ILLTKPYNVLQLRGG |
| BA71V-CP2475L (P220) | BA71V-CP2475L (p220) | Core shell | 1430 | LLTKPYNVLQLRGGA |
| BA71V-CP2475L (P220) | BA71V-CP2475L (p220) | Core shell | 1431 | LTKPYNVLQLRGGAQ |
| BA71V-CP2475L (P220) | BA71V-CP2475L (p220) | Core shell | 1432 | TKPYNVLQLRGGAQR |
| BA71V-CP2475L (P220) | BA71V-CP2475L (p220) | Core shell | 1433 | KPYNVLQLRGGAQRG |
| BA71V-CP2475L (P220) | BA71V-CP2475L (p220) | Core shell | 1434 | PYNVLQLRGGAQRGN |
| BA71V-CP2475L (P220) | BA71V-CP2475L (p220) | Core shell | 1435 | YNVLQLRGGAQRGNA |
| BA71V-CP2475L (P220) | BA71V-CP2475L (p220) | Core shell | 1436 | NVLQLRGGAQRGNAA |
| BA71V-CP2475L (P220) | BA71V-CP2475L (p220) | Core shell | 1437 | VLQLRGGAQRGNAAN |
| BA71V-CP2475L (P220) | BA71V-CP2475L (p220) | Core shell | 1438 | LQLRGGAQRGNAANI |
| BA71V-CP2475L (P220) | BA71V-CP2475L (p220) | Core shell | 1439 | QLRGGAQRGNAANIQ |
| BA71V-CP2475L (P220) | BA71V-CP2475L (p220) | Core shell | 1440 | LRGGAQRGNAANIQI |
| BA71V-CP2475L (P220) | BA71V-CP2475L (p220) | Core shell | 1441 | RGGAQRGNAANIQIN |
| BA71V-CP2475L (P220) | BA71V-CP2475L (p220) | Core shell | 1442 | GGAQRGNAANIQINN |
| BA71V-CP2475L (P220) | BA71V-CP2475L (p220) | Core shell | 1443 | GAQRGNAANIQINNN |
| BA71V-CP2475L (P220) | BA71V-CP2475L (p220) | Core shell | 1444 | AQRGNAANIQINNNP |
| BA71V-CP2475L (P220) | BA71V-CP2475L (p220) | Core shell | 1445 | QRGNAANIQINNNPE |
| BA71V-CP2475L (P220) | BA71V-CP2475L (p220) | Core shell | 1446 | RGNAANIQINNNPEL |
| BA71V-CP2475L (P220) | BA71V-CP2475L (p220) | Core shell | 1447 | GNAANIQINNNPELS |
| BA71V-CP2475L (P220) | BA71V-CP2475L (p220) | Core shell | 1448 | NAANIQINNNPELSE |
| BA71V-CP2475L (P220) | BA71V-CP2475L (p220) | Core shell | 1449 | AANIQINNNPELSER |
| BA71V-CP2475L (P220) | BA71V-CP2475L (p220) | Core shell | 1450 | ANIQINNNPELSERY |
| BA71V-CP2475L (P220) | BA71V-CP2475L (p220) | Core shell | 1451 | NIQINNNPELSERYE |
| BA71V-CP2475L (P220) | BA71V-CP2475L (p220) | Core shell | 1452 | IQINNNPELSERYEQ |
| BA71V-CP2475L (P220) | BA71V-CP2475L (p220) | Core shell | 1453 | QINNNPELSERYEQY |
| BA71V-CP2475L (P220) | BA71V-CP2475L (p220) | Core shell | 1454 | INNNPELSERYEQYG |
| BA71V-CP2475L (P220) | BA71V-CP2475L (p220) | Core shell | 1455 | NNNPELSERYEQYGR |
| BA71V-CP2475L (P220) | BA71V-CP2475L (p220) | Core shell | 1456 | NNPELSERYEQYGRV |
| BA71V-CP2475L (P220) | BA71V-CP2475L (p220) | Core shell | 1457 | NPELSERYEQYGRVF |
| BA71V-CP2475L (P220) | BA71V-CP2475L (p220) | Core shell | 1458 | PELSERYEQYGRVFS |
| BA71V-CP2475L (P220) | BA71V-CP2475L (p220) | Core shell | 1459 | ELSERYEQYGRVFSR |
| BA71V-CP2475L (P220) | BA71V-CP2475L (p220) | Core shell | 1460 | LSERYEQYGRVFSRL |
| BA71V-CP2475L (P220) | BA71V-CP2475L (p220) | Core shell | 1461 | SERYEQYGRVFSRLV |
| BA71V-CP2475L (P220) | BA71V-CP2475L (p220) | Core shell | 1462 | ERYEQYGRVFSRLVF |
| BA71V-CP2475L (P220) | BA71V-CP2475L (p220) | Core shell | 1463 | RYEQYGRVFSRLVFY |
| BA71V-CP2475L (P220) | BA71V-CP2475L (p220) | Core shell | 1464 | YEQYGRVFSRLVFYD |
| BA71V-CP2475L (P220) | BA71V-CP2475L (p220) | Core shell | 1465 | EQYGRVFSRLVFYDA |
| BA71V-CP2475L (P220) | BA71V-CP2475L (p220) | Core shell | 1466 | QYGRVFSRLVFYDAL |
| BA71V-CP2475L (P220) | BA71V-CP2475L (p220) | Core shell | 1467 | YGRVFSRLVFYDALI |
| BA71V-CP2475L (P220) | BA71V-CP2475L (p220) | Core shell | 1468 | GRVFSRLVFYDALIE |
| BA71V-CP2475L (P220) | BA71V-CP2475L (p220) | Core shell | 1469 | RVFSRLVFYDALIEN |
| BA71V-CP2475L (P220) | BA71V-CP2475L (p220) | Core shell | 1470 | VFSRLVFYDALIENS |
| BA71V-CP2475L (P220) | BA71V-CP2475L (p220) | Core shell | 1471 | FSRLVFYDALIENSG |
| BA71V-CP2475L (P220) | BA71V-CP2475L (p220) | Core shell | 1472 | SRLVFYDALIENSGL |
| BA71V-CP2475L (P220) | BA71V-CP2475L (p220) | Core shell | 1473 | RLVFYDALIENSGLR |
| BA71V-CP2475L (P220) | BA71V-CP2475L (p220) | Core shell | 1474 | LVFYDALIENSGLRV |
| BA71V-CP2475L (P220) | BA71V-CP2475L (p220) | Core shell | 1475 | VFYDALIENSGLRVE |
| BA71V-CP2475L (P220) | BA71V-CP2475L (p220) | Core shell | 1476 | FYDALIENSGLRVEQ |
| BA71V-CP2475L (P220) | BA71V-CP2475L (p220) | Core shell | 1477 | YDALIENSGLRVEQV |
| BA71V-CP2475L (P220) | BA71V-CP2475L (p220) | Core shell | 1478 | DALIENSGLRVEQVA |
| BA71V-CP2475L (P220) | BA71V-CP2475L (p220) | Core shell | 1479 | ALIENSGLRVEQVAL |
| BA71V-CP2475L (P220) | BA71V-CP2475L (p220) | Core shell | 1480 | LIENSGLRVEQVALG |
| BA71V-CP2475L (P220) | BA71V-CP2475L (p220) | Core shell | 1481 | IENSGLRVEQVALGD |
| BA71V-CP2475L (P220) | BA71V-CP2475L (p220) | Core shell | 1482 | ENSGLRVEQVALGDF |
| BA71V-CP2475L (P220) | BA71V-CP2475L (p220) | Core shell | 1483 | NSGLRVEQVALGDFR |
| BA71V-CP2475L (P220) | BA71V-CP2475L (p220) | Core shell | 1484 | SGLRVEQVALGDFRL |
| BA71V-CP2475L (P220) | BA71V-CP2475L (p220) | Core shell | 1485 | GLRVEQVALGDFRLS |
| BA71V-CP2475L (P220) | BA71V-CP2475L (p220) | Core shell | 1486 | LRVEQVALGDFRLSN |
| BA71V-CP2475L (P220) | BA71V-CP2475L (p220) | Core shell | 1487 | RVEQVALGDFRLSNL |
| BA71V-CP2475L (P220) | BA71V-CP2475L (p220) | Core shell | 1488 | VEQVALGDFRLSNLV |
| BA71V-CP2475L (P220) | BA71V-CP2475L (p220) | Core shell | 1489 | EQVALGDFRLSNLVR |
| BA71V-CP2475L (P220) | BA71V-CP2475L (p220) | Core shell | 1490 | QVALGDFRLSNLVRT |
| BA71V-CP2475L (P220) | BA71V-CP2475L (p220) | Core shell | 1491 | VALGDFRLSNLVRTN |
| BA71V-CP2475L (P220) | BA71V-CP2475L (p220) | Core shell | 1492 | ALGDFRLSNLVRTNN |

| | | | | |
|---|---|---|---|---|
| BA71V-CP2475L (P220) | BA71V-CP2475L (p220) | Core shell | 1493 | LGDFRLSNLVRTNNA |
| BA71V-CP2475L (P220) | BA71V-CP2475L (p220) | Core shell | 1494 | GDFRLSNLVRTNNAQ |
| BA71V-CP2475L (P220) | BA71V-CP2475L (p220) | Core shell | 1495 | DFRLSNLVRTNNAQE |
| BA71V-CP2475L (P220) | BA71V-CP2475L (p220) | Core shell | 1496 | FRLSNLVRTNNAQEE |
| BA71V-CP2475L (P220) | BA71V-CP2475L (p220) | Core shell | 1497 | RLSNLVRTNNAQEEN |
| BA71V-CP2475L (P220) | BA71V-CP2475L (p220) | Core shell | 1498 | LSNLVRTNNAQEENA |
| BA71V-CP2475L (P220) | BA71V-CP2475L (p220) | Core shell | 1499 | SNLVRTNNAQEENAL |
| BA71V-CP2475L (P220) | BA71V-CP2475L (p220) | Core shell | 1500 | NLVRTNNAQEENALS |
| BA71V-CP2475L (P220) | BA71V-CP2475L (p220) | Core shell | 1501 | LVRTNNAQEENALSF |
| BA71V-CP2475L (P220) | BA71V-CP2475L (p220) | Core shell | 1502 | VRTNNAQEENALSFW |
| BA71V-CP2475L (P220) | BA71V-CP2475L (p220) | Core shell | 1503 | RTNNAQEENALSFWT |
| BA71V-CP2475L (P220) | BA71V-CP2475L (p220) | Core shell | 1504 | TNNAQEENALSFWTA |
| BA71V-CP2475L (P220) | BA71V-CP2475L (p220) | Core shell | 1505 | NNAQEENALSFWTAV |
| BA71V-CP2475L (P220) | BA71V-CP2475L (p220) | Core shell | 1506 | NAQEENALSFWTAVA |
| BA71V-CP2475L (P220) | BA71V-CP2475L (p220) | Core shell | 1507 | AQEENALSFWTAVAP |
| BA71V-CP2475L (P220) | BA71V-CP2475L (p220) | Core shell | 1508 | QEENALSFWTAVAPR |
| BA71V-CP2475L (P220) | BA71V-CP2475L (p220) | Core shell | 1509 | EENALSFWTAVAPRA |
| BA71V-CP2475L (P220) | BA71V-CP2475L (p220) | Core shell | 1510 | ENALSFWTAVAPRAY |
| BA71V-CP2475L (P220) | BA71V-CP2475L (p220) | Core shell | 1511 | NALSFWTAVAPRAYA |
| BA71V-CP2475L (P220) | BA71V-CP2475L (p220) | Core shell | 1512 | ALSFWTAVAPRAYAN |
| BA71V-CP2475L (P220) | BA71V-CP2475L (p220) | Core shell | 1513 | LSFWTAVAPRAYANV |
| BA71V-CP2475L (P220) | BA71V-CP2475L (p220) | Core shell | 1514 | SFWTAVAPRAYANVN |
| BA71V-CP2475L (P220) | BA71V-CP2475L (p220) | Core shell | 1515 | FWTAVAPRAYANVND |
| BA71V-CP2475L (P220) | BA71V-CP2475L (p220) | Core shell | 1516 | WTAVAPRAYANVNDA |
| BA71V-CP2475L (P220) | BA71V-CP2475L (p220) | Core shell | 1517 | TAVAPRAYANVNDAA |
| BA71V-CP2475L (P220) | BA71V-CP2475L (p220) | Core shell | 1518 | AVAPRAYANVNDAAN |
| BA71V-CP2475L (P220) | BA71V-CP2475L (p220) | Core shell | 1519 | VAPRAYANVNDAANN |
| BA71V-CP2475L (P220) | BA71V-CP2475L (p220) | Core shell | 1520 | APRAYANVNDAANNL |
| BA71V-CP2475L (P220) | BA71V-CP2475L (p220) | Core shell | 1521 | PRAYANVNDAANNLR |
| BA71V-CP2475L (P220) | BA71V-CP2475L (p220) | Core shell | 1522 | RAYANVNDAANNLRR |
| BA71V-CP2475L (P220) | BA71V-CP2475L (p220) | Core shell | 1523 | AYANVNDAANNLRRY |
| BA71V-CP2475L (P220) | BA71V-CP2475L (p220) | Core shell | 1524 | YANVNDAANNLRRYR |
| BA71V-CP2475L (P220) | BA71V-CP2475L (p220) | Core shell | 1525 | ANVNDAANNLRRYRL |
| BA71V-CP2475L (P220) | BA71V-CP2475L (p220) | Core shell | 1526 | NVNDAANNLRRYRLY |
| BA71V-CP2475L (P220) | BA71V-CP2475L (p220) | Core shell | 1527 | VNDAANNLRRYRLYG |
| BA71V-CP2475L (P220) | BA71V-CP2475L (p220) | Core shell | 1528 | NDAANNLRRYRLYGS |
| BA71V-CP2475L (P220) | BA71V-CP2475L (p220) | Core shell | 1529 | DAANNLRRYRLYGSD |
| BA71V-CP2475L (P220) | BA71V-CP2475L (p220) | Core shell | 1530 | AANNLRRYRLYGSDY |
| BA71V-CP2475L (P220) | BA71V-CP2475L (p220) | Core shell | 1531 | ANNLRRYRLYGSDYG |
| BA71V-CP2475L (P220) | BA71V-CP2475L (p220) | Core shell | 1532 | NNLRRYRLYGSDYGI |
| BA71V-CP2475L (P220) | BA71V-CP2475L (p220) | Core shell | 1533 | NLRRYRLYGSDYGIR |
| BA71V-CP2475L (P220) | BA71V-CP2475L (p220) | Core shell | 1534 | LRRYRLYGSDYGIRN |
| BA71V-CP2475L (P220) | BA71V-CP2475L (p220) | Core shell | 1535 | RRYRLYGSDYGIRNN |
| BA71V-CP2475L (P220) | BA71V-CP2475L (p220) | Core shell | 1536 | RYRLYGSDYGIRNNR |
| BA71V-CP2475L (P220) | BA71V-CP2475L (p220) | Core shell | 1537 | YRLYGSDYGIRNNRS |
| BA71V-CP2475L (P220) | BA71V-CP2475L (p220) | Core shell | 1538 | RLYGSDYGIRNNRSM |
| BA71V-CP2475L (P220) | BA71V-CP2475L (p220) | Core shell | 1539 | LYGSDYGIRNNRSMM |
| BA71V-CP2475L (P220) | BA71V-CP2475L (p220) | Core shell | 1540 | YGSDYGIRNNRSMMM |
| BA71V-CP2475L (P220) | BA71V-CP2475L (p220) | Core shell | 1541 | GSDYGIRNNRSMMMV |
| BA71V-CP2475L (P220) | BA71V-CP2475L (p220) | Core shell | 1542 | SDYGIRNNRSMMMVF |
| BA71V-CP2475L (P220) | BA71V-CP2475L (p220) | Core shell | 1543 | DYGIRNNRSMMMVFN |
| BA71V-CP2475L (P220) | BA71V-CP2475L (p220) | Core shell | 1544 | YGIRNNRSMMMVFNQ |
| BA71V-CP2475L (P220) | BA71V-CP2475L (p220) | Core shell | 1545 | GIRNNRSMMMVFNQL |
| BA71V-CP2475L (P220) | BA71V-CP2475L (p220) | Core shell | 1546 | IRNNRSMMMVFNQLV |
| BA71V-CP2475L (P220) | BA71V-CP2475L (p220) | Core shell | 1547 | RNNRSMMMVFNQLVA |
| BA71V-CP2475L (P220) | BA71V-CP2475L (p220) | Core shell | 1548 | NNRSMMMVFNQLVAS |
| BA71V-CP2475L (P220) | BA71V-CP2475L (p220) | Core shell | 1549 | NRSMMMVFNQLVASY |
| BA71V-CP2475L (P220) | BA71V-CP2475L (p220) | Core shell | 1550 | RSMMMVFNQLVASYI |
| BA71V-CP2475L (P220) | BA71V-CP2475L (p220) | Core shell | 1551 | SMMMVFNQLVASYIA |
| BA71V-CP2475L (P220) | BA71V-CP2475L (p220) | Core shell | 1552 | MMMVFNQLVASYIAR |
| BA71V-CP2475L (P220) | BA71V-CP2475L (p220) | Core shell | 1553 | MMVFNQLVASYIARF |
| BA71V-CP2475L (P220) | BA71V-CP2475L (p220) | Core shell | 1554 | MVFNQLVASYIARFY |
| BA71V-CP2475L (P220) | BA71V-CP2475L (p220) | Core shell | 1555 | VFNQLVASYIARFYD |
| BA71V-CP2475L (P220) | BA71V-CP2475L (p220) | Core shell | 1556 | FNQLVASYIARFYDA |
| BA71V-CP2475L (P220) | BA71V-CP2475L (p220) | Core shell | 1557 | NQLVASYIARFYDAP |
| BA71V-CP2475L (P220) | BA71V-CP2475L (p220) | Core shell | 1558 | QLVASYIARFYDAPS |
| BA71V-CP2475L (P220) | BA71V-CP2475L (p220) | Core shell | 1559 | LVASYIARFYDAPSG |

| | | | |
|---|---|---|---|
| BA71V-CP2475L (P220) | BA71V-CP2475L (p220) | Core shell | 1560 VASYIARFYDAPSGK |
| BA71V-CP2475L (P220) | BA71V-CP2475L (p220) | Core shell | 1561 ASYIARFYDAPSGKI |
| BA71V-CP2475L (P220) | BA71V-CP2475L (p220) | Core shell | 1562 SYIARFYDAPSGKIY |
| BA71V-CP2475L (P220) | BA71V-CP2475L (p220) | Core shell | 1563 YIARFYDAPSGKIYL |
| BA71V-CP2475L (P220) | BA71V-CP2475L (p220) | Core shell | 1564 IARFYDAPSGKIYLN |
| BA71V-CP2475L (P220) | BA71V-CP2475L (p220) | Core shell | 1565 ARFYDAPSGKIYLNL |
| BA71V-CP2475L (P220) | BA71V-CP2475L (p220) | Core shell | 1566 RFYDAPSGKIYLNLI |
| BA71V-CP2475L (P220) | BA71V-CP2475L (p220) | Core shell | 1567 FYDAPSGKIYLNLIN |
| BA71V-CP2475L (P220) | BA71V-CP2475L (p220) | Core shell | 1568 YDAPSGKIYLNLINA |
| BA71V-CP2475L (P220) | BA71V-CP2475L (p220) | Core shell | 1569 DAPSGKIYLNLINAF |
| BA71V-CP2475L (P220) | BA71V-CP2475L (p220) | Core shell | 1570 APSGKIYLNLINAFA |
| BA71V-CP2475L (P220) | BA71V-CP2475L (p220) | Core shell | 1571 PSGKIYLNLINAFAN |
| BA71V-CP2475L (P220) | BA71V-CP2475L (p220) | Core shell | 1572 SGKIYLNLINAFANG |
| BA71V-CP2475L (P220) | BA71V-CP2475L (p220) | Core shell | 1573 GKIYLNLINAFANGN |
| BA71V-CP2475L (P220) | BA71V-CP2475L (p220) | Core shell | 1574 KIYLNLINAFANGNF |
| BA71V-CP2475L (P220) | BA71V-CP2475L (p220) | Core shell | 1575 IYLNLINAFANGNFS |
| BA71V-CP2475L (P220) | BA71V-CP2475L (p220) | Core shell | 1576 YLNLINAFANGNFSQ |
| BA71V-CP2475L (P220) | BA71V-CP2475L (p220) | Core shell | 1577 LNLINAFANGNFSQA |
| BA71V-CP2475L (P220) | BA71V-CP2475L (p220) | Core shell | 1578 NLINAFANGNFSQAV |
| BA71V-CP2475L (P220) | BA71V-CP2475L (p220) | Core shell | 1579 LINAFANGNFSQAVM |
| BA71V-CP2475L (P220) | BA71V-CP2475L (p220) | Core shell | 1580 INAFANGNFSQAVME |
| BA71V-CP2475L (P220) | BA71V-CP2475L (p220) | Core shell | 1581 NAFANGNFSQAVMEL |
| BA71V-CP2475L (P220) | BA71V-CP2475L (p220) | Core shell | 1582 AFANGNFSQAVMELG |
| BA71V-CP2475L (P220) | BA71V-CP2475L (p220) | Core shell | 1583 FANGNFSQAVMELGY |
| BA71V-CP2475L (P220) | BA71V-CP2475L (p220) | Core shell | 1584 ANGNFSQAVMELGYA |
| BA71V-CP2475L (P220) | BA71V-CP2475L (p220) | Core shell | 1585 NGNFSQAVMELGYAH |
| BA71V-CP2475L (P220) | BA71V-CP2475L (p220) | Core shell | 1586 GNFSQAVMELGYAHP |
| BA71V-CP2475L (P220) | BA71V-CP2475L (p220) | Core shell | 1587 NFSQAVMELGYAHPD |
| BA71V-CP2475L (P220) | BA71V-CP2475L (p220) | Core shell | 1588 FSQAVMELGYAHPDL |
| BA71V-CP2475L (P220) | BA71V-CP2475L (p220) | Core shell | 1589 SQAVMELGYAHPDLA |
| BA71V-CP2475L (P220) | BA71V-CP2475L (p220) | Core shell | 1590 QAVMELGYAHPDLAR |
| BA71V-CP2475L (P220) | BA71V-CP2475L (p220) | Core shell | 1591 AVMELGYAHPDLARD |
| BA71V-CP2475L (P220) | BA71V-CP2475L (p220) | Core shell | 1592 VMELGYAHPDLARDN |
| BA71V-CP2475L (P220) | BA71V-CP2475L (p220) | Core shell | 1593 MELGYAHPDLARDNT |
| BA71V-CP2475L (P220) | BA71V-CP2475L (p220) | Core shell | 1594 ELGYAHPDLARDNTA |
| BA71V-CP2475L (P220) | BA71V-CP2475L (p220) | Core shell | 1595 LGYAHPDLARDNTAF |
| BA71V-CP2475L (P220) | BA71V-CP2475L (p220) | Core shell | 1596 GYAHPDLARDNTAFG |
| BA71V-CP2475L (P220) | BA71V-CP2475L (p220) | Core shell | 1597 YAHPDLARDNTAFGH |
| BA71V-CP2475L (P220) | BA71V-CP2475L (p220) | Core shell | 1598 AHPDLARDNTAFGHR |
| BA71V-CP2475L (P220) | BA71V-CP2475L (p220) | Core shell | 1599 HPDLARDNTAFGHRG |
| BA71V-CP2475L (P220) | BA71V-CP2475L (p220) | Core shell | 1600 PDLARDNTAFGHRGD |
| BA71V-CP2475L (P220) | BA71V-CP2475L (p220) | Core shell | 1601 DLARDNTAFGHRGDP |
| BA71V-CP2475L (P220) | BA71V-CP2475L (p220) | Core shell | 1602 LARDNTAFGHRGDPT |
| BA71V-CP2475L (P220) | BA71V-CP2475L (p220) | Core shell | 1603 ARDNTAFGHRGDPTE |
| BA71V-CP2475L (P220) | BA71V-CP2475L (p220) | Core shell | 1604 RDNTAFGHRGDPTEQ |
| BA71V-CP2475L (P220) | BA71V-CP2475L (p220) | Core shell | 1605 DNTAFGHRGDPTEQS |
| BA71V-CP2475L (P220) | BA71V-CP2475L (p220) | Core shell | 1606 NTAFGHRGDPTEQSV |
| BA71V-CP2475L (P220) | BA71V-CP2475L (p220) | Core shell | 1607 TAFGHRGDPTEQSVL |
| BA71V-CP2475L (P220) | BA71V-CP2475L (p220) | Core shell | 1608 AFGHRGDPTEQSVLL |
| BA71V-CP2475L (P220) | BA71V-CP2475L (p220) | Core shell | 1609 FGHRGDPTEQSVLLL |
| BA71V-CP2475L (P220) | BA71V-CP2475L (p220) | Core shell | 1610 GHRGDPTEQSVLLLS |
| BA71V-CP2475L (P220) | BA71V-CP2475L (p220) | Core shell | 1611 HRGDPTEQSVLLLSL |
| BA71V-CP2475L (P220) | BA71V-CP2475L (p220) | Core shell | 1612 RGDPTEQSVLLLSLG |
| BA71V-CP2475L (P220) | BA71V-CP2475L (p220) | Core shell | 1613 GDPTEQSVLLLSLGL |
| BA71V-CP2475L (P220) | BA71V-CP2475L (p220) | Core shell | 1614 DPTEQSVLLLSLGLM |
| BA71V-CP2475L (P220) | BA71V-CP2475L (p220) | Core shell | 1615 PTEQSVLLLSLGLML |
| BA71V-CP2475L (P220) | BA71V-CP2475L (p220) | Core shell | 1616 TEQSVLLLSLGLMLQ |
| BA71V-CP2475L (P220) | BA71V-CP2475L (p220) | Core shell | 1617 EQSVLLLSLGLMLQR |
| BA71V-CP2475L (P220) | BA71V-CP2475L (p220) | Core shell | 1618 QSVLLLSLGLMLQRL |
| BA71V-CP2475L (P220) | BA71V-CP2475L (p220) | Core shell | 1619 SVLLLSLGLMLQRLI |
| BA71V-CP2475L (P220) | BA71V-CP2475L (p220) | Core shell | 1620 VLLLSLGLMLQRLIK |
| BA71V-CP2475L (P220) | BA71V-CP2475L (p220) | Core shell | 1621 LLLSLGLMLQRLIKD |
| BA71V-CP2475L (P220) | BA71V-CP2475L (p220) | Core shell | 1622 LLSLGLMLQRLIKDT |
| BA71V-CP2475L (P220) | BA71V-CP2475L (p220) | Core shell | 1623 LSLGLMLQRLIKDTN |
| BA71V-CP2475L (P220) | BA71V-CP2475L (p220) | Core shell | 1624 SLGLMLQRLIKDTNR |
| BA71V-CP2475L (P220) | BA71V-CP2475L (p220) | Core shell | 1625 LGLMLQRLIKDTNRQ |
| BA71V-CP2475L (P220) | BA71V-CP2475L (p220) | Core shell | 1626 GLMLQRLIKDTNRQG |

| | | | | |
|---|---|---|---|---|
| BA71V-CP2475L (P220) | BA71V-CP2475L (p220) | Core shell | 1627 | LMLQRLIKDTNRQGL |
| BA71V-CP2475L (P220) | BA71V-CP2475L (p220) | Core shell | 1628 | MLQRLIKDTNRQGLS |
| BA71V-CP2475L (P220) | BA71V-CP2475L (p220) | Core shell | 1629 | LQRLIKDTNRQGLSQ |
| BA71V-CP2475L (P220) | BA71V-CP2475L (p220) | Core shell | 1630 | QRLIKDTNRQGLSQH |
| BA71V-CP2475L (P220) | BA71V-CP2475L (p220) | Core shell | 1631 | RLIKDTNRQGLSQHL |
| BA71V-CP2475L (P220) | BA71V-CP2475L (p220) | Core shell | 1632 | LIKDTNRQGLSQHLI |
| BA71V-CP2475L (P220) | BA71V-CP2475L (p220) | Core shell | 1633 | IKDTNRQGLSQHLIS |
| BA71V-CP2475L (P220) | BA71V-CP2475L (p220) | Core shell | 1634 | KDTNRQGLSQHLIST |
| BA71V-CP2475L (P220) | BA71V-CP2475L (p220) | Core shell | 1635 | DTNRQGLSQHLISTL |
| BA71V-CP2475L (P220) | BA71V-CP2475L (p220) | Core shell | 1636 | TNRQGLSQHLISTLT |
| BA71V-CP2475L (P220) | BA71V-CP2475L (p220) | Core shell | 1637 | NRQGLSQHLISTLTE |
| BA71V-CP2475L (P220) | BA71V-CP2475L (p220) | Core shell | 1638 | RQGLSQHLISTLTEI |
| BA71V-CP2475L (P220) | BA71V-CP2475L (p220) | Core shell | 1639 | QGLSQHLISTLTEIP |
| BA71V-CP2475L (P220) | BA71V-CP2475L (p220) | Core shell | 1640 | GLSQHLISTLTEIPI |
| BA71V-CP2475L (P220) | BA71V-CP2475L (p220) | Core shell | 1641 | LSQHLISTLTEIPIY |
| BA71V-CP2475L (P220) | BA71V-CP2475L (p220) | Core shell | 1642 | SQHLISTLTEIPIYL |
| BA71V-CP2475L (P220) | BA71V-CP2475L (p220) | Core shell | 1643 | QHLISTLTEIPIYLK |
| BA71V-CP2475L (P220) | BA71V-CP2475L (p220) | Core shell | 1644 | HLISTLTEIPIYLKE |
| BA71V-CP2475L (P220) | BA71V-CP2475L (p220) | Core shell | 1645 | LISTLTEIPIYLKEN |
| BA71V-CP2475L (P220) | BA71V-CP2475L (p220) | Core shell | 1646 | ISTLTEIPIYLKENY |
| BA71V-CP2475L (P220) | BA71V-CP2475L (p220) | Core shell | 1647 | STLTEIPIYLKENYR |
| BA71V-CP2475L (P220) | BA71V-CP2475L (p220) | Core shell | 1648 | TLTEIPIYLKENYRA |
| BA71V-CP2475L (P220) | BA71V-CP2475L (p220) | Core shell | 1649 | LTEIPIYLKENYRAN |
| BA71V-CP2475L (P220) | BA71V-CP2475L (p220) | Core shell | 1650 | TEIPIYLKENYRANL |
| BA71V-CP2475L (P220) | BA71V-CP2475L (p220) | Core shell | 1651 | EIPIYLKENYRANLP |
| BA71V-CP2475L (P220) | BA71V-CP2475L (p220) | Core shell | 1652 | IPIYLKENYRANLPL |
| BA71V-CP2475L (P220) | BA71V-CP2475L (p220) | Core shell | 1653 | PIYLKENYRANLPLF |
| BA71V-CP2475L (P220) | BA71V-CP2475L (p220) | Core shell | 1654 | IYLKENYRANLPLFN |
| BA71V-CP2475L (P220) | BA71V-CP2475L (p220) | Core shell | 1655 | YLKENYRANLPLFNK |
| BA71V-CP2475L (P220) | BA71V-CP2475L (p220) | Core shell | 1656 | LKENYRANLPLFNKM |
| BA71V-CP2475L (P220) | BA71V-CP2475L (p220) | Core shell | 1657 | KENYRANLPLFNKMF |
| BA71V-CP2475L (P220) | BA71V-CP2475L (p220) | Core shell | 1658 | ENYRANLPLFNKMFN |
| BA71V-CP2475L (P220) | BA71V-CP2475L (p220) | Core shell | 1659 | NYRANLPLFNKMFNI |
| BA71V-CP2475L (P220) | BA71V-CP2475L (p220) | Core shell | 1660 | YRANLPLFNKMFNIL |
| BA71V-CP2475L (P220) | BA71V-CP2475L (p220) | Core shell | 1661 | RANLPLFNKMFNILI |
| BA71V-CP2475L (P220) | BA71V-CP2475L (p220) | Core shell | 1662 | ANLPLFNKMFNILIS |
| BA71V-CP2475L (P220) | BA71V-CP2475L (p220) | Core shell | 1663 | NLPLFNKMFNILISQ |
| BA71V-CP2475L (P220) | BA71V-CP2475L (p220) | Core shell | 1664 | LPLFNKMFNILISQG |
| BA71V-CP2475L (P220) | BA71V-CP2475L (p220) | Core shell | 1665 | PLFNKMFNILISQGE |
| BA71V-CP2475L (P220) | BA71V-CP2475L (p220) | Core shell | 1666 | LFNKMFNILISQGEL |
| BA71V-CP2475L (P220) | BA71V-CP2475L (p220) | Core shell | 1667 | FNKMFNILISQGELL |
| BA71V-CP2475L (P220) | BA71V-CP2475L (p220) | Core shell | 1668 | NKMFNILISQGELLK |
| BA71V-CP2475L (P220) | BA71V-CP2475L (p220) | Core shell | 1669 | KMFNILISQGELLKQ |
| BA71V-CP2475L (P220) | BA71V-CP2475L (p220) | Core shell | 1670 | MFNILISQGELLKQF |
| BA71V-CP2475L (P220) | BA71V-CP2475L (p220) | Core shell | 1671 | FNILISQGELLKQFI |
| BA71V-CP2475L (P220) | BA71V-CP2475L (p220) | Core shell | 1672 | NILISQGELLKQFIQ |
| BA71V-CP2475L (P220) | BA71V-CP2475L (p220) | Core shell | 1673 | ILISQGELLKQFIQY |
| BA71V-CP2475L (P220) | BA71V-CP2475L (p220) | Core shell | 1674 | LISQGELLKQFIQYT |
| BA71V-CP2475L (P220) | BA71V-CP2475L (p220) | Core shell | 1675 | ISQGELLKQFIQYTK |
| BA71V-CP2475L (P220) | BA71V-CP2475L (p220) | Core shell | 1676 | SQGELLKQFIQYTKV |
| BA71V-CP2475L (P220) | BA71V-CP2475L (p220) | Core shell | 1677 | QGELLKQFIQYTKVQ |
| BA71V-CP2475L (P220) | BA71V-CP2475L (p220) | Core shell | 1678 | GELLKQFIQYTKVQL |
| BA71V-CP2475L (P220) | BA71V-CP2475L (p220) | Core shell | 1679 | ELLKQFIQYTKVQLA |
| BA71V-CP2475L (P220) | BA71V-CP2475L (p220) | Core shell | 1680 | LLKQFIQYTKVQLAR |
| BA71V-CP2475L (P220) | BA71V-CP2475L (p220) | Core shell | 1681 | LKQFIQYTKVQLARP |
| BA71V-CP2475L (P220) | BA71V-CP2475L (p220) | Core shell | 1682 | KQFIQYTKVQLARPN |
| BA71V-CP2475L (P220) | BA71V-CP2475L (p220) | Core shell | 1683 | QFIQYTKVQLARPNL |
| BA71V-CP2475L (P220) | BA71V-CP2475L (p220) | Core shell | 1684 | FIQYTKVQLARPNLT |
| BA71V-CP2475L (P220) | BA71V-CP2475L (p220) | Core shell | 1685 | IQYTKVQLARPNLTA |
| BA71V-CP2475L (P220) | BA71V-CP2475L (p220) | Core shell | 1686 | QYTKVQLARPNLTAL |
| BA71V-CP2475L (P220) | BA71V-CP2475L (p220) | Core shell | 1687 | YTKVQLARPNLTALL |
| BA71V-CP2475L (P220) | BA71V-CP2475L (p220) | Core shell | 1688 | TKVQLARPNLTALLG |
| BA71V-CP2475L (P220) | BA71V-CP2475L (p220) | Core shell | 1689 | KVQLARPNLTALLGA |
| BA71V-CP2475L (P220) | BA71V-CP2475L (p220) | Core shell | 1690 | VQLARPNLTALLGAN |
| BA71V-CP2475L (P220) | BA71V-CP2475L (p220) | Core shell | 1691 | QLARPNLTALLGANN |
| BA71V-CP2475L (P220) | BA71V-CP2475L (p220) | Core shell | 1692 | LARPNLTALLGANND |
| BA71V-CP2475L (P220) | BA71V-CP2475L (p220) | Core shell | 1693 | ARPNLTALLGANNDS |

| | | | | |
|---|---|---|---|---|
| BA71V-CP2475L (P220) | BA71V-CP2475L (p220) | Core shell | 1694 | RPNLTALLGANNDSI |
| BA71V-CP2475L (P220) | BA71V-CP2475L (p220) | Core shell | 1695 | PNLTALLGANNDSII |
| BA71V-CP2475L (P220) | BA71V-CP2475L (p220) | Core shell | 1696 | NLTALLGANNDSIIY |
| BA71V-CP2475L (P220) | BA71V-CP2475L (p220) | Core shell | 1697 | LTALLGANNDSIIYY |
| BA71V-CP2475L (P220) | BA71V-CP2475L (p220) | Core shell | 1698 | TALLGANNDSIIYYN |
| BA71V-CP2475L (P220) | BA71V-CP2475L (p220) | Core shell | 1699 | ALLGANNDSIIYYNN |
| BA71V-CP2475L (P220) | BA71V-CP2475L (p220) | Core shell | 1700 | LLGANNDSIIYYNNN |
| BA71V-CP2475L (P220) | BA71V-CP2475L (p220) | Core shell | 1701 | LGANNDSIIYYNNNN |
| BA71V-CP2475L (P220) | BA71V-CP2475L (p220) | Core shell | 1702 | GANNDSIIYYNNNNV |
| BA71V-CP2475L (P220) | BA71V-CP2475L (p220) | Core shell | 1703 | ANNDSIIYYNNNNVP |
| BA71V-CP2475L (P220) | BA71V-CP2475L (p220) | Core shell | 1704 | NNDSIIYYNNNNVPN |
| BA71V-CP2475L (P220) | BA71V-CP2475L (p220) | Core shell | 1705 | NDSIIYYNNNNVPNT |
| BA71V-CP2475L (P220) | BA71V-CP2475L (p220) | Core shell | 1706 | DSIIYYNNNNVPNTG |
| BA71V-CP2475L (P220) | BA71V-CP2475L (p220) | Core shell | 1707 | SIIYYNNNNVPNTGL |
| BA71V-CP2475L (P220) | BA71V-CP2475L (p220) | Core shell | 1708 | IIYYNNNNVPNTGLT |
| BA71V-CP2475L (P220) | BA71V-CP2475L (p220) | Core shell | 1709 | IYYNNNNVPNTGLTV |
| BA71V-CP2475L (P220) | BA71V-CP2475L (p220) | Core shell | 1710 | YYNNNNVPNTGLTVG |
| BA71V-CP2475L (P220) | BA71V-CP2475L (p220) | Core shell | 1711 | YNNNNVPNTGLTVGQ |
| BA71V-CP2475L (P220) | BA71V-CP2475L (p220) | Core shell | 1712 | NNNNVPNTGLTVGQA |
| BA71V-CP2475L (P220) | BA71V-CP2475L (p220) | Core shell | 1713 | NNNVPNTGLTVGQAA |
| BA71V-CP2475L (P220) | BA71V-CP2475L (p220) | Core shell | 1714 | NNVPNTGLTVGQAAL |
| BA71V-CP2475L (P220) | BA71V-CP2475L (p220) | Core shell | 1715 | NVPNTGLTVGQAALR |
| BA71V-CP2475L (P220) | BA71V-CP2475L (p220) | Core shell | 1716 | VPNTGLTVGQAALRG |
| BA71V-CP2475L (P220) | BA71V-CP2475L (p220) | Core shell | 1717 | PNTGLTVGQAALRGI |
| BA71V-CP2475L (P220) | BA71V-CP2475L (p220) | Core shell | 1718 | NTGLTVGQAALRGIG |
| BA71V-CP2475L (P220) | BA71V-CP2475L (p220) | Core shell | 1719 | TGLTVGQAALRGIGS |
| BA71V-CP2475L (P220) | BA71V-CP2475L (p220) | Core shell | 1720 | GLTVGQAALRGIGSV |
| BA71V-CP2475L (P220) | BA71V-CP2475L (p220) | Core shell | 1721 | LTVGQAALRGIGSVF |
| BA71V-CP2475L (P220) | BA71V-CP2475L (p220) | Core shell | 1722 | TVGQAALRGIGSVFR |
| BA71V-CP2475L (P220) | BA71V-CP2475L (p220) | Core shell | 1723 | VGQAALRGIGSVFRP |
| BA71V-CP2475L (P220) | BA71V-CP2475L (p220) | Core shell | 1724 | GQAALRGIGSVFRPD |
| BA71V-CP2475L (P220) | BA71V-CP2475L (p220) | Core shell | 1725 | QAALRGIGSVFRPDI |
| BA71V-CP2475L (P220) | BA71V-CP2475L (p220) | Core shell | 1726 | AALRGIGSVFRPDIT |
| BA71V-CP2475L (P220) | BA71V-CP2475L (p220) | Core shell | 1727 | ALRGIGSVFRPDITL |
| BA71V-CP2475L (P220) | BA71V-CP2475L (p220) | Core shell | 1728 | LRGIGSVFRPDITLM |
| BA71V-CP2475L (P220) | BA71V-CP2475L (p220) | Core shell | 1729 | RGIGSVFRPDITLMP |
| BA71V-CP2475L (P220) | BA71V-CP2475L (p220) | Core shell | 1730 | GIGSVFRPDITLMPL |
| BA71V-CP2475L (P220) | BA71V-CP2475L (p220) | Core shell | 1731 | IGSVFRPDITLMPLG |
| BA71V-CP2475L (P220) | BA71V-CP2475L (p220) | Core shell | 1732 | GSVFRPDITLMPLGN |
| BA71V-CP2475L (P220) | BA71V-CP2475L (p220) | Core shell | 1733 | SVFRPDITLMPLGNA |
| BA71V-CP2475L (P220) | BA71V-CP2475L (p220) | Core shell | 1734 | VFRPDITLMPLGNAQ |
| BA71V-CP2475L (P220) | BA71V-CP2475L (p220) | Core shell | 1735 | FRPDITLMPLGNAQN |
| BA71V-CP2475L (P220) | BA71V-CP2475L (p220) | Core shell | 1736 | RPDITLMPLGNAQNN |
| BA71V-CP2475L (P220) | BA71V-CP2475L (p220) | Core shell | 1737 | PDITLMPLGNAQNNT |
| BA71V-CP2475L (P220) | BA71V-CP2475L (p220) | Core shell | 1738 | DITLMPLGNAQNNTN |
| BA71V-CP2475L (P220) | BA71V-CP2475L (p220) | Core shell | 1739 | ITLMPLGNAQNNTND |
| BA71V-CP2475L (P220) | BA71V-CP2475L (p220) | Core shell | 1740 | TLMPLGNAQNNTNDV |
| BA71V-CP2475L (P220) | BA71V-CP2475L (p220) | Core shell | 1741 | LMPLGNAQNNTNDVV |
| BA71V-CP2475L (P220) | BA71V-CP2475L (p220) | Core shell | 1742 | MPLGNAQNNTNDVVR |
| BA71V-CP2475L (P220) | BA71V-CP2475L (p220) | Core shell | 1743 | PLGNAQNNTNDVVRK |
| BA71V-CP2475L (P220) | BA71V-CP2475L (p220) | Core shell | 1744 | LGNAQNNTNDVVRKR |
| BA71V-CP2475L (P220) | BA71V-CP2475L (p220) | Core shell | 1745 | GNAQNNTNDVVRKRL |
| BA71V-CP2475L (P220) | BA71V-CP2475L (p220) | Core shell | 1746 | NAQNNTNDVVRKRLI |
| BA71V-CP2475L (P220) | BA71V-CP2475L (p220) | Core shell | 1747 | AQNNTNDVVRKRLIA |
| BA71V-CP2475L (P220) | BA71V-CP2475L (p220) | Core shell | 1748 | QNNTNDVVRKRLIAV |
| BA71V-CP2475L (P220) | BA71V-CP2475L (p220) | Core shell | 1749 | NNTNDVVRKRLIAVI |
| BA71V-CP2475L (P220) | BA71V-CP2475L (p220) | Core shell | 1750 | NTNDVVRKRLIAVIN |
| BA71V-CP2475L (P220) | BA71V-CP2475L (p220) | Core shell | 1751 | TNDVVRKRLIAVING |
| BA71V-CP2475L (P220) | BA71V-CP2475L (p220) | Core shell | 1752 | NDVVRKRLIAVINGI |
| BA71V-CP2475L (P220) | BA71V-CP2475L (p220) | Core shell | 1753 | DVVRKRLIAVINGII |
| BA71V-CP2475L (P220) | BA71V-CP2475L (p220) | Core shell | 1754 | VVRKRLIAVINGIIR |
| BA71V-CP2475L (P220) | BA71V-CP2475L (p220) | Core shell | 1755 | VRKRLIAVINGIIRG |
| BA71V-CP2475L (P220) | BA71V-CP2475L (p220) | Core shell | 1756 | RKRLIAVINGIIRGS |
| BA71V-CP2475L (P220) | BA71V-CP2475L (p220) | Core shell | 1757 | KRLIAVINGIIRGSL |
| BA71V-CP2475L (P220) | BA71V-CP2475L (p220) | Core shell | 1758 | RLIAVINGIIRGSLT |
| BA71V-CP2475L (P220) | BA71V-CP2475L (p220) | Core shell | 1759 | LIAVINGIIRGSLTL |
| BA71V-CP2475L (P220) | BA71V-CP2475L (p220) | Core shell | 1760 | IAVINGIIRGSLTLA |

| | | | | |
|---|---|---|---|---|
| BA71V-CP2475L (P220) | BA71V-CP2475L (p220) | Core shell | 1761 | AVINGIIRGSLTLAN |
| BA71V-CP2475L (P220) | BA71V-CP2475L (p220) | Core shell | 1762 | VINGIIRGSLTLANS |
| BA71V-CP2475L (P220) | BA71V-CP2475L (p220) | Core shell | 1763 | INGIIRGSLTLANSA |
| BA71V-CP2475L (P220) | BA71V-CP2475L (p220) | Core shell | 1764 | NGIIRGSLTLANSAM |
| BA71V-CP2475L (P220) | BA71V-CP2475L (p220) | Core shell | 1765 | GIIRGSLTLANSAME |
| BA71V-CP2475L (P220) | BA71V-CP2475L (p220) | Core shell | 1766 | IIRGSLTLANSAMEV |
| BA71V-CP2475L (P220) | BA71V-CP2475L (p220) | Core shell | 1767 | IRGSLTLANSAMEVL |
| BA71V-CP2475L (P220) | BA71V-CP2475L (p220) | Core shell | 1768 | RGSLTLANSAMEVLH |
| BA71V-CP2475L (P220) | BA71V-CP2475L (p220) | Core shell | 1769 | GSLTLANSAMEVLHE |
| BA71V-CP2475L (P220) | BA71V-CP2475L (p220) | Core shell | 1770 | SLTLANSAMEVLHEL |
| BA71V-CP2475L (P220) | BA71V-CP2475L (p220) | Core shell | 1771 | LTLANSAMEVLHELT |
| BA71V-CP2475L (P220) | BA71V-CP2475L (p220) | Core shell | 1772 | TLANSAMEVLHELTD |
| BA71V-CP2475L (P220) | BA71V-CP2475L (p220) | Core shell | 1773 | LANSAMEVLHELTDH |
| BA71V-CP2475L (P220) | BA71V-CP2475L (p220) | Core shell | 1774 | ANSAMEVLHELTDHP |
| BA71V-CP2475L (P220) | BA71V-CP2475L (p220) | Core shell | 1775 | NSAMEVLHELTDHPI |
| BA71V-CP2475L (P220) | BA71V-CP2475L (p220) | Core shell | 1776 | SAMEVLHELTDHPIY |
| BA71V-CP2475L (P220) | BA71V-CP2475L (p220) | Core shell | 1777 | AMEVLHELTDHPIYF |
| BA71V-CP2475L (P220) | BA71V-CP2475L (p220) | Core shell | 1778 | MEVLHELTDHPIYFE |
| BA71V-CP2475L (P220) | BA71V-CP2475L (p220) | Core shell | 1779 | EVLHELTDHPIYFET |
| BA71V-CP2475L (P220) | BA71V-CP2475L (p220) | Core shell | 1780 | VLHELTDHPIYFETE |
| BA71V-CP2475L (P220) | BA71V-CP2475L (p220) | Core shell | 1781 | LHELTDHPIYFETEE |
| BA71V-CP2475L (P220) | BA71V-CP2475L (p220) | Core shell | 1782 | HELTDHPIYFETEEH |
| BA71V-CP2475L (P220) | BA71V-CP2475L (p220) | Core shell | 1783 | ELTDHPIYFETEEHF |
| BA71V-CP2475L (P220) | BA71V-CP2475L (p220) | Core shell | 1784 | LTDHPIYFETEEHFI |
| BA71V-CP2475L (P220) | BA71V-CP2475L (p220) | Core shell | 1785 | TDHPIYFETEEHFIQ |
| BA71V-CP2475L (P220) | BA71V-CP2475L (p220) | Core shell | 1786 | DHPIYFETEEHFIQN |
| BA71V-CP2475L (P220) | BA71V-CP2475L (p220) | Core shell | 1787 | HPIYFETEEHFIQNY |
| BA71V-CP2475L (P220) | BA71V-CP2475L (p220) | Core shell | 1788 | PIYFETEEHFIQNYM |
| BA71V-CP2475L (P220) | BA71V-CP2475L (p220) | Core shell | 1789 | IYFETEEHFIQNYMS |
| BA71V-CP2475L (P220) | BA71V-CP2475L (p220) | Core shell | 1790 | YFETEEHFIQNYMSR |
| BA71V-CP2475L (P220) | BA71V-CP2475L (p220) | Core shell | 1791 | FETEEHFIQNYMSRY |
| BA71V-CP2475L (P220) | BA71V-CP2475L (p220) | Core shell | 1792 | ETEEHFIQNYMSRYN |
| BA71V-CP2475L (P220) | BA71V-CP2475L (p220) | Core shell | 1793 | TEEHFIQNYMSRYNK |
| BA71V-CP2475L (P220) | BA71V-CP2475L (p220) | Core shell | 1794 | EEHFIQNYMSRYNKE |
| BA71V-CP2475L (P220) | BA71V-CP2475L (p220) | Core shell | 1795 | EHFIQNYMSRYNKEP |
| BA71V-CP2475L (P220) | BA71V-CP2475L (p220) | Core shell | 1796 | HFIQNYMSRYNKEPL |
| BA71V-CP2475L (P220) | BA71V-CP2475L (p220) | Core shell | 1797 | FIQNYMSRYNKEPLM |
| BA71V-CP2475L (P220) | BA71V-CP2475L (p220) | Core shell | 1798 | IQNYMSRYNKEPLMP |
| BA71V-CP2475L (P220) | BA71V-CP2475L (p220) | Core shell | 1799 | QNYMSRYNKEPLMPF |
| BA71V-CP2475L (P220) | BA71V-CP2475L (p220) | Core shell | 1800 | NYMSRYNKEPLMPFS |
| BA71V-CP2475L (P220) | BA71V-CP2475L (p220) | Core shell | 1801 | YMSRYNKEPLMPFSL |
| BA71V-CP2475L (P220) | BA71V-CP2475L (p220) | Core shell | 1802 | MSRYNKEPLMPFSLS |
| BA71V-CP2475L (P220) | BA71V-CP2475L (p220) | Core shell | 1803 | SRYNKEPLMPFSLSL |
| BA71V-CP2475L (P220) | BA71V-CP2475L (p220) | Core shell | 1804 | RYNKEPLMPFSLSLY |
| BA71V-CP2475L (P220) | BA71V-CP2475L (p220) | Core shell | 1805 | YNKEPLMPFSLSLYY |
| BA71V-CP2475L (P220) | BA71V-CP2475L (p220) | Core shell | 1806 | NKEPLMPFSLSLYYL |
| BA71V-CP2475L (P220) | BA71V-CP2475L (p220) | Core shell | 1807 | KEPLMPFSLSLYYLR |
| BA71V-CP2475L (P220) | BA71V-CP2475L (p220) | Core shell | 1808 | EPLMPFSLSLYYLRD |
| BA71V-CP2475L (P220) | BA71V-CP2475L (p220) | Core shell | 1809 | PLMPFSLSLYYLRDL |
| BA71V-CP2475L (P220) | BA71V-CP2475L (p220) | Core shell | 1810 | LMPFSLSLYYLRDLR |
| BA71V-CP2475L (P220) | BA71V-CP2475L (p220) | Core shell | 1811 | MPFSLSLYYLRDLRI |
| BA71V-CP2475L (P220) | BA71V-CP2475L (p220) | Core shell | 1812 | PFSLSLYYLRDLRIE |
| BA71V-CP2475L (P220) | BA71V-CP2475L (p220) | Core shell | 1813 | FSLSLYYLRDLRIEN |
| BA71V-CP2475L (P220) | BA71V-CP2475L (p220) | Core shell | 1814 | SLSLYYLRDLRIENN |
| BA71V-CP2475L (P220) | BA71V-CP2475L (p220) | Core shell | 1815 | LSLYYLRDLRIENNE |
| BA71V-CP2475L (P220) | BA71V-CP2475L (p220) | Core shell | 1816 | SLYYLRDLRIENNEV |
| BA71V-CP2475L (P220) | BA71V-CP2475L (p220) | Core shell | 1817 | LYYLRDLRIENNEVY |
| BA71V-CP2475L (P220) | BA71V-CP2475L (p220) | Core shell | 1818 | YYLRDLRIENNEVYD |
| BA71V-CP2475L (P220) | BA71V-CP2475L (p220) | Core shell | 1819 | YLRDLRIENNEVYDP |
| BA71V-CP2475L (P220) | BA71V-CP2475L (p220) | Core shell | 1820 | LRDLRIENNEVYDPL |
| BA71V-CP2475L (P220) | BA71V-CP2475L (p220) | Core shell | 1821 | RDLRIENNEVYDPLL |
| BA71V-CP2475L (P220) | BA71V-CP2475L (p220) | Core shell | 1822 | DLRIENNEVYDPLLY |
| BA71V-CP2475L (P220) | BA71V-CP2475L (p220) | Core shell | 1823 | LRIENNEVYDPLLYP |
| BA71V-CP2475L (P220) | BA71V-CP2475L (p220) | Core shell | 1824 | RIENNEVYDPLLYPN |
| BA71V-CP2475L (P220) | BA71V-CP2475L (p220) | Core shell | 1825 | IENNEVYDPLLYPNL |
| BA71V-CP2475L (P220) | BA71V-CP2475L (p220) | Core shell | 1826 | ENNEVYDPLLYPNLE |
| BA71V-CP2475L (P220) | BA71V-CP2475L (p220) | Core shell | 1827 | NNEVYDPLLYPNLES |

| | | | | |
|---|---|---|---|---|
| BA71V-CP2475L (P220) | BA71V-CP2475L (p220) | Core shell | 1828 | NEVYDPLLYPNLESG |
| BA71V-CP2475L (P220) | BA71V-CP2475L (p220) | Core shell | 1829 | EVYDPLLYPNLESGS |
| BA71V-CP2475L (P220) | BA71V-CP2475L (p220) | Core shell | 1830 | VYDPLLYPNLESGSP |
| BA71V-CP2475L (P220) | BA71V-CP2475L (p220) | Core shell | 1831 | YDPLLYPNLESGSPE |
| BA71V-CP2475L (P220) | BA71V-CP2475L (p220) | Core shell | 1832 | DPLLYPNLESGSPEF |
| BA71V-CP2475L (P220) | BA71V-CP2475L (p220) | Core shell | 1833 | PLLYPNLESGSPEFK |
| BA71V-CP2475L (P220) | BA71V-CP2475L (p220) | Core shell | 1834 | LLYPNLESGSPEFKI |
| BA71V-CP2475L (P220) | BA71V-CP2475L (p220) | Core shell | 1835 | LYPNLESGSPEFKIL |
| BA71V-CP2475L (P220) | BA71V-CP2475L (p220) | Core shell | 1836 | YPNLESGSPEFKILY |
| BA71V-CP2475L (P220) | BA71V-CP2475L (p220) | Core shell | 1837 | PNLESGSPEFKILYG |
| BA71V-CP2475L (P220) | BA71V-CP2475L (p220) | Core shell | 1838 | NLESGSPEFKILYGT |
| BA71V-CP2475L (P220) | BA71V-CP2475L (p220) | Core shell | 1839 | LESGSPEFKILYGTR |
| BA71V-CP2475L (P220) | BA71V-CP2475L (p220) | Core shell | 1840 | ESGSPEFKILYGTRK |
| BA71V-CP2475L (P220) | BA71V-CP2475L (p220) | Core shell | 1841 | SGSPEFKILYGTRKL |
| BA71V-CP2475L (P220) | BA71V-CP2475L (p220) | Core shell | 1842 | GSPEFKILYGTRKLL |
| BA71V-CP2475L (P220) | BA71V-CP2475L (p220) | Core shell | 1843 | SPEFKILYGTRKLLG |
| BA71V-CP2475L (P220) | BA71V-CP2475L (p220) | Core shell | 1844 | PEFKILYGTRKLLGN |
| BA71V-CP2475L (P220) | BA71V-CP2475L (p220) | Core shell | 1845 | EFKILYGTRKLLGND |
| BA71V-CP2475L (P220) | BA71V-CP2475L (p220) | Core shell | 1846 | FKILYGTRKLLGNDP |
| BA71V-CP2475L (P220) | BA71V-CP2475L (p220) | Core shell | 1847 | KILYGTRKLLGNDPV |
| BA71V-CP2475L (P220) | BA71V-CP2475L (p220) | Core shell | 1848 | ILYGTRKLLGNDPVQ |
| BA71V-CP2475L (P220) | BA71V-CP2475L (p220) | Core shell | 1849 | LYGTRKLLGNDPVQL |
| BA71V-CP2475L (P220) | BA71V-CP2475L (p220) | Core shell | 1850 | YGTRKLLGNDPVQLS |
| BA71V-CP2475L (P220) | BA71V-CP2475L (p220) | Core shell | 1851 | GTRKLLGNDPVQLSD |
| BA71V-CP2475L (P220) | BA71V-CP2475L (p220) | Core shell | 1852 | TRKLLGNDPVQLSDM |
| BA71V-CP2475L (P220) | BA71V-CP2475L (p220) | Core shell | 1853 | RKLLGNDPVQLSDMP |
| BA71V-CP2475L (P220) | BA71V-CP2475L (p220) | Core shell | 1854 | KLLGNDPVQLSDMPG |
| BA71V-CP2475L (P220) | BA71V-CP2475L (p220) | Core shell | 1855 | LLGNDPVQLSDMPGV |
| BA71V-CP2475L (P220) | BA71V-CP2475L (p220) | Core shell | 1856 | LGNDPVQLSDMPGVQ |
| BA71V-CP2475L (P220) | BA71V-CP2475L (p220) | Core shell | 1857 | GNDPVQLSDMPGVQL |
| BA71V-CP2475L (P220) | BA71V-CP2475L (p220) | Core shell | 1858 | NDPVQLSDMPGVQLI |
| BA71V-CP2475L (P220) | BA71V-CP2475L (p220) | Core shell | 1859 | DPVQLSDMPGVQLIM |
| BA71V-CP2475L (P220) | BA71V-CP2475L (p220) | Core shell | 1860 | PVQLSDMPGVQLIMK |
| BA71V-CP2475L (P220) | BA71V-CP2475L (p220) | Core shell | 1861 | VQLSDMPGVQLIMKN |
| BA71V-CP2475L (P220) | BA71V-CP2475L (p220) | Core shell | 1862 | QLSDMPGVQLIMKNY |
| BA71V-CP2475L (P220) | BA71V-CP2475L (p220) | Core shell | 1863 | LSDMPGVQLIMKNYN |
| BA71V-CP2475L (P220) | BA71V-CP2475L (p220) | Core shell | 1864 | SDMPGVQLIMKNYNE |
| BA71V-CP2475L (P220) | BA71V-CP2475L (p220) | Core shell | 1865 | DMPGVQLIMKNYNET |
| BA71V-CP2475L (P220) | BA71V-CP2475L (p220) | Core shell | 1866 | MPGVQLIMKNYNETV |
| BA71V-CP2475L (P220) | BA71V-CP2475L (p220) | Core shell | 1867 | PGVQLIMKNYNETVV |
| BA71V-CP2475L (P220) | BA71V-CP2475L (p220) | Core shell | 1868 | GVQLIMKNYNETVVA |
| BA71V-CP2475L (P220) | BA71V-CP2475L (p220) | Core shell | 1869 | VQLIMKNYNETVVAR |
| BA71V-CP2475L (P220) | BA71V-CP2475L (p220) | Core shell | 1870 | QLIMKNYNETVVARE |
| BA71V-CP2475L (P220) | BA71V-CP2475L (p220) | Core shell | 1871 | LIMKNYNETVVAREQ |
| BA71V-CP2475L (P220) | BA71V-CP2475L (p220) | Core shell | 1872 | IMKNYNETVVAREQI |
| BA71V-CP2475L (P220) | BA71V-CP2475L (p220) | Core shell | 1873 | MKNYNETVVAREQIT |
| BA71V-CP2475L (P220) | BA71V-CP2475L (p220) | Core shell | 1874 | KNYNETVVAREQITP |
| BA71V-CP2475L (P220) | BA71V-CP2475L (p220) | Core shell | 1875 | NYNETVVAREQITPT |
| BA71V-CP2475L (P220) | BA71V-CP2475L (p220) | Core shell | 1876 | YNETVVAREQITPTR |
| BA71V-CP2475L (P220) | BA71V-CP2475L (p220) | Core shell | 1877 | NETVVAREQITPTRF |
| BA71V-CP2475L (P220) | BA71V-CP2475L (p220) | Core shell | 1878 | ETVVAREQITPTRFE |
| BA71V-CP2475L (P220) | BA71V-CP2475L (p220) | Core shell | 1879 | TVVAREQITPTRFEH |
| BA71V-CP2475L (P220) | BA71V-CP2475L (p220) | Core shell | 1880 | VVAREQITPTRFEHF |
| BA71V-CP2475L (P220) | BA71V-CP2475L (p220) | Core shell | 1881 | VAREQITPTRFEHFY |
| BA71V-CP2475L (P220) | BA71V-CP2475L (p220) | Core shell | 1882 | AREQITPTRFEHFYI |
| BA71V-CP2475L (P220) | BA71V-CP2475L (p220) | Core shell | 1883 | REQITPTRFEHFYIH |
| BA71V-CP2475L (P220) | BA71V-CP2475L (p220) | Core shell | 1884 | EQITPTRFEHFYIHA |
| BA71V-CP2475L (P220) | BA71V-CP2475L (p220) | Core shell | 1885 | QITPTRFEHFYIHAI |
| BA71V-CP2475L (P220) | BA71V-CP2475L (p220) | Core shell | 1886 | ITPTRFEHFYIHAIQ |
| BA71V-CP2475L (P220) | BA71V-CP2475L (p220) | Core shell | 1887 | TPTRFEHFYIHAIQA |
| BA71V-CP2475L (P220) | BA71V-CP2475L (p220) | Core shell | 1888 | PTRFEHFYIHAIQAL |
| BA71V-CP2475L (P220) | BA71V-CP2475L (p220) | Core shell | 1889 | TRFEHFYIHAIQALR |
| BA71V-CP2475L (P220) | BA71V-CP2475L (p220) | Core shell | 1890 | RFEHFYIHAIQALRF |
| BA71V-CP2475L (P220) | BA71V-CP2475L (p220) | Core shell | 1891 | FEHFYIHAIQALRFI |
| BA71V-CP2475L (P220) | BA71V-CP2475L (p220) | Core shell | 1892 | EHFYIHAIQALRFII |
| BA71V-CP2475L (P220) | BA71V-CP2475L (p220) | Core shell | 1893 | HFYIHAIQALRFIIN |
| BA71V-CP2475L (P220) | BA71V-CP2475L (p220) | Core shell | 1894 | FYIHAIQALRFIINI |

| | | | | |
|---|---|---|---|---|
| BA71V-CP2475L (P220) | BA71V-CP2475L (p220) | Core shell | 1895 | YIHAIQALRFIINIR |
| BA71V-CP2475L (P220) | BA71V-CP2475L (p220) | Core shell | 1896 | IHAIQALRFIINIRS |
| BA71V-CP2475L (P220) | BA71V-CP2475L (p220) | Core shell | 1897 | HAIQALRFIINIRSF |
| BA71V-CP2475L (P220) | BA71V-CP2475L (p220) | Core shell | 1898 | AIQALRFIINIRSFK |
| BA71V-CP2475L (P220) | BA71V-CP2475L (p220) | Core shell | 1899 | IQALRFIINIRSFKT |
| BA71V-CP2475L (P220) | BA71V-CP2475L (p220) | Core shell | 1900 | QALRFIINIRSFKTV |
| BA71V-CP2475L (P220) | BA71V-CP2475L (p220) | Core shell | 1901 | ALRFIINIRSFKTVM |
| BA71V-CP2475L (P220) | BA71V-CP2475L (p220) | Core shell | 1902 | LRFIINIRSFKTVMT |
| BA71V-CP2475L (P220) | BA71V-CP2475L (p220) | Core shell | 1903 | RFIINIRSFKTVMTY |
| BA71V-CP2475L (P220) | BA71V-CP2475L (p220) | Core shell | 1904 | FIINIRSFKTVMTYN |
| BA71V-CP2475L (P220) | BA71V-CP2475L (p220) | Core shell | 1905 | IINIRSFKTVMTYNE |
| BA71V-CP2475L (P220) | BA71V-CP2475L (p220) | Core shell | 1906 | INIRSFKTVMTYNEN |
| BA71V-CP2475L (P220) | BA71V-CP2475L (p220) | Core shell | 1907 | NIRSFKTVMTYNENT |
| BA71V-CP2475L (P220) | BA71V-CP2475L (p220) | Core shell | 1908 | IRSFKTVMTYNENTF |
| BA71V-CP2475L (P220) | BA71V-CP2475L (p220) | Core shell | 1909 | RSFKTVMTYNENTFG |
| BA71V-CP2475L (P220) | BA71V-CP2475L (p220) | Core shell | 1910 | SFKTVMTYNENTFGG |
| BA71V-CP2475L (P220) | BA71V-CP2475L (p220) | Core shell | 1911 | FKTVMTYNENTFGGV |
| BA71V-CP2475L (P220) | BA71V-CP2475L (p220) | Core shell | 1912 | KTVMTYNENTFGGVN |
| BA71V-CP2475L (P220) | BA71V-CP2475L (p220) | Core shell | 1913 | TVMTYNENTFGGVNL |
| BA71V-CP2475L (P220) | BA71V-CP2475L (p220) | Core shell | 1914 | VMTYNENTFGGVNLI |
| BA71V-CP2475L (P220) | BA71V-CP2475L (p220) | Core shell | 1915 | MTYNENTFGGVNLIS |
| BA71V-CP2475L (P220) | BA71V-CP2475L (p220) | Core shell | 1916 | TYNENTFGGVNLISE |
| BA71V-CP2475L (P220) | BA71V-CP2475L (p220) | Core shell | 1917 | YNENTFGGVNLISED |
| BA71V-CP2475L (P220) | BA71V-CP2475L (p220) | Core shell | 1918 | NENTFGGVNLISEDR |
| BA71V-CP2475L (P220) | BA71V-CP2475L (p220) | Core shell | 1919 | ENTFGGVNLISEDRD |
| BA71V-CP2475L (P220) | BA71V-CP2475L (p220) | Core shell | 1920 | NTFGGVNLISEDRDD |
| BA71V-CP2475L (P220) | BA71V-CP2475L (p220) | Core shell | 1921 | TFGGVNLISEDRDDK |
| BA71V-CP2475L (P220) | BA71V-CP2475L (p220) | Core shell | 1922 | FGGVNLISEDRDDKP |
| BA71V-CP2475L (P220) | BA71V-CP2475L (p220) | Core shell | 1923 | GGVNLISEDRDDKPI |
| BA71V-CP2475L (P220) | BA71V-CP2475L (p220) | Core shell | 1924 | GVNLISEDRDDKPII |
| BA71V-CP2475L (P220) | BA71V-CP2475L (p220) | Core shell | 1925 | VNLISEDRDDKPIIT |
| BA71V-CP2475L (P220) | BA71V-CP2475L (p220) | Core shell | 1926 | NLISEDRDDKPIITE |
| BA71V-CP2475L (P220) | BA71V-CP2475L (p220) | Core shell | 1927 | LISEDRDDKPIITEG |
| BA71V-CP2475L (P220) | BA71V-CP2475L (p220) | Core shell | 1928 | ISEDRDDKPIITEGI |
| BA71V-CP2475L (P220) | BA71V-CP2475L (p220) | Core shell | 1929 | SEDRDDKPIITEGIG |
| BA71V-CP2475L (P220) | BA71V-CP2475L (p220) | Core shell | 1930 | EDRDDKPIITEGIGM |
| BA71V-CP2475L (P220) | BA71V-CP2475L (p220) | Core shell | 1931 | DRDDKPIITEGIGMN |
| BA71V-CP2475L (P220) | BA71V-CP2475L (p220) | Core shell | 1932 | RDDKPIITEGIGMNA |
| BA71V-CP2475L (P220) | BA71V-CP2475L (p220) | Core shell | 1933 | DDKPIITEGIGMNAV |
| BA71V-CP2475L (P220) | BA71V-CP2475L (p220) | Core shell | 1934 | DKPIITEGIGMNAVY |
| BA71V-CP2475L (P220) | BA71V-CP2475L (p220) | Core shell | 1935 | KPIITEGIGMNAVYS |
| BA71V-CP2475L (P220) | BA71V-CP2475L (p220) | Core shell | 1936 | PIITEGIGMNAVYSL |
| BA71V-CP2475L (P220) | BA71V-CP2475L (p220) | Core shell | 1937 | IITEGIGMNAVYSLR |
| BA71V-CP2475L (P220) | BA71V-CP2475L (p220) | Core shell | 1938 | ITEGIGMNAVYSLRK |
| BA71V-CP2475L (P220) | BA71V-CP2475L (p220) | Core shell | 1939 | TEGIGMNAVYSLRKT |
| BA71V-CP2475L (P220) | BA71V-CP2475L (p220) | Core shell | 1940 | EGIGMNAVYSLRKTL |
| BA71V-CP2475L (P220) | BA71V-CP2475L (p220) | Core shell | 1941 | GIGMNAVYSLRKTLQ |
| BA71V-CP2475L (P220) | BA71V-CP2475L (p220) | Core shell | 1942 | IGMNAVYSLRKTLQD |
| BA71V-CP2475L (P220) | BA71V-CP2475L (p220) | Core shell | 1943 | GMNAVYSLRKTLQDV |
| BA71V-CP2475L (P220) | BA71V-CP2475L (p220) | Core shell | 1944 | MNAVYSLRKTLQDVI |
| BA71V-CP2475L (P220) | BA71V-CP2475L (p220) | Core shell | 1945 | NAVYSLRKTLQDVIS |
| BA71V-CP2475L (P220) | BA71V-CP2475L (p220) | Core shell | 1946 | AVYSLRKTLQDVISF |
| BA71V-CP2475L (P220) | BA71V-CP2475L (p220) | Core shell | 1947 | VYSLRKTLQDVISFV |
| BA71V-CP2475L (P220) | BA71V-CP2475L (p220) | Core shell | 1948 | YSLRKTLQDVISFVE |
| BA71V-CP2475L (P220) | BA71V-CP2475L (p220) | Core shell | 1949 | SLRKTLQDVISFVES |
| BA71V-CP2475L (P220) | BA71V-CP2475L (p220) | Core shell | 1950 | LRKTLQDVISFVESS |
| BA71V-CP2475L (P220) | BA71V-CP2475L (p220) | Core shell | 1951 | RKTLQDVISFVESSY |
| BA71V-CP2475L (P220) | BA71V-CP2475L (p220) | Core shell | 1952 | KTLQDVISFVESSYQ |
| BA71V-CP2475L (P220) | BA71V-CP2475L (p220) | Core shell | 1953 | TLQDVISFVESSYQE |
| BA71V-CP2475L (P220) | BA71V-CP2475L (p220) | Core shell | 1954 | LQDVISFVESSYQEE |
| BA71V-CP2475L (P220) | BA71V-CP2475L (p220) | Core shell | 1955 | QDVISFVESSYQEEQ |
| BA71V-CP2475L (P220) | BA71V-CP2475L (p220) | Core shell | 1956 | DVISFVESSYQEEQI |
| BA71V-CP2475L (P220) | BA71V-CP2475L (p220) | Core shell | 1957 | VISFVESSYQEEQIN |
| BA71V-CP2475L (P220) | BA71V-CP2475L (p220) | Core shell | 1958 | ISFVESSYQEEQINN |
| BA71V-CP2475L (P220) | BA71V-CP2475L (p220) | Core shell | 1959 | SFVESSYQEEQINNI |
| BA71V-CP2475L (P220) | BA71V-CP2475L (p220) | Core shell | 1960 | FVESSYQEEQINNIH |
| BA71V-CP2475L (P220) | BA71V-CP2475L (p220) | Core shell | 1961 | VESSYQEEQINNIHK |

| | | | | |
|---|---|---|---|---|
| BA71V-CP2475L (P220) | BA71V-CP2475L (p220) | Core shell | 1962 | ESSYQEEQINNIHKI |
| BA71V-CP2475L (P220) | BA71V-CP2475L (p220) | Core shell | 1963 | SSYQEEQINNIHKIV |
| BA71V-CP2475L (P220) | BA71V-CP2475L (p220) | Core shell | 1964 | SYQEEQINNIHKIVS |
| BA71V-CP2475L (P220) | BA71V-CP2475L (p220) | Core shell | 1965 | YQEEQINNIHKIVSP |
| BA71V-CP2475L (P220) | BA71V-CP2475L (p220) | Core shell | 1966 | QEEQINNIHKIVSPR |
| BA71V-CP2475L (P220) | BA71V-CP2475L (p220) | Core shell | 1967 | EEQINNIHKIVSPRS |
| BA71V-CP2475L (P220) | BA71V-CP2475L (p220) | Core shell | 1968 | EQINNIHKIVSPRSQ |
| BA71V-CP2475L (P220) | BA71V-CP2475L (p220) | Core shell | 1969 | QINNIHKIVSPRSQT |
| BA71V-CP2475L (P220) | BA71V-CP2475L (p220) | Core shell | 1970 | INNIHKIVSPRSQTR |
| BA71V-CP2475L (P220) | BA71V-CP2475L (p220) | Core shell | 1971 | NNIHKIVSPRSQTRS |
| BA71V-CP2475L (P220) | BA71V-CP2475L (p220) | Core shell | 1972 | NIHKIVSPRSQTRSL |
| BA71V-CP2475L (P220) | BA71V-CP2475L (p220) | Core shell | 1973 | IHKIVSPRSQTRSLG |
| BA71V-CP2475L (P220) | BA71V-CP2475L (p220) | Core shell | 1974 | HKIVSPRSQTRSLGS |
| BA71V-CP2475L (P220) | BA71V-CP2475L (p220) | Core shell | 1975 | KIVSPRSQTRSLGSN |
| BA71V-CP2475L (P220) | BA71V-CP2475L (p220) | Core shell | 1976 | IVSPRSQTRSLGSNR |
| BA71V-CP2475L (P220) | BA71V-CP2475L (p220) | Core shell | 1977 | VSPRSQTRSLGSNRE |
| BA71V-CP2475L (P220) | BA71V-CP2475L (p220) | Core shell | 1978 | SPRSQTRSLGSNRER |
| BA71V-CP2475L (P220) | BA71V-CP2475L (p220) | Core shell | 1979 | PRSQTRSLGSNRERE |
| BA71V-CP2475L (P220) | BA71V-CP2475L (p220) | Core shell | 1980 | RSQTRSLGSNRERER |
| BA71V-CP2475L (P220) | BA71V-CP2475L (p220) | Core shell | 1981 | SQTRSLGSNRERERI |
| BA71V-CP2475L (P220) | BA71V-CP2475L (p220) | Core shell | 1982 | QTRSLGSNRERERIF |
| BA71V-CP2475L (P220) | BA71V-CP2475L (p220) | Core shell | 1983 | TRSLGSNRERERIFN |
| BA71V-CP2475L (P220) | BA71V-CP2475L (p220) | Core shell | 1984 | RSLGSNRERERIFNL |
| BA71V-CP2475L (P220) | BA71V-CP2475L (p220) | Core shell | 1985 | SLGSNRERERIFNLF |
| BA71V-CP2475L (P220) | BA71V-CP2475L (p220) | Core shell | 1986 | LGSNRERERIFNLFD |
| BA71V-CP2475L (P220) | BA71V-CP2475L (p220) | Core shell | 1987 | GSNRERERIFNLFDM |
| BA71V-CP2475L (P220) | BA71V-CP2475L (p220) | Core shell | 1988 | SNRERERIFNLFDMN |
| BA71V-CP2475L (P220) | BA71V-CP2475L (p220) | Core shell | 1989 | NRERERIFNLFDMNI |
| BA71V-CP2475L (P220) | BA71V-CP2475L (p220) | Core shell | 1990 | RERERIFNLFDMNIM |
| BA71V-CP2475L (P220) | BA71V-CP2475L (p220) | Core shell | 1991 | ERERIFNLFDMNIMP |
| BA71V-CP2475L (P220) | BA71V-CP2475L (p220) | Core shell | 1992 | RERIFNLFDMNIMPI |
| BA71V-CP2475L (P220) | BA71V-CP2475L (p220) | Core shell | 1993 | ERIFNLFDMNIMPIN |
| BA71V-CP2475L (P220) | BA71V-CP2475L (p220) | Core shell | 1994 | RIFNLFDMNIMPINV |
| BA71V-CP2475L (P220) | BA71V-CP2475L (p220) | Core shell | 1995 | IFNLFDMNIMPINVN |
| BA71V-CP2475L (P220) | BA71V-CP2475L (p220) | Core shell | 1996 | FNLFDMNIMPINVNA |
| BA71V-CP2475L (P220) | BA71V-CP2475L (p220) | Core shell | 1997 | NLFDMNIMPINVNAL |
| BA71V-CP2475L (P220) | BA71V-CP2475L (p220) | Core shell | 1998 | LFDMNIMPINVNALM |
| BA71V-CP2475L (P220) | BA71V-CP2475L (p220) | Core shell | 1999 | FDMNIMPINVNALMR |
| BA71V-CP2475L (P220) | BA71V-CP2475L (p220) | Core shell | 2000 | DMNIMPINVNALMRS |
| BA71V-CP2475L (P220) | BA71V-CP2475L (p220) | Core shell | 2001 | MNIMPINVNALMRSI |
| BA71V-CP2475L (P220) | BA71V-CP2475L (p220) | Core shell | 2002 | NIMPINVNALMRSIP |
| BA71V-CP2475L (P220) | BA71V-CP2475L (p220) | Core shell | 2003 | IMPINVNALMRSIPL |
| BA71V-CP2475L (P220) | BA71V-CP2475L (p220) | Core shell | 2004 | MPINVNALMRSIPLA |
| BA71V-CP2475L (P220) | BA71V-CP2475L (p220) | Core shell | 2005 | PINVNALMRSIPLAN |
| BA71V-CP2475L (P220) | BA71V-CP2475L (p220) | Core shell | 2006 | INVNALMRSIPLANI |
| BA71V-CP2475L (P220) | BA71V-CP2475L (p220) | Core shell | 2007 | NVNALMRSIPLANIY |
| BA71V-CP2475L (P220) | BA71V-CP2475L (p220) | Core shell | 2008 | VNALMRSIPLANIYN |
| BA71V-CP2475L (P220) | BA71V-CP2475L (p220) | Core shell | 2009 | NALMRSIPLANIYNY |
| BA71V-CP2475L (P220) | BA71V-CP2475L (p220) | Core shell | 2010 | ALMRSIPLANIYNYD |
| BA71V-CP2475L (P220) | BA71V-CP2475L (p220) | Core shell | 2011 | LMRSIPLANIYNYDY |
| BA71V-CP2475L (P220) | BA71V-CP2475L (p220) | Core shell | 2012 | MRSIPLANIYNYDYS |
| BA71V-CP2475L (P220) | BA71V-CP2475L (p220) | Core shell | 2013 | RSIPLANIYNYDYSF |
| BA71V-CP2475L (P220) | BA71V-CP2475L (p220) | Core shell | 2014 | SIPLANIYNYDYSFE |
| BA71V-CP2475L (P220) | BA71V-CP2475L (p220) | Core shell | 2015 | IPLANIYNYDYSFEE |
| BA71V-CP2475L (P220) | BA71V-CP2475L (p220) | Core shell | 2016 | PLANIYNYDYSFEEI |
| BA71V-CP2475L (P220) | BA71V-CP2475L (p220) | Core shell | 2017 | LANIYNYDYSFEEIA |
| BA71V-CP2475L (P220) | BA71V-CP2475L (p220) | Core shell | 2018 | ANIYNYDYSFEEIAC |
| BA71V-CP2475L (P220) | BA71V-CP2475L (p220) | Core shell | 2019 | NIYNYDYSFEEIACL |
| BA71V-CP2475L (P220) | BA71V-CP2475L (p220) | Core shell | 2020 | IYNYDYSFEEIACLM |
| BA71V-CP2475L (P220) | BA71V-CP2475L (p220) | Core shell | 2021 | YNYDYSFEEIACLMY |
| BA71V-CP2475L (P220) | BA71V-CP2475L (p220) | Core shell | 2022 | NYDYSFEEIACLMYG |
| BA71V-CP2475L (P220) | BA71V-CP2475L (p220) | Core shell | 2023 | YDYSFEEIACLMYGI |
| BA71V-CP2475L (P220) | BA71V-CP2475L (p220) | Core shell | 2024 | DYSFEEIACLMYGIS |
| BA71V-CP2475L (P220) | BA71V-CP2475L (p220) | Core shell | 2025 | YSFEEIACLMYGISA |
| BA71V-CP2475L (P220) | BA71V-CP2475L (p220) | Core shell | 2026 | SFEEIACLMYGISAE |
| BA71V-CP2475L (P220) | BA71V-CP2475L (p220) | Core shell | 2027 | FEEIACLMYGISAEK |
| BA71V-CP2475L (P220) | BA71V-CP2475L (p220) | Core shell | 2028 | EEIACLMYGISAEKV |

| | | | | |
|---|---|---|---|---|
| BA71V-CP2475L (P220) | BA71V-CP2475L (p220) | Core shell | 2029 | EIACLMYGISAEKVR |
| BA71V-CP2475L (P220) | BA71V-CP2475L (p220) | Core shell | 2030 | IACLMYGISAEKVRS |
| BA71V-CP2475L (P220) | BA71V-CP2475L (p220) | Core shell | 2031 | ACLMYGISAEKVRSL |
| BA71V-CP2475L (P220) | BA71V-CP2475L (p220) | Core shell | 2032 | CLMYGISAEKVRSLD |
| BA71V-CP2475L (P220) | BA71V-CP2475L (p220) | Core shell | 2033 | LMYGISAEKVRSLDT |
| BA71V-CP2475L (P220) | BA71V-CP2475L (p220) | Core shell | 2034 | MYGISAEKVRSLDTA |
| BA71V-CP2475L (P220) | BA71V-CP2475L (p220) | Core shell | 2035 | YGISAEKVRSLDTAA |
| BA71V-CP2475L (P220) | BA71V-CP2475L (p220) | Core shell | 2036 | GISAEKVRSLDTAAP |
| BA71V-CP2475L (P220) | BA71V-CP2475L (p220) | Core shell | 2037 | ISAEKVRSLDTAAPQ |
| BA71V-CP2475L (P220) | BA71V-CP2475L (p220) | Core shell | 2038 | SAEKVRSLDTAAPQP |
| BA71V-CP2475L (P220) | BA71V-CP2475L (p220) | Core shell | 2039 | AEKVRSLDTAAPQPD |
| BA71V-CP2475L (P220) | BA71V-CP2475L (p220) | Core shell | 2040 | EKVRSLDTAAPQPDV |
| BA71V-CP2475L (P220) | BA71V-CP2475L (p220) | Core shell | 2041 | KVRSLDTAAPQPDVA |
| BA71V-CP2475L (P220) | BA71V-CP2475L (p220) | Core shell | 2042 | VRSLDTAAPQPDVAQ |
| BA71V-CP2475L (P220) | BA71V-CP2475L (p220) | Core shell | 2043 | RSLDTAAPQPDVAQV |
| BA71V-CP2475L (P220) | BA71V-CP2475L (p220) | Core shell | 2044 | SLDTAAPQPDVAQVL |
| BA71V-CP2475L (P220) | BA71V-CP2475L (p220) | Core shell | 2045 | LDTAAPQPDVAQVLN |
| BA71V-CP2475L (P220) | BA71V-CP2475L (p220) | Core shell | 2046 | DTAAPQPDVAQVLNI |
| BA71V-CP2475L (P220) | BA71V-CP2475L (p220) | Core shell | 2047 | TAAPQPDVAQVLNIP |
| BA71V-CP2475L (P220) | BA71V-CP2475L (p220) | Core shell | 2048 | AAPQPDVAQVLNIPN |
| BA71V-CP2475L (P220) | BA71V-CP2475L (p220) | Core shell | 2049 | APQPDVAQVLNIPNR |
| BA71V-CP2475L (P220) | BA71V-CP2475L (p220) | Core shell | 2050 | PQPDVAQVLNIPNRP |
| BA71V-CP2475L (P220) | BA71V-CP2475L (p220) | Core shell | 2051 | QPDVAQVLNIPNRPP |
| BA71V-CP2475L (P220) | BA71V-CP2475L (p220) | Core shell | 2052 | PDVAQVLNIPNRPPM |
| BA71V-CP2475L (P220) | BA71V-CP2475L (p220) | Core shell | 2053 | DVAQVLNIPNRPPMN |
| BA71V-CP2475L (P220) | BA71V-CP2475L (p220) | Core shell | 2054 | VAQVLNIPNRPPMNT |
| BA71V-CP2475L (P220) | BA71V-CP2475L (p220) | Core shell | 2055 | AQVLNIPNRPPMNTR |
| BA71V-CP2475L (P220) | BA71V-CP2475L (p220) | Core shell | 2056 | QVLNIPNRPPMNTRE |
| BA71V-CP2475L (P220) | BA71V-CP2475L (p220) | Core shell | 2057 | VLNIPNRPPMNTREF |
| BA71V-CP2475L (P220) | BA71V-CP2475L (p220) | Core shell | 2058 | LNIPNRPPMNTREFM |
| BA71V-CP2475L (P220) | BA71V-CP2475L (p220) | Core shell | 2059 | NIPNRPPMNTREFML |
| BA71V-CP2475L (P220) | BA71V-CP2475L (p220) | Core shell | 2060 | IPNRPPMNTREFMLK |
| BA71V-CP2475L (P220) | BA71V-CP2475L (p220) | Core shell | 2061 | PNRPPMNTREFMLKL |
| BA71V-CP2475L (P220) | BA71V-CP2475L (p220) | Core shell | 2062 | NRPPMNTREFMLKLL |
| BA71V-CP2475L (P220) | BA71V-CP2475L (p220) | Core shell | 2063 | RPPMNTREFMLKLLI |
| BA71V-CP2475L (P220) | BA71V-CP2475L (p220) | Core shell | 2064 | PPMNTREFMLKLLIN |
| BA71V-CP2475L (P220) | BA71V-CP2475L (p220) | Core shell | 2065 | PMNTREFMLKLLINP |
| BA71V-CP2475L (P220) | BA71V-CP2475L (p220) | Core shell | 2066 | MNTREFMLKLLINPY |
| BA71V-CP2475L (P220) | BA71V-CP2475L (p220) | Core shell | 2067 | NTREFMLKLLINPYV |
| BA71V-CP2475L (P220) | BA71V-CP2475L (p220) | Core shell | 2068 | TREFMLKLLINPYVT |
| BA71V-CP2475L (P220) | BA71V-CP2475L (p220) | Core shell | 2069 | REFMLKLLINPYVTV |
| BA71V-CP2475L (P220) | BA71V-CP2475L (p220) | Core shell | 2070 | EFMLKLLINPYVTVS |
| BA71V-CP2475L (P220) | BA71V-CP2475L (p220) | Core shell | 2071 | FMLKLLINPYVTVSI |
| BA71V-CP2475L (P220) | BA71V-CP2475L (p220) | Core shell | 2072 | MLKLLINPYVTVSIT |
| BA71V-CP2475L (P220) | BA71V-CP2475L (p220) | Core shell | 2073 | LKLLINPYVTVSITQ |
| BA71V-CP2475L (P220) | BA71V-CP2475L (p220) | Core shell | 2074 | KLLINPYVTVSITQY |
| BA71V-CP2475L (P220) | BA71V-CP2475L (p220) | Core shell | 2075 | LLINPYVTVSITQYG |
| BA71V-CP2475L (P220) | BA71V-CP2475L (p220) | Core shell | 2076 | LINPYVTVSITQYGN |
| BA71V-CP2475L (P220) | BA71V-CP2475L (p220) | Core shell | 2077 | INPYVTVSITQYGNE |
| BA71V-CP2475L (P220) | BA71V-CP2475L (p220) | Core shell | 2078 | NPYVTVSITQYGNEL |
| BA71V-CP2475L (P220) | BA71V-CP2475L (p220) | Core shell | 2079 | PYVTVSITQYGNELL |
| BA71V-CP2475L (P220) | BA71V-CP2475L (p220) | Core shell | 2080 | YVTVSITQYGNELLF |
| BA71V-CP2475L (P220) | BA71V-CP2475L (p220) | Core shell | 2081 | VTVSITQYGNELLFR |
| BA71V-CP2475L (P220) | BA71V-CP2475L (p220) | Core shell | 2082 | TVSITQYGNELLFRG |
| BA71V-CP2475L (P220) | BA71V-CP2475L (p220) | Core shell | 2083 | VSITQYGNELLFRGN |
| BA71V-CP2475L (P220) | BA71V-CP2475L (p220) | Core shell | 2084 | SITQYGNELLFRGNT |
| BA71V-CP2475L (P220) | BA71V-CP2475L (p220) | Core shell | 2085 | ITQYGNELLFRGNTG |
| BA71V-CP2475L (P220) | BA71V-CP2475L (p220) | Core shell | 2086 | TQYGNELLFRGNTGY |
| BA71V-CP2475L (P220) | BA71V-CP2475L (p220) | Core shell | 2087 | QYGNELLFRGNTGYM |
| BA71V-CP2475L (P220) | BA71V-CP2475L (p220) | Core shell | 2088 | YGNELLFRGNTGYMS |
| BA71V-CP2475L (P220) | BA71V-CP2475L (p220) | Core shell | 2089 | GNELLFRGNTGYMSR |
| BA71V-CP2475L (P220) | BA71V-CP2475L (p220) | Core shell | 2090 | NELLFRGNTGYMSRI |
| BA71V-CP2475L (P220) | BA71V-CP2475L (p220) | Core shell | 2091 | ELLFRGNTGYMSRIF |
| BA71V-CP2475L (P220) | BA71V-CP2475L (p220) | Core shell | 2092 | LLFRGNTGYMSRIFR |
| BA71V-CP2475L (P220) | BA71V-CP2475L (p220) | Core shell | 2093 | LFRGNTGYMSRIFRG |
| BA71V-CP2475L (P220) | BA71V-CP2475L (p220) | Core shell | 2094 | FRGNTGYMSRIFRGD |
| BA71V-CP2475L (P220) | BA71V-CP2475L (p220) | Core shell | 2095 | RGNTGYMSRIFRGDN |

| | | | | |
|---|---|---|---|---|
| BA71V-CP2475L (P220) | BA71V-CP2475L (p220) | Core shell | 2096 | GNTGYMSRIFRGDNA |
| BA71V-CP2475L (P220) | BA71V-CP2475L (p220) | Core shell | 2097 | NTGYMSRIFRGDNAL |
| BA71V-CP2475L (P220) | BA71V-CP2475L (p220) | Core shell | 2098 | TGYMSRIFRGDNALN |
| BA71V-CP2475L (P220) | BA71V-CP2475L (p220) | Core shell | 2099 | GYMSRIFRGDNALNM |
| BA71V-CP2475L (P220) | BA71V-CP2475L (p220) | Core shell | 2100 | YMSRIFRGDNALNMG |
| BA71V-CP2475L (P220) | BA71V-CP2475L (p220) | Core shell | 2101 | MSRIFRGDNALNMGR |
| BA71V-CP2475L (P220) | BA71V-CP2475L (p220) | Core shell | 2102 | SRIFRGDNALNMGRP |
| BA71V-CP2475L (P220) | BA71V-CP2475L (p220) | Core shell | 2103 | RIFRGDNALNMGRPK |
| BA71V-CP2475L (P220) | BA71V-CP2475L (p220) | Core shell | 2104 | IFRGDNALNMGRPKF |
| BA71V-CP2475L (P220) | BA71V-CP2475L (p220) | Core shell | 2105 | FRGDNALNMGRPKFL |
| BA71V-CP2475L (P220) | BA71V-CP2475L (p220) | Core shell | 2106 | RGDNALNMGRPKFLS |
| BA71V-CP2475L (P220) | BA71V-CP2475L (p220) | Core shell | 2107 | GDNALNMGRPKFLSD |
| BA71V-CP2475L (P220) | BA71V-CP2475L (p220) | Core shell | 2108 | DNALNMGRPKFLSDQ |
| BA71V-CP2475L (P220) | BA71V-CP2475L (p220) | Core shell | 2109 | NALNMGRPKFLSDQI |
| BA71V-CP2475L (P220) | BA71V-CP2475L (p220) | Core shell | 2110 | ALNMGRPKFLSDQIF |
| BA71V-CP2475L (P220) | BA71V-CP2475L (p220) | Core shell | 2111 | LNMGRPKFLSDQIFN |
| BA71V-CP2475L (P220) | BA71V-CP2475L (p220) | Core shell | 2112 | NMGRPKFLSDQIFNK |
| BA71V-CP2475L (P220) | BA71V-CP2475L (p220) | Core shell | 2113 | MGRPKFLSDQIFNKV |
| BA71V-CP2475L (P220) | BA71V-CP2475L (p220) | Core shell | 2114 | GRPKFLSDQIFNKVL |
| BA71V-CP2475L (P220) | BA71V-CP2475L (p220) | Core shell | 2115 | RPKFLSDQIFNKVLF |
| BA71V-CP2475L (P220) | BA71V-CP2475L (p220) | Core shell | 2116 | PKFLSDQIFNKVLFG |
| BA71V-CP2475L (P220) | BA71V-CP2475L (p220) | Core shell | 2117 | KFLSDQIFNKVLFGS |
| BA71V-CP2475L (P220) | BA71V-CP2475L (p220) | Core shell | 2118 | FLSDQIFNKVLFGSL |
| BA71V-CP2475L (P220) | BA71V-CP2475L (p220) | Core shell | 2119 | LSDQIFNKVLFGSLY |
| BA71V-CP2475L (P220) | BA71V-CP2475L (p220) | Core shell | 2120 | SDQIFNKVLFGSLYP |
| BA71V-CP2475L (P220) | BA71V-CP2475L (p220) | Core shell | 2121 | DQIFNKVLFGSLYPT |
| BA71V-CP2475L (P220) | BA71V-CP2475L (p220) | Core shell | 2122 | QIFNKVLFGSLYPTQ |
| BA71V-CP2475L (P220) | BA71V-CP2475L (p220) | Core shell | 2123 | IFNKVLFGSLYPTQF |
| BA71V-CP2475L (P220) | BA71V-CP2475L (p220) | Core shell | 2124 | FNKVLFGSLYPTQFD |
| BA71V-CP2475L (P220) | BA71V-CP2475L (p220) | Core shell | 2125 | NKVLFGSLYPTQFDY |
| BA71V-CP2475L (P220) | BA71V-CP2475L (p220) | Core shell | 2126 | KVLFGSLYPTQFDYD |
| BA71V-CP2475L (P220) | BA71V-CP2475L (p220) | Core shell | 2127 | VLFGSLYPTQFDYDE |
| BA71V-CP2475L (P220) | BA71V-CP2475L (p220) | Core shell | 2128 | LFGSLYPTQFDYDEA |
| BA71V-CP2475L (P220) | BA71V-CP2475L (p220) | Core shell | 2129 | FGSLYPTQFDYDEAG |
| BA71V-CP2475L (P220) | BA71V-CP2475L (p220) | Core shell | 2130 | GSLYPTQFDYDEAGP |
| BA71V-CP2475L (P220) | BA71V-CP2475L (p220) | Core shell | 2131 | SLYPTQFDYDEAGPG |
| BA71V-CP2475L (P220) | BA71V-CP2475L (p220) | Core shell | 2132 | LYPTQFDYDEAGPGL |
| BA71V-CP2475L (P220) | BA71V-CP2475L (p220) | Core shell | 2133 | YPTQFDYDEAGPGLA |
| BA71V-CP2475L (P220) | BA71V-CP2475L (p220) | Core shell | 2134 | PTQFDYDEAGPGLAA |
| BA71V-CP2475L (P220) | BA71V-CP2475L (p220) | Core shell | 2135 | TQFDYDEAGPGLAAG |
| BA71V-CP2475L (P220) | BA71V-CP2475L (p220) | Core shell | 2136 | QFDYDEAGPGLAAGI |
| BA71V-CP2475L (P220) | BA71V-CP2475L (p220) | Core shell | 2137 | FDYDEAGPGLAAGIQ |
| BA71V-CP2475L (P220) | BA71V-CP2475L (p220) | Core shell | 2138 | DYDEAGPGLAAGIQR |
| BA71V-CP2475L (P220) | BA71V-CP2475L (p220) | Core shell | 2139 | YDEAGPGLAAGIQRG |
| BA71V-CP2475L (P220) | BA71V-CP2475L (p220) | Core shell | 2140 | DEAGPGLAAGIQRGR |
| BA71V-CP2475L (P220) | BA71V-CP2475L (p220) | Core shell | 2141 | EAGPGLAAGIQRGRE |
| BA71V-CP2475L (P220) | BA71V-CP2475L (p220) | Core shell | 2142 | AGPGLAAGIQRGREQ |
| BA71V-CP2475L (P220) | BA71V-CP2475L (p220) | Core shell | 2143 | GPGLAAGIQRGREQW |
| BA71V-CP2475L (P220) | BA71V-CP2475L (p220) | Core shell | 2144 | PGLAAGIQRGREQWG |
| BA71V-CP2475L (P220) | BA71V-CP2475L (p220) | Core shell | 2145 | GLAAGIQRGREQWGQ |
| BA71V-CP2475L (P220) | BA71V-CP2475L (p220) | Core shell | 2146 | LAAGIQRGREQWGQP |
| BA71V-CP2475L (P220) | BA71V-CP2475L (p220) | Core shell | 2147 | AAGIQRGREQWGQPF |
| BA71V-CP2475L (P220) | BA71V-CP2475L (p220) | Core shell | 2148 | AGIQRGREQWGQPFS |
| BA71V-CP2475L (P220) | BA71V-CP2475L (p220) | Core shell | 2149 | GIQRGREQWGQPFSD |
| BA71V-CP2475L (P220) | BA71V-CP2475L (p220) | Core shell | 2150 | IQRGREQWGQPFSDY |
| BA71V-CP2475L (P220) | BA71V-CP2475L (p220) | Core shell | 2151 | QRGREQWGQPFSDYI |
| BA71V-CP2475L (P220) | BA71V-CP2475L (p220) | Core shell | 2152 | RGREQWGQPFSDYIN |
| BA71V-CP2475L (P220) | BA71V-CP2475L (p220) | Core shell | 2153 | GREQWGQPFSDYINQ |
| BA71V-CP2475L (P220) | BA71V-CP2475L (p220) | Core shell | 2154 | REQWGQPFSDYINQA |
| BA71V-CP2475L (P220) | BA71V-CP2475L (p220) | Core shell | 2155 | EQWGQPFSDYINQAL |
| BA71V-CP2475L (P220) | BA71V-CP2475L (p220) | Core shell | 2156 | QWGQPFSDYINQALH |
| BA71V-CP2475L (P220) | BA71V-CP2475L (p220) | Core shell | 2157 | WGQPFSDYINQALHE |
| BA71V-CP2475L (P220) | BA71V-CP2475L (p220) | Core shell | 2158 | GQPFSDYINQALHEL |
| BA71V-CP2475L (P220) | BA71V-CP2475L (p220) | Core shell | 2159 | QPFSDYINQALHELV |
| BA71V-CP2475L (P220) | BA71V-CP2475L (p220) | Core shell | 2160 | PFSDYINQALHELVR |
| BA71V-CP2475L (P220) | BA71V-CP2475L (p220) | Core shell | 2161 | FSDYINQALHELVRT |
| BA71V-CP2475L (P220) | BA71V-CP2475L (p220) | Core shell | 2162 | SDYINQALHELVRTI |

| | | | | |
|---|---|---|---|---|
| BA71V-CP2475L (P220) | BA71V-CP2475L (p220) | Core shell | 2163 | DYINQALHELVRTIR |
| BA71V-CP2475L (P220) | BA71V-CP2475L (p220) | Core shell | 2164 | YINQALHELVRTIRI |
| BA71V-CP2475L (P220) | BA71V-CP2475L (p220) | Core shell | 2165 | INQALHELVRTIRIP |
| BA71V-CP2475L (P220) | BA71V-CP2475L (p220) | Core shell | 2166 | NQALHELVRTIRIPQ |
| BA71V-CP2475L (P220) | BA71V-CP2475L (p220) | Core shell | 2167 | QALHELVRTIRIPQN |
| BA71V-CP2475L (P220) | BA71V-CP2475L (p220) | Core shell | 2168 | ALHELVRTIRIPQNL |
| BA71V-CP2475L (P220) | BA71V-CP2475L (p220) | Core shell | 2169 | LHELVRTIRIPQNLR |
| BA71V-CP2475L (P220) | BA71V-CP2475L (p220) | Core shell | 2170 | HELVRTIRIPQNLRV |
| BA71V-CP2475L (P220) | BA71V-CP2475L (p220) | Core shell | 2171 | ELVRTIRIPQNLRVL |
| BA71V-CP2475L (P220) | BA71V-CP2475L (p220) | Core shell | 2172 | LVRTIRIPQNLRVLR |
| BA71V-CP2475L (P220) | BA71V-CP2475L (p220) | Core shell | 2173 | VRTIRIPQNLRVLRN |
| BA71V-CP2475L (P220) | BA71V-CP2475L (p220) | Core shell | 2174 | RTIRIPQNLRVLRNI |
| BA71V-CP2475L (P220) | BA71V-CP2475L (p220) | Core shell | 2175 | TIRIPQNLRVLRNIM |
| BA71V-CP2475L (P220) | BA71V-CP2475L (p220) | Core shell | 2176 | IRIPQNLRVLRNIMV |
| BA71V-CP2475L (P220) | BA71V-CP2475L (p220) | Core shell | 2177 | RIPQNLRVLRNIMVK |
| BA71V-CP2475L (P220) | BA71V-CP2475L (p220) | Core shell | 2178 | IPQNLRVLRNIMVKN |
| BA71V-CP2475L (P220) | BA71V-CP2475L (p220) | Core shell | 2179 | PQNLRVLRNIMVKNQ |
| BA71V-CP2475L (P220) | BA71V-CP2475L (p220) | Core shell | 2180 | QNLRVLRNIMVKNQL |
| BA71V-CP2475L (P220) | BA71V-CP2475L (p220) | Core shell | 2181 | NLRVLRNIMVKNQLI |
| BA71V-CP2475L (P220) | BA71V-CP2475L (p220) | Core shell | 2182 | LRVLRNIMVKNQLIA |
| BA71V-CP2475L (P220) | BA71V-CP2475L (p220) | Core shell | 2183 | RVLRNIMVKNQLIAD |
| BA71V-CP2475L (P220) | BA71V-CP2475L (p220) | Core shell | 2184 | VLRNIMVKNQLIADL |
| BA71V-CP2475L (P220) | BA71V-CP2475L (p220) | Core shell | 2185 | LRNIMVKNQLIADLA |
| BA71V-CP2475L (P220) | BA71V-CP2475L (p220) | Core shell | 2186 | RNIMVKNQLIADLAA |
| BA71V-CP2475L (P220) | BA71V-CP2475L (p220) | Core shell | 2187 | NIMVKNQLIADLAAI |
| BA71V-CP2475L (P220) | BA71V-CP2475L (p220) | Core shell | 2188 | IMVKNQLIADLAAIR |
| BA71V-CP2475L (P220) | BA71V-CP2475L (p220) | Core shell | 2189 | MVKNQLIADLAAIRE |
| BA71V-CP2475L (P220) | BA71V-CP2475L (p220) | Core shell | 2190 | VKNQLIADLAAIREQ |
| BA71V-CP2475L (P220) | BA71V-CP2475L (p220) | Core shell | 2191 | KNQLIADLAAIREQL |
| BA71V-CP2475L (P220) | BA71V-CP2475L (p220) | Core shell | 2192 | NQLIADLAAIREQLV |
| BA71V-CP2475L (P220) | BA71V-CP2475L (p220) | Core shell | 2193 | QLIADLAAIREQLVR |
| BA71V-CP2475L (P220) | BA71V-CP2475L (p220) | Core shell | 2194 | LIADLAAIREQLVRM |
| BA71V-CP2475L (P220) | BA71V-CP2475L (p220) | Core shell | 2195 | IADLAAIREQLVRMR |
| BA71V-CP2475L (P220) | BA71V-CP2475L (p220) | Core shell | 2196 | ADLAAIREQLVRMRR |
| BA71V-CP2475L (P220) | BA71V-CP2475L (p220) | Core shell | 2197 | DLAAIREQLVRMRRE |
| BA71V-CP2475L (P220) | BA71V-CP2475L (p220) | Core shell | 2198 | LAAIREQLVRMRREV |
| BA71V-CP2475L (P220) | BA71V-CP2475L (p220) | Core shell | 2199 | AAIREQLVRMRREVE |
| BA71V-CP2475L (P220) | BA71V-CP2475L (p220) | Core shell | 2200 | AIREQLVRMRREVEN |
| BA71V-CP2475L (P220) | BA71V-CP2475L (p220) | Core shell | 2201 | IREQLVRMRREVENM |
| BA71V-CP2475L (P220) | BA71V-CP2475L (p220) | Core shell | 2202 | REQLVRMRREVENMV |
| BA71V-CP2475L (P220) | BA71V-CP2475L (p220) | Core shell | 2203 | EQLVRMRREVENMVQ |
| BA71V-CP2475L (P220) | BA71V-CP2475L (p220) | Core shell | 2204 | QLVRMRREVENMVQT |
| BA71V-CP2475L (P220) | BA71V-CP2475L (p220) | Core shell | 2205 | LVRMRREVENMVQTP |
| BA71V-CP2475L (P220) | BA71V-CP2475L (p220) | Core shell | 2206 | VRMRREVENMVQTPE |
| BA71V-CP2475L (P220) | BA71V-CP2475L (p220) | Core shell | 2207 | RMRREVENMVQTPEI |
| BA71V-CP2475L (P220) | BA71V-CP2475L (p220) | Core shell | 2208 | MRREVENMVQTPEIQ |
| BA71V-CP2475L (P220) | BA71V-CP2475L (p220) | Core shell | 2209 | RREVENMVQTPEIQN |
| BA71V-CP2475L (P220) | BA71V-CP2475L (p220) | Core shell | 2210 | REVENMVQTPEIQNN |
| BA71V-CP2475L (P220) | BA71V-CP2475L (p220) | Core shell | 2211 | EVENMVQTPEIQNNP |
| BA71V-CP2475L (P220) | BA71V-CP2475L (p220) | Core shell | 2212 | VENMVQTPEIQNNPT |
| BA71V-CP2475L (P220) | BA71V-CP2475L (p220) | Core shell | 2213 | ENMVQTPEIQNNPTP |
| BA71V-CP2475L (P220) | BA71V-CP2475L (p220) | Core shell | 2214 | NMVQTPEIQNNPTPE |
| BA71V-CP2475L (P220) | BA71V-CP2475L (p220) | Core shell | 2215 | MVQTPEIQNNPTPEV |
| BA71V-CP2475L (P220) | BA71V-CP2475L (p220) | Core shell | 2216 | VQTPEIQNNPTPEVI |
| BA71V-CP2475L (P220) | BA71V-CP2475L (p220) | Core shell | 2217 | QTPEIQNNPTPEVIA |
| BA71V-CP2475L (P220) | BA71V-CP2475L (p220) | Core shell | 2218 | TPEIQNNPTPEVIAA |
| BA71V-CP2475L (P220) | BA71V-CP2475L (p220) | Core shell | 2219 | PEIQNNPTPEVIAAA |
| BA71V-CP2475L (P220) | BA71V-CP2475L (p220) | Core shell | 2220 | EIQNNPTPEVIAAAQ |
| BA71V-CP2475L (P220) | BA71V-CP2475L (p220) | Core shell | 2221 | IQNNPTPEVIAAAQT |
| BA71V-CP2475L (P220) | BA71V-CP2475L (p220) | Core shell | 2222 | QNNPTPEVIAAAQTW |
| BA71V-CP2475L (P220) | BA71V-CP2475L (p220) | Core shell | 2223 | NNPTPEVIAAAQTWA |
| BA71V-CP2475L (P220) | BA71V-CP2475L (p220) | Core shell | 2224 | NPTPEVIAAAQTWAQ |
| BA71V-CP2475L (P220) | BA71V-CP2475L (p220) | Core shell | 2225 | PTPEVIAAAQTWAQQ |
| BA71V-CP2475L (P220) | BA71V-CP2475L (p220) | Core shell | 2226 | TPEVIAAAQTWAQQY |
| BA71V-CP2475L (P220) | BA71V-CP2475L (p220) | Core shell | 2227 | PEVIAAAQTWAQQYR |
| BA71V-CP2475L (P220) | BA71V-CP2475L (p220) | Core shell | 2228 | EVIAAAQTWAQQYRA |
| BA71V-CP2475L (P220) | BA71V-CP2475L (p220) | Core shell | 2229 | VIAAAQTWAQQYRAR |

| | | | | |
|---|---|---|---|---|
| BA71V-CP2475L (P220) | BA71V-CP2475L (p220) | Core shell | 2230 | IAAAQTWAQQYRARV |
| BA71V-CP2475L (P220) | BA71V-CP2475L (p220) | Core shell | 2231 | AAAQTWAQQYRARVD |
| BA71V-CP2475L (P220) | BA71V-CP2475L (p220) | Core shell | 2232 | AAQTWAQQYRARVDF |
| BA71V-CP2475L (P220) | BA71V-CP2475L (p220) | Core shell | 2233 | AQTWAQQYRARVDFL |
| BA71V-CP2475L (P220) | BA71V-CP2475L (p220) | Core shell | 2234 | QTWAQQYRARVDFLI |
| BA71V-CP2475L (P220) | BA71V-CP2475L (p220) | Core shell | 2235 | TWAQQYRARVDFLIN |
| BA71V-CP2475L (P220) | BA71V-CP2475L (p220) | Core shell | 2236 | WAQQYRARVDFLINF |
| BA71V-CP2475L (P220) | BA71V-CP2475L (p220) | Core shell | 2237 | AQQYRARVDFLINFI |
| BA71V-CP2475L (P220) | BA71V-CP2475L (p220) | Core shell | 2238 | QQYRARVDFLINFIG |
| BA71V-CP2475L (P220) | BA71V-CP2475L (p220) | Core shell | 2239 | QYRARVDFLINFIGN |
| BA71V-CP2475L (P220) | BA71V-CP2475L (p220) | Core shell | 2240 | YRARVDFLINFIGNA |
| BA71V-CP2475L (P220) | BA71V-CP2475L (p220) | Core shell | 2241 | RARVDFLINFIGNAQ |
| BA71V-CP2475L (P220) | BA71V-CP2475L (p220) | Core shell | 2242 | ARVDFLINFIGNAQQ |
| BA71V-CP2475L (P220) | BA71V-CP2475L (p220) | Core shell | 2243 | RVDFLINFIGNAQQP |
| BA71V-CP2475L (P220) | BA71V-CP2475L (p220) | Core shell | 2244 | VDFLINFIGNAQQPN |
| BA71V-CP2475L (P220) | BA71V-CP2475L (p220) | Core shell | 2245 | DFLINFIGNAQQPNS |
| BA71V-CP2475L (P220) | BA71V-CP2475L (p220) | Core shell | 2246 | FLINFIGNAQQPNSL |
| BA71V-CP2475L (P220) | BA71V-CP2475L (p220) | Core shell | 2247 | LINFIGNAQQPNSLI |
| BA71V-CP2475L (P220) | BA71V-CP2475L (p220) | Core shell | 2248 | INFIGNAQQPNSLIQ |
| BA71V-CP2475L (P220) | BA71V-CP2475L (p220) | Core shell | 2249 | NFIGNAQQPNSLIQL |
| BA71V-CP2475L (P220) | BA71V-CP2475L (p220) | Core shell | 2250 | FIGNAQQPNSLIQLI |
| BA71V-CP2475L (P220) | BA71V-CP2475L (p220) | Core shell | 2251 | IGNAQQPNSLIQLIQ |
| BA71V-CP2475L (P220) | BA71V-CP2475L (p220) | Core shell | 2252 | GNAQQPNSLIQLIQN |
| BA71V-CP2475L (P220) | BA71V-CP2475L (p220) | Core shell | 2253 | NAQQPNSLIQLIQNI |
| BA71V-CP2475L (P220) | BA71V-CP2475L (p220) | Core shell | 2254 | AQQPNSLIQLIQNIT |
| BA71V-CP2475L (P220) | BA71V-CP2475L (p220) | Core shell | 2255 | QQPNSLIQLIQNITP |
| BA71V-CP2475L (P220) | BA71V-CP2475L (p220) | Core shell | 2256 | QPNSLIQLIQNITPL |
| BA71V-CP2475L (P220) | BA71V-CP2475L (p220) | Core shell | 2257 | PNSLIQLIQNITPLT |
| BA71V-CP2475L (P220) | BA71V-CP2475L (p220) | Core shell | 2258 | NSLIQLIQNITPLTV |
| BA71V-CP2475L (P220) | BA71V-CP2475L (p220) | Core shell | 2259 | SLIQLIQNITPLTVR |
| BA71V-CP2475L (P220) | BA71V-CP2475L (p220) | Core shell | 2260 | LIQLIQNITPLTVRA |
| BA71V-CP2475L (P220) | BA71V-CP2475L (p220) | Core shell | 2261 | IQLIQNITPLTVRAQ |
| BA71V-CP2475L (P220) | BA71V-CP2475L (p220) | Core shell | 2262 | QLIQNITPLTVRAQL |
| BA71V-CP2475L (P220) | BA71V-CP2475L (p220) | Core shell | 2263 | LIQNITPLTVRAQLT |
| BA71V-CP2475L (P220) | BA71V-CP2475L (p220) | Core shell | 2264 | IQNITPLTVRAQLTT |
| BA71V-CP2475L (P220) | BA71V-CP2475L (p220) | Core shell | 2265 | QNITPLTVRAQLTTV |
| BA71V-CP2475L (P220) | BA71V-CP2475L (p220) | Core shell | 2266 | NITPLTVRAQLTTVF |
| BA71V-CP2475L (P220) | BA71V-CP2475L (p220) | Core shell | 2267 | ITPLTVRAQLTTVFI |
| BA71V-CP2475L (P220) | BA71V-CP2475L (p220) | Core shell | 2268 | TPLTVRAQLTTVFIR |
| BA71V-CP2475L (P220) | BA71V-CP2475L (p220) | Core shell | 2269 | PLTVRAQLTTVFIRH |
| BA71V-CP2475L (P220) | BA71V-CP2475L (p220) | Core shell | 2270 | LTVRAQLTTVFIRHG |
| BA71V-CP2475L (P220) | BA71V-CP2475L (p220) | Core shell | 2271 | TVRAQLTTVFIRHGL |
| BA71V-CP2475L (P220) | BA71V-CP2475L (p220) | Core shell | 2272 | VRAQLTTVFIRHGLP |
| BA71V-CP2475L (P220) | BA71V-CP2475L (p220) | Core shell | 2273 | RAQLTTVFIRHGLPV |
| BA71V-CP2475L (P220) | BA71V-CP2475L (p220) | Core shell | 2274 | AQLTTVFIRHGLPVP |
| BA71V-CP2475L (P220) | BA71V-CP2475L (p220) | Core shell | 2275 | QLTTVFIRHGLPVPD |
| BA71V-CP2475L (P220) | BA71V-CP2475L (p220) | Core shell | 2276 | LTTVFIRHGLPVPDP |
| BA71V-CP2475L (P220) | BA71V-CP2475L (p220) | Core shell | 2277 | TTVFIRHGLPVPDPD |
| BA71V-CP2475L (P220) | BA71V-CP2475L (p220) | Core shell | 2278 | TVFIRHGLPVPDPDQ |
| BA71V-CP2475L (P220) | BA71V-CP2475L (p220) | Core shell | 2279 | VFIRHGLPVPDPDQA |
| BA71V-CP2475L (P220) | BA71V-CP2475L (p220) | Core shell | 2280 | FIRHGLPVPDPDQAL |
| BA71V-CP2475L (P220) | BA71V-CP2475L (p220) | Core shell | 2281 | IRHGLPVPDPDQALQ |
| BA71V-CP2475L (P220) | BA71V-CP2475L (p220) | Core shell | 2282 | RHGLPVPDPDQALQT |
| BA71V-CP2475L (P220) | BA71V-CP2475L (p220) | Core shell | 2283 | HGLPVPDPDQALQTD |
| BA71V-CP2475L (P220) | BA71V-CP2475L (p220) | Core shell | 2284 | GLPVPDPDQALQTDD |
| BA71V-CP2475L (P220) | BA71V-CP2475L (p220) | Core shell | 2285 | LPVPDPDQALQTDDE |
| BA71V-CP2475L (P220) | BA71V-CP2475L (p220) | Core shell | 2286 | PVPDPDQALQTDDEA |
| BA71V-CP2475L (P220) | BA71V-CP2475L (p220) | Core shell | 2287 | VPDPDQALQTDDEAT |
| BA71V-CP2475L (P220) | BA71V-CP2475L (p220) | Core shell | 2288 | PDPDQALQTDDEATQ |
| BA71V-CP2475L (P220) | BA71V-CP2475L (p220) | Core shell | 2289 | DPDQALQTDDEATQW |
| BA71V-CP2475L (P220) | BA71V-CP2475L (p220) | Core shell | 2290 | PDQALQTDDEATQWF |
| BA71V-CP2475L (P220) | BA71V-CP2475L (p220) | Core shell | 2291 | DQALQTDDEATQWFM |
| BA71V-CP2475L (P220) | BA71V-CP2475L (p220) | Core shell | 2292 | QALQTDDEATQWFMT |
| BA71V-CP2475L (P220) | BA71V-CP2475L (p220) | Core shell | 2293 | ALQTDDEATQWFMTN |
| BA71V-CP2475L (P220) | BA71V-CP2475L (p220) | Core shell | 2294 | LQTDDEATQWFMTNI |
| BA71V-CP2475L (P220) | BA71V-CP2475L (p220) | Core shell | 2295 | QTDDEATQWFMTNII |
| BA71V-CP2475L (P220) | BA71V-CP2475L (p220) | Core shell | 2296 | TDDEATQWFMTNIIN |

| | | | | |
|---|---|---|---|---|
| BA71V-CP2475L (P220) | BA71V-CP2475L (p220) | Core shell | 2297 | DDEATQWFMTNIINQ |
| BA71V-CP2475L (P220) | BA71V-CP2475L (p220) | Core shell | 2298 | DEATQWFMTNIINQP |
| BA71V-CP2475L (P220) | BA71V-CP2475L (p220) | Core shell | 2299 | EATQWFMTNIINQPI |
| BA71V-CP2475L (P220) | BA71V-CP2475L (p220) | Core shell | 2300 | ATQWFMTNIINQPIT |
| BA71V-CP2475L (P220) | BA71V-CP2475L (p220) | Core shell | 2301 | TQWFMTNIINQPITM |
| BA71V-CP2475L (P220) | BA71V-CP2475L (p220) | Core shell | 2302 | QWFMTNIINQPITMI |
| BA71V-CP2475L (P220) | BA71V-CP2475L (p220) | Core shell | 2303 | WFMTNIINQPITMIT |
| BA71V-CP2475L (P220) | BA71V-CP2475L (p220) | Core shell | 2304 | FMTNIINQPITMITP |
| BA71V-CP2475L (P220) | BA71V-CP2475L (p220) | Core shell | 2305 | MTNIINQPITMITPF |
| BA71V-CP2475L (P220) | BA71V-CP2475L (p220) | Core shell | 2306 | TNIINQPITMITPFT |
| BA71V-CP2475L (P220) | BA71V-CP2475L (p220) | Core shell | 2307 | NIINQPITMITPFTD |
| BA71V-CP2475L (P220) | BA71V-CP2475L (p220) | Core shell | 2308 | IINQPITMITPFTDL |
| BA71V-CP2475L (P220) | BA71V-CP2475L (p220) | Core shell | 2309 | INQPITMITPFTDLA |
| BA71V-CP2475L (P220) | BA71V-CP2475L (p220) | Core shell | 2310 | NQPITMITPFTDLAD |
| BA71V-CP2475L (P220) | BA71V-CP2475L (p220) | Core shell | 2311 | QPITMITPFTDLADD |
| BA71V-CP2475L (P220) | BA71V-CP2475L (p220) | Core shell | 2312 | PITMITPFTDLADDL |
| BA71V-CP2475L (P220) | BA71V-CP2475L (p220) | Core shell | 2313 | ITMITPFTDLADDLR |
| BA71V-CP2475L (P220) | BA71V-CP2475L (p220) | Core shell | 2314 | TMITPFTDLADDLRI |
| BA71V-CP2475L (P220) | BA71V-CP2475L (p220) | Core shell | 2315 | MITPFTDLADDLRIF |
| BA71V-CP2475L (P220) | BA71V-CP2475L (p220) | Core shell | 2316 | ITPFTDLADDLRIFL |
| BA71V-CP2475L (P220) | BA71V-CP2475L (p220) | Core shell | 2317 | TPFTDLADDLRIFLE |
| BA71V-CP2475L (P220) | BA71V-CP2475L (p220) | Core shell | 2318 | PFTDLADDLRIFLET |
| BA71V-CP2475L (P220) | BA71V-CP2475L (p220) | Core shell | 2319 | FTDLADDLRIFLETL |
| BA71V-CP2475L (P220) | BA71V-CP2475L (p220) | Core shell | 2320 | TDLADDLRIFLETLE |
| BA71V-CP2475L (P220) | BA71V-CP2475L (p220) | Core shell | 2321 | DLADDLRIFLETLER |
| BA71V-CP2475L (P220) | BA71V-CP2475L (p220) | Core shell | 2322 | LADDLRIFLETLERY |
| BA71V-CP2475L (P220) | BA71V-CP2475L (p220) | Core shell | 2323 | ADDLRIFLETLERYV |
| BA71V-CP2475L (P220) | BA71V-CP2475L (p220) | Core shell | 2324 | DDLRIFLETLERYVF |
| BA71V-CP2475L (P220) | BA71V-CP2475L (p220) | Core shell | 2325 | DLRIFLETLERYVFN |
| BA71V-CP2475L (P220) | BA71V-CP2475L (p220) | Core shell | 2326 | LRIFLETLERYVFNV |
| BA71V-CP2475L (P220) | BA71V-CP2475L (p220) | Core shell | 2327 | RIFLETLERYVFNVP |
| BA71V-CP2475L (P220) | BA71V-CP2475L (p220) | Core shell | 2328 | IFLETLERYVFNVPR |
| BA71V-CP2475L (P220) | BA71V-CP2475L (p220) | Core shell | 2329 | FLETLERYVFNVPRW |
| BA71V-CP2475L (P220) | BA71V-CP2475L (p220) | Core shell | 2330 | LETLERYVFNVPRWL |
| BA71V-CP2475L (P220) | BA71V-CP2475L (p220) | Core shell | 2331 | ETLERYVFNVPRWLG |
| BA71V-CP2475L (P220) | BA71V-CP2475L (p220) | Core shell | 2332 | TLERYVFNVPRWLGP |
| BA71V-CP2475L (P220) | BA71V-CP2475L (p220) | Core shell | 2333 | LERYVFNVPRWLGPS |
| BA71V-CP2475L (P220) | BA71V-CP2475L (p220) | Core shell | 2334 | ERYVFNVPRWLGPST |
| BA71V-CP2475L (P220) | BA71V-CP2475L (p220) | Core shell | 2335 | RYVFNVPRWLGPSTG |
| BA71V-CP2475L (P220) | BA71V-CP2475L (p220) | Core shell | 2336 | YVFNVPRWLGPSTGR |
| BA71V-CP2475L (P220) | BA71V-CP2475L (p220) | Core shell | 2337 | VFNVPRWLGPSTGRV |
| BA71V-CP2475L (P220) | BA71V-CP2475L (p220) | Core shell | 2338 | FNVPRWLGPSTGRVA |
| BA71V-CP2475L (P220) | BA71V-CP2475L (p220) | Core shell | 2339 | NVPRWLGPSTGRVAR |
| BA71V-CP2475L (P220) | BA71V-CP2475L (p220) | Core shell | 2340 | VPRWLGPSTGRVARV |
| BA71V-CP2475L (P220) | BA71V-CP2475L (p220) | Core shell | 2341 | PRWLGPSTGRVARVP |
| BA71V-CP2475L (P220) | BA71V-CP2475L (p220) | Core shell | 2342 | RWLGPSTGRVARVPV |
| BA71V-CP2475L (P220) | BA71V-CP2475L (p220) | Core shell | 2343 | WLGPSTGRVARVPVN |
| BA71V-CP2475L (P220) | BA71V-CP2475L (p220) | Core shell | 2344 | LGPSTGRVARVPVNM |
| BA71V-CP2475L (P220) | BA71V-CP2475L (p220) | Core shell | 2345 | GPSTGRVARVPVNMA |
| BA71V-CP2475L (P220) | BA71V-CP2475L (p220) | Core shell | 2346 | PSTGRVARVPVNMAP |
| BA71V-CP2475L (P220) | BA71V-CP2475L (p220) | Core shell | 2347 | STGRVARVPVNMAPG |
| BA71V-CP2475L (P220) | BA71V-CP2475L (p220) | Core shell | 2348 | TGRVARVPVNMAPGN |
| BA71V-CP2475L (P220) | BA71V-CP2475L (p220) | Core shell | 2349 | GRVARVPVNMAPGNM |
| BA71V-CP2475L (P220) | BA71V-CP2475L (p220) | Core shell | 2350 | RVARVPVNMAPGNMR |
| BA71V-CP2475L (P220) | BA71V-CP2475L (p220) | Core shell | 2351 | VARVPVNMAPGNMRY |
| BA71V-CP2475L (P220) | BA71V-CP2475L (p220) | Core shell | 2352 | ARVPVNMAPGNMRYR |
| BA71V-CP2475L (P220) | BA71V-CP2475L (p220) | Core shell | 2353 | RVPVNMAPGNMRYRT |
| BA71V-CP2475L (P220) | BA71V-CP2475L (p220) | Core shell | 2354 | VPVNMAPGNMRYRTS |
| BA71V-CP2475L (P220) | BA71V-CP2475L (p220) | Core shell | 2355 | PVNMAPGNMRYRTSY |
| BA71V-CP2475L (P220) | BA71V-CP2475L (p220) | Core shell | 2356 | VNMAPGNMRYRTSYT |
| BA71V-CP2475L (P220) | BA71V-CP2475L (p220) | Core shell | 2357 | NMAPGNMRYRTSYTE |
| BA71V-CP2475L (P220) | BA71V-CP2475L (p220) | Core shell | 2358 | MAPGNMRYRTSYTEN |
| BA71V-CP2475L (P220) | BA71V-CP2475L (p220) | Core shell | 2359 | APGNMRYRTSYTENS |
| BA71V-CP2475L (P220) | BA71V-CP2475L (p220) | Core shell | 2360 | PGNMRYRTSYTENSV |
| BA71V-CP2475L (P220) | BA71V-CP2475L (p220) | Core shell | 2361 | GNMRYRTSYTENSVL |
| BA71V-CP2475L (P220) | BA71V-CP2475L (p220) | Core shell | 2362 | NMRYRTSYTENSVLT |
| BA71V-CP2475L (P220) | BA71V-CP2475L (p220) | Core shell | 2363 | MRYRTSYTENSVLTY |

| | | | | |
|---|---|---|---|---|
| BA71V-CP2475L (P220) | BA71V-CP2475L (p220) | Core shell | 2364 | RYRTSYTENSVLTYI |
| BA71V-CP2475L (P220) | BA71V-CP2475L (p220) | Core shell | 2365 | YRTSYTENSVLTYIA |
| BA71V-CP2475L (P220) | BA71V-CP2475L (p220) | Core shell | 2366 | RTSYTENSVLTYIAE |
| BA71V-CP2475L (P220) | BA71V-CP2475L (p220) | Core shell | 2367 | TSYTENSVLTYIAEQ |
| BA71V-CP2475L (P220) | BA71V-CP2475L (p220) | Core shell | 2368 | SYTENSVLTYIAEQN |
| BA71V-CP2475L (P220) | BA71V-CP2475L (p220) | Core shell | 2369 | YTENSVLTYIAEQNQ |
| BA71V-CP2475L (P220) | BA71V-CP2475L (p220) | Core shell | 2370 | TENSVLTYIAEQNQE |
| BA71V-CP2475L (P220) | BA71V-CP2475L (p220) | Core shell | 2371 | ENSVLTYIAEQNQEE |
| BA71V-CP2475L (P220) | BA71V-CP2475L (p220) | Core shell | 2372 | NSVLTYIAEQNQEEG |
| BA71V-CP2475L (P220) | BA71V-CP2475L (p220) | Core shell | 2373 | SVLTYIAEQNQEEGP |
| BA71V-CP2475L (P220) | BA71V-CP2475L (p220) | Core shell | 2374 | VLTYIAEQNQEEGPW |
| BA71V-CP2475L (P220) | BA71V-CP2475L (p220) | Core shell | 2375 | LTYIAEQNQEEGPWS |
| BA71V-CP2475L (P220) | BA71V-CP2475L (p220) | Core shell | 2376 | TYIAEQNQEEGPWSI |
| BA71V-CP2475L (P220) | BA71V-CP2475L (p220) | Core shell | 2377 | YIAEQNQEEGPWSIV |
| BA71V-CP2475L (P220) | BA71V-CP2475L (p220) | Core shell | 2378 | IAEQNQEEGPWSIVK |
| BA71V-CP2475L (P220) | BA71V-CP2475L (p220) | Core shell | 2379 | AEQNQEEGPWSIVKQ |
| BA71V-CP2475L (P220) | BA71V-CP2475L (p220) | Core shell | 2380 | EQNQEEGPWSIVKQV |
| BA71V-CP2475L (P220) | BA71V-CP2475L (p220) | Core shell | 2381 | QNQEEGPWSIVKQVG |
| BA71V-CP2475L (P220) | BA71V-CP2475L (p220) | Core shell | 2382 | NQEEGPWSIVKQVGV |
| BA71V-CP2475L (P220) | BA71V-CP2475L (p220) | Core shell | 2383 | QEEGPWSIVKQVGVG |
| BA71V-CP2475L (P220) | BA71V-CP2475L (p220) | Core shell | 2384 | EEGPWSIVKQVGVGI |
| BA71V-CP2475L (P220) | BA71V-CP2475L (p220) | Core shell | 2385 | EGPWSIVKQVGVGIQ |
| BA71V-CP2475L (P220) | BA71V-CP2475L (p220) | Core shell | 2386 | GPWSIVKQVGVGIQK |
| BA71V-CP2475L (P220) | BA71V-CP2475L (p220) | Core shell | 2387 | PWSIVKQVGVGIQKP |
| BA71V-CP2475L (P220) | BA71V-CP2475L (p220) | Core shell | 2388 | WSIVKQVGVGIQKPA |
| BA71V-CP2475L (P220) | BA71V-CP2475L (p220) | Core shell | 2389 | SIVKQVGVGIQKPAL |
| BA71V-CP2475L (P220) | BA71V-CP2475L (p220) | Core shell | 2390 | IVKQVGVGIQKPALI |
| BA71V-CP2475L (P220) | BA71V-CP2475L (p220) | Core shell | 2391 | VKQVGVGIQKPALIQ |
| BA71V-CP2475L (P220) | BA71V-CP2475L (p220) | Core shell | 2392 | KQVGVGIQKPALIQI |
| BA71V-CP2475L (P220) | BA71V-CP2475L (p220) | Core shell | 2393 | QVGVGIQKPALIQIG |
| BA71V-CP2475L (P220) | BA71V-CP2475L (p220) | Core shell | 2394 | VGVGIQKPALIQIGK |
| BA71V-CP2475L (P220) | BA71V-CP2475L (p220) | Core shell | 2395 | GVGIQKPALIQIGKD |
| BA71V-CP2475L (P220) | BA71V-CP2475L (p220) | Core shell | 2396 | VGIQKPALIQIGKDR |
| BA71V-CP2475L (P220) | BA71V-CP2475L (p220) | Core shell | 2397 | GIQKPALIQIGKDRF |
| BA71V-CP2475L (P220) | BA71V-CP2475L (p220) | Core shell | 2398 | IQKPALIQIGKDRFD |
| BA71V-CP2475L (P220) | BA71V-CP2475L (p220) | Core shell | 2399 | QKPALIQIGKDRFDT |
| BA71V-CP2475L (P220) | BA71V-CP2475L (p220) | Core shell | 2400 | KPALIQIGKDRFDTR |
| BA71V-CP2475L (P220) | BA71V-CP2475L (p220) | Core shell | 2401 | PALIQIGKDRFDTRL |
| BA71V-CP2475L (P220) | BA71V-CP2475L (p220) | Core shell | 2402 | ALIQIGKDRFDTRLI |
| BA71V-CP2475L (P220) | BA71V-CP2475L (p220) | Core shell | 2403 | LIQIGKDRFDTRLIR |
| BA71V-CP2475L (P220) | BA71V-CP2475L (p220) | Core shell | 2404 | IQIGKDRFDTRLIRN |
| BA71V-CP2475L (P220) | BA71V-CP2475L (p220) | Core shell | 2405 | QIGKDRFDTRLIRNL |
| BA71V-CP2475L (P220) | BA71V-CP2475L (p220) | Core shell | 2406 | IGKDRFDTRLIRNLI |
| BA71V-CP2475L (P220) | BA71V-CP2475L (p220) | Core shell | 2407 | GKDRFDTRLIRNLIF |
| BA71V-CP2475L (P220) | BA71V-CP2475L (p220) | Core shell | 2408 | KDRFDTRLIRNLIFI |
| BA71V-CP2475L (P220) | BA71V-CP2475L (p220) | Core shell | 2409 | DRFDTRLIRNLIFIT |
| BA71V-CP2475L (P220) | BA71V-CP2475L (p220) | Core shell | 2410 | RFDTRLIRNLIFITN |
| BA71V-CP2475L (P220) | BA71V-CP2475L (p220) | Core shell | 2411 | FDTRLIRNLIFITNI |
| BA71V-CP2475L (P220) | BA71V-CP2475L (p220) | Core shell | 2412 | DTRLIRNLIFITNIQ |
| BA71V-CP2475L (P220) | BA71V-CP2475L (p220) | Core shell | 2413 | TRLIRNLIFITNIQR |
| BA71V-CP2475L (P220) | BA71V-CP2475L (p220) | Core shell | 2414 | RLIRNLIFITNIQRL |
| BA71V-CP2475L (P220) | BA71V-CP2475L (p220) | Core shell | 2415 | LIRNLIFITNIQRLL |
| BA71V-CP2475L (P220) | BA71V-CP2475L (p220) | Core shell | 2416 | IRNLIFITNIQRLLR |
| BA71V-CP2475L (P220) | BA71V-CP2475L (p220) | Core shell | 2417 | RNLIFITNIQRLLRL |
| BA71V-CP2475L (P220) | BA71V-CP2475L (p220) | Core shell | 2418 | NLIFITNIQRLLRLR |
| BA71V-CP2475L (P220) | BA71V-CP2475L (p220) | Core shell | 2419 | LIFITNIQRLLRLRL |
| BA71V-CP2475L (P220) | BA71V-CP2475L (p220) | Core shell | 2420 | IFITNIQRLLRLRLN |
| BA71V-CP2475L (P220) | BA71V-CP2475L (p220) | Core shell | 2421 | FITNIQRLLRLRLNL |
| BA71V-CP2475L (P220) | BA71V-CP2475L (p220) | Core shell | 2422 | ITNIQRLLRLRLNLE |
| BA71V-CP2475L (P220) | BA71V-CP2475L (p220) | Core shell | 2423 | TNIQRLLRLRLNLEL |
| BA71V-CP2475L (P220) | BA71V-CP2475L (p220) | Core shell | 2424 | NIQRLLRLRLNLELS |
| BA71V-CP2475L (P220) | BA71V-CP2475L (p220) | Core shell | 2425 | IQRLLRLRLNLELSQ |
| BA71V-CP2475L (P220) | BA71V-CP2475L (p220) | Core shell | 2426 | QRLLRLRLNLELSQF |
| BA71V-CP2475L (P220) | BA71V-CP2475L (p220) | Core shell | 2427 | RLLRLRLNLELSQFR |
| BA71V-CP2475L (P220) | BA71V-CP2475L (p220) | Core shell | 2428 | LLRLRLNLELSQFRN |
| BA71V-CP2475L (P220) | BA71V-CP2475L (p220) | Core shell | 2429 | LRLRLNLELSQFRNV |
| BA71V-CP2475L (P220) | BA71V-CP2475L (p220) | Core shell | 2430 | RLRLNLELSQFRNVL |

| | | | | |
|---|---|---|---|---|
| BA71V-CP2475L (P220) | BA71V-CP2475L (p220) | Core shell | 2431 | LRLNLELSQFRNVLV |
| BA71V-CP2475L (P220) | BA71V-CP2475L (p220) | Core shell | 2432 | RLNLELSQFRNVLVS |
| BA71V-CP2475L (P220) | BA71V-CP2475L (p220) | Core shell | 2433 | LNLELSQFRNVLVSP |
| BA71V-CP2475L (P220) | BA71V-CP2475L (p220) | Core shell | 2434 | NLELSQFRNVLVSPD |
| BA71V-CP2475L (P220) | BA71V-CP2475L (p220) | Core shell | 2435 | LELSQFRNVLVSPDH |
| BA71V-CP2475L (P220) | BA71V-CP2475L (p220) | Core shell | 2436 | ELSQFRNVLVSPDHI |
| BA71V-CP2475L (P220) | BA71V-CP2475L (p220) | Core shell | 2437 | LSQFRNVLVSPDHII |
| BA71V-CP2475L (P220) | BA71V-CP2475L (p220) | Core shell | 2438 | SQFRNVLVSPDHIIN |
| BA71V-CP2475L (P220) | BA71V-CP2475L (p220) | Core shell | 2439 | QFRNVLVSPDHIINP |
| BA71V-CP2475L (P220) | BA71V-CP2475L (p220) | Core shell | 2440 | FRNVLVSPDHIINPS |
| BA71V-CP2475L (P220) | BA71V-CP2475L (p220) | Core shell | 2441 | RNVLVSPDHIINPSI |
| BA71V-CP2475L (P220) | BA71V-CP2475L (p220) | Core shell | 2442 | NVLVSPDHIINPSIT |
| BA71V-CP2475L (P220) | BA71V-CP2475L (p220) | Core shell | 2443 | VLVSPDHIINPSITE |
| BA71V-CP2475L (P220) | BA71V-CP2475L (p220) | Core shell | 2444 | LVSPDHIINPSITEY |
| BA71V-CP2475L (P220) | BA71V-CP2475L (p220) | Core shell | 2445 | VSPDHIINPSITEYG |
| BA71V-CP2475L (P220) | BA71V-CP2475L (p220) | Core shell | 2446 | SPDHIINPSITEYGF |
| BA71V-CP2475L (P220) | BA71V-CP2475L (p220) | Core shell | 2447 | PDHIINPSITEYGFS |
| BA71V-CP2475L (P220) | BA71V-CP2475L (p220) | Core shell | 2448 | DHIINPSITEYGFSI |
| BA71V-CP2475L (P220) | BA71V-CP2475L (p220) | Core shell | 2449 | HIINPSITEYGFSIT |
| BA71V-CP2475L (P220) | BA71V-CP2475L (p220) | Core shell | 2450 | IINPSITEYGFSITG |
| BA71V-CP2475L (P220) | BA71V-CP2475L (p220) | Core shell | 2451 | INPSITEYGFSITGP |
| BA71V-CP2475L (P220) | BA71V-CP2475L (p220) | Core shell | 2452 | NPSITEYGFSITGPS |
| BA71V-CP2475L (P220) | BA71V-CP2475L (p220) | Core shell | 2453 | PSITEYGFSITGPSE |
| BA71V-CP2475L (P220) | BA71V-CP2475L (p220) | Core shell | 2454 | SITEYGFSITGPSET |
| BA71V-CP2475L (P220) | BA71V-CP2475L (p220) | Core shell | 2455 | ITEYGFSITGPSETF |
| BA71V-CP2475L (P220) | BA71V-CP2475L (p220) | Core shell | 2456 | TEYGFSITGPSETFS |
| BA71V-CP2475L (P220) | BA71V-CP2475L (p220) | Core shell | 2457 | EYGFSITGPSETFSD |
| BA71V-CP2475L (P220) | BA71V-CP2475L (p220) | Core shell | 2458 | YGFSITGPSETFSDK |
| BA71V-CP2475L (P220) | BA71V-CP2475L (p220) | Core shell | 2459 | GFSITGPSETFSDKQ |
| BA71V-CP2475L (P220) | BA71V-CP2475L (p220) | Core shell | 2460 | FSITGPSETFSDKQY |
| BA71V-CP2475L (P220) | BA71V-CP2475L (p220) | Core shell | 2461 | SITGPSETFSDKQYD |
| BA71V-CP2475L (P220) | BA71V-CP2475L (p220) | Core shell | 2462 | ITGPSETFSDKQYDS |
| BA71V-CP2475L (P220) | BA71V-CP2475L (p220) | Core shell | 2463 | TGPSETFSDKQYDSD |
| BA71V-CP2475L (P220) | BA71V-CP2475L (p220) | Core shell | 2464 | GPSETFSDKQYDSDI |
| BA71V-CP2475L (P220) | BA71V-CP2475L (p220) | Core shell | 2465 | PSETFSDKQYDSDIR |
| BA71V-CP2475L (P220) | BA71V-CP2475L (p220) | Core shell | 2466 | SETFSDKQYDSDIRI |
| BA71V-CP2475L (P220) | BA71V-CP2475L (p220) | Core shell | 2467 | ETFSDKQYDSDIRIL |
| BA71V-CP2475L (P220) | BA71V-CP2475L (p220) | Core shell | 2468 | TFSDKQYDSDIRILG |
| BA71V-CP2475L (P220) | BA71V-CP2475L (p220) | Core shell | 2469 | FSDKQYDSDIRILGS |
| BA71V-CP2475L (P220) | BA71V-CP2475L (p220) | Core shell | 2470 | SDKQYDSDIRILGSG |
| BA71V-CP2475L (P220) | BA71V-CP2475L (p220) | Core shell | 2471 | DKQYDSDIRILGSGS |
| BA71V-CP2475L (P220) | BA71V-CP2475L (p220) | Core shell | 2472 | KQYDSDIRILGSGSG |
| BA71V-CP2475L (P220) | BA71V-CP2475L (p220) | Core shell | 2473 | QYDSDIRILGSGSGS |
| BA71V-CP2475L (P220) | BA71V-CP2475L (p220) | Core shell | 2474 | YDSDIRILGSGSGSG |
| Polyprotein pp62 | Ba71V-94 | Core shell | 1 | GSGSGSGMPSNMKQF |
| Polyprotein pp62 | Ba71V-94 | Core shell | 2 | SGSGSGMPSNMKQFC |
| Polyprotein pp62 | Ba71V-94 | Core shell | 3 | GSGSGMPSNMKQFCK |
| Polyprotein pp62 | Ba71V-94 | Core shell | 4 | SGSGMPSNMKQFCKI |
| Polyprotein pp62 | Ba71V-94 | Core shell | 5 | GSGMPSNMKQFCKIS |
| Polyprotein pp62 | Ba71V-94 | Core shell | 6 | SGMPSNMKQFCKISV |
| Polyprotein pp62 | Ba71V-94 | Core shell | 7 | GMPSNMKQFCKISVW |
| Polyprotein pp62 | Ba71V-94 | Core shell | 8 | MPSNMKQFCKISVWL |
| Polyprotein pp62 | Ba71V-94 | Core shell | 9 | PSNMKQFCKISVWLQ |
| Polyprotein pp62 | Ba71V-94 | Core shell | 10 | SNMKQFCKISVWLQQ |
| Polyprotein pp62 | Ba71V-94 | Core shell | 11 | NMKQFCKISVWLQQH |
| Polyprotein pp62 | Ba71V-94 | Core shell | 12 | MKQFCKISVWLQQHD |
| Polyprotein pp62 | Ba71V-94 | Core shell | 13 | KQFCKISVWLQQHDP |
| Polyprotein pp62 | Ba71V-94 | Core shell | 14 | QFCKISVWLQQHDPD |
| Polyprotein pp62 | Ba71V-94 | Core shell | 15 | FCKISVWLQQHDPDL |
| Polyprotein pp62 | Ba71V-94 | Core shell | 16 | CKISVWLQQHDPDLL |
| Polyprotein pp62 | Ba71V-94 | Core shell | 17 | KISVWLQQHDPDLLE |
| Polyprotein pp62 | Ba71V-94 | Core shell | 18 | ISVWLQQHDPDLLEI |
| Polyprotein pp62 | Ba71V-94 | Core shell | 19 | SVWLQQHDPDLLEII |
| Polyprotein pp62 | Ba71V-94 | Core shell | 20 | VWLQQHDPDLLEIIN |
| Polyprotein pp62 | Ba71V-94 | Core shell | 21 | WLQQHDPDLLEIINN |
| Polyprotein pp62 | Ba71V-94 | Core shell | 22 | LQQHDPDLLEIINNL |
| Polyprotein pp62 | Ba71V-94 | Core shell | 23 | QQHDPDLLEIINNLC |

| | | | | |
|---|---|---|---|---|
| Polyprotein pp62 | Ba71V-94 | Core shell | 24 | QHDPDLLEIINNLCM |
| Polyprotein pp62 | Ba71V-94 | Core shell | 25 | HDPDLLEIINNLCML |
| Polyprotein pp62 | Ba71V-94 | Core shell | 26 | DPDLLEIINNLCMLG |
| Polyprotein pp62 | Ba71V-94 | Core shell | 27 | PDLLEIINNLCMLGN |
| Polyprotein pp62 | Ba71V-94 | Core shell | 28 | DLLEIINNLCMLGNL |
| Polyprotein pp62 | Ba71V-94 | Core shell | 29 | LLEIINNLCMLGNLS |
| Polyprotein pp62 | Ba71V-94 | Core shell | 30 | LEIINNLCMLGNLSA |
| Polyprotein pp62 | Ba71V-94 | Core shell | 31 | EIINNLCMLGNLSAA |
| Polyprotein pp62 | Ba71V-94 | Core shell | 32 | IINNLCMLGNLSAAK |
| Polyprotein pp62 | Ba71V-94 | Core shell | 33 | INNLCMLGNLSAAKY |
| Polyprotein pp62 | Ba71V-94 | Core shell | 34 | NNLCMLGNLSAAKYK |
| Polyprotein pp62 | Ba71V-94 | Core shell | 35 | NLCMLGNLSAAKYKH |
| Polyprotein pp62 | Ba71V-94 | Core shell | 36 | LCMLGNLSAAKYKHG |
| Polyprotein pp62 | Ba71V-94 | Core shell | 37 | CMLGNLSAAKYKHGV |
| Polyprotein pp62 | Ba71V-94 | Core shell | 38 | MLGNLSAAKYKHGVT |
| Polyprotein pp62 | Ba71V-94 | Core shell | 39 | LGNLSAAKYKHGVTF |
| Polyprotein pp62 | Ba71V-94 | Core shell | 40 | GNLSAAKYKHGVTFI |
| Polyprotein pp62 | Ba71V-94 | Core shell | 41 | NLSAAKYKHGVTFIY |
| Polyprotein pp62 | Ba71V-94 | Core shell | 42 | LSAAKYKHGVTFIYP |
| Polyprotein pp62 | Ba71V-94 | Core shell | 43 | SAAKYKHGVTFIYPK |
| Polyprotein pp62 | Ba71V-94 | Core shell | 44 | AAKYKHGVTFIYPKQ |
| Polyprotein pp62 | Ba71V-94 | Core shell | 45 | AKYKHGVTFIYPKQA |
| Polyprotein pp62 | Ba71V-94 | Core shell | 46 | KYKHGVTFIYPKQAK |
| Polyprotein pp62 | Ba71V-94 | Core shell | 47 | YKHGVTFIYPKQAKI |
| Polyprotein pp62 | Ba71V-94 | Core shell | 48 | KHGVTFIYPKQAKIR |
| Polyprotein pp62 | Ba71V-94 | Core shell | 49 | HGVTFIYPKQAKIRD |
| Polyprotein pp62 | Ba71V-94 | Core shell | 50 | GVTFIYPKQAKIRDE |
| Polyprotein pp62 | Ba71V-94 | Core shell | 51 | VTFIYPKQAKIRDEI |
| Polyprotein pp62 | Ba71V-94 | Core shell | 52 | TFIYPKQAKIRDEIK |
| Polyprotein pp62 | Ba71V-94 | Core shell | 53 | FIYPKQAKIRDEIKK |
| Polyprotein pp62 | Ba71V-94 | Core shell | 54 | IYPKQAKIRDEIKKH |
| Polyprotein pp62 | Ba71V-94 | Core shell | 55 | YPKQAKIRDEIKKHA |
| Polyprotein pp62 | Ba71V-94 | Core shell | 56 | PKQAKIRDEIKKHAY |
| Polyprotein pp62 | Ba71V-94 | Core shell | 57 | KQAKIRDEIKKHAYS |
| Polyprotein pp62 | Ba71V-94 | Core shell | 58 | QAKIRDEIKKHAYSN |
| Polyprotein pp62 | Ba71V-94 | Core shell | 59 | AKIRDEIKKHAYSND |
| Polyprotein pp62 | Ba71V-94 | Core shell | 60 | KIRDEIKKHAYSNDP |
| Polyprotein pp62 | Ba71V-94 | Core shell | 61 | IRDEIKKHAYSNDPS |
| Polyprotein pp62 | Ba71V-94 | Core shell | 62 | RDEIKKHAYSNDPSQ |
| Polyprotein pp62 | Ba71V-94 | Core shell | 63 | DEIKKHAYSNDPSQA |
| Polyprotein pp62 | Ba71V-94 | Core shell | 64 | EIKKHAYSNDPSQAI |
| Polyprotein pp62 | Ba71V-94 | Core shell | 65 | IKKHAYSNDPSQAIK |
| Polyprotein pp62 | Ba71V-94 | Core shell | 66 | KKHAYSNDPSQAIKT |
| Polyprotein pp62 | Ba71V-94 | Core shell | 67 | KHAYSNDPSQAIKTL |
| Polyprotein pp62 | Ba71V-94 | Core shell | 68 | HAYSNDPSQAIKTLE |
| Polyprotein pp62 | Ba71V-94 | Core shell | 69 | AYSNDPSQAIKTLES |
| Polyprotein pp62 | Ba71V-94 | Core shell | 70 | YSNDPSQAIKTLESL |
| Polyprotein pp62 | Ba71V-94 | Core shell | 71 | SNDPSQAIKTLESLI |
| Polyprotein pp62 | Ba71V-94 | Core shell | 72 | NDPSQAIKTLESLIL |
| Polyprotein pp62 | Ba71V-94 | Core shell | 73 | DPSQAIKTLESLILP |
| Polyprotein pp62 | Ba71V-94 | Core shell | 74 | PSQAIKTLESLILPF |
| Polyprotein pp62 | Ba71V-94 | Core shell | 75 | SQAIKTLESLILPFY |
| Polyprotein pp62 | Ba71V-94 | Core shell | 76 | QAIKTLESLILPFYI |
| Polyprotein pp62 | Ba71V-94 | Core shell | 77 | AIKTLESLILPFYIP |
| Polyprotein pp62 | Ba71V-94 | Core shell | 78 | IKTLESLILPFYIPT |
| Polyprotein pp62 | Ba71V-94 | Core shell | 79 | KTLESLILPFYIPTP |
| Polyprotein pp62 | Ba71V-94 | Core shell | 80 | TLESLILPFYIPTPA |
| Polyprotein pp62 | Ba71V-94 | Core shell | 81 | LESLILPFYIPTPAE |
| Polyprotein pp62 | Ba71V-94 | Core shell | 82 | ESLILPFYIPTPAEF |
| Polyprotein pp62 | Ba71V-94 | Core shell | 83 | SLILPFYIPTPAEFT |
| Polyprotein pp62 | Ba71V-94 | Core shell | 84 | LILPFYIPTPAEFTG |
| Polyprotein pp62 | Ba71V-94 | Core shell | 85 | ILPFYIPTPAEFTGE |
| Polyprotein pp62 | Ba71V-94 | Core shell | 86 | LPFYIPTPAEFTGEI |
| Polyprotein pp62 | Ba71V-94 | Core shell | 87 | PFYIPTPAEFTGEIG |
| Polyprotein pp62 | Ba71V-94 | Core shell | 88 | FYIPTPAEFTGEIGS |
| Polyprotein pp62 | Ba71V-94 | Core shell | 89 | YIPTPAEFTGEIGSY |
| Polyprotein pp62 | Ba71V-94 | Core shell | 90 | IPTPAEFTGEIGSYT |

| | | | | |
|---|---|---|---|---|
| Polyprotein pp62 | Ba71V-94 | Core shell | 91 | PTPAEFTGEIGSYTG |
| Polyprotein pp62 | Ba71V-94 | Core shell | 92 | TPAEFTGEIGSYTGV |
| Polyprotein pp62 | Ba71V-94 | Core shell | 93 | PAEFTGEIGSYTGVK |
| Polyprotein pp62 | Ba71V-94 | Core shell | 94 | AEFTGEIGSYTGVKL |
| Polyprotein pp62 | Ba71V-94 | Core shell | 95 | EFTGEIGSYTGVKLE |
| Polyprotein pp62 | Ba71V-94 | Core shell | 96 | FTGEIGSYTGVKLEV |
| Polyprotein pp62 | Ba71V-94 | Core shell | 97 | TGEIGSYTGVKLEVE |
| Polyprotein pp62 | Ba71V-94 | Core shell | 98 | GEIGSYTGVKLEVEK |
| Polyprotein pp62 | Ba71V-94 | Core shell | 99 | EIGSYTGVKLEVEKT |
| Polyprotein pp62 | Ba71V-94 | Core shell | 100 | IGSYTGVKLEVEKTE |
| Polyprotein pp62 | Ba71V-94 | Core shell | 101 | GSYTGVKLEVEKTEA |
| Polyprotein pp62 | Ba71V-94 | Core shell | 102 | SYTGVKLEVEKTEAN |
| Polyprotein pp62 | Ba71V-94 | Core shell | 103 | YTGVKLEVEKTEANK |
| Polyprotein pp62 | Ba71V-94 | Core shell | 104 | TGVKLEVEKTEANKV |
| Polyprotein pp62 | Ba71V-94 | Core shell | 105 | GVKLEVEKTEANKVI |
| Polyprotein pp62 | Ba71V-94 | Core shell | 106 | VKLEVEKTEANKVIL |
| Polyprotein pp62 | Ba71V-94 | Core shell | 107 | KLEVEKTEANKVILK |
| Polyprotein pp62 | Ba71V-94 | Core shell | 108 | LEVEKTEANKVILKN |
| Polyprotein pp62 | Ba71V-94 | Core shell | 109 | EVEKTEANKVILKNG |
| Polyprotein pp62 | Ba71V-94 | Core shell | 110 | VEKTEANKVILKNGE |
| Polyprotein pp62 | Ba71V-94 | Core shell | 111 | EKTEANKVILKNGEA |
| Polyprotein pp62 | Ba71V-94 | Core shell | 112 | KTEANKVILKNGEAV |
| Polyprotein pp62 | Ba71V-94 | Core shell | 113 | TEANKVILKNGEAVL |
| Polyprotein pp62 | Ba71V-94 | Core shell | 114 | EANKVILKNGEAVLV |
| Polyprotein pp62 | Ba71V-94 | Core shell | 115 | ANKVILKNGEAVLVP |
| Polyprotein pp62 | Ba71V-94 | Core shell | 116 | NKVILKNGEAVLVPA |
| Polyprotein pp62 | Ba71V-94 | Core shell | 117 | KVILKNGEAVLVPAA |
| Polyprotein pp62 | Ba71V-94 | Core shell | 118 | VILKNGEAVLVPAAD |
| Polyprotein pp62 | Ba71V-94 | Core shell | 119 | ILKNGEAVLVPAADF |
| Polyprotein pp62 | Ba71V-94 | Core shell | 120 | LKNGEAVLVPAADFK |
| Polyprotein pp62 | Ba71V-94 | Core shell | 121 | KNGEAVLVPAADFKP |
| Polyprotein pp62 | Ba71V-94 | Core shell | 122 | NGEAVLVPAADFKPF |
| Polyprotein pp62 | Ba71V-94 | Core shell | 123 | GEAVLVPAADFKPFP |
| Polyprotein pp62 | Ba71V-94 | Core shell | 124 | EAVLVPAADFKPFPD |
| Polyprotein pp62 | Ba71V-94 | Core shell | 125 | AVLVPAADFKPFPDR |
| Polyprotein pp62 | Ba71V-94 | Core shell | 126 | VLVPAADFKPFPDRR |
| Polyprotein pp62 | Ba71V-94 | Core shell | 127 | LVPAADFKPFPDRRL |
| Polyprotein pp62 | Ba71V-94 | Core shell | 128 | VPAADFKPFPDRRLA |
| Polyprotein pp62 | Ba71V-94 | Core shell | 129 | PAADFKPFPDRRLAV |
| Polyprotein pp62 | Ba71V-94 | Core shell | 130 | AADFKPFPDRRLAVW |
| Polyprotein pp62 | Ba71V-94 | Core shell | 131 | ADFKPFPDRRLAVWI |
| Polyprotein pp62 | Ba71V-94 | Core shell | 132 | DFKPFPDRRLAVWIM |
| Polyprotein pp62 | Ba71V-94 | Core shell | 133 | FKPFPDRRLAVWIME |
| Polyprotein pp62 | Ba71V-94 | Core shell | 134 | KPFPDRRLAVWIMES |
| Polyprotein pp62 | Ba71V-94 | Core shell | 135 | PFPDRRLAVWIMESG |
| Polyprotein pp62 | Ba71V-94 | Core shell | 136 | FPDRRLAVWIMESGS |
| Polyprotein pp62 | Ba71V-94 | Core shell | 137 | PDRRLAVWIMESGSM |
| Polyprotein pp62 | Ba71V-94 | Core shell | 138 | DRRLAVWIMESGSMP |
| Polyprotein pp62 | Ba71V-94 | Core shell | 139 | RRLAVWIMESGSMPL |
| Polyprotein pp62 | Ba71V-94 | Core shell | 140 | RLAVWIMESGSMPLE |
| Polyprotein pp62 | Ba71V-94 | Core shell | 141 | LAVWIMESGSMPLEG |
| Polyprotein pp62 | Ba71V-94 | Core shell | 142 | AVWIMESGSMPLEGP |
| Polyprotein pp62 | Ba71V-94 | Core shell | 143 | VWIMESGSMPLEGPP |
| Polyprotein pp62 | Ba71V-94 | Core shell | 144 | WIMESGSMPLEGPPY |
| Polyprotein pp62 | Ba71V-94 | Core shell | 145 | IMESGSMPLEGPPYK |
| Polyprotein pp62 | Ba71V-94 | Core shell | 146 | MESGSMPLEGPPYKR |
| Polyprotein pp62 | Ba71V-94 | Core shell | 147 | ESGSMPLEGPPYKRK |
| Polyprotein pp62 | Ba71V-94 | Core shell | 148 | SGSMPLEGPPYKRKK |
| Polyprotein pp62 | Ba71V-94 | Core shell | 149 | GSMPLEGPPYKRKKE |
| Polyprotein pp62 | Ba71V-94 | Core shell | 150 | SMPLEGPPYKRKKEG |
| Polyprotein pp62 | Ba71V-94 | Core shell | 151 | MPLEGPPYKRKKEGG |
| Polyprotein pp62 | Ba71V-94 | Core shell | 152 | PLEGPPYKRKKEGGG |
| Polyprotein pp62 | Ba71V-94 | Core shell | 153 | LEGPPYKRKKEGGGN |
| Polyprotein pp62 | Ba71V-94 | Core shell | 154 | EGPPYKRKKEGGGND |
| Polyprotein pp62 | Ba71V-94 | Core shell | 155 | GPPYKRKKEGGGNDP |
| Polyprotein pp62 | Ba71V-94 | Core shell | 156 | PPYKRKKEGGGNDPP |
| Polyprotein pp62 | Ba71V-94 | Core shell | 157 | PYKRKKEGGGNDPPV |

| | | | |
|---|---|---|---|
| Polyprotein pp62 | Ba71V-94 | Core shell | 158 YKRKKEGGGNDPPVP |
| Polyprotein pp62 | Ba71V-94 | Core shell | 159 KRKKEGGGNDPPVPK |
| Polyprotein pp62 | Ba71V-94 | Core shell | 160 RKKEGGGNDPPVPKH |
| Polyprotein pp62 | Ba71V-94 | Core shell | 161 KKEGGGNDPPVPKHI |
| Polyprotein pp62 | Ba71V-94 | Core shell | 162 KEGGGNDPPVPKHIS |
| Polyprotein pp62 | Ba71V-94 | Core shell | 163 EGGGNDPPVPKHISP |
| Polyprotein pp62 | Ba71V-94 | Core shell | 164 GGGNDPPVPKHISPY |
| Polyprotein pp62 | Ba71V-94 | Core shell | 165 GGNDPPVPKHISPYT |
| Polyprotein pp62 | Ba71V-94 | Core shell | 166 GNDPPVPKHISPYTP |
| Polyprotein pp62 | Ba71V-94 | Core shell | 167 NDPPVPKHISPYTPR |
| Polyprotein pp62 | Ba71V-94 | Core shell | 168 DPPVPKHISPYTPRT |
| Polyprotein pp62 | Ba71V-94 | Core shell | 169 PPVPKHISPYTPRTR |
| Polyprotein pp62 | Ba71V-94 | Core shell | 170 PVPKHISPYTPRTRI |
| Polyprotein pp62 | Ba71V-94 | Core shell | 171 VPKHISPYTPRTRIA |
| Polyprotein pp62 | Ba71V-94 | Core shell | 172 PKHISPYTPRTRIAI |
| Polyprotein pp62 | Ba71V-94 | Core shell | 173 KHISPYTPRTRIAIE |
| Polyprotein pp62 | Ba71V-94 | Core shell | 174 HISPYTPRTRIAIEV |
| Polyprotein pp62 | Ba71V-94 | Core shell | 175 ISPYTPRTRIAIEVE |
| Polyprotein pp62 | Ba71V-94 | Core shell | 176 SPYTPRTRIAIEVEK |
| Polyprotein pp62 | Ba71V-94 | Core shell | 177 PYTPRTRIAIEVEKA |
| Polyprotein pp62 | Ba71V-94 | Core shell | 178 YTPRTRIAIEVEKAF |
| Polyprotein pp62 | Ba71V-94 | Core shell | 179 TPRTRIAIEVEKAFD |
| Polyprotein pp62 | Ba71V-94 | Core shell | 180 PRTRIAIEVEKAFDD |
| Polyprotein pp62 | Ba71V-94 | Core shell | 181 RTRIAIEVEKAFDDC |
| Polyprotein pp62 | Ba71V-94 | Core shell | 182 TRIAIEVEKAFDDCM |
| Polyprotein pp62 | Ba71V-94 | Core shell | 183 RIAIEVEKAFDDCMR |
| Polyprotein pp62 | Ba71V-94 | Core shell | 184 IAIEVEKAFDDCMRQ |
| Polyprotein pp62 | Ba71V-94 | Core shell | 185 AIEVEKAFDDCMRQN |
| Polyprotein pp62 | Ba71V-94 | Core shell | 186 IEVEKAFDDCMRQNW |
| Polyprotein pp62 | Ba71V-94 | Core shell | 187 EVEKAFDDCMRQNWC |
| Polyprotein pp62 | Ba71V-94 | Core shell | 188 VEKAFDDCMRQNWCS |
| Polyprotein pp62 | Ba71V-94 | Core shell | 189 EKAFDDCMRQNWCSV |
| Polyprotein pp62 | Ba71V-94 | Core shell | 190 KAFDDCMRQNWCSVN |
| Polyprotein pp62 | Ba71V-94 | Core shell | 191 AFDDCMRQNWCSVNN |
| Polyprotein pp62 | Ba71V-94 | Core shell | 192 FDDCMRQNWCSVNNP |
| Polyprotein pp62 | Ba71V-94 | Core shell | 193 DDCMRQNWCSVNNPY |
| Polyprotein pp62 | Ba71V-94 | Core shell | 194 DCMRQNWCSVNNPYL |
| Polyprotein pp62 | Ba71V-94 | Core shell | 195 CMRQNWCSVNNPYLA |
| Polyprotein pp62 | Ba71V-94 | Core shell | 196 MRQNWCSVNNPYLAK |
| Polyprotein pp62 | Ba71V-94 | Core shell | 197 RQNWCSVNNPYLAKS |
| Polyprotein pp62 | Ba71V-94 | Core shell | 198 QNWCSVNNPYLAKSV |
| Polyprotein pp62 | Ba71V-94 | Core shell | 199 NWCSVNNPYLAKSVS |
| Polyprotein pp62 | Ba71V-94 | Core shell | 200 WCSVNNPYLAKSVSL |
| Polyprotein pp62 | Ba71V-94 | Core shell | 201 CSVNNPYLAKSVSLL |
| Polyprotein pp62 | Ba71V-94 | Core shell | 202 SVNNPYLAKSVSLLS |
| Polyprotein pp62 | Ba71V-94 | Core shell | 203 VNNPYLAKSVSLLSF |
| Polyprotein pp62 | Ba71V-94 | Core shell | 204 NNPYLAKSVSLLSFL |
| Polyprotein pp62 | Ba71V-94 | Core shell | 205 NPYLAKSVSLLSFLS |
| Polyprotein pp62 | Ba71V-94 | Core shell | 206 PYLAKSVSLLSFLSL |
| Polyprotein pp62 | Ba71V-94 | Core shell | 207 YLAKSVSLLSFLSLN |
| Polyprotein pp62 | Ba71V-94 | Core shell | 208 LAKSVSLLSFLSLNH |
| Polyprotein pp62 | Ba71V-94 | Core shell | 209 AKSVSLLSFLSLNHP |
| Polyprotein pp62 | Ba71V-94 | Core shell | 210 KSVSLLSFLSLNHPT |
| Polyprotein pp62 | Ba71V-94 | Core shell | 211 SVSLLSFLSLNHPTE |
| Polyprotein pp62 | Ba71V-94 | Core shell | 212 VSLLSFLSLNHPTEF |
| Polyprotein pp62 | Ba71V-94 | Core shell | 213 SLLSFLSLNHPTEFI |
| Polyprotein pp62 | Ba71V-94 | Core shell | 214 LLSFLSLNHPTEFIK |
| Polyprotein pp62 | Ba71V-94 | Core shell | 215 LSFLSLNHPTEFIKV |
| Polyprotein pp62 | Ba71V-94 | Core shell | 216 SFLSLNHPTEFIKVL |
| Polyprotein pp62 | Ba71V-94 | Core shell | 217 FLSLNHPTEFIKVLP |
| Polyprotein pp62 | Ba71V-94 | Core shell | 218 LSLNHPTEFIKVLPL |
| Polyprotein pp62 | Ba71V-94 | Core shell | 219 SLNHPTEFIKVLPLI |
| Polyprotein pp62 | Ba71V-94 | Core shell | 220 LNHPTEFIKVLPLID |
| Polyprotein pp62 | Ba71V-94 | Core shell | 221 NHPTEFIKVLPLIDF |
| Polyprotein pp62 | Ba71V-94 | Core shell | 222 HPTEFIKVLPLIDFD |
| Polyprotein pp62 | Ba71V-94 | Core shell | 223 PTEFIKVLPLIDFDP |
| Polyprotein pp62 | Ba71V-94 | Core shell | 224 TEFIKVLPLIDFDPL |

| | | | | |
|---|---|---|---|---|
| Polyprotein pp62 | Ba71V-94 | Core shell | 225 | EFIKVLPLIDFDPLV |
| Polyprotein pp62 | Ba71V-94 | Core shell | 226 | FIKVLPLIDFDPLVT |
| Polyprotein pp62 | Ba71V-94 | Core shell | 227 | IKVLPLIDFDPLVTF |
| Polyprotein pp62 | Ba71V-94 | Core shell | 228 | KVLPLIDFDPLVTFY |
| Polyprotein pp62 | Ba71V-94 | Core shell | 229 | VLPLIDFDPLVTFYL |
| Polyprotein pp62 | Ba71V-94 | Core shell | 230 | LPLIDFDPLVTFYLL |
| Polyprotein pp62 | Ba71V-94 | Core shell | 231 | PLIDFDPLVTFYLLL |
| Polyprotein pp62 | Ba71V-94 | Core shell | 232 | LIDFDPLVTFYLLLE |
| Polyprotein pp62 | Ba71V-94 | Core shell | 233 | IDFDPLVTFYLLLEP |
| Polyprotein pp62 | Ba71V-94 | Core shell | 234 | DFDPLVTFYLLLEPY |
| Polyprotein pp62 | Ba71V-94 | Core shell | 235 | FDPLVTFYLLLEPYK |
| Polyprotein pp62 | Ba71V-94 | Core shell | 236 | DPLVTFYLLLEPYKT |
| Polyprotein pp62 | Ba71V-94 | Core shell | 237 | PLVTFYLLLEPYKTH |
| Polyprotein pp62 | Ba71V-94 | Core shell | 238 | LVTFYLLLEPYKTHG |
| Polyprotein pp62 | Ba71V-94 | Core shell | 239 | VTFYLLLEPYKTHGD |
| Polyprotein pp62 | Ba71V-94 | Core shell | 240 | TFYLLLEPYKTHGDD |
| Polyprotein pp62 | Ba71V-94 | Core shell | 241 | FYLLLEPYKTHGDDF |
| Polyprotein pp62 | Ba71V-94 | Core shell | 242 | YLLLEPYKTHGDDFL |
| Polyprotein pp62 | Ba71V-94 | Core shell | 243 | LLLEPYKTHGDDFLI |
| Polyprotein pp62 | Ba71V-94 | Core shell | 244 | LLEPYKTHGDDFLIP |
| Polyprotein pp62 | Ba71V-94 | Core shell | 245 | LEPYKTHGDDFLIPE |
| Polyprotein pp62 | Ba71V-94 | Core shell | 246 | EPYKTHGDDFLIPET |
| Polyprotein pp62 | Ba71V-94 | Core shell | 247 | PYKTHGDDFLIPETI |
| Polyprotein pp62 | Ba71V-94 | Core shell | 248 | YKTHGDDFLIPETIL |
| Polyprotein pp62 | Ba71V-94 | Core shell | 249 | KTHGDDFLIPETILF |
| Polyprotein pp62 | Ba71V-94 | Core shell | 250 | THGDDFLIPETILFG |
| Polyprotein pp62 | Ba71V-94 | Core shell | 251 | HGDDFLIPETILFGP |
| Polyprotein pp62 | Ba71V-94 | Core shell | 252 | GDDFLIPETILFGPT |
| Polyprotein pp62 | Ba71V-94 | Core shell | 253 | DDFLIPETILFGPTG |
| Polyprotein pp62 | Ba71V-94 | Core shell | 254 | DFLIPETILFGPTGW |
| Polyprotein pp62 | Ba71V-94 | Core shell | 255 | FLIPETILFGPTGWN |
| Polyprotein pp62 | Ba71V-94 | Core shell | 256 | LIPETILFGPTGWNG |
| Polyprotein pp62 | Ba71V-94 | Core shell | 257 | IPETILFGPTGWNGT |
| Polyprotein pp62 | Ba71V-94 | Core shell | 258 | PETILFGPTGWNGTD |
| Polyprotein pp62 | Ba71V-94 | Core shell | 259 | ETILFGPTGWNGTDL |
| Polyprotein pp62 | Ba71V-94 | Core shell | 260 | TILFGPTGWNGTDLY |
| Polyprotein pp62 | Ba71V-94 | Core shell | 261 | ILFGPTGWNGTDLYQ |
| Polyprotein pp62 | Ba71V-94 | Core shell | 262 | LFGPTGWNGTDLYQS |
| Polyprotein pp62 | Ba71V-94 | Core shell | 263 | FGPTGWNGTDLYQSA |
| Polyprotein pp62 | Ba71V-94 | Core shell | 264 | GPTGWNGTDLYQSAM |
| Polyprotein pp62 | Ba71V-94 | Core shell | 265 | PTGWNGTDLYQSAML |
| Polyprotein pp62 | Ba71V-94 | Core shell | 266 | TGWNGTDLYQSAMLE |
| Polyprotein pp62 | Ba71V-94 | Core shell | 267 | GWNGTDLYQSAMLEF |
| Polyprotein pp62 | Ba71V-94 | Core shell | 268 | WNGTDLYQSAMLEFK |
| Polyprotein pp62 | Ba71V-94 | Core shell | 269 | NGTDLYQSAMLEFKK |
| Polyprotein pp62 | Ba71V-94 | Core shell | 270 | GTDLYQSAMLEFKKF |
| Polyprotein pp62 | Ba71V-94 | Core shell | 271 | TDLYQSAMLEFKKFF |
| Polyprotein pp62 | Ba71V-94 | Core shell | 272 | DLYQSAMLEFKKFFT |
| Polyprotein pp62 | Ba71V-94 | Core shell | 273 | LYQSAMLEFKKFFTQ |
| Polyprotein pp62 | Ba71V-94 | Core shell | 274 | YQSAMLEFKKFFTQI |
| Polyprotein pp62 | Ba71V-94 | Core shell | 275 | QSAMLEFKKFFTQIT |
| Polyprotein pp62 | Ba71V-94 | Core shell | 276 | SAMLEFKKFFTQITR |
| Polyprotein pp62 | Ba71V-94 | Core shell | 277 | AMLEFKKFFTQITRQ |
| Polyprotein pp62 | Ba71V-94 | Core shell | 278 | MLEFKKFFTQITRQT |
| Polyprotein pp62 | Ba71V-94 | Core shell | 279 | LEFKKFFTQITRQTF |
| Polyprotein pp62 | Ba71V-94 | Core shell | 280 | EFKKFFTQITRQTFM |
| Polyprotein pp62 | Ba71V-94 | Core shell | 281 | FKKFFTQITRQTFMD |
| Polyprotein pp62 | Ba71V-94 | Core shell | 282 | KKFFTQITRQTFMDI |
| Polyprotein pp62 | Ba71V-94 | Core shell | 283 | KFFTQITRQTFMDIA |
| Polyprotein pp62 | Ba71V-94 | Core shell | 284 | FFTQITRQTFMDIAD |
| Polyprotein pp62 | Ba71V-94 | Core shell | 285 | FTQITRQTFMDIADS |
| Polyprotein pp62 | Ba71V-94 | Core shell | 286 | TQITRQTFMDIADSA |
| Polyprotein pp62 | Ba71V-94 | Core shell | 287 | QITRQTFMDIADSAT |
| Polyprotein pp62 | Ba71V-94 | Core shell | 288 | ITRQTFMDIADSATK |
| Polyprotein pp62 | Ba71V-94 | Core shell | 289 | TRQTFMDIADSATKE |
| Polyprotein pp62 | Ba71V-94 | Core shell | 290 | RQTFMDIADSATKEV |
| Polyprotein pp62 | Ba71V-94 | Core shell | 291 | QTFMDIADSATKEVD |

| | | | | |
|---|---|---|---|---|
| Polyprotein pp62 | Ba71V-94 | Core shell | 292 | TFMDIADSATKEVDV |
| Polyprotein pp62 | Ba71V-94 | Core shell | 293 | FMDIADSATKEVDVP |
| Polyprotein pp62 | Ba71V-94 | Core shell | 294 | MDIADSATKEVDVPI |
| Polyprotein pp62 | Ba71V-94 | Core shell | 295 | DIADSATKEVDVPIC |
| Polyprotein pp62 | Ba71V-94 | Core shell | 296 | IADSATKEVDVPICY |
| Polyprotein pp62 | Ba71V-94 | Core shell | 297 | ADSATKEVDVPICYS |
| Polyprotein pp62 | Ba71V-94 | Core shell | 298 | DSATKEVDVPICYSD |
| Polyprotein pp62 | Ba71V-94 | Core shell | 299 | SATKEVDVPICYSDP |
| Polyprotein pp62 | Ba71V-94 | Core shell | 300 | ATKEVDVPICYSDPE |
| Polyprotein pp62 | Ba71V-94 | Core shell | 301 | TKEVDVPICYSDPET |
| Polyprotein pp62 | Ba71V-94 | Core shell | 302 | KEVDVPICYSDPETV |
| Polyprotein pp62 | Ba71V-94 | Core shell | 303 | EVDVPICYSDPETVH |
| Polyprotein pp62 | Ba71V-94 | Core shell | 304 | VDVPICYSDPETVHS |
| Polyprotein pp62 | Ba71V-94 | Core shell | 305 | DVPICYSDPETVHSY |
| Polyprotein pp62 | Ba71V-94 | Core shell | 306 | VPICYSDPETVHSYT |
| Polyprotein pp62 | Ba71V-94 | Core shell | 307 | PICYSDPETVHSYTN |
| Polyprotein pp62 | Ba71V-94 | Core shell | 308 | ICYSDPETVHSYTNH |
| Polyprotein pp62 | Ba71V-94 | Core shell | 309 | CYSDPETVHSYTNHV |
| Polyprotein pp62 | Ba71V-94 | Core shell | 310 | YSDPETVHSYTNHVR |
| Polyprotein pp62 | Ba71V-94 | Core shell | 311 | SDPETVHSYTNHVRT |
| Polyprotein pp62 | Ba71V-94 | Core shell | 312 | DPETVHSYTNHVRTE |
| Polyprotein pp62 | Ba71V-94 | Core shell | 313 | PETVHSYTNHVRTEI |
| Polyprotein pp62 | Ba71V-94 | Core shell | 314 | ETVHSYTNHVRTEIL |
| Polyprotein pp62 | Ba71V-94 | Core shell | 315 | TVHSYTNHVRTEILH |
| Polyprotein pp62 | Ba71V-94 | Core shell | 316 | VHSYTNHVRTEILHH |
| Polyprotein pp62 | Ba71V-94 | Core shell | 317 | HSYTNHVRTEILHHN |
| Polyprotein pp62 | Ba71V-94 | Core shell | 318 | SYTNHVRTEILHHNA |
| Polyprotein pp62 | Ba71V-94 | Core shell | 319 | YTNHVRTEILHHNAV |
| Polyprotein pp62 | Ba71V-94 | Core shell | 320 | TNHVRTEILHHNAVN |
| Polyprotein pp62 | Ba71V-94 | Core shell | 321 | NHVRTEILHHNAVNK |
| Polyprotein pp62 | Ba71V-94 | Core shell | 322 | HVRTEILHHNAVNKV |
| Polyprotein pp62 | Ba71V-94 | Core shell | 323 | VRTEILHHNAVNKVT |
| Polyprotein pp62 | Ba71V-94 | Core shell | 324 | RTEILHHNAVNKVTT |
| Polyprotein pp62 | Ba71V-94 | Core shell | 325 | TEILHHNAVNKVTTP |
| Polyprotein pp62 | Ba71V-94 | Core shell | 326 | EILHHNAVNKVTTPN |
| Polyprotein pp62 | Ba71V-94 | Core shell | 327 | ILHHNAVNKVTTPNL |
| Polyprotein pp62 | Ba71V-94 | Core shell | 328 | LHHNAVNKVTTPNLV |
| Polyprotein pp62 | Ba71V-94 | Core shell | 329 | HHNAVNKVTTPNLVV |
| Polyprotein pp62 | Ba71V-94 | Core shell | 330 | HNAVNKVTTPNLVVQ |
| Polyprotein pp62 | Ba71V-94 | Core shell | 331 | NAVNKVTTPNLVVQA |
| Polyprotein pp62 | Ba71V-94 | Core shell | 332 | AVNKVTTPNLVVQAY |
| Polyprotein pp62 | Ba71V-94 | Core shell | 333 | VNKVTTPNLVVQAYN |
| Polyprotein pp62 | Ba71V-94 | Core shell | 334 | NKVTTPNLVVQAYNE |
| Polyprotein pp62 | Ba71V-94 | Core shell | 335 | KVTTPNLVVQAYNEL |
| Polyprotein pp62 | Ba71V-94 | Core shell | 336 | VTTPNLVVQAYNELE |
| Polyprotein pp62 | Ba71V-94 | Core shell | 337 | TTPNLVVQAYNELEQ |
| Polyprotein pp62 | Ba71V-94 | Core shell | 338 | TPNLVVQAYNELEQT |
| Polyprotein pp62 | Ba71V-94 | Core shell | 339 | PNLVVQAYNELEQTN |
| Polyprotein pp62 | Ba71V-94 | Core shell | 340 | NLVVQAYNELEQTNT |
| Polyprotein pp62 | Ba71V-94 | Core shell | 341 | LVVQAYNELEQTNTI |
| Polyprotein pp62 | Ba71V-94 | Core shell | 342 | VVQAYNELEQTNTIR |
| Polyprotein pp62 | Ba71V-94 | Core shell | 343 | VQAYNELEQTNTIRH |
| Polyprotein pp62 | Ba71V-94 | Core shell | 344 | QAYNELEQTNTIRHY |
| Polyprotein pp62 | Ba71V-94 | Core shell | 345 | AYNELEQTNTIRHYG |
| Polyprotein pp62 | Ba71V-94 | Core shell | 346 | YNELEQTNTIRHYGP |
| Polyprotein pp62 | Ba71V-94 | Core shell | 347 | NELEQTNTIRHYGPI |
| Polyprotein pp62 | Ba71V-94 | Core shell | 348 | ELEQTNTIRHYGPIF |
| Polyprotein pp62 | Ba71V-94 | Core shell | 349 | LEQTNTIRHYGPIFP |
| Polyprotein pp62 | Ba71V-94 | Core shell | 350 | EQTNTIRHYGPIFPE |
| Polyprotein pp62 | Ba71V-94 | Core shell | 351 | QTNTIRHYGPIFPES |
| Polyprotein pp62 | Ba71V-94 | Core shell | 352 | TNTIRHYGPIFPEST |
| Polyprotein pp62 | Ba71V-94 | Core shell | 353 | NTIRHYGPIFPESTI |
| Polyprotein pp62 | Ba71V-94 | Core shell | 354 | TIRHYGPIFPESTIN |
| Polyprotein pp62 | Ba71V-94 | Core shell | 355 | IRHYGPIFPESTINA |
| Polyprotein pp62 | Ba71V-94 | Core shell | 356 | RHYGPIFPESTINAL |
| Polyprotein pp62 | Ba71V-94 | Core shell | 357 | HYGPIFPESTINALR |
| Polyprotein pp62 | Ba71V-94 | Core shell | 358 | YGPIFPESTINALRF |

| | | | |
|---|---|---|---|
| Polyprotein pp62 | Ba71V-94 | Core shell | 359 GPIFPESTINALRFW |
| Polyprotein pp62 | Ba71V-94 | Core shell | 360 PIFPESTINALRFWK |
| Polyprotein pp62 | Ba71V-94 | Core shell | 361 IFPESTINALRFWKK |
| Polyprotein pp62 | Ba71V-94 | Core shell | 362 FPESTINALRFWKKL |
| Polyprotein pp62 | Ba71V-94 | Core shell | 363 PESTINALRFWKKLW |
| Polyprotein pp62 | Ba71V-94 | Core shell | 364 ESTINALRFWKKLWQ |
| Polyprotein pp62 | Ba71V-94 | Core shell | 365 STINALRFWKKLWQD |
| Polyprotein pp62 | Ba71V-94 | Core shell | 366 TINALRFWKKLWQDE |
| Polyprotein pp62 | Ba71V-94 | Core shell | 367 INALRFWKKLWQDEQ |
| Polyprotein pp62 | Ba71V-94 | Core shell | 368 NALRFWKKLWQDEQR |
| Polyprotein pp62 | Ba71V-94 | Core shell | 369 ALRFWKKLWQDEQRF |
| Polyprotein pp62 | Ba71V-94 | Core shell | 370 LRFWKKLWQDEQRFV |
| Polyprotein pp62 | Ba71V-94 | Core shell | 371 RFWKKLWQDEQRFVI |
| Polyprotein pp62 | Ba71V-94 | Core shell | 372 FWKKLWQDEQRFVIH |
| Polyprotein pp62 | Ba71V-94 | Core shell | 373 WKKLWQDEQRFVIHG |
| Polyprotein pp62 | Ba71V-94 | Core shell | 374 KKLWQDEQRFVIHGL |
| Polyprotein pp62 | Ba71V-94 | Core shell | 375 KLWQDEQRFVIHGLH |
| Polyprotein pp62 | Ba71V-94 | Core shell | 376 LWQDEQRFVIHGLHR |
| Polyprotein pp62 | Ba71V-94 | Core shell | 377 WQDEQRFVIHGLHRT |
| Polyprotein pp62 | Ba71V-94 | Core shell | 378 QDEQRFVIHGLHRTL |
| Polyprotein pp62 | Ba71V-94 | Core shell | 379 DEQRFVIHGLHRTLM |
| Polyprotein pp62 | Ba71V-94 | Core shell | 380 EQRFVIHGLHRTLMD |
| Polyprotein pp62 | Ba71V-94 | Core shell | 381 QRFVIHGLHRTLMDQ |
| Polyprotein pp62 | Ba71V-94 | Core shell | 382 RFVIHGLHRTLMDQP |
| Polyprotein pp62 | Ba71V-94 | Core shell | 383 FVIHGLHRTLMDQPT |
| Polyprotein pp62 | Ba71V-94 | Core shell | 384 VIHGLHRTLMDQPTY |
| Polyprotein pp62 | Ba71V-94 | Core shell | 385 IHGLHRTLMDQPTYE |
| Polyprotein pp62 | Ba71V-94 | Core shell | 386 HGLHRTLMDQPTYET |
| Polyprotein pp62 | Ba71V-94 | Core shell | 387 GLHRTLMDQPTYETS |
| Polyprotein pp62 | Ba71V-94 | Core shell | 388 LHRTLMDQPTYETSE |
| Polyprotein pp62 | Ba71V-94 | Core shell | 389 HRTLMDQPTYETSEF |
| Polyprotein pp62 | Ba71V-94 | Core shell | 390 RTLMDQPTYETSEFA |
| Polyprotein pp62 | Ba71V-94 | Core shell | 391 TLMDQPTYETSEFAE |
| Polyprotein pp62 | Ba71V-94 | Core shell | 392 LMDQPTYETSEFAEI |
| Polyprotein pp62 | Ba71V-94 | Core shell | 393 MDQPTYETSEFAEIV |
| Polyprotein pp62 | Ba71V-94 | Core shell | 394 DQPTYETSEFAEIVR |
| Polyprotein pp62 | Ba71V-94 | Core shell | 395 QPTYETSEFAEIVRN |
| Polyprotein pp62 | Ba71V-94 | Core shell | 396 PTYETSEFAEIVRNL |
| Polyprotein pp62 | Ba71V-94 | Core shell | 397 TYETSEFAEIVRNLR |
| Polyprotein pp62 | Ba71V-94 | Core shell | 398 YETSEFAEIVRNLRF |
| Polyprotein pp62 | Ba71V-94 | Core shell | 399 ETSEFAEIVRNLRFS |
| Polyprotein pp62 | Ba71V-94 | Core shell | 400 TSEFAEIVRNLRFSR |
| Polyprotein pp62 | Ba71V-94 | Core shell | 401 SEFAEIVRNLRFSRP |
| Polyprotein pp62 | Ba71V-94 | Core shell | 402 EFAEIVRNLRFSRPG |
| Polyprotein pp62 | Ba71V-94 | Core shell | 403 FAEIVRNLRFSRPGN |
| Polyprotein pp62 | Ba71V-94 | Core shell | 404 AEIVRNLRFSRPGNN |
| Polyprotein pp62 | Ba71V-94 | Core shell | 405 EIVRNLRFSRPGNNY |
| Polyprotein pp62 | Ba71V-94 | Core shell | 406 IVRNLRFSRPGNNYI |
| Polyprotein pp62 | Ba71V-94 | Core shell | 407 VRNLRFSRPGNNYIN |
| Polyprotein pp62 | Ba71V-94 | Core shell | 408 RNLRFSRPGNNYINE |
| Polyprotein pp62 | Ba71V-94 | Core shell | 409 NLRFSRPGNNYINEL |
| Polyprotein pp62 | Ba71V-94 | Core shell | 410 LRFSRPGNNYINELN |
| Polyprotein pp62 | Ba71V-94 | Core shell | 411 RFSRPGNNYINELNI |
| Polyprotein pp62 | Ba71V-94 | Core shell | 412 FSRPGNNYINELNIT |
| Polyprotein pp62 | Ba71V-94 | Core shell | 413 SRPGNNYINELNITS |
| Polyprotein pp62 | Ba71V-94 | Core shell | 414 RPGNNYINELNITSP |
| Polyprotein pp62 | Ba71V-94 | Core shell | 415 PGNNYINELNITSPA |
| Polyprotein pp62 | Ba71V-94 | Core shell | 416 GNNYINELNITSPAM |
| Polyprotein pp62 | Ba71V-94 | Core shell | 417 NNYINELNITSPAMY |
| Polyprotein pp62 | Ba71V-94 | Core shell | 418 NYINELNITSPAMYG |
| Polyprotein pp62 | Ba71V-94 | Core shell | 419 YINELNITSPAMYGD |
| Polyprotein pp62 | Ba71V-94 | Core shell | 420 INELNITSPAMYGDK |
| Polyprotein pp62 | Ba71V-94 | Core shell | 421 NELNITSPAMYGDKH |
| Polyprotein pp62 | Ba71V-94 | Core shell | 422 ELNITSPAMYGDKHT |
| Polyprotein pp62 | Ba71V-94 | Core shell | 423 LNITSPAMYGDKHTT |
| Polyprotein pp62 | Ba71V-94 | Core shell | 424 NITSPAMYGDKHTTG |
| Polyprotein pp62 | Ba71V-94 | Core shell | 425 ITSPAMYGDKHTTGD |

| | | | |
|---|---|---|---|
| Polyprotein pp62 | Ba71V-94 | Core shell | 426 TSPAMYGDKHTTGDI |
| Polyprotein pp62 | Ba71V-94 | Core shell | 427 SPAMYGDKHTTGDIA |
| Polyprotein pp62 | Ba71V-94 | Core shell | 428 PAMYGDKHTTGDIAP |
| Polyprotein pp62 | Ba71V-94 | Core shell | 429 AMYGDKHTTGDIAPN |
| Polyprotein pp62 | Ba71V-94 | Core shell | 430 MYGDKHTTGDIAPND |
| Polyprotein pp62 | Ba71V-94 | Core shell | 431 YGDKHTTGDIAPNDR |
| Polyprotein pp62 | Ba71V-94 | Core shell | 432 GDKHTTGDIAPNDRF |
| Polyprotein pp62 | Ba71V-94 | Core shell | 433 DKHTTGDIAPNDRFA |
| Polyprotein pp62 | Ba71V-94 | Core shell | 434 KHTTGDIAPNDRFAM |
| Polyprotein pp62 | Ba71V-94 | Core shell | 435 HTTGDIAPNDRFAML |
| Polyprotein pp62 | Ba71V-94 | Core shell | 436 TTGDIAPNDRFAMLV |
| Polyprotein pp62 | Ba71V-94 | Core shell | 437 TGDIAPNDRFAMLVA |
| Polyprotein pp62 | Ba71V-94 | Core shell | 438 GDIAPNDRFAMLVAF |
| Polyprotein pp62 | Ba71V-94 | Core shell | 439 DIAPNDRFAMLVAFI |
| Polyprotein pp62 | Ba71V-94 | Core shell | 440 IAPNDRFAMLVAFIN |
| Polyprotein pp62 | Ba71V-94 | Core shell | 441 APNDRFAMLVAFINS |
| Polyprotein pp62 | Ba71V-94 | Core shell | 442 PNDRFAMLVAFINST |
| Polyprotein pp62 | Ba71V-94 | Core shell | 443 NDRFAMLVAFINSTD |
| Polyprotein pp62 | Ba71V-94 | Core shell | 444 DRFAMLVAFINSTDF |
| Polyprotein pp62 | Ba71V-94 | Core shell | 445 RFAMLVAFINSTDFL |
| Polyprotein pp62 | Ba71V-94 | Core shell | 446 FAMLVAFINSTDFLY |
| Polyprotein pp62 | Ba71V-94 | Core shell | 447 AMLVAFINSTDFLYT |
| Polyprotein pp62 | Ba71V-94 | Core shell | 448 MLVAFINSTDFLYTA |
| Polyprotein pp62 | Ba71V-94 | Core shell | 449 LVAFINSTDFLYTAI |
| Polyprotein pp62 | Ba71V-94 | Core shell | 450 VAFINSTDFLYTAIP |
| Polyprotein pp62 | Ba71V-94 | Core shell | 451 AFINSTDFLYTAIPE |
| Polyprotein pp62 | Ba71V-94 | Core shell | 452 FINSTDFLYTAIPEE |
| Polyprotein pp62 | Ba71V-94 | Core shell | 453 INSTDFLYTAIPEEK |
| Polyprotein pp62 | Ba71V-94 | Core shell | 454 NSTDFLYTAIPEEKV |
| Polyprotein pp62 | Ba71V-94 | Core shell | 455 STDFLYTAIPEEKVG |
| Polyprotein pp62 | Ba71V-94 | Core shell | 456 TDFLYTAIPEEKVGG |
| Polyprotein pp62 | Ba71V-94 | Core shell | 457 DFLYTAIPEEKVGGN |
| Polyprotein pp62 | Ba71V-94 | Core shell | 458 FLYTAIPEEKVGGNE |
| Polyprotein pp62 | Ba71V-94 | Core shell | 459 LYTAIPEEKVGGNET |
| Polyprotein pp62 | Ba71V-94 | Core shell | 460 YTAIPEEKVGGNETQ |
| Polyprotein pp62 | Ba71V-94 | Core shell | 461 TAIPEEKVGGNETQT |
| Polyprotein pp62 | Ba71V-94 | Core shell | 462 AIPEEKVGGNETQTS |
| Polyprotein pp62 | Ba71V-94 | Core shell | 463 IPEEKVGGNETQTSS |
| Polyprotein pp62 | Ba71V-94 | Core shell | 464 PEEKVGGNETQTSSL |
| Polyprotein pp62 | Ba71V-94 | Core shell | 465 EEKVGGNETQTSSLT |
| Polyprotein pp62 | Ba71V-94 | Core shell | 466 EKVGGNETQTSSLTD |
| Polyprotein pp62 | Ba71V-94 | Core shell | 467 KVGGNETQTSSLTDL |
| Polyprotein pp62 | Ba71V-94 | Core shell | 468 VGGNETQTSSLTDLV |
| Polyprotein pp62 | Ba71V-94 | Core shell | 469 GGNETQTSSLTDLVP |
| Polyprotein pp62 | Ba71V-94 | Core shell | 470 GNETQTSSLTDLVPT |
| Polyprotein pp62 | Ba71V-94 | Core shell | 471 NETQTSSLTDLVPTR |
| Polyprotein pp62 | Ba71V-94 | Core shell | 472 ETQTSSLTDLVPTRL |
| Polyprotein pp62 | Ba71V-94 | Core shell | 473 TQTSSLTDLVPTRLH |
| Polyprotein pp62 | Ba71V-94 | Core shell | 474 QTSSLTDLVPTRLHS |
| Polyprotein pp62 | Ba71V-94 | Core shell | 475 TSSLTDLVPTRLHSF |
| Polyprotein pp62 | Ba71V-94 | Core shell | 476 SSLTDLVPTRLHSFL |
| Polyprotein pp62 | Ba71V-94 | Core shell | 477 SLTDLVPTRLHSFLN |
| Polyprotein pp62 | Ba71V-94 | Core shell | 478 LTDLVPTRLHSFLNH |
| Polyprotein pp62 | Ba71V-94 | Core shell | 479 TDLVPTRLHSFLNHN |
| Polyprotein pp62 | Ba71V-94 | Core shell | 480 DLVPTRLHSFLNHNL |
| Polyprotein pp62 | Ba71V-94 | Core shell | 481 LVPTRLHSFLNHNLS |
| Polyprotein pp62 | Ba71V-94 | Core shell | 482 VPTRLHSFLNHNLSK |
| Polyprotein pp62 | Ba71V-94 | Core shell | 483 PTRLHSFLNHNLSKL |
| Polyprotein pp62 | Ba71V-94 | Core shell | 484 TRLHSFLNHNLSKLK |
| Polyprotein pp62 | Ba71V-94 | Core shell | 485 RLHSFLNHNLSKLKI |
| Polyprotein pp62 | Ba71V-94 | Core shell | 486 LHSFLNHNLSKLKIL |
| Polyprotein pp62 | Ba71V-94 | Core shell | 487 HSFLNHNLSKLKILN |
| Polyprotein pp62 | Ba71V-94 | Core shell | 488 SFLNHNLSKLKILNR |
| Polyprotein pp62 | Ba71V-94 | Core shell | 489 FLNHNLSKLKILNRA |
| Polyprotein pp62 | Ba71V-94 | Core shell | 490 LNHNLSKLKILNRAQ |
| Polyprotein pp62 | Ba71V-94 | Core shell | 491 NHNLSKLKILNRAQQ |
| Polyprotein pp62 | Ba71V-94 | Core shell | 492 HNLSKLKILNRAQQT |

| | | | | |
|---|---|---|---|---|
| Polyprotein pp62 | Ba71V-94 | Core shell | 493 | NLSKLKILNRAQQTV |
| Polyprotein pp62 | Ba71V-94 | Core shell | 494 | LSKLKILNRAQQTVR |
| Polyprotein pp62 | Ba71V-94 | Core shell | 495 | SKLKILNRAQQTVRN |
| Polyprotein pp62 | Ba71V-94 | Core shell | 496 | KLKILNRAQQTVRNI |
| Polyprotein pp62 | Ba71V-94 | Core shell | 497 | LKILNRAQQTVRNIL |
| Polyprotein pp62 | Ba71V-94 | Core shell | 498 | KILNRAQQTVRNILS |
| Polyprotein pp62 | Ba71V-94 | Core shell | 499 | ILNRAQQTVRNILSN |
| Polyprotein pp62 | Ba71V-94 | Core shell | 500 | LNRAQQTVRNILSND |
| Polyprotein pp62 | Ba71V-94 | Core shell | 501 | NRAQQTVRNILSNDC |
| Polyprotein pp62 | Ba71V-94 | Core shell | 502 | RAQQTVRNILSNDCL |
| Polyprotein pp62 | Ba71V-94 | Core shell | 503 | AQQTVRNILSNDCLN |
| Polyprotein pp62 | Ba71V-94 | Core shell | 504 | QQTVRNILSNDCLNQ |
| Polyprotein pp62 | Ba71V-94 | Core shell | 505 | QTVRNILSNDCLNQL |
| Polyprotein pp62 | Ba71V-94 | Core shell | 506 | TVRNILSNDCLNQLK |
| Polyprotein pp62 | Ba71V-94 | Core shell | 507 | VRNILSNDCLNQLKH |
| Polyprotein pp62 | Ba71V-94 | Core shell | 508 | RNILSNDCLNQLKHY |
| Polyprotein pp62 | Ba71V-94 | Core shell | 509 | NILSNDCLNQLKHYV |
| Polyprotein pp62 | Ba71V-94 | Core shell | 510 | ILSNDCLNQLKHYVK |
| Polyprotein pp62 | Ba71V-94 | Core shell | 511 | LSNDCLNQLKHYVKH |
| Polyprotein pp62 | Ba71V-94 | Core shell | 512 | SNDCLNQLKHYVKHT |
| Polyprotein pp62 | Ba71V-94 | Core shell | 513 | NDCLNQLKHYVKHTG |
| Polyprotein pp62 | Ba71V-94 | Core shell | 514 | DCLNQLKHYVKHTGK |
| Polyprotein pp62 | Ba71V-94 | Core shell | 515 | CLNQLKHYVKHTGKN |
| Polyprotein pp62 | Ba71V-94 | Core shell | 516 | LNQLKHYVKHTGKNE |
| Polyprotein pp62 | Ba71V-94 | Core shell | 517 | NQLKHYVKHTGKNEI |
| Polyprotein pp62 | Ba71V-94 | Core shell | 518 | QLKHYVKHTGKNEIL |
| Polyprotein pp62 | Ba71V-94 | Core shell | 519 | LKHYVKHTGKNEILK |
| Polyprotein pp62 | Ba71V-94 | Core shell | 520 | KHYVKHTGKNEILKL |
| Polyprotein pp62 | Ba71V-94 | Core shell | 521 | HYVKHTGKNEILKLL |
| Polyprotein pp62 | Ba71V-94 | Core shell | 522 | YVKHTGKNEILKLLQ |
| Polyprotein pp62 | Ba71V-94 | Core shell | 523 | VKHTGKNEILKLLQE |
| Polyprotein pp62 | Ba71V-94 | Core shell | 524 | KHTGKNEILKLLQEG |
| Polyprotein pp62 | Ba71V-94 | Core shell | 525 | HTGKNEILKLLQEGS |
| Polyprotein pp62 | Ba71V-94 | Core shell | 526 | TGKNEILKLLQEGSG |
| Polyprotein pp62 | Ba71V-94 | Core shell | 527 | GKNEILKLLQEGSGS |
| Polyprotein pp62 | Ba71V-94 | Core shell | 528 | KNEILKLLQEGSGSG |
| Polyprotein pp62 | Ba71V-94 | Core shell | 529 | NEILKLLQEGSGSGS |
| Polyprotein pp62 | Ba71V-94 | Core shell | 530 | EILKLLQEGSGSGSG |
| Cysteine protease S273R | S273R CDS | Core shell | 1 | GSGSGSGMSILEKIT |
| Cysteine protease S273R | S273R CDS | Core shell | 2 | SGSGSGMSILEKITS |
| Cysteine protease S273R | S273R CDS | Core shell | 3 | GSGSGMSILEKITSS |
| Cysteine protease S273R | S273R CDS | Core shell | 4 | SGSGMSILEKITSSP |
| Cysteine protease S273R | S273R CDS | Core shell | 5 | GSGMSILEKITSSPS |
| Cysteine protease S273R | S273R CDS | Core shell | 6 | SGMSILEKITSSPSE |
| Cysteine protease S273R | S273R CDS | Core shell | 7 | GMSILEKITSSPSEC |
| Cysteine protease S273R | S273R CDS | Core shell | 8 | MSILEKITSSPSECA |
| Cysteine protease S273R | S273R CDS | Core shell | 9 | SILEKITSSPSECAE |
| Cysteine protease S273R | S273R CDS | Core shell | 10 | ILEKITSSPSECAEH |
| Cysteine protease S273R | S273R CDS | Core shell | 11 | LEKITSSPSECAEHL |
| Cysteine protease S273R | S273R CDS | Core shell | 12 | EKITSSPSECAEHLT |
| Cysteine protease S273R | S273R CDS | Core shell | 13 | KITSSPSECAEHLTN |
| Cysteine protease S273R | S273R CDS | Core shell | 14 | ITSSPSECAEHLTNK |
| Cysteine protease S273R | S273R CDS | Core shell | 15 | TSSPSECAEHLTNKD |
| Cysteine protease S273R | S273R CDS | Core shell | 16 | SSPSECAEHLTNKDS |
| Cysteine protease S273R | S273R CDS | Core shell | 17 | SPSECAEHLTNKDSC |
| Cysteine protease S273R | S273R CDS | Core shell | 18 | PSECAEHLTNKDSCL |
| Cysteine protease S273R | S273R CDS | Core shell | 19 | SECAEHLTNKDSCLS |
| Cysteine protease S273R | S273R CDS | Core shell | 20 | ECAEHLTNKDSCLSK |
| Cysteine protease S273R | S273R CDS | Core shell | 21 | CAEHLTNKDSCLSKK |
| Cysteine protease S273R | S273R CDS | Core shell | 22 | AEHLTNKDSCLSKKI |
| Cysteine protease S273R | S273R CDS | Core shell | 23 | EHLTNKDSCLSKKIQ |
| Cysteine protease S273R | S273R CDS | Core shell | 24 | HLTNKDSCLSKKIQK |
| Cysteine protease S273R | S273R CDS | Core shell | 25 | LTNKDSCLSKKIQKE |
| Cysteine protease S273R | S273R CDS | Core shell | 26 | TNKDSCLSKKIQKEL |
| Cysteine protease S273R | S273R CDS | Core shell | 27 | NKDSCLSKKIQKELT |
| Cysteine protease S273R | S273R CDS | Core shell | 28 | KDSCLSKKIQKELTS |
| Cysteine protease S273R | S273R CDS | Core shell | 29 | DSCLSKKIQKELTSF |

| | | | | |
|---|---|---|---|---|
| Cysteine protease S273R | S273R CDS | Core shell | 30 | SCLSKKIQKELTSFL |
| Cysteine protease S273R | S273R CDS | Core shell | 31 | CLSKKIQKELTSFLE |
| Cysteine protease S273R | S273R CDS | Core shell | 32 | LSKKIQKELTSFLEK |
| Cysteine protease S273R | S273R CDS | Core shell | 33 | SKKIQKELTSFLEKK |
| Cysteine protease S273R | S273R CDS | Core shell | 34 | KKIQKELTSFLEKKE |
| Cysteine protease S273R | S273R CDS | Core shell | 35 | KIQKELTSFLEKKET |
| Cysteine protease S273R | S273R CDS | Core shell | 36 | IQKELTSFLEKKETL |
| Cysteine protease S273R | S273R CDS | Core shell | 37 | QKELTSFLEKKETLG |
| Cysteine protease S273R | S273R CDS | Core shell | 38 | KELTSFLEKKETLGC |
| Cysteine protease S273R | S273R CDS | Core shell | 39 | ELTSFLEKKETLGCD |
| Cysteine protease S273R | S273R CDS | Core shell | 40 | LTSFLEKKETLGCDS |
| Cysteine protease S273R | S273R CDS | Core shell | 41 | TSFLEKKETLGCDSE |
| Cysteine protease S273R | S273R CDS | Core shell | 42 | SFLEKKETLGCDSES |
| Cysteine protease S273R | S273R CDS | Core shell | 43 | FLEKKETLGCDSESC |
| Cysteine protease S273R | S273R CDS | Core shell | 44 | LEKKETLGCDSESCV |
| Cysteine protease S273R | S273R CDS | Core shell | 45 | EKKETLGCDSESCVI |
| Cysteine protease S273R | S273R CDS | Core shell | 46 | KKETLGCDSESCVIT |
| Cysteine protease S273R | S273R CDS | Core shell | 47 | KETLGCDSESCVITH |
| Cysteine protease S273R | S273R CDS | Core shell | 48 | ETLGCDSESCVITHP |
| Cysteine protease S273R | S273R CDS | Core shell | 49 | TLGCDSESCVITHPA |
| Cysteine protease S273R | S273R CDS | Core shell | 50 | LGCDSESCVITHPAV |
| Cysteine protease S273R | S273R CDS | Core shell | 51 | GCDSESCVITHPAVK |
| Cysteine protease S273R | S273R CDS | Core shell | 52 | CDSESCVITHPAVKA |
| Cysteine protease S273R | S273R CDS | Core shell | 53 | DSESCVITHPAVKAY |
| Cysteine protease S273R | S273R CDS | Core shell | 54 | SESCVITHPAVKAYA |
| Cysteine protease S273R | S273R CDS | Core shell | 55 | ESCVITHPAVKAYAQ |
| Cysteine protease S273R | S273R CDS | Core shell | 56 | SCVITHPAVKAYAQQ |
| Cysteine protease S273R | S273R CDS | Core shell | 57 | CVITHPAVKAYAQQK |
| Cysteine protease S273R | S273R CDS | Core shell | 58 | VITHPAVKAYAQQKG |
| Cysteine protease S273R | S273R CDS | Core shell | 59 | ITHPAVKAYAQQKGL |
| Cysteine protease S273R | S273R CDS | Core shell | 60 | THPAVKAYAQQKGLD |
| Cysteine protease S273R | S273R CDS | Core shell | 61 | HPAVKAYAQQKGLDL |
| Cysteine protease S273R | S273R CDS | Core shell | 62 | PAVKAYAQQKGLDLS |
| Cysteine protease S273R | S273R CDS | Core shell | 63 | AVKAYAQQKGLDLSK |
| Cysteine protease S273R | S273R CDS | Core shell | 64 | VKAYAQQKGLDLSKE |
| Cysteine protease S273R | S273R CDS | Core shell | 65 | KAYAQQKGLDLSKEL |
| Cysteine protease S273R | S273R CDS | Core shell | 66 | AYAQQKGLDLSKELE |
| Cysteine protease S273R | S273R CDS | Core shell | 67 | YAQQKGLDLSKELET |
| Cysteine protease S273R | S273R CDS | Core shell | 68 | AQQKGLDLSKELETR |
| Cysteine protease S273R | S273R CDS | Core shell | 69 | QQKGLDLSKELETRF |
| Cysteine protease S273R | S273R CDS | Core shell | 70 | QKGLDLSKELETRFK |
| Cysteine protease S273R | S273R CDS | Core shell | 71 | KGLDLSKELETRFKA |
| Cysteine protease S273R | S273R CDS | Core shell | 72 | GLDLSKELETRFKAP |
| Cysteine protease S273R | S273R CDS | Core shell | 73 | LDLSKELETRFKAPG |
| Cysteine protease S273R | S273R CDS | Core shell | 74 | DLSKELETRFKAPGP |
| Cysteine protease S273R | S273R CDS | Core shell | 75 | LSKELETRFKAPGPR |
| Cysteine protease S273R | S273R CDS | Core shell | 76 | SKELETRFKAPGPRN |
| Cysteine protease S273R | S273R CDS | Core shell | 77 | KELETRFKAPGPRNN |
| Cysteine protease S273R | S273R CDS | Core shell | 78 | ELETRFKAPGPRNNT |
| Cysteine protease S273R | S273R CDS | Core shell | 79 | LETRFKAPGPRNNTG |
| Cysteine protease S273R | S273R CDS | Core shell | 80 | ETRFKAPGPRNNTGL |
| Cysteine protease S273R | S273R CDS | Core shell | 81 | TRFKAPGPRNNTGLL |
| Cysteine protease S273R | S273R CDS | Core shell | 82 | RFKAPGPRNNTGLLT |
| Cysteine protease S273R | S273R CDS | Core shell | 83 | FKAPGPRNNTGLLTN |
| Cysteine protease S273R | S273R CDS | Core shell | 84 | KAPGPRNNTGLLTNF |
| Cysteine protease S273R | S273R CDS | Core shell | 85 | APGPRNNTGLLTNFN |
| Cysteine protease S273R | S273R CDS | Core shell | 86 | PGPRNNTGLLTNFNI |
| Cysteine protease S273R | S273R CDS | Core shell | 87 | GPRNNTGLLTNFNID |
| Cysteine protease S273R | S273R CDS | Core shell | 88 | PRNNTGLLTNFNIDE |
| Cysteine protease S273R | S273R CDS | Core shell | 89 | RNNTGLLTNFNIDET |
| Cysteine protease S273R | S273R CDS | Core shell | 90 | NNTGLLTNFNIDETL |
| Cysteine protease S273R | S273R CDS | Core shell | 91 | NTGLLTNFNIDETLQ |
| Cysteine protease S273R | S273R CDS | Core shell | 92 | TGLLTNFNIDETLQR |
| Cysteine protease S273R | S273R CDS | Core shell | 93 | GLLTNFNIDETLQRW |
| Cysteine protease S273R | S273R CDS | Core shell | 94 | LLTNFNIDETLQRWA |
| Cysteine protease S273R | S273R CDS | Core shell | 95 | LTNFNIDETLQRWAI |
| Cysteine protease S273R | S273R CDS | Core shell | 96 | TNFNIDETLQRWAIK |

| | | | | |
|---|---|---|---|---|
| Cysteine protease S273R | S273R CDS | Core shell | 97 | NFNIDETLQRWAIKY |
| Cysteine protease S273R | S273R CDS | Core shell | 98 | FNIDETLQRWAIKYT |
| Cysteine protease S273R | S273R CDS | Core shell | 99 | NIDETLQRWAIKYTK |
| Cysteine protease S273R | S273R CDS | Core shell | 100 | IDETLQRWAIKYTKF |
| Cysteine protease S273R | S273R CDS | Core shell | 101 | DETLQRWAIKYTKFF |
| Cysteine protease S273R | S273R CDS | Core shell | 102 | ETLQRWAIKYTKFFN |
| Cysteine protease S273R | S273R CDS | Core shell | 103 | TLQRWAIKYTKFFNC |
| Cysteine protease S273R | S273R CDS | Core shell | 104 | LQRWAIKYTKFFNCP |
| Cysteine protease S273R | S273R CDS | Core shell | 105 | QRWAIKYTKFFNCPF |
| Cysteine protease S273R | S273R CDS | Core shell | 106 | RWAIKYTKFFNCPFS |
| Cysteine protease S273R | S273R CDS | Core shell | 107 | WAIKYTKFFNCPFSM |
| Cysteine protease S273R | S273R CDS | Core shell | 108 | AIKYTKFFNCPFSMM |
| Cysteine protease S273R | S273R CDS | Core shell | 109 | IKYTKFFNCPFSMMD |
| Cysteine protease S273R | S273R CDS | Core shell | 110 | KYTKFFNCPFSMMDF |
| Cysteine protease S273R | S273R CDS | Core shell | 111 | YTKFFNCPFSMMDFE |
| Cysteine protease S273R | S273R CDS | Core shell | 112 | TKFFNCPFSMMDFER |
| Cysteine protease S273R | S273R CDS | Core shell | 113 | KFFNCPFSMMDFERV |
| Cysteine protease S273R | S273R CDS | Core shell | 114 | FFNCPFSMMDFERVH |
| Cysteine protease S273R | S273R CDS | Core shell | 115 | FNCPFSMMDFERVHY |
| Cysteine protease S273R | S273R CDS | Core shell | 116 | NCPFSMMDFERVHYK |
| Cysteine protease S273R | S273R CDS | Core shell | 117 | CPFSMMDFERVHYKF |
| Cysteine protease S273R | S273R CDS | Core shell | 118 | PFSMMDFERVHYKFN |
| Cysteine protease S273R | S273R CDS | Core shell | 119 | FSMMDFERVHYKFNQ |
| Cysteine protease S273R | S273R CDS | Core shell | 120 | SMMDFERVHYKFNQV |
| Cysteine protease S273R | S273R CDS | Core shell | 121 | MMDFERVHYKFNQVD |
| Cysteine protease S273R | S273R CDS | Core shell | 122 | MDFERVHYKFNQVDM |
| Cysteine protease S273R | S273R CDS | Core shell | 123 | DFERVHYKFNQVDMV |
| Cysteine protease S273R | S273R CDS | Core shell | 124 | FERVHYKFNQVDMVK |
| Cysteine protease S273R | S273R CDS | Core shell | 125 | ERVHYKFNQVDMVKV |
| Cysteine protease S273R | S273R CDS | Core shell | 126 | RVHYKFNQVDMVKVY |
| Cysteine protease S273R | S273R CDS | Core shell | 127 | VHYKFNQVDMVKVYK |
| Cysteine protease S273R | S273R CDS | Core shell | 128 | HYKFNQVDMVKVYKG |
| Cysteine protease S273R | S273R CDS | Core shell | 129 | YKFNQVDMVKVYKGE |
| Cysteine protease S273R | S273R CDS | Core shell | 130 | KFNQVDMVKVYKGEE |
| Cysteine protease S273R | S273R CDS | Core shell | 131 | FNQVDMVKVYKGEEL |
| Cysteine protease S273R | S273R CDS | Core shell | 132 | NQVDMVKVYKGEELQ |
| Cysteine protease S273R | S273R CDS | Core shell | 133 | QVDMVKVYKGEELQY |
| Cysteine protease S273R | S273R CDS | Core shell | 134 | VDMVKVYKGEELQYV |
| Cysteine protease S273R | S273R CDS | Core shell | 135 | DMVKVYKGEELQYVE |
| Cysteine protease S273R | S273R CDS | Core shell | 136 | MVKVYKGEELQYVEG |
| Cysteine protease S273R | S273R CDS | Core shell | 137 | VKVYKGEELQYVEGK |
| Cysteine protease S273R | S273R CDS | Core shell | 138 | KVYKGEELQYVEGKV |
| Cysteine protease S273R | S273R CDS | Core shell | 139 | VYKGEELQYVEGKVV |
| Cysteine protease S273R | S273R CDS | Core shell | 140 | YKGEELQYVEGKVVK |
| Cysteine protease S273R | S273R CDS | Core shell | 141 | KGEELQYVEGKVVKR |
| Cysteine protease S273R | S273R CDS | Core shell | 142 | GEELQYVEGKVVKRP |
| Cysteine protease S273R | S273R CDS | Core shell | 143 | EELQYVEGKVVKRPC |
| Cysteine protease S273R | S273R CDS | Core shell | 144 | ELQYVEGKVVKRPCN |
| Cysteine protease S273R | S273R CDS | Core shell | 145 | LQYVEGKVVKRPCNT |
| Cysteine protease S273R | S273R CDS | Core shell | 146 | QYVEGKVVKRPCNTF |
| Cysteine protease S273R | S273R CDS | Core shell | 147 | YVEGKVVKRPCNTFG |
| Cysteine protease S273R | S273R CDS | Core shell | 148 | VEGKVVKRPCNTFGC |
| Cysteine protease S273R | S273R CDS | Core shell | 149 | EGKVVKRPCNTFGCV |
| Cysteine protease S273R | S273R CDS | Core shell | 150 | GKVVKRPCNTFGCVL |
| Cysteine protease S273R | S273R CDS | Core shell | 151 | KVVKRPCNTFGCVLN |
| Cysteine protease S273R | S273R CDS | Core shell | 152 | VVKRPCNTFGCVLNT |
| Cysteine protease S273R | S273R CDS | Core shell | 153 | VKRPCNTFGCVLNTD |
| Cysteine protease S273R | S273R CDS | Core shell | 154 | KRPCNTFGCVLNTDF |
| Cysteine protease S273R | S273R CDS | Core shell | 155 | RPCNTFGCVLNTDFS |
| Cysteine protease S273R | S273R CDS | Core shell | 156 | PCNTFGCVLNTDFST |
| Cysteine protease S273R | S273R CDS | Core shell | 157 | CNTFGCVLNTDFSTG |
| Cysteine protease S273R | S273R CDS | Core shell | 158 | NTFGCVLNTDFSTGT |
| Cysteine protease S273R | S273R CDS | Core shell | 159 | TFGCVLNTDFSTGTG |
| Cysteine protease S273R | S273R CDS | Core shell | 160 | FGCVLNTDFSTGTGK |
| Cysteine protease S273R | S273R CDS | Core shell | 161 | GCVLNTDFSTGTGKH |
| Cysteine protease S273R | S273R CDS | Core shell | 162 | CVLNTDFSTGTGKHW |
| Cysteine protease S273R | S273R CDS | Core shell | 163 | VLNTDFSTGTGKHWV |

| | | | | |
|---|---|---|---|---|
| Cysteine protease S273R | S273R CDS | Core shell | 164 | LNTDFSTGTGKHWVA |
| Cysteine protease S273R | S273R CDS | Core shell | 165 | NTDFSTGTGKHWVAI |
| Cysteine protease S273R | S273R CDS | Core shell | 166 | TDFSTGTGKHWVAIF |
| Cysteine protease S273R | S273R CDS | Core shell | 167 | DFSTGTGKHWVAIFV |
| Cysteine protease S273R | S273R CDS | Core shell | 168 | FSTGTGKHWVAIFVD |
| Cysteine protease S273R | S273R CDS | Core shell | 169 | STGTGKHWVAIFVDM |
| Cysteine protease S273R | S273R CDS | Core shell | 170 | TGTGKHWVAIFVDMR |
| Cysteine protease S273R | S273R CDS | Core shell | 171 | GTGKHWVAIFVDMRG |
| Cysteine protease S273R | S273R CDS | Core shell | 172 | TGKHWVAIFVDMRGD |
| Cysteine protease S273R | S273R CDS | Core shell | 173 | GKHWVAIFVDMRGDC |
| Cysteine protease S273R | S273R CDS | Core shell | 174 | KHWVAIFVDMRGDCW |
| Cysteine protease S273R | S273R CDS | Core shell | 175 | HWVAIFVDMRGDCWS |
| Cysteine protease S273R | S273R CDS | Core shell | 176 | WVAIFVDMRGDCWSI |
| Cysteine protease S273R | S273R CDS | Core shell | 177 | VAIFVDMRGDCWSIE |
| Cysteine protease S273R | S273R CDS | Core shell | 178 | AIFVDMRGDCWSIEY |
| Cysteine protease S273R | S273R CDS | Core shell | 179 | IFVDMRGDCWSIEYF |
| Cysteine protease S273R | S273R CDS | Core shell | 180 | FVDMRGDCWSIEYFN |
| Cysteine protease S273R | S273R CDS | Core shell | 181 | VDMRGDCWSIEYFNS |
| Cysteine protease S273R | S273R CDS | Core shell | 182 | DMRGDCWSIEYFNST |
| Cysteine protease S273R | S273R CDS | Core shell | 183 | MRGDCWSIEYFNSTG |
| Cysteine protease S273R | S273R CDS | Core shell | 184 | RGDCWSIEYFNSTGN |
| Cysteine protease S273R | S273R CDS | Core shell | 185 | GDCWSIEYFNSTGNS |
| Cysteine protease S273R | S273R CDS | Core shell | 186 | DCWSIEYFNSTGNSP |
| Cysteine protease S273R | S273R CDS | Core shell | 187 | CWSIEYFNSTGNSPP |
| Cysteine protease S273R | S273R CDS | Core shell | 188 | WSIEYFNSTGNSPPG |
| Cysteine protease S273R | S273R CDS | Core shell | 189 | SIEYFNSTGNSPPGP |
| Cysteine protease S273R | S273R CDS | Core shell | 190 | IEYFNSTGNSPPGPV |
| Cysteine protease S273R | S273R CDS | Core shell | 191 | EYFNSTGNSPPGPVI |
| Cysteine protease S273R | S273R CDS | Core shell | 192 | YFNSTGNSPPGPVIR |
| Cysteine protease S273R | S273R CDS | Core shell | 193 | FNSTGNSPPGPVIRW |
| Cysteine protease S273R | S273R CDS | Core shell | 194 | NSTGNSPPGPVIRWM |
| Cysteine protease S273R | S273R CDS | Core shell | 195 | STGNSPPGPVIRWME |
| Cysteine protease S273R | S273R CDS | Core shell | 196 | TGNSPPGPVIRWMER |
| Cysteine protease S273R | S273R CDS | Core shell | 197 | GNSPPGPVIRWMERV |
| Cysteine protease S273R | S273R CDS | Core shell | 198 | NSPPGPVIRWMERVK |
| Cysteine protease S273R | S273R CDS | Core shell | 199 | SPPGPVIRWMERVKQ |
| Cysteine protease S273R | S273R CDS | Core shell | 200 | PPGPVIRWMERVKQQ |
| Cysteine protease S273R | S273R CDS | Core shell | 201 | PGPVIRWMERVKQQL |
| Cysteine protease S273R | S273R CDS | Core shell | 202 | GPVIRWMERVKQQLL |
| Cysteine protease S273R | S273R CDS | Core shell | 203 | PVIRWMERVKQQLLK |
| Cysteine protease S273R | S273R CDS | Core shell | 204 | VIRWMERVKQQLLKI |
| Cysteine protease S273R | S273R CDS | Core shell | 205 | IRWMERVKQQLLKIH |
| Cysteine protease S273R | S273R CDS | Core shell | 206 | RWMERVKQQLLKIHH |
| Cysteine protease S273R | S273R CDS | Core shell | 207 | WMERVKQQLLKIHHT |
| Cysteine protease S273R | S273R CDS | Core shell | 208 | MERVKQQLLKIHHTV |
| Cysteine protease S273R | S273R CDS | Core shell | 209 | ERVKQQLLKIHHTVK |
| Cysteine protease S273R | S273R CDS | Core shell | 210 | RVKQQLLKIHHTVKT |
| Cysteine protease S273R | S273R CDS | Core shell | 211 | VKQQLLKIHHTVKTL |
| Cysteine protease S273R | S273R CDS | Core shell | 212 | KQQLLKIHHTVKTLA |
| Cysteine protease S273R | S273R CDS | Core shell | 213 | QQLLKIHHTVKTLAV |
| Cysteine protease S273R | S273R CDS | Core shell | 214 | QLLKIHHTVKTLAVT |
| Cysteine protease S273R | S273R CDS | Core shell | 215 | LLKIHHTVKTLAVTN |
| Cysteine protease S273R | S273R CDS | Core shell | 216 | LKIHHTVKTLAVTNI |
| Cysteine protease S273R | S273R CDS | Core shell | 217 | KIHHTVKTLAVTNIR |
| Cysteine protease S273R | S273R CDS | Core shell | 218 | IHHTVKTLAVTNIRH |
| Cysteine protease S273R | S273R CDS | Core shell | 219 | HHTVKTLAVTNIRHQ |
| Cysteine protease S273R | S273R CDS | Core shell | 220 | HTVKTLAVTNIRHQR |
| Cysteine protease S273R | S273R CDS | Core shell | 221 | TVKTLAVTNIRHQRS |
| Cysteine protease S273R | S273R CDS | Core shell | 222 | VKTLAVTNIRHQRSQ |
| Cysteine protease S273R | S273R CDS | Core shell | 223 | KTLAVTNIRHQRSQT |
| Cysteine protease S273R | S273R CDS | Core shell | 224 | TLAVTNIRHQRSQTE |
| Cysteine protease S273R | S273R CDS | Core shell | 225 | LAVTNIRHQRSQTEC |
| Cysteine protease S273R | S273R CDS | Core shell | 226 | AVTNIRHQRSQTECG |
| Cysteine protease S273R | S273R CDS | Core shell | 227 | VTNIRHQRSQTECGP |
| Cysteine protease S273R | S273R CDS | Core shell | 228 | TNIRHQRSQTECGPY |
| Cysteine protease S273R | S273R CDS | Core shell | 229 | NIRHQRSQTECGPYS |
| Cysteine protease S273R | S273R CDS | Core shell | 230 | IRHQRSQTECGPYSL |

| | | | | |
|---|---|---|---|---|
| Cysteine protease S273R | S273R CDS | Core shell | 231 | RHQRSQTECGPYSLF |
| Cysteine protease S273R | S273R CDS | Core shell | 232 | HQRSQTECGPYSLFY |
| Cysteine protease S273R | S273R CDS | Core shell | 233 | QRSQTECGPYSLFYI |
| Cysteine protease S273R | S273R CDS | Core shell | 234 | RSQTECGPYSLFYIR |
| Cysteine protease S273R | S273R CDS | Core shell | 235 | SQTECGPYSLFYIRA |
| Cysteine protease S273R | S273R CDS | Core shell | 236 | QTECGPYSLFYIRAR |
| Cysteine protease S273R | S273R CDS | Core shell | 237 | TECGPYSLFYIRARL |
| Cysteine protease S273R | S273R CDS | Core shell | 238 | ECGPYSLFYIRARLD |
| Cysteine protease S273R | S273R CDS | Core shell | 239 | CGPYSLFYIRARLDN |
| Cysteine protease S273R | S273R CDS | Core shell | 240 | GPYSLFYIRARLDNV |
| Cysteine protease S273R | S273R CDS | Core shell | 241 | PYSLFYIRARLDNVS |
| Cysteine protease S273R | S273R CDS | Core shell | 242 | YSLFYIRARLDNVSY |
| Cysteine protease S273R | S273R CDS | Core shell | 243 | SLFYIRARLDNVSYA |
| Cysteine protease S273R | S273R CDS | Core shell | 244 | LFYIRARLDNVSYAH |
| Cysteine protease S273R | S273R CDS | Core shell | 245 | FYIRARLDNVSYAHF |
| Cysteine protease S273R | S273R CDS | Core shell | 246 | YIRARLDNVSYAHFI |
| Cysteine protease S273R | S273R CDS | Core shell | 247 | IRARLDNVSYAHFIS |
| Cysteine protease S273R | S273R CDS | Core shell | 248 | RARLDNVSYAHFISA |
| Cysteine protease S273R | S273R CDS | Core shell | 249 | ARLDNVSYAHFISAR |
| Cysteine protease S273R | S273R CDS | Core shell | 250 | RLDNVSYAHFISARI |
| Cysteine protease S273R | S273R CDS | Core shell | 251 | LDNVSYAHFISARIT |
| Cysteine protease S273R | S273R CDS | Core shell | 252 | DNVSYAHFISARITD |
| Cysteine protease S273R | S273R CDS | Core shell | 253 | NVSYAHFISARITDE |
| Cysteine protease S273R | S273R CDS | Core shell | 254 | VSYAHFISARITDED |
| Cysteine protease S273R | S273R CDS | Core shell | 255 | SYAHFISARITDEDM |
| Cysteine protease S273R | S273R CDS | Core shell | 256 | YAHFISARITDEDMY |
| Cysteine protease S273R | S273R CDS | Core shell | 257 | AHFISARITDEDMYK |
| Cysteine protease S273R | S273R CDS | Core shell | 258 | HFISARITDEDMYKF |
| Cysteine protease S273R | S273R CDS | Core shell | 259 | FISARITDEDMYKFR |
| Cysteine protease S273R | S273R CDS | Core shell | 260 | ISARITDEDMYKFRT |
| Cysteine protease S273R | S273R CDS | Core shell | 261 | SARITDEDMYKFRTH |
| Cysteine protease S273R | S273R CDS | Core shell | 262 | ARITDEDMYKFRTHL |
| Cysteine protease S273R | S273R CDS | Core shell | 263 | RITDEDMYKFRTHLF |
| Cysteine protease S273R | S273R CDS | Core shell | 264 | ITDEDMYKFRTHLFR |
| Cysteine protease S273R | S273R CDS | Core shell | 265 | TDEDMYKFRTHLFRI |
| Cysteine protease S273R | S273R CDS | Core shell | 266 | DEDMYKFRTHLFRIA |
| Cysteine protease S273R | S273R CDS | Core shell | 267 | EDMYKFRTHLFRIAG |
| Cysteine protease S273R | S273R CDS | Core shell | 268 | DMYKFRTHLFRIAGS |
| Cysteine protease S273R | S273R CDS | Core shell | 269 | MYKFRTHLFRIAGSG |
| Cysteine protease S273R | S273R CDS | Core shell | 270 | YKFRTHLFRIAGSGS |
| Cysteine protease S273R | S273R CDS | Core shell | 271 | KFRTHLFRIAGSGSG |
| Cysteine protease S273R | S273R CDS | Core shell | 272 | FRTHLFRIAGSGSGS |
| Cysteine protease S273R | S273R CDS | Core shell | 273 | RTHLFRIAGSGSGSG |
| BA71V-K78R (P10) | BA71V-K78R (p10) | Nucleoid | 1 | GSGSGSGMPTKAGTK |
| BA71V-K78R (P10) | BA71V-K78R (p10) | Nucleoid | 2 | SGSGSGMPTKAGTKS |
| BA71V-K78R (P10) | BA71V-K78R (p10) | Nucleoid | 3 | GSGSGMPTKAGTKST |
| BA71V-K78R (P10) | BA71V-K78R (p10) | Nucleoid | 4 | SGSGMPTKAGTKSTA |
| BA71V-K78R (P10) | BA71V-K78R (p10) | Nucleoid | 5 | GSGMPTKAGTKSTAN |
| BA71V-K78R (P10) | BA71V-K78R (p10) | Nucleoid | 6 | SGMPTKAGTKSTANK |
| BA71V-K78R (P10) | BA71V-K78R (p10) | Nucleoid | 7 | GMPTKAGTKSTANKK |
| BA71V-K78R (P10) | BA71V-K78R (p10) | Nucleoid | 8 | MPTKAGTKSTANKKT |
| BA71V-K78R (P10) | BA71V-K78R (p10) | Nucleoid | 9 | PTKAGTKSTANKKTT |
| BA71V-K78R (P10) | BA71V-K78R (p10) | Nucleoid | 10 | TKAGTKSTANKKTTK |
| BA71V-K78R (P10) | BA71V-K78R (p10) | Nucleoid | 11 | KAGTKSTANKKTTKG |
| BA71V-K78R (P10) | BA71V-K78R (p10) | Nucleoid | 12 | AGTKSTANKKTTKGP |
| BA71V-K78R (P10) | BA71V-K78R (p10) | Nucleoid | 13 | GTKSTANKKTTKGPS |
| BA71V-K78R (P10) | BA71V-K78R (p10) | Nucleoid | 14 | TKSTANKKTTKGPSK |
| BA71V-K78R (P10) | BA71V-K78R (p10) | Nucleoid | 15 | KSTANKKTTKGPSKS |
| BA71V-K78R (P10) | BA71V-K78R (p10) | Nucleoid | 16 | STANKKTTKGPSKSG |
| BA71V-K78R (P10) | BA71V-K78R (p10) | Nucleoid | 17 | TANKKTTKGPSKSGS |
| BA71V-K78R (P10) | BA71V-K78R (p10) | Nucleoid | 18 | ANKKTTKGPSKSGSA |
| BA71V-K78R (P10) | BA71V-K78R (p10) | Nucleoid | 19 | NKKTTKGPSKSGSAK |
| BA71V-K78R (P10) | BA71V-K78R (p10) | Nucleoid | 20 | KKTTKGPSKSGSAKG |
| BA71V-K78R (P10) | BA71V-K78R (p10) | Nucleoid | 21 | KTTKGPSKSGSAKGH |
| BA71V-K78R (P10) | BA71V-K78R (p10) | Nucleoid | 22 | TTKGPSKSGSAKGHT |
| BA71V-K78R (P10) | BA71V-K78R (p10) | Nucleoid | 23 | TKGPSKSGSAKGHTG |
| BA71V-K78R (P10) | BA71V-K78R (p10) | Nucleoid | 24 | KGPSKSGSAKGHTGK |

| | | | | |
|---|---|---|---|---|
| BA71V-K78R (P10) | BA71V-K78R (p10) | Nucleoid | 25 | GPSKSGSAKGHTGKT |
| BA71V-K78R (P10) | BA71V-K78R (p10) | Nucleoid | 26 | PSKSGSAKGHTGKTH |
| BA71V-K78R (P10) | BA71V-K78R (p10) | Nucleoid | 27 | SKSGSAKGHTGKTHA |
| BA71V-K78R (P10) | BA71V-K78R (p10) | Nucleoid | 28 | KSGSAKGHTGKTHAT |
| BA71V-K78R (P10) | BA71V-K78R (p10) | Nucleoid | 29 | SGSAKGHTGKTHATA |
| BA71V-K78R (P10) | BA71V-K78R (p10) | Nucleoid | 30 | GSAKGHTGKTHATAL |
| BA71V-K78R (P10) | BA71V-K78R (p10) | Nucleoid | 31 | SAKGHTGKTHATALH |
| BA71V-K78R (P10) | BA71V-K78R (p10) | Nucleoid | 32 | AKGHTGKTHATALHQ |
| BA71V-K78R (P10) | BA71V-K78R (p10) | Nucleoid | 33 | KGHTGKTHATALHQG |
| BA71V-K78R (P10) | BA71V-K78R (p10) | Nucleoid | 34 | GHTGKTHATALHQGM |
| BA71V-K78R (P10) | BA71V-K78R (p10) | Nucleoid | 35 | HTGKTHATALHQGML |
| BA71V-K78R (P10) | BA71V-K78R (p10) | Nucleoid | 36 | TGKTHATALHQGMLY |
| BA71V-K78R (P10) | BA71V-K78R (p10) | Nucleoid | 37 | GKTHATALHQGMLYK |
| BA71V-K78R (P10) | BA71V-K78R (p10) | Nucleoid | 38 | KTHATALHQGMLYKD |
| BA71V-K78R (P10) | BA71V-K78R (p10) | Nucleoid | 39 | THATALHQGMLYKDM |
| BA71V-K78R (P10) | BA71V-K78R (p10) | Nucleoid | 40 | HATALHQGMLYKDMV |
| BA71V-K78R (P10) | BA71V-K78R (p10) | Nucleoid | 41 | ATALHQGMLYKDMVN |
| BA71V-K78R (P10) | BA71V-K78R (p10) | Nucleoid | 42 | TALHQGMLYKDMVNI |
| BA71V-K78R (P10) | BA71V-K78R (p10) | Nucleoid | 43 | ALHQGMLYKDMVNIA |
| BA71V-K78R (P10) | BA71V-K78R (p10) | Nucleoid | 44 | LHQGMLYKDMVNIAK |
| BA71V-K78R (P10) | BA71V-K78R (p10) | Nucleoid | 45 | HQGMLYKDMVNIAKS |
| BA71V-K78R (P10) | BA71V-K78R (p10) | Nucleoid | 46 | QGMLYKDMVNIAKSK |
| BA71V-K78R (P10) | BA71V-K78R (p10) | Nucleoid | 47 | GMLYKDMVNIAKSKG |
| BA71V-K78R (P10) | BA71V-K78R (p10) | Nucleoid | 48 | MLYKDMVNIAKSKGI |
| BA71V-K78R (P10) | BA71V-K78R (p10) | Nucleoid | 49 | LYKDMVNIAKSKGIP |
| BA71V-K78R (P10) | BA71V-K78R (p10) | Nucleoid | 50 | YKDMVNIAKSKGIPI |
| BA71V-K78R (P10) | BA71V-K78R (p10) | Nucleoid | 51 | KDMVNIAKSKGIPIY |
| BA71V-K78R (P10) | BA71V-K78R (p10) | Nucleoid | 52 | DMVNIAKSKGIPIYQ |
| BA71V-K78R (P10) | BA71V-K78R (p10) | Nucleoid | 53 | MVNIAKSKGIPIYQN |
| BA71V-K78R (P10) | BA71V-K78R (p10) | Nucleoid | 54 | VNIAKSKGIPIYQNG |
| BA71V-K78R (P10) | BA71V-K78R (p10) | Nucleoid | 55 | NIAKSKGIPIYQNGS |
| BA71V-K78R (P10) | BA71V-K78R (p10) | Nucleoid | 56 | IAKSKGIPIYQNGSR |
| BA71V-K78R (P10) | BA71V-K78R (p10) | Nucleoid | 57 | AKSKGIPIYQNGSRL |
| BA71V-K78R (P10) | BA71V-K78R (p10) | Nucleoid | 58 | KSKGIPIYQNGSRLT |
| BA71V-K78R (P10) | BA71V-K78R (p10) | Nucleoid | 59 | SKGIPIYQNGSRLTK |
| BA71V-K78R (P10) | BA71V-K78R (p10) | Nucleoid | 60 | KGIPIYQNGSRLTKS |
| BA71V-K78R (P10) | BA71V-K78R (p10) | Nucleoid | 61 | GIPIYQNGSRLTKSE |
| BA71V-K78R (P10) | BA71V-K78R (p10) | Nucleoid | 62 | IPIYQNGSRLTKSEL |
| BA71V-K78R (P10) | BA71V-K78R (p10) | Nucleoid | 63 | PIYQNGSRLTKSELE |
| BA71V-K78R (P10) | BA71V-K78R (p10) | Nucleoid | 64 | IYQNGSRLTKSELEK |
| BA71V-K78R (P10) | BA71V-K78R (p10) | Nucleoid | 65 | YQNGSRLTKSELEKK |
| BA71V-K78R (P10) | BA71V-K78R (p10) | Nucleoid | 66 | QNGSRLTKSELEKKI |
| BA71V-K78R (P10) | BA71V-K78R (p10) | Nucleoid | 67 | NGSRLTKSELEKKIK |
| BA71V-K78R (P10) | BA71V-K78R (p10) | Nucleoid | 68 | GSRLTKSELEKKIKR |
| BA71V-K78R (P10) | BA71V-K78R (p10) | Nucleoid | 69 | SRLTKSELEKKIKRS |
| BA71V-K78R (P10) | BA71V-K78R (p10) | Nucleoid | 70 | RLTKSELEKKIKRSK |
| BA71V-K78R (P10) | BA71V-K78R (p10) | Nucleoid | 71 | LTKSELEKKIKRSKG |
| BA71V-K78R (P10) | BA71V-K78R (p10) | Nucleoid | 72 | TKSELEKKIKRSKGS |
| BA71V-K78R (P10) | BA71V-K78R (p10) | Nucleoid | 73 | KSELEKKIKRSKGSG |
| BA71V-K78R (P10) | BA71V-K78R (p10) | Nucleoid | 74 | SELEKKIKRSKGSGS |
| BA71V-K78R (P10) | BA71V-K78R (p10) | Nucleoid | 75 | ELEKKIKRSKGSGSG |
| BA71V-K78R (P10) | BA71V-K78R (p10) | Nucleoid | 76 | LEKKIKRSKGSGSGS |
| BA71V-K78R (P10) | BA71V-K78R (p10) | Nucleoid | 77 | EKKIKRSKGSGSGSG |
